# A survey of putative secreted and transmembrane proteins encoded in the *C. elegans* genome

**DOI:** 10.1186/1471-2164-13-333

**Published:** 2012-07-23

**Authors:** Jinkyo Suh, Harald Hutter

**Affiliations:** 1 Department of Biological Sciences, Simon Fraser University, Burnaby, BC, Canada

**Keywords:** *Caenorhabditis elegans*, Secretome, Transmembrane proteins, Genome, Protein domain, Signal peptide

## Abstract

**Background:**

Almost half of the *Caenorhabditis elegans* genome encodes proteins with either a signal peptide or a transmembrane domain. Therefore a substantial fraction of the proteins are localized to membranes, reside in the secretory pathway or are secreted. While these proteins are of interest to a variety of different researchers ranging from developmental biologists to immunologists, most of secreted proteins have not been functionally characterized so far.

**Results:**

We grouped proteins containing a signal peptide or a transmembrane domain using various criteria including evolutionary origin, common domain organization and functional categories. We found that putative secreted proteins are enriched for small proteins and nematode-specific proteins. Many secreted proteins are predominantly expressed in specific life stages or in one of the two sexes suggesting stage- or sex-specific functions. More than a third of the putative secreted proteins are upregulated upon exposure to pathogens, indicating that a substantial fraction may have a role in immune response. Slightly more than half of the transmembrane proteins can be grouped into broad functional categories based on sequence similarity to proteins with known function. By far the largest groups are channels and transporters, various classes of enzymes and putative receptors with signaling function.

**Conclusion:**

Our analysis provides an overview of all putative secreted and transmembrane proteins in *C. elegans*. This can serve as a basis for selecting groups of proteins for large-scale functional analysis using reverse genetic approaches.

## Background

More than ten years ago the draft sequence of the *Caenorhabditis elegans* genome was published [1]. At that time the genome was predicted to contain approximately 19,000 genes. Gene predictions have been refined over the past decade and now the majority of *C. elegans* genes have been experimentally verified. The release 210 of WormBase ( http://www.wormbase.org) contains 20,242 protein-coding genes, which is close to the initial estimate. Most vertebrate genomes contain a comparable number of genes illustrating that anatomical complexity does not correlate well with the number of genes encoded in the genome. This raises the question of why an anatomically simple animal such as *C. elegans* has such a large number of genes. The *C. elegans* genome contains several large gene families, e.g. more than 1,300 genes encoding serpentine-type G-protein coupled receptors (putative chemoreceptors) [2], 326 F-box proteins [3], 278 C-type lectins [4], 284 nuclear hormone receptors [5] and more than 170 cuticular collagens [6]. These and other families contain large nematode-specific subfamilies, but altogether they correspond to a small fraction of the genes encoded in the genome. The TreeFam project groups 16,866 *C. elegans* genes into 7,471 families [7], pointing to a wide variety of different genes present in the *C. elegans* genome. To date only a fraction of the *C. elegans* genes have been functionally characterized and we are still fairly ignorant of the role that the majority of genes play in the life of *C. elegans*.

The domain organization of a protein provides clues to its putative biochemical function. The biological function, however, is not immediately clear from the domain structure. Additional data, such as gene expression and phenotypes associated with mutations in the corresponding genes, provide further insight into potential biological functions. Although large-scale expression studies have been published [8,9], we still lack a detailed knowledge of the expression of most genes. Large-scale projects to generate mutations in every *C. elegans* gene (reviewed in [10]) currently provide mutations in less than half of the genes. For most genes we are left with the domain structure of the protein as the primary (and often only) source of information for a putative function.

The large number of genes in the *C. elegans* genome poses a significant challenge for geneticists employing genome-scale approaches to identify genes of interest. For this study, we examined putative secreted and transmembrane proteins with the goal of providing an overview and practical groupings of proteins as well as educated guesses for putative functions based on selected genome-scale expression data. This work will allow researchers to select and focus on subsets of the genome for further analysis thereby increasing efficiency of large-scale reverse genetic experiments.

## Results

### General characteristics of putative secreted and transmembrane proteins

According to predictions in Wormbase release 210, 5,676 *C. elegans* genes encode proteins with a signal peptide (SP), i.e. proteins likely to enter the secretory pathway through the endoplasmic reticulum (ER). 5,458 proteins are predicted to have a transmembrane (TM) domain. The majority of those (3,539) lack a SP. In total 9,215 genes encode proteins with either a SP or a TM domain (see Additional file 1 for a full list and Additional file 2 for the corresponding RNAi clones).

3,757 SP-containing proteins do not contain a TM domain and are therefore either secreted or reside in various subcellular compartments originating from the ER. Direct experimental evidence for a subcellular localization does not exist for the overwhelming majority of *C. elegans* proteins. To identify the putative subcellular locations of *C. elegans* proteins, we examined homologs of yeast and mouse proteins for which experimental evidence was available. We focused on proteins residing in the ER, the Golgi apparatus, and various vesicular compartments originating from the ER (see Materials and Methods for details and Additional file 3 and Additional file 4 for the corresponding RNAi clones). We examined the resulting list of proteins and removed known extracellular proteins, as these proteins are known to pass through the secretory pathway and are therefore only transiently present in the ER or Golgi. These primary analyses resulted in 207 proteins that likely reside in one of the endomembrane compartments originating from the ER. In addition, we found that 66 proteins were predicted to be mitochondrial. As this group contained *bona fide* mitochondrial proteins, the entire group was classified as potentially mitochondrial and removed from the original list of 3,757 SP-containing proteins. The remaining 3,484 proteins, about 17% of the proteome, are considered in this study as putative secreted proteins.

The second group of proteins consisted of 5,458 proteins with one or more transmembrane domains (TM). 3,539 of those proteins did not contain a signal peptide. Experimental evidence for the localization of yeast and mouse homologs was used as an indicator for putative localization of *C. elegans* transmembrane proteins. In this way, we were able to assign a total of 481 transmembrane proteins to one or more organelles such as mitochondria, ER, Golgi, endosomes, lysosomes or peroxisomes. The overwhelming majority, 4,977 proteins, lacked any predicted subcellular localization. A substantial fraction of these proteins (43%) also lacked any recognizable domain, preventing functional predictions based on the protein sequence itself.

**Figure 1:**
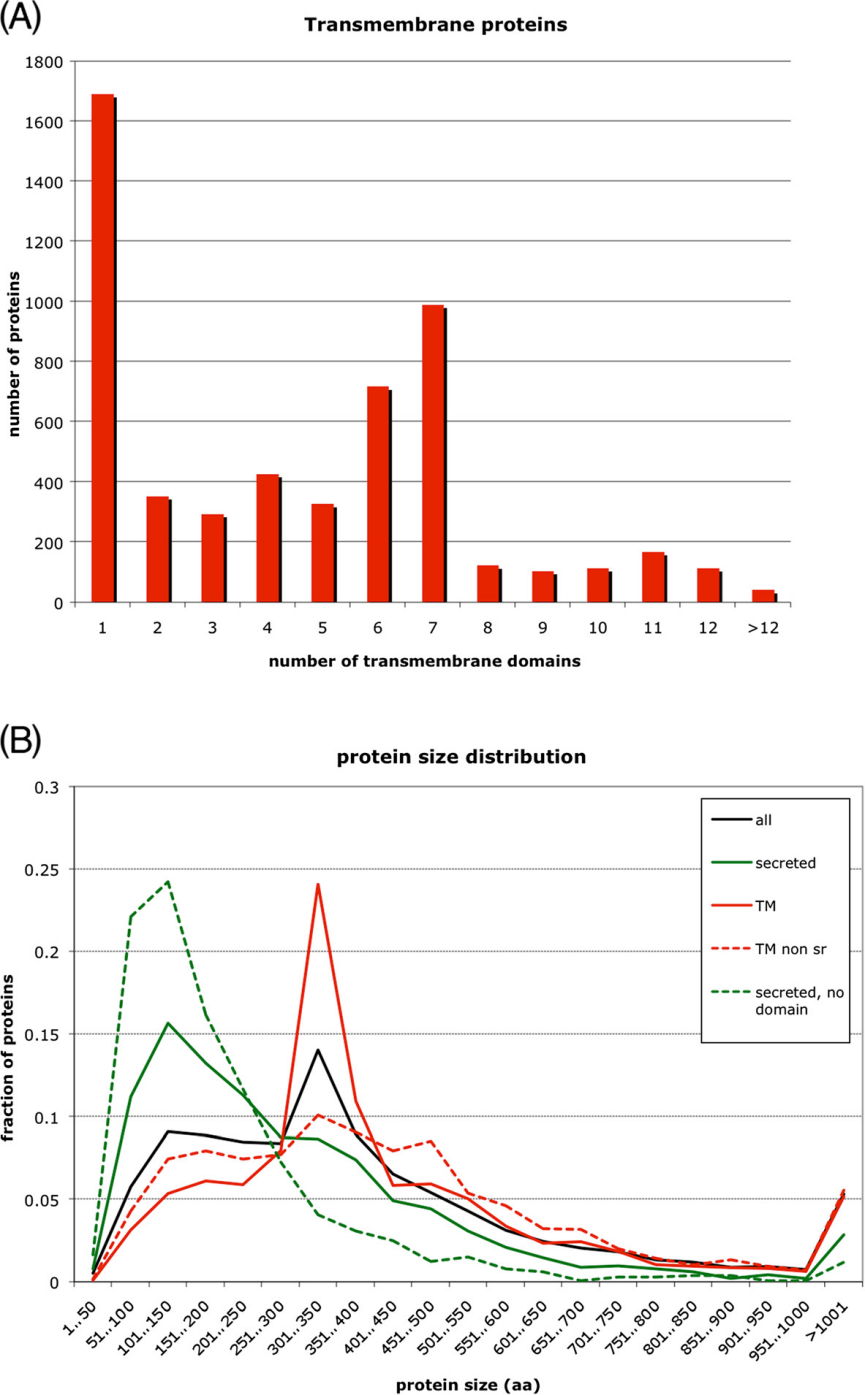
**The general characteristics of secreted and transmembrane proteins.** (**A**) Distribution of transmembrane proteins according to number of TM domains. (**B**) Size distribution of secreted and transmembrane proteins. “all” refers to all secreted or transmembrane proteins. “secreted” refers to putative secreted proteins. “secreted, no domain” refers to putative secreted proteins without additional domains. “TM” refers to transmembrane proteins. “TM non sr” refers to all transmembrane proteins except serpentine-type GPCRs.

When TM proteins were grouped based on the number of TM domains (Figure 1A), we observed almost 1,700 proteins with a single TM domain and a comparable number with six or seven TM domains. The latter group contained 1,469 putative chemoreceptors including a significant fraction where six rather than seven TM domains are predicted. With some notable exceptions, the group of single pass TM proteins contained most of the known receptors for signaling molecules as well as all the families of known adhesion molecules (IgCAMs, cadherins, etc.). We examined this group of proteins further to identify additional potential receptor families. We removed all proteins known to have non-receptor functions and enzymes like proteases or glucosyltransfereases. In addition, we removed proteins expected to localize to certain organelles (see above and Additional file 3). The resulting list contained 1,208 proteins, which could be located at the cell surface. This group likely contains uncharacterized proteins acting in cell-cell communication and/or cell adhesion.

Upon examination of the size distribution of secreted and transmembrane proteins, we observed that smaller proteins are strongly overrepresented within the putative secreted proteins, whereas large proteins are underrepresented (Figure 1B). This effect becomes more pronounced for proteins lacking recognizable domains. Among transmembrane proteins, small proteins (<200 aa) are underrepresented while proteins in a size range of 300–400 aa are strongly overrepresented. The overrepresentation of this size group, however, is due to the fact that 1,469 putative chemoreceptors fall into this size range. Exclusion of this group generates a size distribution of TM proteins that is very similar to the overall size distribution.

Taken together, 9,215 proteins, almost half of the genome, contain either a signal peptide or at least one transmembrane domain. Currently the vast majority of these proteins have not yet been functionally characterized.

### Evolutionary origin of secreted and transmembrane proteins

**Figure 2:**
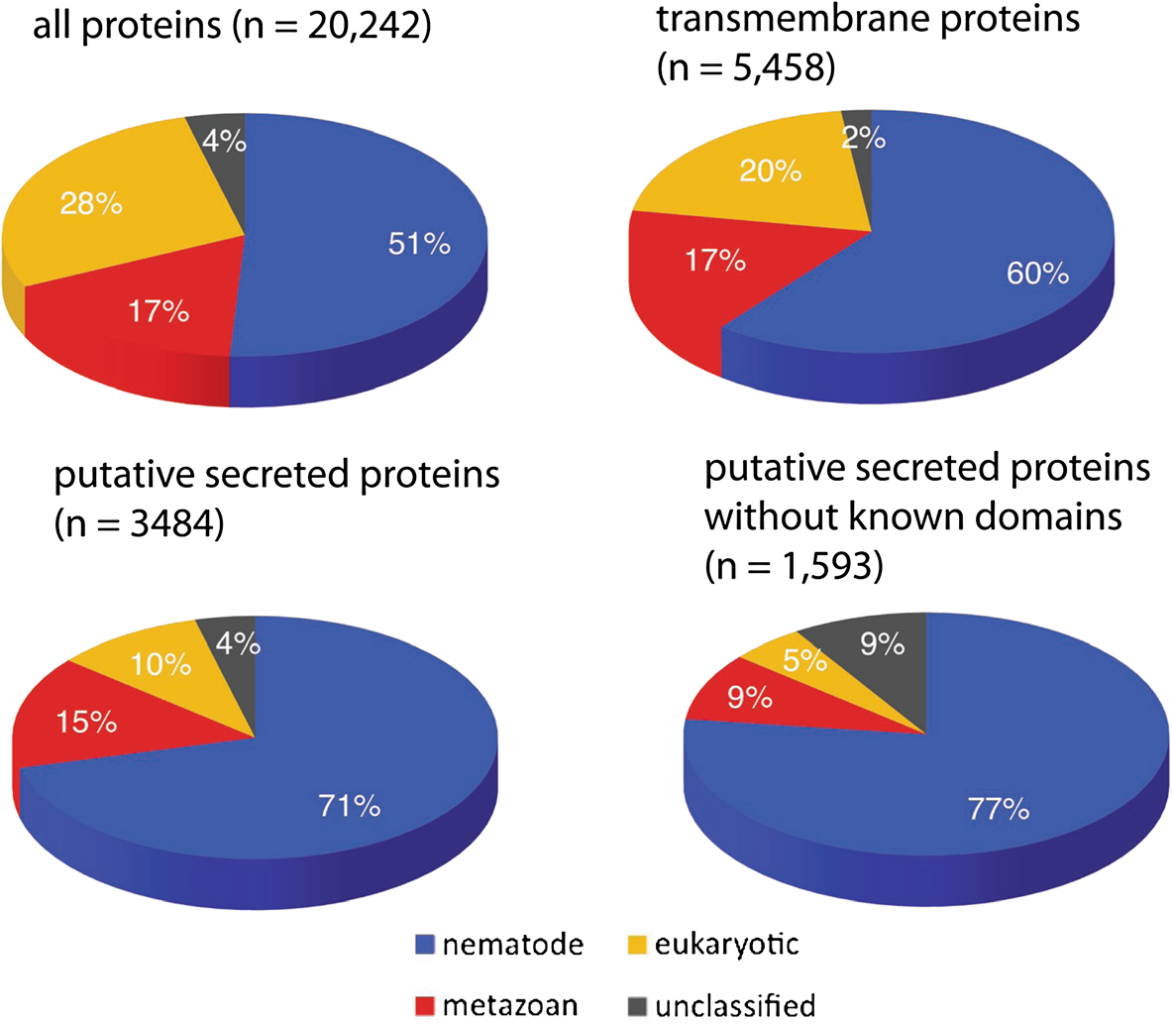
**Evolutionary origin of secreted and transmembrane domain proteins.** Proteins in the ‘nematode’ group have no homologs outside the nematodes. The ‘metazoan’ group consists of proteins with homologs in invertebrate and vertebrates, but not in plants or fungi. The ‘eukaryotic’ group contains proteins with homologs in all the above groups. The ‘unclassified” group contain proteins that could not be grouped (see Materials and Methods for details).

Algorithms are now available to group evolutionarily related proteins based on sequence similarities (see [11,12] for recent reviews). We applied TreeFam [7] and Inparanoid [13] to classify *C. elegans* genes broadly as nematode-specific (not found outside nematodes), of metazoan origin (found in animals, but not in unicellular eukaryotes or plants) or eukaryotic (found in all the above and possibly in prokaryotes as well). Both transmembrane proteins and putative secreted proteins show enrichment in nematode-specific genes in comparison to the overall distribution of proteins (Figure 2). The effect is more pronounced for putative secreted proteins with no known domain. Similarly among TM proteins, most putative chemoreceptors and proteins with no other predicted domains are not found outside nematodes (1,391 out of 1,469 and 1,002 out of 1,309, respectively). In contrast, proteins with recognizable domains are predominantly of metazoan or eukaryotic origin (2,025 out of 2,680) suggesting that a large number of secreted and TM proteins in the *C. elegans* genome have evolved after the split of major animal phyla and probably serve nematode-specific functions.

### Complex and repetitive secreted proteins

**Figure 3:**
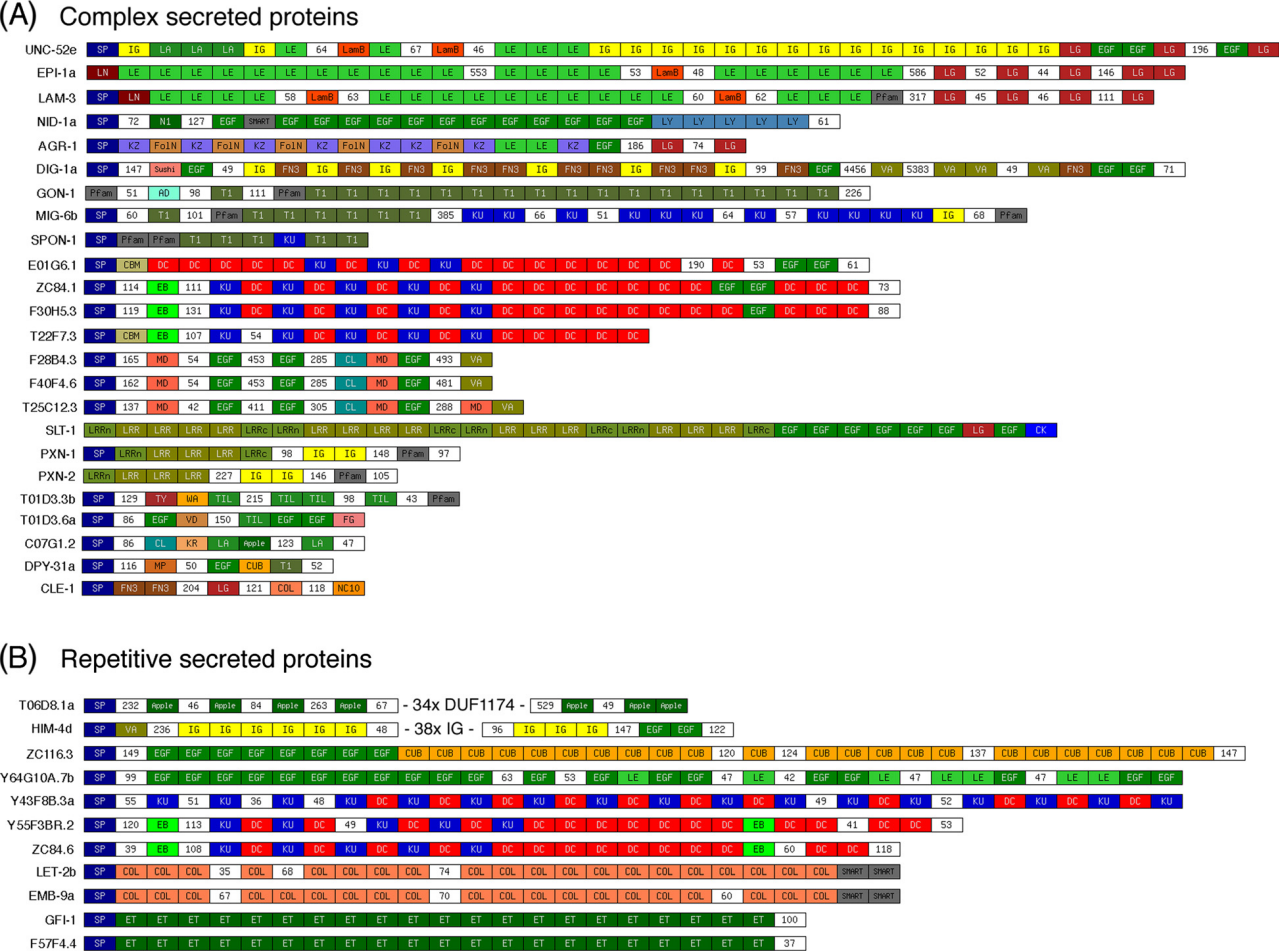
**Complex and repetitive secreted proteins.** (**A**) Complex secreted proteins with four or more different domains. (**B**) Repetitive secreted proteins containing multiple copies of one or two different domains. See the domain help page in GExplore ( http://genome.sfu.ca/gexplore) for a description of the domains.

We surveyed domain organization of putative secreted proteins and found a total of 453 different domains within this set of proteins (data not shown). However, only 10% of these proteins contain two or more domains, indicating these proteins have a fairly simple domain organization. Only 25 proteins contain four or more different domains (Figure 3). Among those are well-known basement membrane components including laminin, nidogen, perlecan and agrin. This group also contains a number of uncharacterized proteins, some of which share a common domain organization. Three large proteins with over 2,000 amino acids (F28B4.3, F40F4.6 and T25C12.3) contain EGF (epidermal growth factor), MD (a domain of unknown function), VA (von Willebrand factor, type A domain) and CL (C-type lectin) domains in a characteristic arrangement. These genes were observed to be upregulated upon exposure to pathogens in several expression profiling studies [14,15] suggesting a role in immune response. Another group of complex proteins (E01G6.3, F30H5.3, T22F7.3, ZC84.1) are characterized by the presence of several DC (Double Cysteine) domains alternating with Kunitz protease inhibitor domains (KU). These proteins are enriched in embryos, most notably in late stages. This enrichment is shared with core basement components such as laminin subunits and nidogen, making it tempting to speculate that large DC-containing proteins are basement membrane components.

The majority of the putative secreted proteins contain only a small number of domains. Only 52 proteins contain more than ten domains and only 21 contain more than 20 domains. Eleven secreted proteins contain multiple copies of one or two different domains (Figure 3) rather than a variety of different domains (see above for complex proteins). Among the highly repetitive secreted proteins are extracellular matrix components like *emb-9*, *let-2* or *him-4*. Three additional proteins (Y43F8B.3, Y55F3BR.2, ZC84.6) have a combination of DC and KU domains and are either strongly (ZC84.6) or moderately (Y43F8B.3, Y55F3BR.2) enriched in embryos raising the possibility that they are also part of the extracellular matrix. RNAi experiments indicate that all three genes are essential for survival [16-18], further supporting this idea.

### Large families of secreted proteins

We used the presence of certain domains or domain combinations in combination with TreeFam annotations to group the putative secreted proteins into families. Previously described protein families such as insulins, collagens or lectins are included to provide a comprehensive picture (Table 1). Most of the predicted families, even well characterized families such as the insulin family, contain some members without predicted signal peptides (Table 1, Additional file 5 and Additional file 6 for the corresponding RNAi clones). We included members without signal peptides in our counts, as these are potentially secreted as well. By defining ‘large families’ as having ten or more members, we grouped 1,404 putative secreted proteins into 39 families including 270 proteins in 17 families (Table 2 and Additional file 5), which are characterized by a ‘domain of unknown function’ (DUF). These proteins typically lack homologs outside nematodes. In addition to these 1,404 proteins there are 585 genes in 23 families with putative enzymatic functions, such as proteases, lipases and various hydrolases (data not shown). Thus, more than half of the 3,484 putative secreted proteins belong to ‘large’ families. 825 proteins in 14 ‘large’ families contain at least one protein domain found outside nematodes. Proteins containing a C-type lectin (CL) domain comprise the largest of these families. While CL domains occur in 263 proteins with various domain compositions, the majority of C-type lectin proteins are small and contain either one or two CL domains alone or in combination with one of a small number of other domains. A total of 236 proteins fall under this definition of ‘small CL proteins’. The second-largest family is putative cuticular collagens with 168 members. Other large families include 66 proteins with multiple copies of a ‘ShK toxin domain’ (ShK), 56 transthyretin-related proteins and 51 proteins containing ‘Receptor L’ (RcpL) domains. The RcpL domain is the ligand-binding domain of the EGF-receptor (*let-23*), the insulin-receptor (*daf-2*) and potentially in other uncharacterized transmembrane proteins. However, most proteins with an RcpL domain have no recognizable transmembrane domain and probably are not receptors. A total of 227 putative secreted proteins can be classified as small putative signaling molecules or peptides. Included in this group are the warthog (*wrt*), groundhog (*grd*), groundhog-like (*grl*), insulin (*ins*) genes [19,20], *nlp-* and *flp-* genes [21]. In summary, most large families of secreted proteins seem to consist entirely of nematode-specific proteins, or reflect a large nematode-specific expansion of evolutionary older families, such as lectins, collagens and insulins.

**Table 1:** Large families of secreted proteins with known domains

**Group**	**No**^**a**^	**Upregulated upon infection**	**Size range (aa)**		**Typical domain organization**	**Comments**
small C-type lectins	236 (184)	83	120-650	*clec-1*		proteins with a C-type lectin domain
				*clec-66*		
				*clec-254*		
				*clec-55*		
collagens	168 (108)	77	270-500	*dpy-5*		putative cuticular collagens
ShK proteins	66 (52)	26	120-300	*phat-3*		proteins containing multiple copies of the ShK toxin domain
Transthyretin-like	56 (46)	24	120-190	*ttr-1*		transthyretin-related proteins
RcpL-genes	51 (38)	1	300-550	F58E1.4		RcpL is the ligand binding domain in the EGF-receptor (*let- 23*) and the insulin-receptor (*daf- 2*)
small CUBproteins	43 (38)	30	300-600	*dct-17*		many members have no predicted signal peptide
insulins	39 (37)	19	70-130	*ins-1*		insulin-like peptides
*grd*- and *grl*-genes	46 (44)	23	140-450	*grl-1*		groundhog proteins (hedgehog related proteins)
*scl*-genes	28 (27)	5	200-250	*scl-2*		contains *lon-1*
Ctx-genes	29 (24)	9	300-570			proteins containing multiple copies of a cysteine-rich repeat (Ctx)
				*abu-9*		
VOMI proteins	19 (13)	5	100-360	C18D4.4		Vitelline membrane outer layer protein I
large KU/DC proteins	12 (12)	3	920-1800	ZC84.1	(see Figure 3 complex proteins)	proteins containing tandem copies of Kunitz and DC domains
*spp*-genes (SapB)	23 (21)	12	100-400	*spp-1*		saposin-like protein family
*wrt*-genes	10 (10)	6	180-550	*wrt-1*		warthog proteins (hedgehogrelated proteins)

^a^Number includes family members without SP; number of family members with SP are in brackets.

^b^Number of genes upregulated upon at least one infection scenario (see Engelmann et al. 2011).

**Table 2:** Large families of putative secreted proteins with ‘domains of unknown function’

**Family**	**No of genes**	**Upregulated upon infection**^**b**^	**Size range (aa)**	**Typical domain organization**	
DUF23	65 (42)	10	400-600	*bah-1*	
*nsp*-genes	58 (41)	25	35-100	*nspd-3*	
DUF19	51 (42)	14	140-220	C17B7.4	
*nlp*-genes	44 (41)	25	60-180	*nlp-1*	
DUF130	41 (25)	1	140-230	F43C11.2	
DUF13	35 (15)	4	200-350	C13A2.4	
DUF148	32 (31)	9	150-250	C32H11.5	
*flp*-genes	30 (28)	4	66-170	*flp-4*	
CW	28 (21)	0	230-330	F32D8.2	
DUF274	22 (15)	20	340-470	F54E2.1	
small DB proteins	18 (17)	11	130-260	F26G1.9	
DUF229	15 (11)	4	400-700	F32D8.2	
DUF263	13 (11)	3	260-560	R13D7.2	
DUF1647	14 (11)	3	340-410	T15D6.9	
DUF236	13 (10)	0	200-320	F27C1.3	
TF352284^c^	13 (13)	6	120-160	F17E9.2	
DUF316	12 (10)	1	300-750	R01H2.2	
DUF273	12 (7)	0	300-400	F31F4.1	
DUF271	11 (7)	0	350-400	F28G4.4	
DUF268	11 (9)	0	310-380	K04A8.1	
DUF870	11 (8)	3	100-200	ZC239.22	
DUF672	10 (7)	0	270-340	R05A10.8	
DUF1261	10 (10)	9	220-270	F56C9.7	
TF319413^c^	10 (8)	7	123-135	K02E11.5	

^a^Number includes family members without SP; number of family members with SP are in brackets.

^b^Number of genes upregulated upon at least one infection scenario (see Engelmann et al. 2011).

^c^Family defined by TreeFam, no recognizable domains.

### Major groups of transmembrane proteins based on their putative biochemical functions

**Figure 4:**
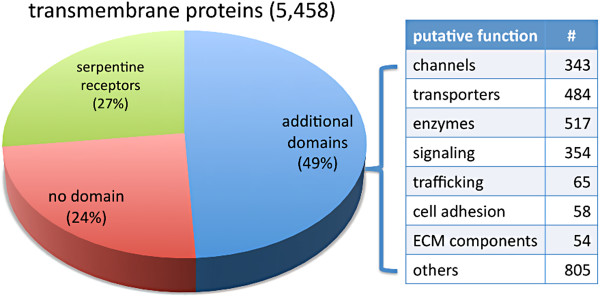
**Grouping of transmembrane proteins.** Three major groups of transmembrane proteins are proteins without any additional predicted domains, proteins with additional domains, and serpentine-type GPCR (putative chemoreceptors). Proteins with additional domains are further divided into broad functional categories (see main text for a detailed description of the categories).

We aimed to classify 5,458 predicted transmembrane proteins according to predicted biochemical functions. 1,469 proteins are members of various nematode-specific expansions of serpentine-type GPCRs, which belong to the class A rhodopsin-like superfamily but are set aside as an “other” group distinct from rhodosin-type or any other hormone-type GPCRs ( http://www.gpcr.org/7tm/). These serpentine-type GPCRs are considered to be putative chemoreceptors and were not further analysed in this study. An additional 1,479 transmembrane proteins lack any predicted domains, but a portion have gene descriptions or GO (Gene Ontology) annotations that provide enough information to sort into functional groups. We took into account recent database updates, which provided additional domain annotations for 170 proteins. No information is presently available for the remaining 1,309 proteins. We classified 2,680 proteins that have additional domain(s) with regard to their putative biochemical functions. Based mainly on domain analysis, and with help from TreeFam and GO annotations (see methods), we defined 8 major groups (Figure 4): 343 channels, 484 transporters, 517 enzymes, 354 signaling proteins, 65 trafficking proteins, 58 cell adhesion proteins, 54 ECM components and 805 “other” proteins. They are composed of subgroups as described below. Representative subgroups are shown in Table 3 and a detailed list of proteins can be found in Additional file 7 (and Additional file 8 for the corresponding RNAi clones).

**Table 3:** Large families of transmembrane proteins with known domains

Group*	**No of genes**	**Pfam ID, SMART ID, reference**		**Typical domain organization**
**(A) Channels**				
Ligand-gated ion channel	102	PF02931, PF02932, *lgc*-*	*unc-29*	
			*lgc-1*	
			
Potassium channel	71	PF07885,	*twk-1*	
		Salkoff et al.		
Sodium channel	46	PF00858,	*nhx-1*	
		PF00999,	*unc-8*	
		PF01699		
Innexin	25	PF00876	*Inx-1*	
**(B) Transporters**				
Major facilitator superfamily MFS-1	117	PF07690	*oct-1*	
ABC transporter,	52	PF00664,	*pgp-1*	
transmembrane region, type 1		PF06472,		
		Zhao et al.	*haf-1*	
Sugar (and other) transporter	43	PF00083	*hmit-1.1*	
Amino acid transporter	36	PF00324,	*aat-1*	
		PF01490	*unc-47*	
**(C) Enzymes**				
Acyltransferase	71	PF01757	*oac-1*	
UDP-glucuronosyl/UDP-glucosyltransferase	67	PF00201	*ugt-1*	
AAA ATPase	50	SM00382	*rpt-1*	
			*wht-1*	
Peptidase	49	see Additional file 7	*nas-17*	
			*bli-4d*	
			*rom-1*	
			*sup-17*	
**(D) Signaling**				
GPCR	143	PF00002,	*dop-1a*	
		PF00003,		
		PF08395		
Kinase	69	PF00069,	*ddr-1*	
		PF07714,	*gcy-1*	
		SM00219	*ver-1*	
		SM00220		
		SM00221		
ANF_receptor	33	PF01094	*nmr-1*	
Guanylate and adenylate cyclase	30	PF00211,	*gcy-1*	
		SM00044		
**(E) Trafficking**				
t-SNARE	10	SM00397	*syn-1*	
**(F) Cell adhesion**				
*clc*-like domain	19	PF07062,	*clc-2*	
		*clc*-*		
Cadherin	12	PF00028,	*cdh-1*	
		SM00112		
**(G) ECM components**				
Cuticlin	34	PF00100,	*cutl-1*	
		SM00241		
Collagen	11	PF01391	*col-47*	
**(H) Others**				
Zinc finger	56	see Additional file 7	*ztf-18*	
Tetraspannin	20	PF00335	*tsp-1*	
F-box	14	PF00646,	*fbxb-11*	
		SM00256,		
		PF07735		

^*^Subgroup names and comments were excerpted or modified from Pfam or InterPro descriptions.

Detailed descriptions for these subgroups are available at Additional file 7.

“Channels” and “Transporters” were grouped based on established definitions [22]. The largest subgroup within channels is ligand-gated ion channels with 102 members, including 29 nicotinic acetylcholine receptors [23], ten ionotropic glutamate receptors as well as glycine, serotonin and GABA receptors. In addition, the *C. elegans* genome contains 71 potassium channels [24] and 46 sodium channels. The largest subgroups within the transporters are 117 major facilitators (PF07690), 52 ABC transporters [25], 43 sugar transporters (PF00083) and 36 amino acid transporters (PF00324, PF01490).

The “Enzymes” group consists of various metabolic enzymes, whereas components of signal transduction pathways such as kinases were placed in the “Signaling” group. 71 O-acyltransferases (PF01757, PF03062, *oac*-genes), 67 UDP-glucuronosyltransferases (PF00201), 50 AAA ATPases (SM00382) and 49 peptidases constitute major groups within the “Enzymes”.

The “Signaling” group consists mainly of well-known receptors, for example 143 GPCRs, 69 kinase receptors, and 30 adenylate and guanylate cyclase receptors (PF00211, SM00044).

Proteins involved in “Trafficking” are composed of a small and diverse family of proteins. Ten t-SNARE proteins, eight synaptobrevins, and five synaptotagmin genes are the major constituents.

“Cell adhesion” proteins were collected following the definition by Cox and Hardin [26]. Cadherins, claudin-like proteins (PF07062) and several immunoglobulin or laminin G domain containing proteins are major families within this group.

“ECM” components are mostly composed of cuticlins and collagens. Eleven collagen proteins are included in this group and ten of which have a TM domain in close proximity to their N-terminus. These collagens may be type II TM proteins that are bound to the plasma membrane and then shed by various proteases as seen in mammalian collagens [27]. A portion of these TM predictions by SMART could be mispredictions of SPs, especially when considering that three proteins (C34F6.2, C34F6.3, F54B11.1) are predicted to have a SP overlapping with a TM domain at their N-terminus according to Wormbase 210. The majority of remaining ECM proteins are cuticlins with a C-terminal TM domain and are expected to be cleaved from the cell surface [6].

A substantial number of proteins (805) do not fit into any of the above categories and also do not form a homogeneous group. Among those proteins are those containing one or more domains, which are not indicative of a particular biochemical or biological function, proteins with a “domain of unknown function”, and proteins, where the fragmentary information available (GO annotation, gene name or description) does not provide sufficient evidence for a conclusive placement into one of the functional groups defined above.

### Putative functions of secreted and transmembrane proteins based on selected genome-wide expression profiles

**Figure 5:**
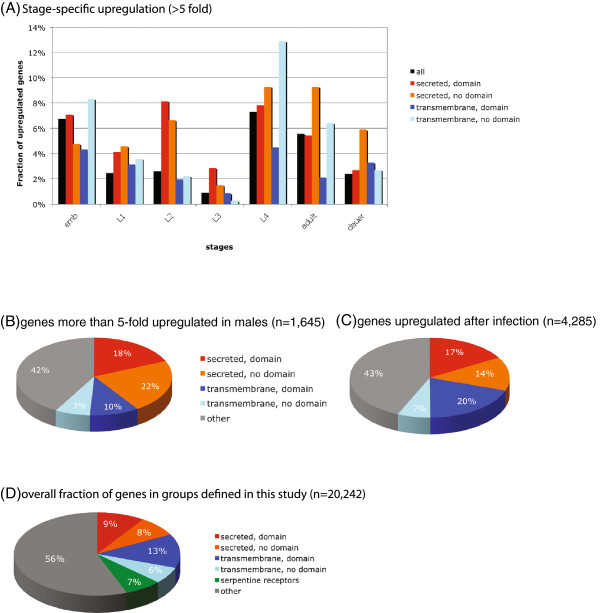
**Proteins upregulated in certain stages, in males or after infection.A**) Genes more than 5-fold upregulated in a particular stage based on RNA-seq data from [14]. **B**) Genes more than 5-fold upregulated in L4 stage males compared to L4 stage hermaphrodites based on RNA-seq data from [14]. **C**) Genes upregulated in at least one of the five infection scenarios used by [28]. **D**) Overall fraction of proteins belonging to the various groups defined in this study.

The overwhelming majority of putative secreted and transmembrane proteins have not been functionally characterized. We examined recently generated stage- and sex-specific expression profiles [14], as well as data sets probing the response to pathogen exposure [28] in an attempt to place putative secreted proteins and transmembrane proteins into broad functional categories. We identified genes that are substantially upregulated in a particular developmental stage (see Material and Methods) or in males compared to hermaphrodites (Figure 5). Data for genes upregulated after pathogen exposure were taken directly from the study by Engelmann et al. [28], which documents expression profiles after three different bacterial infections and two different fungal exposures.

A significant fraction of putative secreted proteins and transmembrane proteins are upregulated in specific stages (Figure 5). Compared to the entire protein data set, a comparatively large number of transmembrane proteins are upregulated in the L4 stage. Putative secreted proteins show a higher proportion of genes upregulated in the L2 stage (Figure 5). Almost 20% of the putative secreted proteins are specifically upregulated in males, suggesting a sex-specific function. While secreted proteins constitute only about 17% of the genome, expression profiles of genes upregulated upon bacterial infections [28] contain 31% secreted proteins. Similarly, profiles of genes upregulated upon exposure to fungal pathogens [28] contain about 37% secreted proteins. In contrast, TM proteins are not overrepresented among the genes upregulated in these infection scenarios. As expected, many signaling proteins (110 of 354) are upregulated upon infection. A substantial fraction of the GPCRs (62 of 143; not including the putative chemoreceptors) and patched receptors (19 of 30) are upregulated upon bacterial infection. Ten of the twelve cadherins and twelve of 23 fatty acid metabolic enzymes (PF01151, PF00487, PF04116) are upregulated as well. Only a small fraction of TM proteins (307 out of 5,458) are up-regulated after fungal exposure including five of twelve ‘fungus induced’ (*fip*) or ‘fungus induced related’ (*fipr*) protein family members. Overall 58% of all proteins upregulated after pathogen exposure have either a signal peptide or a transmembrane domain (Figure 5), indicating putative secreted and cell surface proteins encompass most of the immune response. In total, more than a third of secreted proteins (1,297 out of 3,484) are upregulated in at least one of five pathogen exposure scenarios tested [28], suggesting that a substantial fraction of secreted proteins are part of the nematode’s immune system.

These genome-wide expression profiles also reveal potential functions for some protein families characterized by various ‘domains of unknown function’ (DUF). For example 20 out of 22 DUF274 genes and nine out of ten DUF1261 are among those that are upregulated upon pathogen exposure (Table 1), pointing to a role in the immune response. This analysis also allows a more refined functional characterization of very large families. 83 of the small C-type lectins are upregulated upon pathogen exposure (Table 2), confirming a role for C-lectins in immune response. A comparable number of C-lectins (80) are more than 5-fold upregulated in L4 stage males. Nine of these genes also show a moderate upregulation (3–7 fold) in the L4 stage of hermaphrodites. A similar expression profile is identified in proteins required for sperm production such as ‘major sperm proteins’, suggesting a possible function for this particular group of C-lectins in sperm production. However, the overwhelming majority of C-lectins upregulated in males are expressed at low levels in L4 stage hermaphrodites, suggesting a role in males unrelated to the production of sperm. Only a small number of C-lectins (4–18) are upregulated in particular developmental stages, signifying a lack of stage-specific functions. Eleven of 66 ShK-proteins are upregulated in males and 26 members are upregulated after pathogen exposure, indicating this family may have a similar range of functions as C-lectins. 15 of the 58 nematode-specific peptides (*nsp*-genes) are also more than 5-fold upregulated in males. Notably, six of ten *nspa*-genes and eight of ten *nspd*-genes belong to this group, suggesting a male-specific role for these two families in particular.

The WSN domain (Worm-Specific N-terminal domain, another domain of unknown function found in nematodes) is present in 43 TM proteins. We found that 34 of these proteins are specifically upregulated in hermaphrodites in the L4 stage. Most were also highly expressed in L4 stage males raising the possibility that many WSN-proteins are involved in early germline or sperm development. UDP-glucuronosyltransferases (PF00201) are known to be involved in detoxification in mammals by adding glucosyl-groups to hydrophobic molecules, thereby allowing solubilization and secretion of toxic molecules. In *C. elegans*, we found these enzymes are expressed throughout all developmental stages. 41 of 67 TM domain-containing UDP-glucuronosyltransferases are upregulated after pathogen exposure, suggesting involvement in immune response. Seven of twelve cadherins are upregulated in embryos, implying involvement in early developmental processes, which has been shown previously for *cdh-4*[29], *fmi-1*[30] and *hmr-1*[31].

In summary genome-scale expression profiles provide hints at a potential function for a portion of previously uncharacterized gene families. Based on these data we speculate that many putative secreted proteins potentially have stage-specific functions and that a substantial fraction may be part of the nematode’s immune system.

## Discussion

### Defining the secretome

The secretome is defined as the set of secreted proteins found in the proteome of an organism, i.e. proteins that reside outside the cell [32]. These proteins are important for cell communication, cell adhesion and interactions with the environment. During development secreted proteins are essential for cell fate specification and cell migrations. Some provide structural support for cells and organs to determine the overall appearance of an animal. Others are essential for cell communication within the organism. In addition secreted proteins mediate responses to environmental stresses such as pathogens. Further still secreted proteins have enzymatic functions that range from degrading food to remodeling the body during life stage transitions. The secretome therefore is of interest to a wide variety of researchers ranging from developmental biologists to immunologists.

Bioinformatic approaches are frequently used to define the ‘secretome’. The prediction of a signal peptide [33], which targets proteins to the secretion pathway, is typically used as a first filter to identify secreted proteins. Various algorithms to predict subcellular localization can be used (see [34-36] for recent reviews) to distinguish *bona fide* secreted proteins from those that reside in various intracellular compartments. However, the ability of current prediction algorithms to accurately assign novel proteins to subcellular localizations is somewhat limited, mainly due to localization and retention signals for endomembrane compartments, in particular ER, Golgi, endosomes, lysosomes or peroxisomes, being poorly understood. Thus, a reliable identification of proteins residing in endomembrane compartments by virtue of sequence features alone is currently impossible. A more conservative approach incorporates experimental evidence indicating the presence of proteins in certain subcellular compartments. Large-scale data sets of this kind are typically generated by proteomic studies, i.e. the analysis of the protein content of a purified subcellular compartment. While these studies provide direct evidence for the presence of a protein in a particular subcellular fraction (to the extent that purification is possible), proteins ‘caught in transit’ in the Golgi or the ER add another level of complexity to the identification of truly resident proteins in those compartments. Indeed, our manual inspection of the organelle data sets revealed several secreted proteins like collagens or *bona fide* cell surface receptors annotated as being present in the Golgi or lysosomes. In the absence of localization information for most *C. elegans* proteins we had to rely on localization information from yeast and mouse homologs. Identical subcellular localization across species cannot be taken for granted, especially within gene families that have expanded independently in the species. Consequently the secretome as defined in this study will contain false positives and negatives, which should be kept in mind. Despite these limitations our analysis suggests that as in other organisms the overwhelming majority of proteins in *C. elegans* with a signal peptide and no transmembrane domain are likely to be secreted and do not reside in subcellular compartments.

A number of protein families are known to be secreted and can be confidently assigned to the secretome, despite the lack of a predicted signal peptide in some members. The *C. elegans* genome contains 39 insulins, two of which lack a predicted signal peptide. This may be due to a failure of the prediction algorithm or a false prediction of the gene structure. Predicting the boundaries of a gene is still a challenging problem and the incorrect prediction of the N-terminus of a protein would almost certainly lead to a failure of signal peptide prediction. In our analyses we found proteins lacking signal peptides in almost all families predicted to be secreted, suggesting that current estimates understate the number of secreted proteins.

Some proteins are exported without passing through the ER and consequently do not have a signal peptide. Several such ‘unconventional’ secretion pathways exist [37,38] although we do not know how widespread their use in *C. elegans* is. Finally, secreted proteins or peptides can also be generated by cleavage of membrane proteins. One of the most prominent examples are the β-amyloid peptides, which are generated by cleavage of APP and thought to be causative agents in the development of Alzheimer’s (see [39] for a recent review). Despite our employment of different strategies to minimize false-positive and -negatives in our secretome data set, it should be noted that defining the ‘secretome’ remains a work in progress.

### Contents of the secretome

Based on our analysis we estimate that approximately 17% of the genome encodes putative secreted proteins. The LOCATE database currently lists approximately 7% of the mouse and human proteins as secreted [40]. 17% of zebrafish and human proteins have been reported to contain a signal peptide [41], as well as 12% of mouse proteins [42], again lower than the corresponding number in *C. elegans* (28% proteins with SP). The PEDANT3 database using TargetP prediction on Ensembl data sets lists 32% of *C. elegans* proteins as being in the secretion pathway and comparable and lower numbers (21%-27%) for *Drosphila melanogaster*, *Tribolium castaneum*, zebrafish, mouse and human proteins [43]. This suggests that the *C. elegans* genome contains a larger proportion of secreted proteins as compared to other invertebrate and vertebrate model organisms. This seems counterintuitive, as *C. elegans* is much simpler anatomically and lacks the sophisticated vertebrate immune system as well as many secreted proteins known to have evolved within vertebrates. Nevertheless, *C. elegans* possesses an elaborate set of secreted proteins illustrating that genetic complexity does not necessarily correlate with anatomical complexity.

We used data from the most recent large-scale study [28] to identify genes potentially involved in immune response. The authors used three bacterial and two fungal pathogens and found surprisingly few genes commonly upregulated upon infection. Bacterial infections elicited a response very different from fungal infections and among any two of the infections there was limited overlap in response. We found that more than a third of the putative secreted proteins are upregulated in at least one of the five infection scenarios covered in this study [28]. Given the limited set of potential pathogens tested in this study, this suggests that a substantial fraction of putative secreted proteins may be involved in immune response.

Several large protein families have been of particular interest among researchers since the genome sequence was made available. Among these are cuticular collagens [6], C-type lectins [44], various families of neuropeptides [21] and known putative signaling molecules such as insulins [19,20], warthog, groundhog and groundhog-related proteins [19,20]. Many families of signaling molecules that are expanded in vertebrates such as TGF-βs or ephrins along with their receptors have small families in *C. elegans*. So, while the overall number of potential signaling molecules seems comparable, vertebrates and nematodes differ in which families of signaling molecules are expanded.

Our analysis shows an enrichment of smaller secreted proteins with a significant fraction less than 100 amino acids. The gene structure for many of these genes is experimentally confirmed by cDNA or RNA-seq expression data [14]. Unfortunately, these smaller secreted proteins are very diverse and often contain no recognizable domain preventing speculation on potential functions. Larger families found within this group consist of peptides like FMRF-like peptides (*flp*-genes), neuropeptide-like proteins (*nlp*-genes) and 5 families dubbed nematode-specific peptides (*nsp*-genes) [21]. It is possible that many of the remaining uncharacterized, small secreted proteins encode a variety of peptide families as well. An analysis of the mouse secretome [42] critically evaluated signal peptide-containing proteins smaller than 100 amino acids. By applying stringent criteria for support of the gene model, the authors excluded 649 of the original 741 sequences as unlikely to be real genes. Among the criteria used were the presence of introns, recognizable domains and the presence of orthologs. Many of the small putative secreted proteins in *C. elegans* that have experimentally confirmed gene structures would fail this stringent test as they do not contain recognizable domains, orthologs and sometimes introns. It is therefore possible that the mouse (and human) genome also may contain a larger number of small, secreted proteins than previously predicted. For this reason it would be worthwhile to revisit this point should additional expression information become available.

We broadly grouped the *C. elegans* proteins into three evolutionary categories, namely ‘nematode-specific’, ‘metazoan’ and ‘eukaryotic’ (not further distinguishing between universal and truly eukaryotic genes). We found that orthology databases such as Treefam or Inparanoid each cover only part of the *C. elegans* genome, so that combining the data from various databases significantly improves coverage. Similar observations were made by Shaye and Greenwald, who recently performed a meta-analysis of orthology prediction programs and assembled a list of *C. elegans* proteins with human orthologs [45]. 96% of the proteins contained in this Ortholist are found in our metazoan or eukaryotic categories, confirming the validity of our classification.

The *C. elegans* secretome contains a number of large families with novel domains of unknown function. Of these many are found exclusively in nematodes and few of them have been characterized thus far. Members of one such family, however, have now been shown to encode enzymes, shedding light onto one of these novel families. *galt-1* (DUF23 family) has been identified as a member of a novel glycosyltransferase family [46]. 24 additional DUF23 proteins have also been added to this group of enzymes based on sequence similarity and phylogenetic analysis. It remains to be seen whether the remaining members of the DUF23 family are a more distantly related group of enzymes with similar function. The function of the remaining DUFs currently remains unknown.

### Transmembrane proteins

Transmembrane proteins regulate cell communication and adhesion and allow controlled exchange of chemicals across membranes. The *C. elegans* genome contains a wide variety of different transmembrane proteins. By far the largest group consists of serpentine-type GPCR receptors [2], a *C. elegans* expansion of GPCRs that are separately grouped as a subset within rhodopsin-type GPCR ( http://www.gpcr.org/7tm/). Serpentine-type GPCR receptors are typically expressed in a subset of sensory neurons and are considered to be chemoreceptors. In contrast to vertebrates, *C. elegans* contains a very small number of chemosensory neurons and yet has four times more GPCRs than vertebrates [47]. Why GPCRs are expanded in *C. elegans* is currently unknown, however, one hypothesis is that *C. elegans* depends to a larger extend on chemosensation, since it lacks visual or auditory systems found in other animals [48].

Almost half of all *C. elegans* transmembrane proteins have additional domains or annotations that allowed for grouping into larger families and broad functional categories using Gene Ontology (GO) annotations. During our analysis, we noticed some anomalies one being an unusually large number of genes (589) tagged with a “lipid_storage” phenotype annotation based on the genome-wide RNAi screen by Ashrafi et al. [49]. This group of genes contains transcription factors and signaling molecules, which are merely indirectly involved in lipid storage. Similarly 168 genes are tagged as being involved in “receptor-mediated endocytosis” based on the large-scale RNAi screen by Balklava et al. [50]. Again, no distinction is made between genes that are directly involved and those, which are indirectly involved. In these instances we validated GO annotations by manual curation, however we could not completely solve this problem as many proteins do not have additional information to support or disregard the individual GO annotations. The subgroups and the data used to create the groupings (Pfam, SMART groups defined by other researchers) are listed in Table 3 and Additional file 7. This format offers a simple way to re-group the proteins using existing groups or subgroups as building blocks and can be updated easily as new data becomes available.

## Conclusion

The *C. elegans* genome is predicted to contain around 20,000 genes. The overwhelming majority of these genes are still uncharacterized. A substantial fraction of the genome encodes putative secreted and transmembrane proteins, which are of interest in the context of studying developmental processes as well as interactions of animal and environment. We grouped all proteins containing either a signal peptide or a transmembrane domain, a total of 9,215 proteins, using a number of criteria including similarity and evolutionary conservation. A substantial fraction of the 3,484 putative secreted proteins seem to be nematode-specific and therefore likely have phylum-specific functions. Putative secreted proteins are enriched for small proteins and proteins with no predicted domains limiting further analysis. Single-pass transmembrane proteins are the largest group of transmembrane proteins containing most of the known receptors for cell-cell communication and cell adhesion. The *C. elegans* genome contains 1,208 single-pass TM proteins with no known or predicted function, a group likely containing unidentified receptors for developmental processes and other functions.

Combined with other information, such as genome-scale expression data sets, our classification system can be utilized to select sets of proteins for targeted functional analysis. As the extensive number of genes in the *C. elegans* genome poses a problem for many labor intensive screens these groupings provide an efficient tool for focusing efforts on likely candidate genes.

## Methods

### Domain analysis

We used SMART [51] and Pfam [52] to establish the domain organization of the *C. elegans* proteins. We used SMART predictions to determine signal peptides and transmembrane domains. In cases where both a signal peptide and a transmembrane domain were predicted at the N-terminus in an overlapping fashion, we considered the protein to have a signal peptide, but no transmembrane domain. Other information for the proteins and their corresponding genes like TreeFam and Inparanoid groupings and GO (Gene Ontology) annotations was extracted from WormBase [53]. The data were assembled in a mySQL database, which could be queried using the web interface GExplore ( http://genome.sfu.ca/gexplore/[54]).

### Identification of organelle proteins

For most *C. elegans* proteins direct experimental evidence for subcellular localization is not available. To identify putative organelle proteins we therefore identified homologs of yeast and mouse organelle proteins, where corresponding experimental evidence exists. Mouse organelle proteins with manual experimental evidence were obtained from QuickGO [55]. Yeast organelle proteins with experimental evidence were obtained from the yeast GO slim data set ( http://www.yeastgenome.org). Inparanoid [13] was used to identify the *C. elegans* homologs of these genes. In case of protein families, where there is more than one *C. elegans* homolog for a given mouse or yeast protein, the possibility exists that family members are located in different subcellular compartments. In the absence of further information we are unable to resolve this issue. The resulting list of putative organelle proteins is available at Additional file 3.

### Phylogenetic grouping

We grouped genes into three categories according to their likely phylogenetic origin. Genes of origin ‘nematoda’ are defined as not having homologs outside the nematodes. ‘metazoan’ origin is defined as having homologs in invertebrate and vertebrates, but not in plants or fungi. ‘eukaryotic’ origin is defined as having homologs in all the above groups. The last group contains genes that are ‘universal’ and found even in prokaryotes. We used data from TreeFam 7.0 [7], Inparanoid 7.0 [13] and best BLAST matches to proteins in other species (data extracted from Wormbase 210) to place proteins into individual groups. We found that TreeFam and Inparanoid assignments generally agree, but that both databases cover only part of the proteome. Since TreeFam covered a larger fraction of the proteome (8,153 proteins are not covered by Inparanoid 7 whereas only 4,650 proteins are not assigned to any Treefam in version 7.0), we started with the TreeFam data in the following way

Genes in the ‘nematoda’ group were defined as having only nematode species and at most one non-nematode species (to allow for an outgroup) in the tree. Genes in the ‘metazoa’ group were defined as having a vertebrate or chordate species, an arthropod species, but no plant or fungi species in the tree. Genes in the ‘eukaryota’ group were defined as having a vertebrate or chordate species, an arthropod species and a plant or fungi species in the tree.

We then used Inparanoid data to group those proteins that remained unclassified. Inparanoid clusters are classified as InP_cae (built solely from *C. elegans*-*C. briggsae* ortholog pairs), InP_met (built from metazoan ortholog pairs) or InP_uni (built from non-metazoan, eukaryotic ortholog pairs) (see http://wiki.wormbase.org/index.php/Glossary_of_terms#inparanoid). Of origin ‘nematoda’ is then simply defined as belonging to an InP_cae cluster, but not to an InP_met or InP_uni cluster, of origin ‘metazoa’ is defined as belonging to an InP_met cluster, but not to an InP_uni cluster and of origin ‘eukaryota’ as belonging to an InP_uni cluster. For the 2,723 proteins that still remained unclassified, we used the best BLAST matches in the following way: nematoda: best BLAST matches contain proteins from *C. remanei*, *C. briggsae* or *P. pacificus*, but not *M. musculus*, *H. sapiens*, *S. cerevisiae* or *A. thaliana*; metazoa: BLAST matches contains proteins from *M. musculus* or *H. sapiens* but not from *S. cerevisiae* or *A. thaliana*; eukaryota: BLAST output contains proteins from *S. cerevisiae* or *A. thaliana.* This approach potentially overestimates the number of ‘metazoan’ or ‘eukaryotic’ genes that are classified solely using the BLAST comparisons, but is conservative in defining genes as nematode-specific. This strategy allowed us to classify the overwhelming majority of the proteins and left us with only 834 unclassified proteins.

### Grouping according to GO annotations

GO annotations use standardized hierarchical vocabulary to describe the function of a gene product. We utilized GO annotations in cases where the domain analysis was more difficult, e.g. for proteins belonging to small families or for individual proteins not belonging to families.

### Expression data

We used quantitative expression data generated by high-throughput sequencing (RNA-seq) and provided by the modENCODE project [14] to determine stage and sex-specifically enriched genes. Expression level data were presented as depth of coverage per base per million reads (dcpm). We only considered genes with expression levels higher than 0.04 dcpm, which has been established as a reasonable threshold for true expression [14]. We calculated enrichment in a particular developmental stage by dividing the expression in that stage by the average of expression in all other stages. For male-specific enrichment we used expression in the L4 hermaphrodite as a reference. Genes more than 5-fold enriched in a particular stage or sex were considered to be substantially upregulated. Lists of genes upregulated after infection were taken from [28] using only the RNA-seq data sets. We applied the same threshold of 0.04 dcpm and only considered genes with expression levels higher than this threshold after infection.

### Grouping of transmembrane proteins in functional categories

Transmembrane proteins were grouped into broad functional categories based on domains known to be involved in specific biochemical functions. We employed various approaches to group these proteins. For clarity, we used “family” to refer to an already existing clan from other databases, e.g. acyltransferase family (PF01757) or metalloprotease family (SM00235), and “group” or “subgroup” to refer to a new clan that we generated. From our analysis described above, we had lists of proteins in each TreeFam family. Starting with TreeFam families containing ten or more members we found a number of families whose biochemical functions were easily predicted as they contained catalytic or critical domains for certain biochemical functions. Each TreeFam family may represent all proteins sharing the domain, or may represent only a subset, which share an additional domain or share a distinct sequence feature within the domain. Therefore we extended our grouping from TreeFam families to include more proteins that shared the same catalytic or critical domains. In this way, most groups with many members were easily grouped. Smaller families with five or more members sharing the same domain were manually examined using GExplore [54] and grouped according to their predicted domain function. In addition we used common gene names that define families for grouping; e.g. *lgc-* genes grouped to ligand gated ion channels. Families with less than five members were not manually inspected. Instead we relied mainly on GO annotations. Finally, we included groups that have been defined by other researchers to provide a complete overview. After the initial grouping, we manually checked and reassigned proteins when there was evidence that the GO terms or other definitions were not correctly assigned.

## Abbreviations

aa: Amino acid; AD: Reprolysin (M12B) family zinc metalloprotease (ADAM); CAM: Cell adhesion molecule; CBM: Chitin binding peritrophin-A domain; CK: Cystine knot; CL: C-type lectin; COL: Collagen; CUB: Domain first found in C1r C1s, uEGF, and bone morphogenetic protein; DC: Double cysteine; dcpm: Depth coverage per million; DUF: Domain of unknown function; EGF: Epidermal growth factor; ER: Endoplasmic reticulum; FG: Fibrinogen; FMRF: Phe-Met-Arg-Phe (abbreviation of 4 amino acids); FN3: Fibronectin type III-like fold; FolN: Follistatin-like N-terminal; GO: Gene ontology; GPCR: G-protein coupled receptor; IG: Immunoglobulin; IgCAM: Immunoglobulin cell adhesion molecule; KR: Kringle; KU: Kunitz protease inhibitor domain; KZ: Kazal proteinase inhibitor I1; LA: Low density lipoprotein-receptor class A domain; LamB: Laminin B domain; LE: Laminin EGF-like domain; LG: Laminin G domain; LN: Laminin N-terminal domain; LRR: Leucine-rich repeat; LRRc: Leucine-rich repeat C-terminal; LRRn: Leucine-rich repeat N-terminal; LY: Low-density lipoprotein receptor YWTD repeat; modENCODE: Model organism ENCODE (the ENCyclopedia Of DNA Elements); MP: Metalloprotease; mySQL: My(name of the founder’s daughter) structured query language; N1: Nidogen N-terminal domain; NC10: Collagenase NC10 and Endostatin; PEDANT: Protein Extraction Description and ANalysis Tool; Pfam: A database of protein families; RcpL: Receptor L domain; SapB: Saposin B; ShK: Stichodactyla toxin; SMART: Simple Modular Architecture Research Tool; SNARE: SNAP (Soluble NSF Attachment Protein) REceptor; SP: Signal peptide; Sushi: Sushi/SCR/CCP domain; T1: Thrombospondin type I; TIL: Trypsin inhibitor like cysteine rich domain; TM: Transmembrane; TreeFam: Tree families database; t-SNARE: Target SNARE; TY: Thyroglobulin type-1 domain; VA: von Willebrand factor type A domain; VD: von Willebrand factor type D domain; VOMI: Vitelline membrane outer layer protein I; WA: Whey acidic protein 4-disulphide core; CW,DB,EB,ET,MD: Nematode-specific domains.

## Competing interests

The authors declare that they have no competing interests.

## Authors’ contributions

JS and HH did the analysis together and wrote the manuscript. All authors read and approved the final manuscript.

## Supplementary Material

Additional file 1 Lists of signal peptide and transmembrane domain containing proteins. Click here for file

Additional file 2 **Table S1.** RNAi clones for genes enlisted in Additional file 1. Click here for file

Additional file 3 Lists of putative organelle proteins. Click here for file

Additional file 4 **Table S2.** RNAi clones for genes enlisted in Additional file 3. Click here for file

Additional file 5 Lists with family members of large secreted protein families. Click here for file

Additional file 6 **Table S3.** RNAi clones for genes enlisted in Additional file 5. Click here for file

Additional file 7 Lists of transmembrane proteins grouped by domain structure. Click here for file

Additional file 8 **Table S4.** RNAi clones for genes enlisted in Additional file 7. Click here for file
