# Supplementary material for: A survey of putative secreted and transmembrane proteins encoded in the C. elegans genome
Source: BMC Genomics. 2012 Jul 23;13:333. doi: 10.1186/1471-2164-13-333 (PMC3534327; doi:10.1186/1471-2164-13-333)
Supplement: Additional file 1 — Lists of signal peptide and transmembrane domain containing proteins. [file 1471-2164-13-333-S1.doc]

**Lists of proteins containing signal peptides (SP) or transmembrane domains (TM)**

The following lists of proteins can be directly used in the gene input field of GExplore (<http://genome.sfu.ca/gexplore/>). You can use the compare function in GExplore (<http://genome.sfu.ca/gexplore/gexplore_search_compare.html>) to create additional subgroups. E.g. to find all proteins with SP and TM copy all SP containing proteins listed below into the ‘Gene set 1’ field in the above-mentioned page and all TM proteins into the ‘Gene set 2’ field. The results page will show you, which genes are found in both sets, i.e. have a SP and a TM, and which ones have only a SP or only a TM.

This file contains the following lists:

| **list** | **no of proteins** | **description** |
| --- | --- | --- |
| all SP containing proteins | 3757 | all proteins containing a predicted signal peptide |
| all putative secreted proteins | 3484 | all SP proteins minus putative organelle proteins (see Suppl_data2.doc) |
| putative secreted proteins without domains | 1593 | subset of the 3484 putative secreted protein without predicted domain |
| all TM proteins | 5458 | all proteins with a predicted transmembrane domain |
| serpentine receptors | 1469 | members of the serpentine G-protein coupled receptor (GPCR) families |
| TM proteins with additional domain | 2680 | TM proteins with at least one additional domain |
| TM proteins with no other domain | 1309 | TM proteins with no predicted domains |

**all SP containing proteins (3757)**

4R79.1, AC3.3, AC3.4, AC3.6, AH6.3, B0001.4, B0019.1, B0024.1, B0024.15, B0024.2, B0034.1, B0034.4, B0035.13, B0047.4, B0205.10, B0205.12, B0205.13, B0205.4, B0207.2, B0213.10, B0213.11, B0213.12, B0213.14, B0213.15, B0213.16, B0213.17, B0213.3, B0213.4, B0213.5, B0213.6, B0218.6, B0218.8, B0222.11, B0222.5, B0222.7, B0222.8, B0228.1, B0228.8, B0238.12, B0238.15, B0238.7, B0250.2, B0252.2, B0252.8, B0261.5, B0280.5, B0280.7, B0285.5, B0285.7, B0294.1, B0310.6, B0331.1, B0334.1, B0334.13, B0344.2, B0361.9, B0365.5, B0365.6, B0379.2, B0379.7, B0393.7, B0393.9, B0403.3, B0403.4, B0403.5, B0410.3, B0412.2, B0416.2, B0416.6, B0416.7, B0432.11, B0432.12, B0432.14, B0454.8, B0457.2, B0464.2, B0478.3, B0491.2, B0496.11, B0507.1, B0507.5, B0511.1, B0511.5, B0513.4, B0524.5, B0545.3, B0554.1, B0554.6, B0563.10, B0563.9, BE0003N10.3, BE10.4, C01A2.7, C01A2.9, C01B10.6, C01B12.1, C01B7.7, C01C4.1, C01F1.5, C01G10.15, C01G10.16, C01G10.17, C01G10.18, C01G10.4, C01G10.5, C01G10.6, C01G12.10, C01G12.11, C01G12.13, C01G12.2, C01G12.6, C01G12.9, C01G5.9, C01G6.3, C01G6.9, C01H6.1, C01H6.8, C02A12.4, C02B10.3, C02B4.1, C02B4.4, C02C2.1, C02C6.3, C02E7.6, C02E7.7, C02F12.3, C02F12.5, C02F4.4, C02F5.14, C03A7.12, C03A7.13, C03A7.14, C03A7.4, C03A7.7, C03A7.8, C03B1.6, C03C11.1, C03E10.4, C03E10.5, C03E10.6, C03G5.10, C03G5.11, C03G5.12, C03G5.13, C03G5.2, C03G5.8, C03G5.9, C03G6.14, C03G6.15, C03G6.5, C03G6.6, C03H5.1, C03H5.4, C04B4.3, C04E12.2, C04E6.13, C04E6.6, C04F1.1, C04F12.5, C04F6.3, C04G2.1, C04G6.10, C04G6.5, C04G6.7, C04H5.2, C04H5.7, C04H5.8, C05B5.1, C05B5.11, C05B5.3, C05C10.1, C05C10.4, C05C10.8, C05C8.3, C05C8.8, C05C9.1, C05D11.6, C05D12.2, C05D12.3, C05D2.8, C05D9.3, C05D9.4, C05D9.9, C05E11.6, C05E11.7, C05E11.8, C05E4.7, C05E7.1, C05E7.2, C05E7.3, C05G5.5, C06A1.6, C06A1.7, C06A12.5, C06A12.8, C06A6.5, C06A8.3, C06B3.3, C06B3.4, C06B3.5, C06B8.2, C06C3.10, C06C3.4, C06C3.9, C06C6.6, C06C6.8, C06C6.9, C06E1.5, C06E1.6, C06E1.7, C06E2.8, C06E2.9, C06E4.2, C06E7.2, C06E7.6, C06E7.88, C06E8.5, C06G1.1, C06G1.2, C07A12.2, C07A12.4, C07A4.1, C07A4.2, C07A9.1, C07B5.3, C07B5.5, C07D10.4, C07E3.10, C07E3.9, C07G1.2, C07G2.1, C07G3.10, C07G3.8, C08A9.10, C08B6.10, C08B6.14, C08B6.3, C08B6.4, C08B6.6, C08E3.14, C08E8.11, C08F11.11, C08F11.12, C08F11.17, C08G5.3, C08G5.4, C08H9.1, C08H9.14, C09B7.2, C09C7.1, C09D4.2, C09E7.2, C09E7.3, C09E8.2, C09G1.5, C09G12.17, C09G12.5, C09G5.4, C09G5.5, C09G5.6, C09G5.8, C09G9.3, C09G9.4, C09G9.8, C09H10.5, C09H5.7, C10C5.7, C10F3.1, C10F3.6, C10F3.7, C10G11.10, C10G8.3, C10G8.4, C11D2.2, C11E4.1, C11E4.2, C11H1.5, C11H1.7, C12D12.1, C12D5.10, C12D5.3, C12D5.5, C12D5.7, C12D5.9, C12D8.15, C12D8.4, C12D8.8, C13A10.2, C13A2.2, C13A2.3, C13A2.4, C13A2.7, C13B9.3, C13C12.2, C13C4.7, C14A4.9, C14A6.1, C14A6.3, C14B1.1, C14B1.9, C14B9.2, C14B9.6, C14C10.7, C14C11.3, C14C11.8, C14C6.2, C14C6.3, C14C6.5, C14C6.6, C14C6.7, C14E2.4, C14E2.6, C14F11.7, C14F5.2, C15A11.3, C15A11.5, C15A11.6, C15B12.4, C15C7.7, C15C8.3, C15C8.4, C15F1.2, C15H11.10, C15H11.13, C15H9.11, C15H9.6, C15H9.9, C16A11.8, C16C8.10, C16C8.2, C16C8.8, C16C8.9, C16D9.1, C16D9.5, C16D9.8, C16D9.9, C16E9.1, C17A2.2, C17A2.7, C17B7.12, C17B7.2, C17B7.4, C17B7.9, C17C3.13, C17C3.18, C17C3.19, C17C3.2, C17C3.20, C17C3.4, C17E7.10, C17F3.3, C17F4.12, C17F4.2, C17F4.3, C17F4.7, C17G1.5, C17G1.6, C17G10.5, C17G10.6, C17H12.11, C17H12.6, C17H12.8, C18A11.2, C18A3.11, C18B10.6, C18B2.1, C18B2.2, C18D1.3, C18D11.9, C18D4.4, C18E9.11, C18F10.2, C18G1.6, C18G1.7, C18H7.1, C18H7.3, C18H7.7, C18H9.1, C23G10.11, C23H3.4, C23H3.7, C23H3.9, C23H5.8, C23H5.9, C24A1.1, C24A11.5, C24A3.2, C24B5.5, C24F3.3, C24G6.6, C24G6.7, C24H12.1, C24H12.10, C24H12.11, C24H12.8, C25A1.8, C25A8.2, C25A8.4, C25B8.3, C25D7.15, C25D7.5, C25E10.10, C25E10.11, C25E10.13, C25E10.2, C25E10.8, C25E10.9, C25F6.6, C25F6.8, C25F9.8, C25G4.1, C25G6.4, C25H3.15, C25H3.5, C26B9.2, C26B9.3, C26B9.5, C26C6.3, C26C6.4, C26D10.6, C26F1.1, C26F1.10, C26F1.2, C26F1.5, C27A2.4, C27A2.5, C27A2.8, C27A7.3, C27A7.8, C27A7.9, C27B7.7, C27B7.9, C27C12.3, C27C12.7, C27D6.12, C27D9.2, C27F2.9, C28C12.1, C28C12.3, C28C12.4, C28C12.5, C28C12.7, C28D4.10, C28H8.2, C28H8.7, C28H8.8, C29E4.1, C29E4.10, C29E4.14, C29E4.17, C29E6.1, C29F3.2, C29F3.3, C29F3.4, C29F3.5, C29F3.7, C29F4.1, C29F5.8, C29F9.11, C29G2.6, C30E1.4, C30E1.8, C30F2.1, C30F2.3, C30F2.4, C30F8.3, C30G12.1, C30G4.3, C30G4.6, C30G7.4, C30H6.1, C30H6.10, C30H6.4, C30H7.2, C31B8.7, C31B8.8, C31B8.9, C31C9.1, C31H1.2, C31H1.5, C31H1.7, C31H2.2, C31H5.7, C32A9.1, C32E12.3, C32E8.12, C32E8.4, C32H11.1, C32H11.10, C32H11.11, C32H11.12, C32H11.3, C32H11.4, C32H11.5, C32H11.6, C32H11.8, C32H11.9, C33A12.15, C33A12.19, C33A12.2, C33B4.5, C33C12.3, C33C12.8, C33G3.4, C33G3.5, C33G8.12, C33G8.13, C33G8.2, C33G8.3, C33G8.4, C33H5.2, C34B4.4, C34B4.7, C34B7.1, C34B7.3, C34C12.7, C34C6.3, C34C6.8, C34D4.10, C34D4.11, C34D4.9, C34E7.4, C34F11.8, C34F6.1, C34F6.4, C34G6.1, C34G6.2, C34H4.1, C34H4.2, C34H4.3, C34H4.4, C35A11.2, C35A11.3, C35A5.10, C35A5.11, C35A5.6, C35B1.4, C35B1.7, C35B8.1, C35C5.3, C35C5.8, C35D10.14, C35D10.15, C35E7.7, C35E7.9, C36A4.1, C36A4.2, C36A4.3, C36A4.6, C36B1.1, C36B1.8, C36B7.5, C36B7.7, C36C5.12, C36C5.14, C36C5.15, C36C5.5, C36F7.5, C36H8.3, C37A5.8, C37C3.13, C37C3.4, C37C3.6, C37C3.8, C37H5.10, C37H5.11, C37H5.4, C38C10.6, C38C3.10, C38C3.6, C38C6.3, C38C6.5, C38C6.6, C38H2.2, C39B10.7, C39B5.14, C39B5.5, C39E6.1, C39E9.2, C39E9.3, C39E9.4, C39E9.5, C39E9.6, C39E9.8, C39E9.9, C40A11.10, C40C9.3, C40H1.7, C40H1.8, C40H1.9, C40H5.1, C40H5.4, C41C4.1, C41C4.3, C41C4.4, C41C4.9, C41G11.1, C41G6.1, C41G6.13, C41G6.6, C41G7.6, C41H7.7, C42C1.15, C42D4.1, C42D4.13, C42D4.3, C42D8.5, C43E11.8, C43F9.11, C43F9.5, C43F9.7, C43F9.8, C43G2.3, C43G2.5, C44B11.4, C44B12.1, C44B12.2, C44B12.3, C44B12.5, C44B12.9, C44B7.5, C44C1.2, C44C1.5, C44C1.6, C44C10.7, C44E4.3, C44E4.8, C44H4.3, C44H9.5, C45B11.6, C45B2.1, C45B2.2, C45B2.3, C45E5.4, C45G7.2, C45G7.3, C45G9.10, C45G9.12, C45G9.13, C45G9.6, C45H4.17, C45H4.2, C46A5.3, C46A5.4, C46C2.5, C46C2.6, C46E10.1, C46F2.1, C46F4.3, C46H11.2, C46H11.7, C46H11.8, C46H11.9, C47D12.4, C47D2.1, C47E12.10, C47E12.14, C47E12.3, C47E12.6, C47E12.9, C47E8.11, C47F8.3, C47F8.4, C47F8.5, C47F8.6, C47F8.7, C48B4.12, C48B4.13, C48D1.5, C48D5.3, C48E7.10, C48E7.7, C49A1.1, C49A1.5, C49A1.6, C49C3.11, C49C3.13, C49C3.20, C49C3.4, C49C3.9, C49C8.4, C49C8.5, C49C8.6, C49D10.10, C49F8.3, C49G7.3, C49G7.4, C49G7.8, C49G9.2, C49H3.12, C50B6.4, C50B6.7, C50C3.9, C50D2.1, C50D2.6, C50D2.7, C50E3.10, C50E3.15, C50E3.2, C50E3.6, C50F2.10, C50F2.4, C50F2.6, C50F2.9, C50F4.3, C50F4.8, C50F7.2, C50F7.5, C50F7.9, C50H11.15, C50H11.17, C50H2.10, C50H2.3, C51E3.10, C51E3.7, C52D10.11, C52D10.13, C52E2.2, C52E2.3, C52E4.5, C52G5.2, C53A5.11, C53A5.2, C53B4.4, C53B4.8, C53B7.1, C53B7.2, C53D6.2, C53D6.4, C53D6.8, C54C6.5, C54C8.11, C54C8.12, C54C8.2, C54C8.3, C54C8.4, C54C8.5, C54C8.6, C54C8.7, C54C8.9, C54D1.2, C54D1.7, C54D10.1, C54D10.10, C54D10.13, C54D10.2, C54D10.3, C54D10.9, C54D2.1, C54D2.2, C54D2.4, C54D2.6, C54E4.2, C54F6.12, C54F6.15, C54F6.3, C54F6.5, C54F6.6, C54G10.4, C54G6.5, C54G7.3, C55A1.4, C55A1.6, C55A1.7, C55A6.12, C55B6.2, C55B7.2, C55C3.1, C55C3.3, C55C3.5, C55F2.2, C56A3.1, C56A3.2, C56C10.4, C56C10.9, C56G2.4, C56G3.2, CC4.2, CD4.11, D1005.3, D1007.13, D1007.14, D1007.18, D1007.19, D1009.4, D1014.1, D1014.5, D1014.6, D1022.2, D1022.8, D1025.4, D1025.6, D1025.7, D1025.8, D1025.9, D1044.7, D1054.10, D1054.11, D1054.9, D1065.3, D1081.10, D1081.12, D1086.1, D1086.10, D1086.11, D1086.12, D1086.17, D1086.18, D1086.2, D1086.3, D1086.5, D1086.6, D1086.7, D1086.8, D1086.9, D2005.2, D2007.1, D2023.6, D2023.7, D2024.4, D2024.8, D2030.1, D2030.2, D2045.9, D2062.13, D2062.6, D2089.3, D2092.8, D2096.1, D2096.10, D2096.13, D2096.3, D2096.4, D2096.5, D2096.6, D2096.9, DY3.5, E01A2.10, E01A2.3, E01A2.7, E01G4.6, E01G6.1, E01H11.3, E02A10.2, E02A10.4, E02C12.13, E02C12.4, E02H4.4, E02H4.7, E02H9.1, E02H9.7, E02H9.9, E03D2.1, E03D2.2, E03E2.1, E03G2.3, E03G2.4, E03H12.4, E03H12.5, E03H12.7, E03H12.9, E03H4.10, E03H4.12, E03H4.2, E03H4.3, E03H4.4, E03H4.5, E04A4.6, E04D5.4, E04D5.5, E04F6.6, E04F6.8, E04F6.9, EEED8.11, EGAP1.3, EGAP4.1, EGAP5.1, EGAP7.1, EGAP9.3, F01D4.4, F01D4.8, F01D4.9, F01D5.1, F01D5.2, F01D5.3, F01D5.5, F01D5.6, F01D5.9, F01F1.15, F01G10.10, F01G10.6, F01G10.7, F01G12.5, F02A9.2, F02A9.3, F02A9.6, F02C12.4, F02C12.5, F02C9.2, F02D10.1, F02D8.2, F02D8.4, F02D8.5, F02E11.5, F02H6.6, F07A5.4, F07B7.13, F07C4.10, F07C4.2, F07C4.6, F07C4.7, F07C4.9, F07D3.2, F07F6.5, F07G11.4, F07G6.10, F07G6.2, F07H5.9, F08A8.5, F08B12.4, F08B4.4, F08B6.3, F08C6.1, F08C6.6, F08D12.2, F08D12.7, F08E10.7, F08F1.6, F08F3.1, F08F3.10, F08F3.7, F08G12.11, F08G12.8, F08G2.6, F08G2.8, F08G5.4, F08G5.6, F08G5.7, F08H9.5, F08H9.6, F08H9.7, F08H9.8, F08H9.9, F09A5.3, F09B12.1, F09B12.5, F09B9.4, F09C6.10, F09C8.1, F09D12.1, F09E10.1, F09E5.14, F09E5.16, F09E5.17, F09E8.1, F09E8.5, F09E8.6, F09E8.8, F09F3.5, F09F3.6, F09F7.8, F09F9.2, F09F9.3, F09G8.2, F09G8.5, F09G8.6, F09G8.8, F10A3.11, F10A3.4, F10C1.3, F10C2.3, F10C2.5, F10D11.6, F10D2.8, F10D7.3, F10E7.4, F10E9.12, F10F2.3, F10F2.8, F10F2.9, F10G2.1, F10G2.2, F10G2.3, F10G7.11, F11A5.12, F11A5.17, F11A5.7, F11A6.1, F11C1.4, F11C7.2, F11C7.3, F11C7.5, F11C7.6, F11C7.7, F11D11.1, F11D11.11, F11D11.14, F11D11.3, F11D11.5, F11D11.6, F11D11.7, F11D11.8, F11E6.1, F11E6.2, F11E6.4, F11E6.9, F11F1.2, F11F1.4, F11F1.5, F11F1.6, F11F1.8, F11G11.14, F11H8.3, F12A10.1, F12A10.7, F12A10.9, F12F6.3, F12F6.9, F13A2.1, F13A2.5, F13A7.12, F13B12.4, F13B12.5, F13B6.1, F13B6.3, F13B9.2, F13C5.3, F13D12.3, F13D12.6, F13D2.4, F13E9.10, F13E9.12, F13E9.14, F13E9.15, F13E9.2, F13E9.3, F13E9.4, F13E9.8, F13E9.9, F13G11.3, F13G3.12, F13G3.3, F13H10.9, F13H8.3, F13H8.5, F14B4.1, F14B6.1, F14B6.4, F14B6.6, F14B8.2, F14B8.4, F14D2.5, F14D2.7, F14D7.10, F14D7.14, F14D7.5, F14D7.7, F14E5.4, F14E5.5, F14E5.6, F14F11.2, F14F3.4, F14F3.5, F14F7.1, F14F7.2, F14F7.3, F14F8.8, F14F9.2, F14F9.5, F14H12.1, F14H12.3, F14H3.14, F14H3.5, F14H8.8, F15A2.1, F15A4.2, F15A4.6, F15B9.1, F15B9.11, F15B9.3, F15B9.5, F15B9.9, F15D4.4, F15D4.8, F15E11.11, F15E11.2, F15E11.4, F15E11.5, F15E6.1, F15E6.10, F15E6.3, F15E6.4, F15G9.4, F15G9.5, F15H10.10, F15H10.5, F15H10.6, F15H10.9, F15H9.1, F16B12.2, F16B3.3, F16B4.4, F16B4.7, F16C3.2, F16C3.4, F16F9.2, F16F9.3, F16F9.4, F16G10.10, F16G10.11, F16G10.13, F16G10.14, F16G10.2, F16G10.3, F16G10.4, F16G10.8, F16G10.9, F16H6.1, F16H6.3, F17B5.10, F17B5.3, F17B5.4, F17B5.5, F17B5.6, F17B5.9, F17C11.2, F17C11.3, F17C11.5, F17C8.2, F17E9.1, F17E9.11, F17E9.15, F17E9.2, F17E9.3, F17E9.4, F18A1.7, F18A12.1, F18A12.3, F18A12.4, F18A12.6, F18C5.5, F18C5.9, F18E2.1, F18E9.2, F18E9.3, F18E9.4, F18E9.7, F18F11.4, F19B10.6, F19C6.3, F19C6.4, F19C7.1, F19C7.2, F19C7.4, F19C7.7, F19F10.4, F19F10.8, F19G12.7, F19H8.2, F19H8.4, F20A1.1, F20A1.10, F20A1.6, F20A1.8, F20B10.3, F20B6.1, F20B6.4, F20C5.7, F20G2.4, F20H11.5, F21A3.2, F21A3.3, F21A3.8, F21C10.11, F21C10.5, F21C10.6, F21C10.8, F21C3.2, F21D12.2, F21D5.3, F21D9.3, F21E9.3, F21E9.4, F21E9.6, F21F3.1, F21F8.3, F21F8.4, F21F8.5, F21F8.6, F21G4.3, F21H12.7, F21H7.10, F21H7.4, F21H7.5, F22A3.2, F22A3.6, F22A3.7, F22B3.5, F22B7.1, F22B7.4, F22D3.4, F22D6.10, F22D6.11, F22D6.12, F22D6.15, F22E12.1, F22E5.1, F22E5.17, F22E5.7, F22F4.1, F23B12.4, F23B2.11, F23B2.12, F23B2.5, F23C8.11, F23D12.7, F23F1.2, F23F1.4, F23F12.12, F23F12.8, F23H11.6, F23H11.7, F23H12.4, F23H12.5, F25A2.1, F25B4.9, F25D1.3, F25E2.2, F25E5.10, F25E5.3, F25E5.4, F25E5.7, F25F6.1, F25G6.5, F25H2.7, F25H8.3, F25H8.5, F25H9.1, F25H9.3, F26A1.12, F26A1.15, F26A1.2, F26A10.1, F26B1.1, F26B1.4, F26B1.8, F26C11.1, F26C11.3, F26C11.4, F26D10.12, F26D10.13, F26D11.2, F26D11.4, F26D11.5, F26D11.9, F26D2.13, F26D2.14, F26D2.16, F26D2.3, F26E4.3, F26E4.7, F26F12.1, F26F12.8, F26F2.10, F26F2.6, F26F2.8, F26G1.1, F26G1.10, F26G1.11, F26G1.5, F26G1.9, F26H9.8, F27B10.1, F27C1.3, F27C1.7, F27C1.8, F27C8.4, F27C8.6, F27E5.1, F27E5.7, F28A10.2, F28A10.5, F28A10.8, F28A10.9, F28A12.2, F28A12.4, F28B1.1, F28B1.2, F28B1.9, F28B3.4, F28B4.3, F28C6.10, F28C6.5, F28D1.3, F28D1.4, F28D1.5, F28E10.5, F28F8.2, F28F9.3, F28G4.2, F28G4.3, F28G4.4, F28H7.3, F28H7.4, F28H7.7, F29A7.3, F29A7.7, F29A7.8, F29B9.5, F29B9.9, F29C12.1, F29C4.2, F29C4.4, F29D10.1, F29D10.2, F29D10.3, F30A10.11, F30A10.13, F30A10.14, F30A10.5, F30B5.1, F30H5.2, F30H5.3, F30H5.5, F31A3.1, F31A9.4, F31C3.1, F31C3.3, F31D4.4, F31D4.6, F31D4.9, F31E8.5, F31F4.1, F31F4.11, F31F6.4, F31F6.7, F31F6.8, F31F7.3, F32A5.2, F32A5.3, F32A5.4, F32A7.6, F32B4.1, F32B4.2, F32B4.5, F32B5.2, F32B5.3, F32B5.8, F32D1.11, F32D1.4, F32D8.11, F32D8.2, F32D8.3, F32D8.7, F32D8.8, F32D8.9, F32E10.3, F32E10.8, F32G8.2, F32G8.3, F32G8.5, F32H2.11, F32H5.1, F32H5.3, F33A8.10, F33A8.2, F33A8.7, F33D11.3, F33D11.8, F33D4.3, F33D4.6, F33E2.3, F33E2.7, F33H1.6, F34D6.7, F34D6.8, F34D6.9, F34H10.2, F35A5.2, F35A5.3, F35A5.4, F35B12.2, F35B12.3, F35B12.4, F35B12.6, F35B12.7, F35B3.4, F35C11.1, F35C11.4, F35C11.7, F35C5.1, F35C5.10, F35C5.12, F35C5.3, F35C5.5, F35C5.6, F35C5.7, F35C5.8, F35C5.9, F35D11.1, F35D11.10, F35D11.8, F35D11.9, F35D2.1, F35D2.2, F35D2.5, F35E12.10, F35E12.2, F35E12.5, F35E12.6, F35E12.7, F35E12.8, F35E2.5, F35E2.8, F35E2.9, F35E8.1, F35E8.10, F35E8.13, F35E8.6, F35E8.7, F35E8.9, F35F10.13, F35F10.5, F35G2.1, F35G2.3, F35G2.4, F35G2.5, F35G8.2, F35H10.2, F35H8.1, F35H8.2, F36A4.1, F36A4.10, F36A4.2, F36A4.4, F36A4.8, F36D1.10, F36D1.12, F36D1.5, F36D1.6, F36D1.7, F36D3.14, F36D3.9, F36F12.1, F36F12.3, F36F12.7, F36F2.7, F36F2.8, F36G9.11, F36G9.15, F36H1.1, F36H1.11, F36H12.1, F36H12.17, F36H12.3, F36H12.4, F36H12.5, F36H2.3, F36H5.6, F36H9.2, F36H9.7, F37A8.4, F37B12.3, F37C12.10, F37C4.8, F37H8.5, F38A1.1, F38A1.10, F38A1.14, F38A1.4, F38A6.4, F38A6.5, F38B2.2, F38B2.6, F38B6.2, F38B6.7, F38C2.6, F38E1.7, F38E11.4, F38E11.7, F38E9.2, F38G1.2, F39C12.4, F39D8.4, F39F10.5, F39G3.2, F39G3.3, F39G3.8, F39H2.1, F40A3.1, F40A3.3, F40C5.1, F40C5.3, F40E10.1, F40E10.3, F40E10.4, F40E10.5, F40F11.4, F40F12.4, F40F4.2, F40F4.3, F40F4.4, F40F4.6, F40F4.8, F40F8.4, F40F9.6, F40G12.2, F40G12.4, F40G12.5, F40G12.6, F40G9.10, F40G9.19, F40G9.5, F40G9.6, F40G9.7, F40H3.2, F40H3.3, F40H3.5, F41B4.2, F41B5.2, F41B5.3, F41B5.4, F41B5.7, F41C3.11, F41C3.5, F41C3.6, F41C6.1, F41C6.3, F41C6.4, F41C6.6, F41D3.11, F41D3.6, F41D3.7, F41D3.9, F41D9.2, F41D9.3, F41E6.11, F41E6.12, F41E6.15, F41E6.2, F41E6.6, F41E6.8, F41E7.4, F41E7.5, F41E7.7, F41E7.8, F41F3.3, F41F3.4, F41F3.8, F41G3.1, F41G3.12, F41G3.16, F41G3.17, F41G3.20, F41G3.21, F42A10.6, F42A10.7, F42A6.2, F42A6.4, F42A8.1, F42A9.4, F42A9.5, F42A9.7, F42C5.7, F42F12.1, F42F12.10, F42F12.2, F42F12.6, F42F12.7, F42F12.8, F42F12.9, F42G10.1, F42G9.2, F42G9.4, F42G9.7, F42H11.1, F43C11.1, F43C11.2, F43C11.3, F43C11.4, F44A2.3, F44A6.1, F44B9.1, F44B9.10, F44C8.1, F44D12.6, F44E7.6, F44F4.15, F45B8.3, F45C12.16, F45C12.9, F45D11.5, F45D3.3, F45D3.4, F45E1.3, F45E12.1, F45E12.6, F45E4.1, F45E4.5, F45E4.8, F45E4.9, F45E6.4, F45G2.1, F45G2.5, F45G2.8, F46A8.3, F46A8.4, F46A8.5, F46A8.8, F46B3.1, F46B3.15, F46B3.17, F46B3.2, F46B3.3, F46B3.4, F46B3.5, F46B3.9, F46B6.10, F46B6.13, F46B6.8, F46C3.6, F46C5.1, F46C5.10, F46C5.3, F46C5.4, F46C5.9, F46C8.1, F46E10.2, F46F11.3, F46F11.7, F46F2.3, F46F5.10, F46F5.11, F46F5.14, F46F5.15, F46F5.7, F46G10.4, F46G11.2, F46G11.4, F46G11.6, F46H5.6, F46H6.2, F47B3.4, F47B7.1, F47B7.3, F47B7.4, F47B8.13, F47B8.14, F47C12.11, F47C12.2, F47C12.4, F47C12.6, F47C12.8, F47E1.1, F47F2.1, F47G3.1, F47G9.6, F48A9.3, F48B9.3, F48B9.4, F48C1.8, F48C1.9, F48C11.3, F48C5.2, F48E3.1, F48E3.3, F48E3.4, F48E8.1, F48E8.4, F48F7.3, F48F7.4, F48G7.7, F48G7.8, F49A5.4, F49A5.6, F49C12.10, F49C12.14, F49C12.5, F49C5.10, F49C5.11, F49C5.7, F49D11.11, F49D11.3, F49D11.6, F49E10.2, F49E10.3, F49E11.10, F49E11.11, F49E11.4, F49E11.5, F49E11.6, F49E12.1, F49E12.2, F49F1.10, F49F1.11, F49F1.5, F49F1.6, F49F1.7, F49F1.9, F49H12.4, F49H6.1, F49H6.12, F49H6.15, F49H6.2, F49H6.8, F52A8.3, F52B10.2, F52B11.4, F52B11.6, F52C9.5, F52D1.1, F52D1.3, F52D2.5, F52E1.14, F52E1.2, F52E4.6, F52F12.2, F52F12.7, F52G3.4, F52G3.5, F52H3.1, F53B1.6, F53B3.2, F53B6.2, F53B6.8, F53B7.7, F53C11.1, F53C11.9, F53E10.3, F53E10.4, F53F1.4, F53F1.5, F53F4.13, F53F4.17, F53F4.7, F53F8.4, F53F8.7, F53G12.9, F53G2.2, F53G2.3, F53H4.2, F53H4.3, F54A5.2, F54B11.10, F54B11.11, F54B11.4, F54B3.4, F54B8.1, F54B8.5, F54C4.2, F54C9.2, F54C9.4, F54D10.8, F54D12.1, F54D12.10, F54D12.11, F54D12.7, F54D12.8, F54D12.9, F54D5.3, F54D5.4, F54D7.4, F54D8.1, F54D8.4, F54E2.1, F54E2.2, F54E4.3, F54E4.4, F54E7.6, F54F11.1, F54F11.2, F54F3.1, F54F3.3, F54F7.2, F54F7.3, F54F7.9, F54G8.1, F54H12.5, F55A11.1, F55A12.6, F55B11.2, F55B11.3, F55B11.4, F55B12.5, F55C10.2, F55C10.3, F55C10.4, F55C12.7, F55C9.6, F55D10.1, F55D12.5, F55F3.2, F55F3.4, F55F8.8, F55G11.2, F55G11.4, F55G11.5, F55G11.6, F55G11.7, F55G11.8, F55H12.4, F55H2.1, F56A11.3, F56A4.1, F56A4.2, F56A4.6, F56A4.9, F56A8.9, F56B3.1, F56B3.2, F56B3.5, F56B3.8, F56B6.6, F56C3.8, F56C4.1, F56C4.4, F56C9.7, F56C9.8, F56D1.4, F56D2.8, F56D3.1, F56D5.1, F56D5.6, F56D6.1, F56D6.15, F56D6.2, F56D6.8, F56D6.9, F56E10.2, F56F10.1, F56F11.1, F56F3.6, F56F4.8, F56F4.9, F56G4.2, F56G4.3, F56G4.7, F56H11.1, F56H6.1, F56H6.13, F56H6.2, F56H6.4, F56H6.6, F56H6.7, F56H6.8, F56H6.9, F56H9.2, F56H9.6, F57A8.6, F57A8.8, F57B7.2, F57B7.3, F57B7.4, F57C12.1, F57C2.4, F57C2.5, F57C9.2, F57E7.1, F57E7.2, F57E7.4, F57F4.1, F57F4.3, F57F4.4, F57F5.1, F57G8.7, F57H12.3, F57H12.6, F58A4.1, F58A4.5, F58A6.4, F58B3.1, F58B3.2, F58B3.3, F58B3.8, F58B4.1, F58B4.3, F58B4.4, F58B4.6, F58B4.7, F58B6.1, F58D5.3, F58D5.6, F58E1.4, F58E1.7, F58E10.7, F58E2.2, F58E2.5, F58E6.13, F58E6.4, F58E6.7, F58F6.1, F58F6.2, F58F9.10, F58F9.11, F58F9.6, F58F9.8, F58F9.9, F58G1.4, F58G1.5, F58G1.8, F58G4.3, F58H1.1, F58H1.2, F58H1.4, F58H1.7, F58H10.1, F58H7.1, F58H7.2, F59A1.16, F59A1.6, F59A6.10, F59A6.11, F59A6.12, F59A6.3, F59A7.1, F59A7.11, F59A7.2, F59A7.5, F59B1.2, F59B10.2, F59B10.5, F59B2.12, F59C6.16, F59C6.8, F59D12.3, F59D12.4, F59D6.1, F59D6.2, F59D6.3, F59E11.5, F59E11.6, F59E11.7, F59E12.13, F59F5.4, F59F5.7, H01G02.1, H01M10.2, H01M10.3, H02F09.3, H02I12.1, H02I12.4, H02I12.8, H02K04.1, H02K04.2, H03A11.1, H03E18.1, H05L03.3, H06A10.1, H06A10.2, H06I04.6, H06I04.7, H06O01.1, H10D18.2, H10D18.4, H10E21.4, H12D21.1, H12D21.10, H12D21.12, H12D21.13, H12D21.14, H12D21.15, H12D21.2, H12D21.3, H12D21.4, H12D21.6, H12I13.6, H13N06.2, H14A12.6, H14A12.7, H14N18.2, H14N18.3, H16D19.1, H17B01.4, H17B01.5, H19M22.3, H20E11.2, H20E11.3, H22K11.1, H22K11.2, H22K11.3, H23N18.4, H23N18.5, H25K10.1, H25K10.5, H27A22.1, H27M09.4, H29C22.1, H31B20.2, H31G24.1, H32K16.2, H32K21.1, H34I24.1, H34I24.2, H34I24.3, H35N09.1, H36L18.1, H37A05.4, H38K22.3, H38K22.5, H38K22.6, H38K22.7, H39E23.2, H39E23.3, H41C03.2, H42K12.3, H43E16.1, H43I07.3, JC8.14, JC8.8, K01A12.4, K01A2.11, K01A6.4, K01A6.5, K01A6.7, K01A6.8, K01C8.2, K01C8.8, K01D12.10, K01D12.11, K01D12.12, K01D12.13, K01D12.14, K01D12.5, K01D12.8, K01D12.9, K01G5.6, K02A11.3, K02A11.4, K02A2.5, K02A6.1, K02A6.4, K02B12.6, K02B12.9, K02B7.3, K02B7.4, K02C4.2, K02D3.1, K02D7.4, K02D7.6, K02E10.6, K02E11.10, K02E11.3, K02E11.4, K02E11.5, K02E11.6, K02E11.7, K02E2.2, K02E2.4, K02E7.1, K02F2.5, K02F3.3, K02F3.5, K02F3.9, K02F6.9, K02G10.4, K02H11.4, K03A11.6, K03B4.4, K03B4.7, K03B8.11, K03B8.14, K03B8.6, K03B8.7, K03D3.2, K03D3.5, K03H1.3, K03H1.4, K03H1.6, K03H6.2, K03H6.7, K03H9.2, K04A8.1, K04A8.5, K04A8.8, K04C2.5, K04E7.3, K04F1.10, K04F1.11, K04F1.13, K04F1.14, K04F1.15, K04F1.7, K04F1.8, K04F1.9, K04G7.4, K04H4.1, K04H4.2, K04H4.6, K04H4.7, K04H8.3, K05D4.4, K05F6.11, K05F6.12, K05F6.4, K05F6.6, K06A4.1, K06A4.7, K06A5.6, K06A9.1, K06B9.1, K06G5.1, K06G5.2, K06H6.1, K06H6.2, K06H6.5, K06H6.6, K07A1.4, K07A1.5, K07A1.6, K07A12.6, K07C11.3, K07C11.4, K07C11.5, K07C11.7, K07C11.8, K07C5.7, K07C5.9, K07C6.2, K07C6.3, K07C6.4, K07C6.5, K07D4.6, K07D4.8, K07D4.9, K07E12.1, K07E3.8, K07E8.1, K07G6.1, K07H8.11, K07H8.7, K08B12.1, K08B4.2, K08B4.6, K08B4.7, K08C7.3, K08C9.1, K08C9.10, K08C9.2, K08C9.4, K08C9.5, K08C9.6, K08D12.4, K08D12.5, K08D12.7, K08D8.3, K08D8.4, K08D8.5, K08D8.6, K08D9.2, K08E3.1, K08E3.2, K08F4.5, K08F8.3, K08H10.4, K08H2.10, K08H2.9, K09A11.2, K09A11.3, K09A11.4, K09A9.8, K09B11.10, K09B3.1, K09C4.8, K09C6.3, K09C6.9, K09C8.4, K09C8.5, K09C8.6, K09C8.7, K09C8.9, K09D9.2, K09D9.9, K09E2.1, K09E4.4, K09E4.6, K09F5.2, K09F5.3, K09H11.4, K09H11.9, K09H9.3, K09H9.5, K10B2.2, K10B2.3, K10B3.6, K10B4.1, K10B4.6, K10C2.1, K10C2.5, K10C2.8, K10C3.4, K10C9.1, K10C9.3, K10C9.9, K10D11.1, K10D11.2, K10D11.3, K10D11.6, K10D11.7, K10D2.6, K10D3.4, K10D3.6, K10G6.2, K10G9.3, K10H10.10, K10H10.4, K11D12.1, K11D12.13, K11D12.7, K11D2.2, K11E4.1, K11E4.3, K11G12.1, K11H12.10, K11H12.11, K11H12.4, K11H12.6, K11H12.7, K11H3.5, K12B6.3, K12B6.9, K12D12.3, K12D9.12, K12D9.14, K12G11.6, K12H4.4, K12H4.7, K12H6.10, K12H6.3, K12H6.9, LLC1.2, M01B2.1, M01B2.8, M01D7.1, M01D7.5, M01E11.4, M01F1.1, M01G12.10, M01H9.2, M02D8.6, M02E1.2, M02F4.7, M02G9.1, M02G9.2, M02G9.3, M02H5.8, M03A1.7, M03B6.3, M03D4.3, M03E7.4, M03F4.6, M03F4.7, M03F8.4, M03F8.5, M03F8.6, M04B2.4, M04B2.6, M04D8.1, M04D8.2, M04D8.3, M04G12.2, M05B5.4, M05B5.7, M05D6.4, M05D6.8, M05D6.9, M110.1, M110.7, M110.8, M110.9, M116.2, M153.3, M153.5, M162.2, M163.8, M176.10, M176.5, M18.1, M199.3, M199.4, M199.6, M199.7, M199.9, M28.6, M6.4, M60.2, M7.10, M7.9, M70.2, M70.4, M79.4, PAR2.4, R01B10.1, R01E6.2, R01E6.7, R01H10.4, R01H2.2, R02D3.6, R02D5.10, R02D5.7, R02D5.9, R02F11.1, R02F2.4, R03A10.2, R03C1.1, R03G8.1, R03G8.3, R03G8.4, R03G8.6, R03H10.4, R03H10.5, R04B3.2, R04B3.3, R04D3.2, R04D3.3, R05A10.1, R05A10.2, R05A10.3, R05A10.4, R05A10.5, R05A10.6, R05A10.7, R05A10.8, R05D3.9, R05D7.7, R05D8.11, R05F9.12, R05G6.9, R05G9R.1, R05H5.7, R06A4.6, R06B10.3, R06C7.4, R06C7.7, R06F6.11, R06F6.7, R07B1.11, R07B1.13, R07B1.5, R07B1.6, R07B1.8, R07B7.11, R07C3.1, R07C3.3, R07E3.6, R07E3.7, R07E4.4, R07E5.4, R07G3.2, R07G3.5, R07G3.9, R08A2.1, R08B4.1, R08B4.5, R08C7.1, R08C7.4, R08C7.6, R08E3.2, R08F11.3, R08F11.7, R08H2.10, R09A1.5, R09B5.12, R09B5.5, R09D1.13, R09D1.8, R09E10.13, R09E10.8, R09F10.2, R09F10.7, R09F10.8, R09F10.9, R09H10.1, R09H10.2, R09H10.3, R09H10.5, R102.11, R10D12.15, R10E11.4, R10H1.4, R10H1.5, R10H10.3, R11.2, R11.3, R11A5.6, R11A5.7, R11A8.3, R11D1.12, R11D1.13, R11D1.3, R11D1.4, R11G10.4, R11G11.14, R11G11.6, R11G11.7, R12A1.2, R12A1.3, R12A1.4, R12C12.10, R12C12.8, R12E2.13, R12E2.14, R12E2.15, R12E2.6, R12E2.7, R12E2.8, R12H7.2, R13A1.1, R13A1.5, R13A5.3, R13A5.6, R13D11.10, R13D7.2, R13F6.2, R13F6.8, R13H4.3, R13H4.8, R148.3, R151.5, R155.5, R160.5, R17.3, R173.4, R186.3, R52.4, R52.5, R52.6, R53.5, R53.8, R57.2, R90.2, T01B10.1, T01B10.2, T01B11.2, T01B6.4, T01B7.7, T01B7.8, T01B7.9, T01C3.4, T01D1.3, T01D1.6, T01D3.1, T01D3.3, T01D3.6, T01E8.8, T02B11.3, T02B11.4, T02B11.7, T02B11.8, T02B11.9, T02C12.5, T02D1.7, T02D1.9, T02E1.8, T02E9.2, T02E9.5, T02E9.6, T02G6.7, T02H6.10, T02H6.3, T02H6.4, T02H6.6, T02H6.7, T02H6.8, T02H6.9, T03D8.2, T03D8.3, T03D8.4, T03D8.7, T03E6.7, T03F1.10, T03F6.10, T03G11.10, T03G11.8, T03G6.3, T04A6.3, T04B2.8, T04B8.1, T04B8.2, T04C12.3, T04F3.2, T04F3.4, T04F3.5, T04F8.8, T04G9.7, T04H1.3, T04H1.4, T04H1.6, T05A10.3, T05A10.5, T05A10.6, T05A6.4, T05A7.10, T05A7.2, T05A7.5, T05A7.7, T05A8.2, T05A8.4, T05A8.6, T05B11.4, T05B4.10, T05B4.11, T05B4.9, T05C1.1, T05C1.5, T05C12.10, T05C3.4, T05C3.6, T05D4.4, T05E11.3, T05E11.6, T05E11.8, T05E12.3, T05E12.6, T05E12.8, T05E8.2, T05F1.10, T05G5.1, T05G5.4, T05G5.7, T05H10.3, T05H10.6, T06A1.1, T06A1.5, T06A1.7, T06A4.1, T06A4.3, T06C10.4, T06C12.14, T06D4.3, T06D8.1, T06D8.10, T06E4.10, T06E4.12, T06E4.14, T06E4.4, T06E4.6, T06E4.8, T06E6.10, T06G6.12, T06G6.6, T07A5.1, T07A5.5, T07A9.15, T07C12.10, T07C12.13, T07C12.15, T07C12.7, T07C4.4, T07C4.5, T07D10.1, T07D10.4, T07D10.6, T07D3.2, T07D3.4, T07E3.2, T07F10.6, T07G12.3, T07H3.4, T07H3.5, T07H3.7, T07H6.3, T08A9.11, T08A9.12, T08A9.2, T08A9.7, T08A9.8, T08A9.9, T08B2.12, T08B2.2, T08B6.9, T08D2.1, T08G2.2, T08G3.11, T08G3.6, T08G5.12, T08G5.15, T08G5.3, T08H10.3, T09A12.2, T09A5.14, T09B9.1, T09B9.3, T09D3.3, T09D3.8, T09E11.1, T09E11.10, T09E11.3, T09E11.8, T09E11.9, T09F5.1, T09F5.9, T10B10.1, T10B10.6, T10B5.7, T10B9.2, T10B9.7, T10B9.8, T10B9.9, T10D4.1, T10D4.13, T10D4.15, T10D4.4, T10E10.5, T10E9.3, T10G3.1, T10H4.10, T10H4.11, T10H4.12, T11B7.3, T11F1.2, T11F1.6, T11F1.8, T11F1.9, T11F8.5, T11F9.13, T11F9.22, T11F9.3, T11F9.5, T11F9.6, T11F9.9, T12A2.16, T12A7.2, T12A7.3, T12A7.4, T12A7.5, T12B5.10, T12B5.15, T12D8.5, T12D8.9, T12E12.6, T12G3.8, T13A10.1, T13A10.5, T13B5.3, T13B5.5, T13B5.6, T13B5.7, T13C2.2, T13C2.3, T13C5.3, T13C5.7, T13F2.4, T13F3.6, T13F3.8, T13H5.3, T14A8.2, T14B4.7, T14B4.9, T14F9.3, T14G10.3, T14G10.4, T14G8.3, T15B7.1, T15B7.7, T15D6.1, T15D6.10, T15D6.12, T15D6.3, T15D6.8, T15D6.9, T15H9.7, T16A1.3, T16A1.4, T16A9.4, T16D1.2, T16G1.1, T16G1.2, T16G1.8, T16G12.1, T16H12.9, T17A3.12, T17H7.1, T17H7.7, T18H9.1, T19A5.1, T19A5.3, T19B10.1, T19B10.12, T19B10.2, T19B10.3, T19B10.9, T19B4.1, T19B4.4, T19C3.2, T19C3.5, T19C3.9, T19C4.7, T19C9.5, T19D12.1, T19D12.6, T19D7.3, T19H12.12, T19H12.3, T19H5.7, T20B12.5, T20B3.14, T20B3.16, T20B3.7, T20B6.3, T20D3.1, T20D3.2, T20D4.10, T20D4.11, T20D4.12, T20D4.16, T20D4.17, T20D4.19, T20D4.20, T20D4.8, T20F10.2, T20F7.3, T20G5.12, T20G5.13, T20G5.7, T20G5.8, T21B4.3, T21B6.2, T21B6.3, T21C12.4, T21C9.6, T21C9.8, T21C9.9, T21D11.1, T21D12.12, T21D12.14, T21D12.2, T21D9.1, T21E3.2, T21G5.6, T21H3.1, T22A3.3, T22A3.8, T22B11.1, T22C1.12, T22C8.2, T22C8.6, T22D1.2, T22E5.1, T22E5.3, T22F7.3, T22F7.4, T22G5.1, T22G5.7, T22H6.1, T22H6.5, T22H6.7, T23B12.11, T23B12.8, T23B3.5, T23B7.3, T23D5.3, T23E1.1, T23E7.4, T23F1.6, T23F11.6, T23F2.4, T23F4.1, T23F4.3, T23F4.4, T23F6.1, T23F6.2, T23F6.5, T23G4.5, T23G7.5, T23H2.4, T23H4.3, T24A11.3, T24A6.15, T24A6.16, T24A6.18, T24A6.19, T24B8.5, T24D1.1, T24D8.3, T24D8.4, T24D8.5, T24D8.6, T24F1.5, T24F1.6, T24H10.4, T24H7.2, T25B2.3, T25B6.2, T25B6.3, T25B9.11, T25B9.3, T25C12.2, T25C12.3, T25C12.4, T25D10.1, T25D10.3, T25D3.3, T25E12.14, T25E4.1, T25F10.2, T25G12.11, T25G3.4, T26A8.3, T26C11.9, T26C12.1, T26C12.6, T26C5.2, T26C5.4, T26C5.5, T26E3.10, T26E3.6, T26E3.8, T26E4.1, T26E4.10, T26E4.2, T26E4.3, T26E4.5, T26E4.9, T26F2.1, T26H5.8, T27A1.3, T27A8.1, T27C5.12, T27C5.7, T27D12.3, T27E4.4, T27E4.5, T27E7.1, T27F6.1, T27F6.2, T28A11.16, T28A11.17, T28A11.18, T28A11.19, T28A11.2, T28A11.3, T28A11.4, T28A11.5, T28A8.2, T28B4.3, T28B8.2, T28C6.1, T28C6.4, T28C6.6, T28D6.3, T28D6.5, T28D9.12, T28F2.1, T28H10.2, T28H10.3, T28H10.4, VB0395L.1, VC5.2, VC5.3, VK10D6R.1, VZK822L.2, W01A11.1, W01A8.6, W01A8.7, W01A8.8, W01B11.5, W01B6.1, W01C8.5, W01F3.1, W01F3.2, W01F3.3, W02A2.2, W02A2.3, W02B12.1, W02B12.11, W02B12.13, W02B12.4, W02B3.4, W02B3.7, W02B8.5, W02D3.3, W02D7.10, W02D7.12, W02D7.2, W02D9.5, W02D9.7, W03A5.3, W03C9.1, W03D2.1, W03D2.5, W03D2.9, W03D8.1, W03D8.11, W03F11.1, W03F11.5, W03F8.5, W03F9.11, W03G1.7, W03G11.3, W04A4.2, W04A4.3, W04C9.2, W04E12.6, W04E12.8, W04G3.10, W04G3.12, W04G3.13, W04G3.2, W04G3.3, W04G3.8, W04G5.10, W05B10.3, W05B2.2, W05B2.5, W05B2.6, W05E10.4, W05E7.3, W05F2.7, W05G11.3, W06A11.1, W06A7.5, W07B8.1, W07B8.4, W07E11.2, W07E11.3, W07E11.4, W07G1.7, W07G4.1, W07G4.2, W07G4.7, W07G9.2, W08A12.2, W08A12.3, W08D2.1, W08D2.6, W08E12.2, W08E12.3, W08E12.4, W08E12.5, W08E12.6, W08F4.3, W08F4.5, W08F4.7, W08G11.1, W09B12.1, W09C2.10, W09C2.7, W09C2.8, W09C2.9, W09C3.2, W09C3.7, W09C3.8, W09C5.11, W09C5.4, W09D10.4, W09D6.4, W09G10.5, W09G12.1, W09G12.4, W09G12.9, W09G3.3, W10D9.2, W10G11.1, W10G11.11, W10G11.12, W10G11.13, W10G11.14, W10G11.15, W10G11.19, W10G11.2, W10G11.3, W10G11.4, Y102A11A.5, Y102A5B.2, Y102A5B.3, Y102A5C.27, Y102A5C.4, Y102A5C.40, Y102E9.3, Y102E9.5, Y102E9.6, Y105C5A.17, Y105C5A.3, Y105C5A.4, Y105C5A.5, Y105C5A.6, Y105C5A.8, Y105C5B.15, Y105C5B.18, Y105C5B.25, Y105C5B.3, Y105E8A.2, Y105E8B.8, Y105E8B.9, Y106G6D.6, Y106G6D.8, Y106G6H.1, Y106G6H.5, Y110A2AL.14, Y110A2AL.3, Y110A2AL.4, Y110A2AL.5, Y110A2AL.6, Y110A2AL.7, Y110A2AL.9, Y110A7A.7, Y111B2A.15, Y111B2A.2, Y113G7A.15, Y113G7B.15, Y116A8A.2, Y116A8A.3, Y116A8A.4, Y116A8A.6, Y116A8A.7, Y116A8B.1, Y116A8C.1, Y116A8C.21, Y116A8C.26, Y116A8C.29, Y116A8C.3, Y116A8C.33, Y116A8C.4, Y116A8C.43, Y116A8C.44, Y116A8C.5, Y116F11A.3, Y116F11B.1, Y116F11B.12, Y116F11B.13, Y116F11B.2, Y116F11B.3, Y116F11B.8, Y116F11B.9, Y119C1B.6, Y119C1B.9, Y119D3B.13, Y119D3B.16, Y11D7A.5, Y11D7A.8, Y12A6A.1, Y12A6A.2, Y16B4A.2, Y17D7B.5, Y17D7B.6, Y17D7B.8, Y17D7C.2, Y17G9A.2, Y17G9A.3, Y17G9B.3, Y18D10A.10, Y18D10A.2, Y18D10A.24, Y18H1A.10, Y18H1A.13, Y18H1A.8, Y18H1A.9, Y19D10A.6, Y19D10A.7, Y19D10A.9, Y19D10B.3, Y19D10B.4, Y19D10B.6, Y1A5A.2, Y20C6A.4, Y22D7AL.13, Y22D7AL.14, Y22D7AL.6, Y22D7AR.10, Y22F5A.4, Y22F5A.5, Y23B4A.2, Y23H5B.1, Y23H5B.3, Y23H5B.8, Y25C1A.1, Y25C1A.2, Y25C1A.3, Y25C1A.4, Y26D4A.12, Y26D4A.2, Y26D4A.6, Y26G10.5, Y26G10.6, Y26G10.7, Y27F2A.6, Y2H9A.3, Y32F6A.5, Y32G9A.12, Y32G9A.2, Y32G9A.5, Y34B4A.10, Y34B4A.3, Y34B4A.5, Y34B4A.6, Y34B4A.9, Y34D9A.11, Y34F4.3, Y37A1B.7, Y37A1B.8, Y37A1B.9, Y37B11A.1, Y37D8A.15, Y37D8A.19, Y37D8A.2, Y37D8A.3, Y37E11AL.4, Y37E11AL.6, Y37E11AR.3, Y37E11AR.6, Y37E11AR.7, Y37E11B.7, Y37E3.13, Y37E3.19, Y37F4.3, Y37H2A.10, Y37H2A.11, Y37H2A.13, Y38C1AA.3, Y38C1AA.9, Y38C1AB.1, Y38C1AB.5, Y38C1BA.3, Y38C9B.1, Y38E10A.1, Y38E10A.10, Y38E10A.11, Y38E10A.12, Y38E10A.13, Y38E10A.15, Y38E10A.16, Y38E10A.25, Y38E10A.26, Y38E10A.29, Y38E10A.4, Y38E10A.5, Y38E10A.7, Y38E10A.9, Y38H6A.1, Y38H6A.2, Y38H6A.3, Y38H6A.5, Y38H6C.15, Y38H6C.19, Y38H6C.22, Y38H6C.23, Y38H6C.8, Y38H8A.1, Y39A1A.21, Y39A1A.24, Y39A1A.7, Y39A1B.1, Y39A1C.1, Y39A1C.4, Y39A3A.2, Y39A3A.5, Y39A3B.1, Y39A3CL.6, Y39B6A.48, Y39B6A.5, Y39B6A.9, Y39E4B.12, Y39E4B.13, Y39E4B.8, Y39F10A.1, Y39F10A.3, Y39F10B.1, Y39F10C.1, Y39F10C.2, Y39G10AL.1, Y39G8B.10, Y39G8B.7, Y39G8B.9, Y39G8C.4, Y39H10A.1, Y40B10B.1, Y40C5A.3, Y40H7A.10, Y40H7A.3, Y40H7A.4, Y41C4A.1, Y41C4A.16, Y41C4A.22, Y41C4A.6, Y41C4A.8, Y41D4A.1, Y41D4A.7, Y41D4B.10, Y41D4B.15, Y41D4B.16, Y41D4B.26, Y41D4B.6, Y41E3.1, Y41E3.22, Y41G9A.10, Y41G9A.5, Y42A5A.2, Y42A5A.3, Y42G9A.2, Y42H9AR.5, Y42H9B.1, Y43B11AR.1, Y43C5A.2, Y43C5A.4, Y43F4A.1, Y43F8A.1, Y43F8A.2, Y43F8B.11, Y43F8B.18, Y43F8B.20, Y43F8B.3, Y43F8B.5, Y43F8C.1, Y43F8C.15, Y43F8C.16, Y43F8C.17, Y43F8C.2, Y43F8C.20, Y43F8C.23, Y43F8C.5, Y43F8C.7, Y43F8C.9, Y44A6B.3, Y44A6B.4, Y44E3A.2, Y44E3B.2, Y45F10A.5, Y45F10C.2, Y45F10C.4, Y45F10D.11, Y45F10D.14, Y45F10D.6, Y45F3A.4, Y45G12C.11, Y45G5AM.6, Y46C8AL.1, Y46C8AL.2, Y46C8AL.3, Y46C8AL.4, Y46C8AL.5, Y46C8AL.8, Y46C8AL.9, Y46C8AR.1, Y46C8AR.3, Y46D2A.2, Y46E12A.1, Y46E12A.5, Y46G5A.14, Y46G5A.23, Y46G5A.29, Y46G5A.34, Y46G5A.39, Y46G5A.7, Y46H3A.7, Y46H3B.1, Y46H3B.2, Y46H3C.7, Y46H3D.1, Y46H3D.8, Y47A7.2, Y47D3A.23, Y47D3B.10, Y47D3B.12, Y47D3B.2, Y47D3B.3, Y47D3B.4, Y47D3B.6, Y47D7A.10, Y47D7A.11, Y47D7A.12, Y47D7A.2, Y47D7A.5, Y47D7A.6, Y47D7A.7, Y47D7A.9, Y47G6A.15, Y47G6A.29, Y47G6A.33, Y47H10A.2, Y47H9B.2, Y47H9C.1, Y47H9C.5, Y48A6A.1, Y48A6B.4, Y48B6A.7, Y48B6A.8, Y48B6A.9, Y48D7A.1, Y48D7A.2, Y48E1B.16, Y48E1B.8, Y48E1B.9, Y48G10A.6, Y48G1BL.7, Y48G1BR.1, Y48G1C.13, Y48G8AL.12, Y49A3A.4, Y49C4A.9, Y49E10.10, Y49E10.16, Y49E10.18, Y49E10.25, Y49E10.4, Y49F6B.12, Y49F6B.13, Y49F6B.6, Y49F6C.5, Y49F6C.6, Y49G5A.1, Y49G5B.1, Y4C6A.4, Y4C6B.6, Y4C6B.7, Y50D4B.6, Y50D4C.4, Y50D7A.13, Y50D7A.5, Y50E8A.1, Y50E8A.10, Y50E8A.4, Y50E8A.5, Y50E8A.6, Y50E8A.7, Y51A2A.11, Y51A2A.12, Y51A2A.4, Y51A2A.6, Y51A2A.7, Y51A2B.4, Y51A2B.6, Y51A2B.9, Y51A2D.1, Y51A2D.10, Y51A2D.11, Y51A2D.14, Y51A2D.8, Y51A2D.9, Y51B9A.8, Y51F10.5, Y51F10.7, Y51H4A.1, Y51H4A.10, Y51H4A.12, Y51H4A.22, Y51H4A.32, Y51H4A.5, Y51H4A.8, Y51H4A.935, Y51H7C.1, Y51H7C.13, Y51H7C.15, Y51H7C.2, Y52B11A.5, Y52B11A.8, Y52B11B.1, Y53C10A.10, Y53C10A.15, Y53C12B.6, Y53C12B.7, Y53F4B.14, Y53F4B.18, Y53F4B.27, Y53F4B.39, Y53G8AM.5, Y53G8AM.6, Y53G8AR.1, Y53H1A.4, Y53H1B.2, Y54E10A.17, Y54E10BL.2, Y54E10BL.4, Y54E2A.9, Y54F10AM.2, Y54F10AM.6, Y54F10AM.8, Y54F10BM.12, Y54F10BM.3, Y54G11A.1, Y54G11A.8, Y54G2A.10, Y54G2A.14, Y54G2A.15, Y54G2A.23, Y54G2A.28, Y54G2A.29, Y54G2A.32, Y54G2A.33, Y54G2A.39, Y54G2A.44, Y54G2A.45, Y54G2A.57, Y54G2A.6, Y54G2A.8, Y54G2A.9, Y55B1BL.1, Y55D5A.3, Y55F3AM.11, Y55F3BR.11, Y55F3BR.2, Y55F3BR.8, Y56A3A.12, Y56A3A.14, Y56A3A.19, Y56A3A.21, Y57A10A.11, Y57A10A.23, Y57A10A.24, Y57A10A.29, Y57A10B.3, Y57E12AR.1, Y57E12B.3, Y57G11A.2, Y57G11A.4, Y57G11B.2, Y57G11B.3, Y57G11B.5, Y57G11C.1130, Y57G11C.18, Y57G11C.38, Y57G11C.39, Y57G11C.40, Y57G11C.41, Y57G11C.42, Y57G11C.52, Y57G11C.8, Y57G7A.1, Y57G7A.3, Y58G8A.5, Y59A8B.19, Y59A8B.20, Y59A8B.26, Y59C2A.1, Y59E9AL.1, Y59E9AL.6, Y59E9AR.10, Y59E9AR.4, Y59E9AR.9, Y59H11AM.4, Y59H11AR.5, Y5F2A.1, Y5F2A.2, Y5H2A.1, Y5H2A.3, Y5H2B.5, Y5H2B.6, Y60A3A.21, Y60A3A.23, Y60A3A.7, Y60A3A.9, Y62E10A.13, Y62E10A.19, Y62E10A.3, Y62F5A.9, Y62H9A.3, Y62H9A.4, Y62H9A.5, Y62H9A.6, Y64G10A.1, Y64G10A.2, Y64G10A.7, Y64H9A.2, Y65A5A.2, Y65B4A.2, Y65B4BL.6, Y65B4BR.1, Y65B4BR.2, Y65B4BR.6, Y66A7A.6, Y66A7A.9, Y66H1A.5, Y67A10A.10, Y67A10A.11, Y67A10A.2, Y67D2.2, Y67D8B.5, Y67D8C.7, Y67D8C.8, Y68A4B.1, Y69A2AL.1, Y69A2AR.22, Y69A2AR.5, Y69F12A.3, Y69H2.10, Y69H2.3, Y6E2A.8, Y6G8.14, Y6G8.15, Y6G8.16, Y70C5C.2, Y70C5C.3, Y70C5C.5, Y70D2A.2, Y71A12B.6, Y71F9AM.8, Y71F9B.15, Y71F9B.9, Y71G12B.18, Y71G12B.20, Y71G12B.21, Y71G12B.4, Y71G12B.5, Y71H2B.4, Y73B3B.3, Y73B6A.3, Y73B6BL.1, Y73B6BL.21, Y73B6BL.24, Y73B6BL.25, Y73B6BL.34, Y73B6BL.35, Y73B6BL.37, Y73B6BL.44, Y73C8C.12, Y73C8C.2, Y73C8C.4, Y73E7A.7, Y73E7A.8, Y73F4A.1, Y73F4A.2, Y73F4A.3, Y73F8A.10, Y73F8A.18, Y73F8A.22, Y73F8A.23, Y73F8A.26, Y73F8A.35, Y73F8A.6, Y73F8A.8, Y73F8A.9, Y74C10AR.2, Y74C9A.2, Y75B12B.11, Y75B12B.13, Y75B12B.8, Y75B7AR.1, Y75B8A.11, Y75B8A.20, Y75B8A.3, Y75B8A.44, Y75B8A.9, Y76A2B.2, Y76B12C.3, Y76B12C.8, Y77E11A.15, Y7A5A.9, Y80D3A.10, Y81G3A.4, Y82E9BR.4, Y87G2A.15, Y87G2A.16, Y8A9A.2, Y8A9A.6, Y8G1A.1, Y94A7B.11, Y94H6A.4, Y95B8A.2, Y97E10AL.2, Y97E10B.1, ZC101.2, ZC116.3, ZC13.4, ZC15.2, ZC15.5, ZC15.8, ZC155.5, ZC168.2, ZC168.5, ZC204.1, ZC204.17, ZC204.6, ZC21.3, ZC21.8, ZC239.22, ZC250.1, ZC328.5, ZC334.1, ZC334.10, ZC334.11, ZC334.13, ZC334.2, ZC334.3, ZC334.7, ZC334.8, ZC334.9, ZC373.2, ZC373.6, ZC373.7, ZC374.2, ZC376.8, ZC410.3, ZC412.10, ZC412.3, ZC412.6, ZC412.7, ZC412.8, ZC412.9, ZC434.3, ZC434.9, ZC449.1, ZC449.2, ZC455.10, ZC482.2, ZC482.4, ZC487.4, ZC487.5, ZC504.1, ZC506.1, ZC513.8, ZC64.2, ZC84.1, ZC84.6, ZK1010.4, ZK1025.2, ZK1025.7, ZK1025.8, ZK1037.1, ZK1037.10, ZK105.1, ZK105.3, ZK105.6, ZK1055.5, ZK1058.4, ZK1067.7, ZK1098.9, ZK112.1, ZK112.3, ZK1193.1, ZK1225.1, ZK1225.4, ZK1225.5, ZK1236.1, ZK1248.1, ZK1248.16, ZK1251.11, ZK1251.2, ZK1290.1, ZK1290.10, ZK1290.11, ZK1290.12, ZK1290.7, ZK1290.8, ZK1307.2, ZK1307.5, ZK1307.8, ZK1320.10, ZK1320.2, ZK1320.3, ZK1320.4, ZK1321.1, ZK154.1, ZK154.4, ZK177.2, ZK180.5, ZK180.6, ZK20.6, ZK218.1, ZK218.11, ZK218.3, ZK218.5, ZK218.7, ZK250.2, ZK262.2, ZK262.3, ZK265.2, ZK265.7, ZK265.8, ZK285.2, ZK287.3, ZK287.4, ZK287.8, ZK337.1, ZK337.5, ZK353.10, ZK353.5, ZK354.3, ZK355.1, ZK355.4, ZK355.5, ZK377.1, ZK381.2, ZK384.1, ZK384.2, ZK39.3, ZK39.4, ZK39.6, ZK39.7, ZK39.8, ZK39.9, ZK402.2, ZK430.8, ZK455.4, ZK455.5, ZK470.6, ZK488.10, ZK488.7, ZK507.4, ZK512.10, ZK512.7, ZK525.1, ZK546.15, ZK546.5, ZK550.5, ZK593.2, ZK596.1, ZK6.10, ZK6.11, ZK6.3, ZK6.7, ZK616.9, ZK617.2, ZK622.5, ZK643.6, ZK643.8, ZK662.2, ZK662.6, ZK666.11, ZK666.3, ZK666.5, ZK666.6, ZK666.7, ZK669.1, ZK669.3, ZK673.1, ZK673.9, ZK675.4, ZK678.5, ZK682.4, ZK688.1, ZK688.4, ZK688.8, ZK697.8, ZK75.1, ZK75.3, ZK813.1, ZK813.2, ZK813.7, ZK816.1, ZK816.4, ZK822.2, ZK822.4, ZK829.3, ZK836.1, ZK84.1, ZK84.3, ZK84.6, ZK849.6, ZK856.4, ZK856.5, ZK856.6, ZK856.7, ZK858.3, ZK863.2, ZK863.9, ZK867.3, ZK896.1, ZK896.4, ZK896.5, ZK896.6, ZK896.7, ZK897.1, ZK909.6, ZK970.7, ZK973.11, ZK973.4, ZK994.1, ZK994.3

**all putative secreted proteins (3484)**

4R79.1, AC3.3, AC3.4, AC3.6, AH6.3, B0001.4, B0019.1, B0024.1, B0024.2, B0034.1, B0034.4, B0035.13, B0047.4, B0205.10, B0205.12, B0205.13, B0207.2, B0213.10, B0213.11, B0213.12, B0213.14, B0213.15, B0213.16, B0213.17, B0213.3, B0213.4, B0213.5, B0213.6, B0218.6, B0218.8, B0222.11, B0222.5, B0222.7, B0222.8, B0228.1, B0228.8, B0238.12, B0238.15, B0238.7, B0250.2, B0252.2, B0252.8, B0261.5, B0280.5, B0280.7, B0285.7, B0294.1, B0310.6, B0331.1, B0334.1, B0334.13, B0344.2, B0361.9, B0365.5, B0365.6, B0379.2, B0379.7, B0393.7, B0393.9, B0403.3, B0403.4, B0403.5, B0410.3, B0412.2, B0416.2, B0416.7, B0432.11, B0432.12, B0432.14, B0454.8, B0457.2, B0464.2, B0478.3, B0491.2, B0496.11, B0507.1, B0507.5, B0511.5, B0513.4, B0524.5, B0545.3, B0554.1, B0554.6, B0563.10, B0563.9, BE0003N10.3, BE10.4, C01A2.7, C01A2.9, C01B10.6, C01B12.1, C01B7.7, C01C4.1, C01F1.5, C01G10.15, C01G10.16, C01G10.17, C01G10.18, C01G10.4, C01G10.5, C01G10.6, C01G12.10, C01G12.11, C01G12.13, C01G12.2, C01G12.6, C01G12.9, C01G5.9, C01G6.3, C01G6.9, C01H6.1, C01H6.8, C02A12.4, C02B10.3, C02B4.1, C02B4.4, C02C2.1, C02E7.6, C02E7.7, C02F12.3, C02F12.5, C02F4.4, C02F5.14, C03A7.12, C03A7.13, C03A7.14, C03A7.4, C03A7.7, C03A7.8, C03B1.6, C03C11.1, C03E10.5, C03E10.6, C03G5.10, C03G5.11, C03G5.12, C03G5.13, C03G5.2, C03G5.8, C03G5.9, C03G6.14, C03G6.15, C03G6.5, C03G6.6, C03H5.1, C04B4.3, C04E12.2, C04E6.13, C04E6.6, C04F1.1, C04F12.5, C04G2.1, C04G6.10, C04G6.5, C04G6.7, C04H5.2, C04H5.7, C04H5.8, C05B5.1, C05B5.11, C05B5.3, C05C10.8, C05C8.3, C05C8.8, C05C9.1, C05D11.6, C05D12.2, C05D12.3, C05D2.8, C05D9.3, C05D9.4, C05D9.9, C05E11.6, C05E11.7, C05E11.8, C05E4.7, C05E7.1, C05E7.2, C05E7.3, C05G5.5, C06A12.5, C06A12.8, C06A6.5, C06A8.3, C06B3.3, C06B8.2, C06C3.10, C06C3.4, C06C3.9, C06C6.6, C06C6.8, C06C6.9, C06E1.5, C06E1.6, C06E2.8, C06E2.9, C06E4.2, C06E7.2, C06E7.6, C06E7.88, C06E8.5, C06G1.1, C06G1.2, C07A12.2, C07A4.1, C07A4.2, C07A9.1, C07B5.3, C07B5.5, C07D10.4, C07E3.10, C07E3.9, C07G1.2, C07G2.1, C07G3.10, C07G3.8, C08A9.10, C08B6.10, C08B6.14, C08B6.3, C08B6.4, C08B6.6, C08E3.14, C08E8.11, C08F11.11, C08F11.12, C08F11.17, C08G5.3, C08H9.1, C08H9.14, C09B7.2, C09C7.1, C09D4.2, C09E7.2, C09E7.3, C09E8.2, C09G1.5, C09G12.17, C09G12.5, C09G5.4, C09G5.5, C09G5.6, C09G5.8, C09G9.3, C09G9.4, C09G9.8, C09H10.5, C09H5.7, C10C5.7, C10F3.1, C10F3.7, C10G11.10, C10G8.3, C10G8.4, C11D2.2, C11H1.5, C11H1.7, C12D12.1, C12D5.10, C12D5.3, C12D5.5, C12D5.9, C12D8.15, C12D8.4, C12D8.8, C13A10.2, C13A2.2, C13A2.3, C13A2.4, C13A2.7, C13C12.2, C13C4.7, C14A4.9, C14A6.1, C14A6.3, C14B1.9, C14B9.6, C14C10.7, C14C11.3, C14C11.8, C14C6.2, C14C6.3, C14C6.5, C14C6.6, C14C6.7, C14E2.4, C14E2.6, C14F11.7, C14F5.2, C15A11.3, C15A11.5, C15A11.6, C15B12.4, C15C7.7, C15C8.3, C15F1.2, C15H11.10, C15H11.13, C15H9.11, C15H9.9, C16A11.8, C16C8.10, C16C8.2, C16C8.8, C16C8.9, C16D9.1, C16D9.5, C16D9.8, C16D9.9, C16E9.1, C17A2.2, C17A2.7, C17B7.12, C17B7.2, C17B7.4, C17B7.9, C17C3.13, C17C3.18, C17C3.19, C17C3.2, C17C3.20, C17C3.4, C17E7.10, C17F3.3, C17F4.12, C17F4.2, C17F4.3, C17F4.7, C17G1.5, C17G1.6, C17G10.5, C17G10.6, C17H12.11, C17H12.6, C17H12.8, C18A11.2, C18A3.11, C18B10.6, C18B2.1, C18B2.2, C18D1.3, C18D11.9, C18D4.4, C18F10.2, C18G1.6, C18G1.7, C18H7.1, C18H7.3, C18H7.7, C18H9.1, C23G10.11, C23H3.9, C23H5.8, C23H5.9, C24A1.1, C24A11.5, C24A3.2, C24B5.5, C24F3.3, C24G6.6, C24G6.7, C24H12.1, C24H12.10, C24H12.11, C24H12.8, C25A1.8, C25A8.2, C25A8.4, C25D7.15, C25D7.5, C25E10.10, C25E10.11, C25E10.13, C25E10.8, C25E10.9, C25F6.6, C25F6.8, C25F9.8, C25G4.1, C25G6.4, C25H3.15, C25H3.5, C26B9.2, C26B9.3, C26B9.5, C26C6.3, C26C6.4, C26D10.6, C26F1.1, C26F1.10, C26F1.2, C26F1.5, C27A2.4, C27A2.5, C27A2.8, C27A7.8, C27A7.9, C27B7.7, C27B7.9, C27C12.3, C27D6.12, C27D9.2, C27F2.9, C28C12.1, C28C12.3, C28C12.4, C28D4.10, C28H8.2, C28H8.7, C28H8.8, C29E4.1, C29E4.14, C29E4.17, C29E6.1, C29F3.2, C29F3.3, C29F3.4, C29F3.5, C29F3.7, C29F4.1, C29F5.8, C29F9.11, C29G2.6, C30E1.4, C30E1.8, C30F2.1, C30F2.3, C30F2.4, C30F8.3, C30G12.1, C30G4.3, C30G4.6, C30G7.4, C30H6.1, C30H6.10, C30H6.4, C30H7.2, C31B8.7, C31B8.9, C31C9.1, C31H1.2, C31H1.5, C31H1.7, C31H2.2, C31H5.7, C32A9.1, C32E12.3, C32E8.12, C32E8.4, C32H11.1, C32H11.10, C32H11.11, C32H11.12, C32H11.3, C32H11.4, C32H11.5, C32H11.6, C32H11.8, C32H11.9, C33A12.15, C33A12.19, C33A12.2, C33B4.5, C33G3.4, C33G3.5, C33G8.12, C33G8.13, C33G8.2, C33G8.3, C33G8.4, C33H5.2, C34B4.4, C34B4.7, C34B7.1, C34B7.3, C34C12.7, C34C6.3, C34C6.8, C34D4.10, C34D4.11, C34D4.9, C34E7.4, C34F11.8, C34F6.1, C34F6.4, C34G6.1, C34G6.2, C34H4.1, C34H4.2, C34H4.3, C34H4.4, C35A11.2, C35A11.3, C35A5.10, C35A5.11, C35A5.6, C35B1.4, C35B1.7, C35B8.1, C35C5.3, C35C5.8, C35D10.14, C35D10.15, C35E7.7, C35E7.9, C36B1.1, C36B1.8, C36B7.5, C36B7.7, C36C5.12, C36C5.14, C36C5.15, C36C5.5, C36F7.5, C36H8.3, C37A5.8, C37C3.13, C37C3.4, C37C3.6, C37C3.8, C37H5.10, C37H5.11, C37H5.4, C38C10.6, C38C3.10, C38C3.6, C38C6.3, C38C6.5, C38C6.6, C38H2.2, C39B10.7, C39B5.14, C39B5.5, C39E6.1, C39E9.2, C39E9.3, C39E9.4, C39E9.5, C39E9.6, C39E9.8, C39E9.9, C40A11.10, C40C9.3, C40H1.7, C40H1.8, C40H1.9, C40H5.1, C40H5.4, C41C4.1, C41C4.3, C41C4.9, C41G11.1, C41G6.1, C41G6.13, C41G6.6, C41G7.6, C41H7.7, C42D4.1, C42D4.13, C42D4.3, C42D8.5, C43F9.11, C43F9.5, C43F9.7, C43F9.8, C43G2.3, C43G2.5, C44B11.4, C44B12.1, C44B12.2, C44B12.3, C44B12.5, C44B12.9, C44B7.5, C44C1.2, C44C1.6, C44C10.7, C44E4.8, C44H4.3, C44H9.5, C45B11.6, C45B2.1, C45B2.2, C45B2.3, C45E5.4, C45G7.2, C45G7.3, C45G9.10, C45G9.12, C45G9.13, C45G9.6, C46A5.3, C46A5.4, C46C2.5, C46C2.6, C46E10.1, C46F2.1, C46F4.3, C46H11.7, C46H11.8, C46H11.9, C47D12.4, C47D2.1, C47E12.10, C47E12.14, C47E12.6, C47E12.9, C47E8.11, C47F8.3, C47F8.4, C47F8.5, C47F8.6, C47F8.7, C48B4.12, C48B4.13, C48D1.5, C48D5.3, C48E7.10, C48E7.7, C49A1.1, C49A1.5, C49A1.6, C49C3.11, C49C3.13, C49C3.20, C49C3.4, C49C3.9, C49C8.6, C49D10.10, C49F8.3, C49G7.3, C49G7.4, C49G7.8, C49G9.2, C49H3.12, C50B6.4, C50B6.7, C50D2.1, C50D2.6, C50D2.7, C50E3.10, C50E3.15, C50E3.2, C50E3.6, C50F2.10, C50F2.4, C50F2.6, C50F2.9, C50F4.3, C50F4.8, C50F7.2, C50F7.5, C50F7.9, C50H11.17, C50H2.10, C50H2.3, C51E3.10, C52D10.11, C52D10.13, C52E2.2, C52E2.3, C52G5.2, C53A5.11, C53A5.2, C53B4.8, C53B7.1, C53B7.2, C53D6.2, C53D6.4, C53D6.8, C54C6.5, C54C8.11, C54C8.12, C54C8.2, C54C8.3, C54C8.4, C54C8.5, C54C8.6, C54C8.7, C54C8.9, C54D1.2, C54D1.7, C54D10.1, C54D10.10, C54D10.13, C54D10.2, C54D10.3, C54D10.9, C54D2.1, C54D2.2, C54D2.4, C54D2.6, C54E4.2, C54F6.12, C54F6.15, C54F6.3, C54F6.5, C54F6.6, C54G6.5, C54G7.3, C55A1.4, C55A1.6, C55A1.7, C55A6.12, C55C3.3, C55C3.5, C55F2.2, C56A3.1, C56A3.2, C56C10.4, C56G2.4, C56G3.2, CC4.2, CD4.11, D1005.3, D1007.13, D1007.14, D1007.18, D1007.19, D1009.4, D1014.5, D1014.6, D1022.2, D1022.8, D1025.4, D1025.6, D1025.7, D1025.8, D1025.9, D1044.7, D1054.10, D1054.11, D1054.9, D1065.3, D1081.10, D1081.12, D1086.1, D1086.10, D1086.11, D1086.12, D1086.17, D1086.18, D1086.2, D1086.3, D1086.5, D1086.6, D1086.7, D1086.8, D2005.2, D2007.1, D2023.7, D2024.4, D2024.8, D2062.13, D2062.6, D2089.3, D2092.8, D2096.10, D2096.13, D2096.5, D2096.6, D2096.9, DY3.5, E01A2.10, E01A2.3, E01A2.7, E01G4.6, E01G6.1, E01H11.3, E02A10.2, E02A10.4, E02C12.13, E02C12.4, E02H4.4, E02H4.7, E02H9.1, E02H9.7, E02H9.9, E03D2.1, E03D2.2, E03G2.3, E03G2.4, E03H12.4, E03H12.5, E03H12.7, E03H12.9, E03H4.10, E03H4.12, E03H4.2, E03H4.3, E03H4.4, E03H4.5, E04A4.6, E04D5.4, E04D5.5, E04F6.6, E04F6.8, E04F6.9, EEED8.11, EGAP1.3, EGAP4.1, EGAP5.1, EGAP7.1, F01D4.8, F01D4.9, F01D5.1, F01D5.2, F01D5.3, F01D5.5, F01D5.6, F01D5.9, F01F1.15, F01G10.6, F01G10.7, F01G12.5, F02A9.2, F02A9.3, F02A9.6, F02C12.4, F02C9.2, F02D10.1, F02D8.2, F02D8.4, F02D8.5, F02E11.5, F02H6.6, F07B7.13, F07C4.10, F07C4.2, F07C4.6, F07C4.7, F07C4.9, F07D3.2, F07F6.5, F07G11.4, F07G6.10, F07G6.2, F08B12.4, F08B4.4, F08B6.3, F08C6.1, F08C6.6, F08D12.2, F08D12.7, F08E10.7, F08F1.6, F08F3.1, F08F3.10, F08F3.7, F08G12.11, F08G12.8, F08G2.6, F08G2.8, F08G5.4, F08G5.6, F08G5.7, F08H9.5, F08H9.6, F08H9.7, F08H9.8, F08H9.9, F09A5.3, F09B12.1, F09B12.5, F09B9.4, F09C6.10, F09C8.1, F09D12.1, F09E10.1, F09E5.14, F09E5.16, F09E5.17, F09E8.1, F09E8.5, F09E8.6, F09E8.8, F09F3.5, F09F3.6, F09F7.8, F09F9.2, F09F9.3, F09G8.2, F09G8.5, F09G8.6, F09G8.8, F10A3.11, F10A3.4, F10C1.3, F10C2.3, F10C2.5, F10D11.6, F10D2.8, F10E7.4, F10E9.12, F10F2.3, F10F2.8, F10F2.9, F10G2.1, F10G2.2, F10G2.3, F10G7.11, F11A5.17, F11A5.7, F11C1.4, F11C7.2, F11C7.3, F11C7.5, F11C7.6, F11C7.7, F11D11.1, F11D11.11, F11D11.14, F11D11.3, F11D11.5, F11D11.6, F11D11.7, F11D11.8, F11E6.2, F11E6.4, F11E6.9, F11F1.2, F11F1.4, F11F1.5, F11F1.6, F11F1.8, F11G11.14, F11H8.3, F12A10.1, F12A10.7, F12A10.9, F12F6.3, F12F6.9, F13A2.1, F13A2.5, F13A7.12, F13B12.4, F13B12.5, F13B6.1, F13B6.3, F13B9.2, F13C5.3, F13D12.3, F13D2.4, F13E9.10, F13E9.12, F13E9.14, F13E9.15, F13E9.2, F13E9.3, F13E9.4, F13E9.8, F13E9.9, F13G11.3, F13G3.12, F13G3.3, F13H10.9, F13H8.3, F13H8.5, F14B4.1, F14B6.1, F14B6.4, F14B6.6, F14B8.2, F14B8.4, F14D2.5, F14D2.7, F14D7.10, F14D7.14, F14D7.5, F14D7.7, F14E5.4, F14E5.5, F14E5.6, F14F11.2, F14F3.4, F14F3.5, F14F7.1, F14F8.8, F14F9.2, F14F9.5, F14H12.1, F14H12.3, F14H3.14, F14H3.5, F14H8.8, F15A2.1, F15A4.2, F15A4.6, F15B9.1, F15B9.11, F15B9.3, F15B9.5, F15B9.9, F15D4.4, F15D4.8, F15E11.11, F15E11.2, F15E11.4, F15E11.5, F15E6.1, F15E6.10, F15E6.3, F15E6.4, F15G9.4, F15G9.5, F15H10.10, F15H10.5, F15H10.6, F15H10.9, F15H9.1, F16B12.2, F16B3.3, F16B4.4, F16B4.7, F16C3.2, F16C3.4, F16F9.2, F16F9.3, F16F9.4, F16G10.10, F16G10.11, F16G10.13, F16G10.14, F16G10.2, F16G10.3, F16G10.4, F16G10.8, F16G10.9, F16H6.1, F16H6.3, F17B5.10, F17B5.3, F17B5.4, F17B5.5, F17B5.9, F17C11.2, F17C11.3, F17C11.5, F17C8.2, F17E9.1, F17E9.11, F17E9.15, F17E9.2, F17E9.3, F17E9.4, F18A1.7, F18A12.1, F18A12.3, F18A12.4, F18A12.6, F18C5.5, F18C5.9, F18E2.1, F18E9.2, F18E9.3, F18E9.4, F18E9.7, F18F11.4, F19B10.6, F19C6.3, F19C6.4, F19C7.1, F19C7.2, F19C7.4, F19C7.7, F19F10.4, F19F10.8, F19G12.7, F19H8.2, F19H8.4, F20A1.1, F20A1.10, F20A1.6, F20A1.8, F20B10.3, F20B6.1, F20B6.4, F20C5.7, F20G2.4, F20H11.5, F21A3.2, F21A3.3, F21A3.8, F21C10.11, F21C10.5, F21C10.6, F21C10.8, F21C3.2, F21D12.2, F21D5.3, F21D9.3, F21E9.3, F21E9.4, F21E9.6, F21F3.1, F21F8.3, F21F8.4, F21F8.5, F21F8.6, F21G4.3, F21H12.7, F21H7.10, F21H7.4, F21H7.5, F22A3.2, F22A3.6, F22A3.7, F22B3.5, F22B7.1, F22B7.4, F22D3.4, F22D6.10, F22D6.11, F22D6.12, F22D6.15, F22E12.1, F22E5.1, F22E5.17, F22E5.7, F22F4.1, F23B12.4, F23B2.11, F23B2.12, F23B2.5, F23C8.11, F23D12.7, F23F1.2, F23F1.4, F23F12.12, F23F12.8, F23H11.6, F23H11.7, F23H12.4, F23H12.5, F25A2.1, F25B4.9, F25D1.3, F25E2.2, F25E5.10, F25E5.3, F25E5.4, F25E5.7, F25F6.1, F25H2.7, F25H8.5, F25H9.1, F25H9.3, F26A1.12, F26A1.15, F26A1.2, F26A10.1, F26B1.1, F26B1.4, F26B1.8, F26C11.3, F26C11.4, F26D10.12, F26D10.13, F26D11.2, F26D11.4, F26D11.5, F26D11.9, F26D2.13, F26D2.14, F26D2.16, F26D2.3, F26E4.3, F26E4.7, F26F12.1, F26F12.8, F26F2.10, F26F2.6, F26F2.8, F26G1.1, F26G1.10, F26G1.11, F26G1.5, F26G1.9, F27B10.1, F27C1.3, F27C1.8, F27C8.4, F27E5.7, F28A10.2, F28A10.5, F28A10.8, F28A12.2, F28A12.4, F28B1.1, F28B1.2, F28B1.9, F28B3.4, F28B4.3, F28C6.10, F28C6.5, F28D1.3, F28D1.4, F28D1.5, F28E10.5, F28F9.3, F28G4.2, F28G4.3, F28G4.4, F28H7.3, F28H7.4, F28H7.7, F29A7.3, F29A7.7, F29A7.8, F29B9.5, F29B9.9, F29C12.1, F29C4.2, F29C4.4, F29D10.1, F29D10.2, F29D10.3, F30A10.11, F30A10.13, F30A10.14, F30B5.1, F30H5.2, F30H5.3, F30H5.5, F31A3.1, F31A9.4, F31D4.4, F31D4.6, F31D4.9, F31E8.5, F31F4.1, F31F4.11, F31F6.4, F31F6.7, F31F6.8, F31F7.3, F32A5.2, F32A5.4, F32A7.6, F32B4.1, F32B4.5, F32B5.2, F32B5.3, F32B5.8, F32D1.11, F32D1.4, F32D8.11, F32D8.2, F32D8.3, F32D8.7, F32D8.8, F32D8.9, F32E10.3, F32E10.8, F32G8.2, F32G8.3, F32G8.5, F32H2.11, F32H5.1, F32H5.3, F33A8.10, F33A8.2, F33A8.7, F33D11.3, F33D11.8, F33D4.3, F33D4.6, F33E2.3, F33E2.7, F33H1.6, F34D6.7, F34D6.8, F34D6.9, F34H10.2, F35A5.2, F35A5.3, F35A5.4, F35B12.3, F35B12.4, F35B12.6, F35B12.7, F35B3.4, F35C11.1, F35C11.4, F35C11.7, F35C5.1, F35C5.10, F35C5.12, F35C5.3, F35C5.5, F35C5.6, F35C5.7, F35C5.8, F35C5.9, F35D11.1, F35D11.10, F35D11.8, F35D11.9, F35D2.1, F35D2.2, F35D2.5, F35E12.10, F35E12.2, F35E12.5, F35E12.6, F35E12.7, F35E12.8, F35E2.5, F35E2.8, F35E2.9, F35E8.1, F35E8.10, F35E8.13, F35E8.6, F35E8.7, F35E8.9, F35F10.13, F35F10.5, F35G2.1, F35G2.3, F35G2.5, F35G8.2, F35H10.2, F35H8.1, F35H8.2, F36A4.1, F36A4.10, F36A4.2, F36A4.4, F36A4.8, F36D1.10, F36D1.12, F36D1.5, F36D1.6, F36D1.7, F36D3.14, F36D3.9, F36F12.1, F36F12.3, F36F12.7, F36F2.7, F36F2.8, F36G9.11, F36G9.15, F36H1.11, F36H12.1, F36H12.17, F36H12.3, F36H12.4, F36H12.5, F36H2.3, F36H5.6, F36H9.2, F36H9.7, F37A8.4, F37C12.10, F37C4.8, F38A1.1, F38A1.10, F38A1.14, F38A1.4, F38A6.4, F38A6.5, F38B2.2, F38B2.6, F38B6.2, F38B6.7, F38C2.6, F38E1.7, F38E11.4, F38E11.7, F38E9.2, F38G1.2, F39C12.4, F39D8.4, F39F10.5, F39G3.2, F39G3.3, F39G3.8, F39H2.1, F40A3.1, F40A3.3, F40C5.1, F40C5.3, F40E10.1, F40E10.4, F40E10.5, F40F11.4, F40F12.4, F40F4.2, F40F4.3, F40F4.4, F40F4.6, F40F4.8, F40F8.4, F40G12.2, F40G12.4, F40G12.5, F40G12.6, F40G9.10, F40G9.19, F40G9.5, F40G9.6, F40G9.7, F40H3.2, F40H3.3, F40H3.5, F41B4.2, F41C3.11, F41C3.6, F41C6.1, F41C6.3, F41C6.4, F41C6.6, F41D3.11, F41D3.7, F41D3.9, F41D9.2, F41D9.3, F41E6.11, F41E6.12, F41E6.15, F41E6.2, F41E6.6, F41E6.8, F41E7.4, F41E7.5, F41E7.7, F41E7.8, F41F3.3, F41F3.4, F41F3.8, F41G3.1, F41G3.12, F41G3.16, F41G3.17, F41G3.20, F41G3.21, F42A10.6, F42A10.7, F42A6.2, F42A8.1, F42A9.4, F42A9.7, F42C5.7, F42F12.1, F42F12.10, F42F12.2, F42F12.6, F42F12.7, F42F12.8, F42F12.9, F42G10.1, F42G9.4, F42H11.1, F43C11.1, F43C11.2, F43C11.3, F43C11.4, F44A2.3, F44B9.1, F44B9.10, F44D12.6, F44E7.6, F44F4.15, F45B8.3, F45C12.16, F45C12.9, F45D11.5, F45D3.3, F45D3.4, F45E1.3, F45E12.1, F45E12.6, F45E4.1, F45E4.5, F45E4.8, F45E6.4, F45G2.1, F45G2.5, F46A8.3, F46A8.4, F46A8.5, F46A8.8, F46B3.1, F46B3.15, F46B3.17, F46B3.2, F46B3.3, F46B3.4, F46B3.5, F46B3.9, F46B6.10, F46B6.13, F46B6.8, F46C3.6, F46C5.1, F46C5.10, F46C5.3, F46C5.4, F46C8.1, F46E10.2, F46F11.3, F46F11.7, F46F2.3, F46F5.10, F46F5.11, F46F5.14, F46F5.15, F46F5.7, F46G10.4, F46G11.2, F46G11.4, F46G11.6, F46H5.6, F46H6.2, F47B3.4, F47B7.1, F47B7.3, F47B7.4, F47B8.13, F47B8.14, F47C12.11, F47C12.2, F47C12.4, F47C12.6, F47C12.8, F47E1.1, F47F2.1, F47G3.1, F47G9.6, F48A9.3, F48B9.3, F48B9.4, F48C1.8, F48C1.9, F48C11.3, F48C5.2, F48E3.4, F48E8.1, F48E8.4, F48F7.3, F48F7.4, F48G7.7, F48G7.8, F49A5.4, F49A5.6, F49C12.10, F49C12.14, F49C12.5, F49C5.10, F49C5.11, F49C5.7, F49D11.11, F49D11.3, F49D11.6, F49E10.2, F49E10.3, F49E11.10, F49E11.11, F49E11.4, F49E11.5, F49E11.6, F49E12.1, F49E12.2, F49F1.10, F49F1.11, F49F1.5, F49F1.6, F49F1.7, F49F1.9, F49H12.4, F49H6.1, F49H6.12, F49H6.15, F49H6.2, F49H6.8, F52A8.3, F52B10.2, F52B11.4, F52B11.6, F52C9.5, F52D1.3, F52D2.5, F52E1.14, F52E1.2, F52E4.6, F52F12.2, F52G3.4, F52G3.5, F53B1.6, F53B3.2, F53B6.2, F53B6.8, F53B7.7, F53C11.1, F53C11.9, F53E10.3, F53E10.4, F53F1.4, F53F1.5, F53F4.13, F53F4.17, F53F4.7, F53F8.4, F53F8.7, F53G12.9, F53G2.2, F53G2.3, F53H4.2, F53H4.3, F54A5.2, F54B11.10, F54B11.11, F54B11.4, F54B3.4, F54B8.1, F54B8.5, F54C4.2, F54C9.2, F54C9.4, F54D10.8, F54D12.1, F54D12.10, F54D12.11, F54D12.7, F54D12.8, F54D12.9, F54D5.3, F54D5.4, F54D7.4, F54D8.1, F54D8.4, F54E2.1, F54E2.2, F54E4.3, F54E4.4, F54E7.6, F54F11.1, F54F11.2, F54F3.1, F54F3.3, F54F7.2, F54F7.3, F54F7.9, F54G8.1, F54H12.5, F55A11.1, F55A12.6, F55B11.2, F55B11.3, F55B11.4, F55B12.5, F55C10.2, F55C10.3, F55C10.4, F55C12.7, F55C9.6, F55D10.1, F55D12.5, F55F3.2, F55F3.4, F55F8.8, F55G11.2, F55G11.4, F55G11.5, F55G11.6, F55G11.7, F55G11.8, F55H12.4, F55H2.1, F56A11.3, F56A4.1, F56A4.2, F56A4.6, F56A4.9, F56A8.9, F56B3.1, F56B3.2, F56B6.6, F56C3.8, F56C4.1, F56C4.4, F56C9.7, F56C9.8, F56D1.4, F56D2.8, F56D3.1, F56D5.1, F56D5.6, F56D6.1, F56D6.15, F56D6.2, F56D6.8, F56D6.9, F56E10.2, F56F10.1, F56F11.1, F56F3.6, F56F4.8, F56F4.9, F56G4.2, F56G4.3, F56G4.7, F56H11.1, F56H6.1, F56H6.13, F56H6.2, F56H6.4, F56H6.6, F56H6.7, F56H6.8, F56H6.9, F56H9.2, F56H9.6, F57A8.6, F57A8.8, F57B7.2, F57B7.3, F57B7.4, F57C12.1, F57C2.4, F57C9.2, F57E7.1, F57E7.2, F57E7.4, F57F4.3, F57F4.4, F57G8.7, F57H12.3, F57H12.6, F58A4.1, F58A4.5, F58A6.4, F58B3.1, F58B3.2, F58B3.3, F58B3.8, F58B4.1, F58B4.3, F58B4.4, F58B4.6, F58B4.7, F58B6.1, F58D5.3, F58D5.6, F58E1.4, F58E1.7, F58E10.7, F58E2.2, F58E2.5, F58E6.13, F58E6.4, F58E6.7, F58F6.1, F58F6.2, F58F9.10, F58F9.11, F58F9.6, F58F9.8, F58F9.9, F58G1.4, F58G1.5, F58G1.8, F58G4.3, F58H1.2, F58H1.4, F58H1.7, F58H10.1, F58H7.1, F58H7.2, F59A1.16, F59A1.6, F59A6.10, F59A6.11, F59A6.12, F59A6.3, F59A7.1, F59A7.11, F59A7.2, F59A7.5, F59B1.2, F59B10.2, F59B10.5, F59B2.12, F59C6.16, F59C6.8, F59D12.3, F59D6.1, F59D6.2, F59D6.3, F59E11.5, F59E11.6, F59E11.7, F59E12.13, F59F5.4, F59F5.7, H01G02.1, H01M10.2, H01M10.3, H02F09.3, H02I12.1, H02I12.4, H02K04.1, H02K04.2, H03A11.1, H03E18.1, H05L03.3, H06A10.1, H06A10.2, H06I04.6, H06I04.7, H10D18.2, H10D18.4, H10E21.4, H12D21.1, H12D21.10, H12D21.12, H12D21.13, H12D21.14, H12D21.15, H12D21.2, H12D21.3, H12D21.6, H12I13.6, H13N06.2, H14A12.6, H14A12.7, H14N18.2, H14N18.3, H16D19.1, H17B01.5, H19M22.3, H20E11.2, H20E11.3, H22K11.2, H22K11.3, H23N18.4, H23N18.5, H25K10.1, H25K10.5, H27M09.4, H29C22.1, H31B20.2, H31G24.1, H32K16.2, H32K21.1, H34I24.1, H34I24.2, H34I24.3, H35N09.1, H36L18.1, H37A05.4, H38K22.3, H38K22.5, H38K22.6, H38K22.7, H39E23.2, H41C03.2, H42K12.3, H43E16.1, JC8.14, JC8.8, K01A12.4, K01A2.11, K01A6.4, K01A6.5, K01A6.7, K01A6.8, K01C8.2, K01C8.8, K01D12.10, K01D12.11, K01D12.12, K01D12.13, K01D12.14, K01D12.5, K01D12.8, K01D12.9, K02A11.3, K02A11.4, K02A2.5, K02A6.1, K02A6.4, K02B12.6, K02B12.9, K02B7.3, K02B7.4, K02C4.2, K02D3.1, K02D7.4, K02D7.6, K02E10.6, K02E11.10, K02E11.3, K02E11.4, K02E11.5, K02E11.6, K02E11.7, K02E2.2, K02E2.4, K02E7.1, K02F2.5, K02F3.3, K02F3.5, K02F3.9, K02F6.9, K02G10.4, K02H11.4, K03A11.6, K03B4.4, K03B4.7, K03B8.11, K03B8.14, K03B8.6, K03B8.7, K03D3.2, K03D3.5, K03H1.3, K03H1.4, K03H1.6, K03H6.2, K03H6.7, K03H9.2, K04A8.1, K04A8.5, K04A8.8, K04C2.5, K04E7.3, K04F1.10, K04F1.11, K04F1.13, K04F1.14, K04F1.7, K04F1.8, K04F1.9, K04H4.1, K04H4.2, K04H4.6, K04H4.7, K04H8.3, K05F6.11, K05F6.12, K05F6.4, K05F6.6, K06A4.1, K06A4.7, K06A9.1, K06B9.1, K06G5.1, K06H6.1, K06H6.2, K06H6.5, K07A1.4, K07A1.5, K07A1.6, K07A12.6, K07C11.3, K07C11.4, K07C11.5, K07C11.7, K07C11.8, K07C5.7, K07C5.9, K07C6.2, K07C6.3, K07C6.4, K07C6.5, K07D4.6, K07D4.8, K07D4.9, K07E12.1, K07E3.8, K07E8.1, K07G6.1, K07H8.11, K07H8.7, K08B12.1, K08B4.2, K08B4.6, K08B4.7, K08C7.3, K08C9.1, K08C9.10, K08C9.2, K08C9.4, K08C9.5, K08C9.6, K08D12.4, K08D12.5, K08D12.7, K08D8.3, K08D8.4, K08D8.5, K08D8.6, K08D9.2, K08E3.1, K08E3.2, K08F4.5, K08H2.10, K08H2.9, K09A11.2, K09A11.3, K09A11.4, K09A9.8, K09B11.10, K09B3.1, K09C4.8, K09C6.3, K09C6.9, K09C8.5, K09C8.6, K09C8.7, K09C8.9, K09D9.2, K09D9.9, K09E2.1, K09E4.4, K09E4.6, K09F5.2, K09F5.3, K09H11.4, K09H11.9, K09H9.3, K09H9.5, K10B2.3, K10B3.6, K10B4.1, K10B4.6, K10C2.1, K10C2.5, K10C2.8, K10C3.4, K10C9.1, K10C9.3, K10C9.9, K10D11.1, K10D11.2, K10D11.3, K10D11.6, K10D11.7, K10D3.4, K10D3.6, K10G6.2, K10G9.3, K10H10.10, K10H10.4, K11D12.1, K11D12.13, K11D12.7, K11E4.1, K11E4.3, K11G12.1, K11H12.10, K11H12.11, K11H12.4, K11H12.6, K11H12.7, K11H3.5, K12B6.3, K12B6.9, K12D12.3, K12D9.12, K12D9.14, K12G11.6, K12H4.7, K12H6.10, K12H6.9, LLC1.2, M01B2.1, M01B2.8, M01D7.1, M01D7.5, M01E11.4, M01G12.10, M01H9.2, M02D8.6, M02E1.2, M02F4.7, M02G9.1, M02G9.2, M02G9.3, M02H5.8, M03A1.7, M03B6.3, M03D4.3, M03E7.4, M03F4.6, M03F8.4, M03F8.5, M03F8.6, M04B2.6, M04D8.1, M04D8.2, M04D8.3, M04G12.2, M05B5.7, M05D6.4, M05D6.8, M05D6.9, M110.1, M110.8, M110.9, M116.2, M153.3, M153.5, M162.2, M163.8, M176.10, M176.5, M18.1, M199.3, M199.4, M199.6, M199.7, M199.9, M28.6, M6.4, M60.2, M7.10, M7.9, M70.2, M70.4, M79.4, R01B10.1, R01E6.2, R01E6.7, R01H10.4, R01H2.2, R02D3.6, R02D5.10, R02D5.7, R02D5.9, R02F11.1, R02F2.4, R03A10.2, R03C1.1, R03G8.1, R03G8.3, R03G8.4, R03G8.6, R03H10.4, R03H10.5, R04B3.3, R04D3.2, R04D3.3, R05A10.1, R05A10.2, R05A10.3, R05A10.4, R05A10.5, R05A10.6, R05A10.7, R05A10.8, R05D3.9, R05D7.7, R05D8.11, R05G6.9, R05G9R.1, R05H5.7, R06A4.6, R06B10.3, R06C7.4, R06F6.11, R06F6.7, R07B1.11, R07B1.13, R07B1.5, R07B1.6, R07B1.8, R07C3.1, R07C3.3, R07E3.6, R07E3.7, R07E5.4, R07G3.2, R07G3.5, R07G3.9, R08A2.1, R08B4.1, R08B4.5, R08C7.1, R08C7.4, R08C7.6, R08E3.2, R08F11.7, R08H2.10, R09A1.5, R09B5.12, R09B5.5, R09D1.13, R09D1.8, R09E10.13, R09E10.8, R09F10.2, R09F10.7, R09F10.8, R09F10.9, R09H10.1, R09H10.2, R09H10.5, R102.11, R10D12.15, R10E11.4, R10H1.4, R10H1.5, R10H10.3, R11.2, R11.3, R11A5.6, R11A8.3, R11D1.12, R11D1.13, R11D1.3, R11D1.4, R11G10.4, R11G11.14, R11G11.6, R11G11.7, R12A1.3, R12A1.4, R12C12.10, R12C12.8, R12E2.13, R12E2.14, R12E2.15, R12E2.6, R12E2.7, R12E2.8, R13A1.1, R13A1.5, R13A5.3, R13A5.6, R13D11.10, R13D7.2, R13F6.2, R13F6.8, R13H4.3, R13H4.8, R148.3, R151.5, R155.5, R160.5, R17.3, R173.4, R52.4, R52.5, R52.6, R53.8, R57.2, R90.2, T01B10.1, T01B10.2, T01B6.4, T01B7.7, T01B7.8, T01B7.9, T01C3.4, T01D1.3, T01D1.6, T01D3.1, T01D3.3, T01D3.6, T01E8.8, T02B11.3, T02B11.4, T02B11.7, T02B11.8, T02B11.9, T02C12.5, T02D1.7, T02D1.9, T02E1.8, T02E9.2, T02E9.5, T02E9.6, T02G6.7, T02H6.10, T02H6.3, T02H6.4, T02H6.6, T02H6.7, T02H6.8, T02H6.9, T03D8.3, T03D8.4, T03D8.7, T03F1.10, T03F6.10, T03G11.10, T03G11.8, T03G6.3, T04A6.3, T04B2.8, T04B8.1, T04B8.2, T04C12.3, T04F3.2, T04F3.4, T04F3.5, T04F8.8, T04G9.7, T04H1.3, T04H1.6, T05A10.3, T05A10.5, T05A10.6, T05A6.4, T05A7.2, T05A7.7, T05A8.2, T05A8.6, T05B11.4, T05B4.10, T05B4.11, T05B4.9, T05C1.1, T05C1.5, T05C12.10, T05C3.4, T05C3.6, T05D4.4, T05E11.8, T05E12.3, T05E12.6, T05E12.8, T05E8.2, T05G5.1, T05G5.4, T05H10.3, T06A1.1, T06A1.5, T06A1.7, T06C10.4, T06C12.14, T06D4.3, T06D8.1, T06D8.10, T06E4.10, T06E4.12, T06E4.14, T06E4.4, T06E4.6, T06E4.8, T06E6.10, T06G6.12, T06G6.6, T07A5.5, T07A9.15, T07C12.10, T07C12.13, T07C12.15, T07C12.7, T07C4.4, T07C4.5, T07D10.1, T07D10.4, T07D10.6, T07D3.2, T07E3.2, T07F10.6, T07G12.3, T07H3.4, T07H3.5, T07H3.7, T07H6.3, T08A9.11, T08A9.12, T08A9.2, T08A9.7, T08A9.8, T08A9.9, T08B2.12, T08B2.2, T08B6.9, T08G2.2, T08G3.11, T08G3.6, T08G5.12, T08G5.15, T08G5.3, T08H10.3, T09A5.14, T09B9.3, T09D3.3, T09D3.8, T09E11.1, T09E11.10, T09E11.3, T09E11.8, T09E11.9, T09F5.1, T09F5.9, T10B10.1, T10B10.6, T10B5.7, T10B9.9, T10D4.1, T10D4.13, T10D4.15, T10D4.4, T10E10.5, T10E9.3, T10G3.1, T10H4.10, T10H4.11, T10H4.12, T11B7.3, T11F1.2, T11F1.6, T11F1.8, T11F1.9, T11F8.5, T11F9.13, T11F9.22, T11F9.3, T11F9.5, T11F9.6, T11F9.9, T12A2.16, T12A7.2, T12A7.3, T12A7.4, T12A7.5, T12B5.10, T12B5.15, T12D8.5, T12D8.9, T12E12.6, T12G3.8, T13A10.1, T13A10.5, T13B5.3, T13B5.5, T13B5.6, T13B5.7, T13C2.2, T13C2.3, T13C5.3, T13C5.7, T13F2.4, T13F3.6, T13F3.8, T13H5.3, T14A8.2, T14B4.7, T14B4.9, T14G10.3, T14G10.4, T15B7.1, T15B7.7, T15D6.1, T15D6.10, T15D6.12, T15D6.3, T15D6.8, T15D6.9, T16A1.3, T16A1.4, T16A9.4, T16G1.1, T16G1.2, T16G1.8, T16G12.1, T16H12.9, T17A3.12, T17H7.1, T17H7.7, T18H9.1, T19A5.1, T19A5.3, T19B10.12, T19B10.2, T19B10.3, T19B10.9, T19B4.1, T19C3.2, T19C3.5, T19C3.9, T19C4.7, T19C9.5, T19D12.1, T19D12.6, T19D7.3, T19H12.12, T19H12.3, T19H5.7, T20B12.5, T20B3.14, T20B3.16, T20B3.7, T20B6.3, T20D3.1, T20D3.2, T20D4.10, T20D4.11, T20D4.12, T20D4.16, T20D4.17, T20D4.19, T20D4.20, T20D4.8, T20F10.2, T20F7.3, T20G5.12, T20G5.13, T20G5.7, T20G5.8, T21B4.3, T21B6.2, T21B6.3, T21C12.4, T21C9.6, T21C9.8, T21C9.9, T21D11.1, T21D12.12, T21D12.14, T21D12.2, T21D9.1, T21E3.2, T21G5.6, T21H3.1, T22A3.3, T22A3.8, T22B11.1, T22C1.12, T22C8.2, T22C8.6, T22D1.2, T22E5.1, T22E5.3, T22F7.3, T22F7.4, T22G5.1, T22G5.7, T22H6.1, T22H6.5, T22H6.7, T23B12.11, T23B12.8, T23B3.5, T23B7.3, T23D5.3, T23E1.1, T23E7.4, T23F1.6, T23F11.6, T23F2.4, T23F4.4, T23F6.1, T23F6.2, T23F6.5, T23G4.5, T23G7.5, T23H2.4, T23H4.3, T24A11.3, T24A6.15, T24A6.16, T24A6.18, T24A6.19, T24B8.5, T24D8.3, T24D8.4, T24D8.5, T24D8.6, T24F1.5, T24F1.6, T24H10.4, T24H7.2, T25B2.3, T25B6.2, T25B6.3, T25B9.11, T25B9.3, T25C12.2, T25C12.3, T25C12.4, T25D10.1, T25D10.3, T25D3.3, T25E12.14, T25E4.1, T25F10.2, T25G12.11, T26A8.3, T26C11.9, T26C12.1, T26C12.6, T26C5.2, T26C5.4, T26C5.5, T26E3.10, T26E3.6, T26E3.8, T26E4.1, T26E4.10, T26E4.2, T26E4.3, T26E4.5, T26E4.9, T26F2.1, T26H5.8, T27A1.3, T27A8.1, T27C5.12, T27C5.7, T27D12.3, T27E4.4, T27E4.5, T27E7.1, T27F6.1, T27F6.2, T28A11.16, T28A11.17, T28A11.18, T28A11.19, T28A11.2, T28A11.3, T28A11.4, T28A11.5, T28B4.3, T28B8.2, T28C6.1, T28C6.4, T28C6.6, T28D6.3, T28D6.5, T28D9.12, T28H10.2, T28H10.4, VB0395L.1, VC5.2, VC5.3, VK10D6R.1, VZK822L.2, W01A11.1, W01A8.6, W01A8.7, W01A8.8, W01B11.5, W01B6.1, W01C8.5, W01F3.1, W01F3.2, W01F3.3, W02A2.2, W02A2.3, W02B12.1, W02B12.11, W02B12.13, W02B12.4, W02B3.7, W02B8.5, W02D3.3, W02D7.10, W02D7.12, W02D7.2, W02D9.5, W02D9.7, W03A5.3, W03C9.1, W03D2.1, W03D2.5, W03D2.9, W03D8.1, W03D8.11, W03F11.1, W03F11.5, W03F8.5, W03F9.11, W03G1.7, W04A4.2, W04A4.3, W04C9.2, W04E12.6, W04E12.8, W04G3.10, W04G3.12, W04G3.13, W04G3.2, W04G3.3, W04G3.8, W04G5.10, W05B10.3, W05B2.2, W05B2.5, W05B2.6, W05E7.3, W05F2.7, W05G11.3, W06A11.1, W06A7.5, W07B8.1, W07B8.4, W07E11.2, W07E11.3, W07E11.4, W07G1.7, W07G4.1, W07G4.7, W07G9.2, W08A12.2, W08A12.3, W08D2.6, W08E12.2, W08E12.3, W08E12.4, W08E12.5, W08E12.6, W08F4.5, W08F4.7, W08G11.1, W09C2.10, W09C2.7, W09C2.8, W09C2.9, W09C3.2, W09C3.7, W09C3.8, W09C5.11, W09C5.4, W09D6.4, W09G10.5, W09G12.1, W09G12.4, W09G12.9, W09G3.3, W10D9.2, W10G11.1, W10G11.11, W10G11.12, W10G11.13, W10G11.14, W10G11.15, W10G11.19, W10G11.2, W10G11.3, W10G11.4, Y102A11A.5, Y102A5B.2, Y102A5B.3, Y102A5C.27, Y102A5C.4, Y102A5C.40, Y102E9.3, Y102E9.5, Y102E9.6, Y105C5A.17, Y105C5A.3, Y105C5A.4, Y105C5A.5, Y105C5A.6, Y105C5A.8, Y105C5B.15, Y105C5B.18, Y105C5B.25, Y105C5B.3, Y105E8A.2, Y106G6D.6, Y106G6D.8, Y106G6H.1, Y110A2AL.3, Y110A2AL.4, Y110A2AL.5, Y110A2AL.6, Y110A2AL.7, Y110A2AL.9, Y110A7A.7, Y111B2A.15, Y111B2A.2, Y113G7A.15, Y113G7B.15, Y116A8A.2, Y116A8A.3, Y116A8A.4, Y116A8A.6, Y116A8A.7, Y116A8B.1, Y116A8C.1, Y116A8C.21, Y116A8C.26, Y116A8C.29, Y116A8C.3, Y116A8C.33, Y116A8C.4, Y116A8C.43, Y116A8C.44, Y116A8C.5, Y116F11A.3, Y116F11B.1, Y116F11B.13, Y116F11B.2, Y116F11B.3, Y116F11B.8, Y116F11B.9, Y119C1B.6, Y119C1B.9, Y119D3B.13, Y11D7A.5, Y11D7A.8, Y12A6A.1, Y12A6A.2, Y16B4A.2, Y17D7B.5, Y17D7B.6, Y17D7B.8, Y17D7C.2, Y17G9A.2, Y17G9A.3, Y18D10A.10, Y18D10A.2, Y18D10A.24, Y18H1A.10, Y18H1A.13, Y18H1A.8, Y19D10A.6, Y19D10A.7, Y19D10A.9, Y19D10B.3, Y19D10B.4, Y19D10B.6, Y1A5A.2, Y20C6A.4, Y22D7AL.13, Y22D7AL.14, Y22D7AL.6, Y22D7AR.10, Y22F5A.4, Y22F5A.5, Y23B4A.2, Y23H5B.1, Y23H5B.3, Y23H5B.8, Y25C1A.1, Y25C1A.2, Y25C1A.3, Y25C1A.4, Y26D4A.12, Y26D4A.2, Y26D4A.6, Y26G10.5, Y26G10.6, Y26G10.7, Y27F2A.6, Y2H9A.3, Y32F6A.5, Y32G9A.12, Y32G9A.2, Y32G9A.5, Y34B4A.10, Y34B4A.3, Y34B4A.5, Y34B4A.6, Y34B4A.9, Y34D9A.11, Y34F4.3, Y37A1B.7, Y37A1B.8, Y37A1B.9, Y37B11A.1, Y37D8A.15, Y37D8A.19, Y37D8A.2, Y37D8A.3, Y37E11AL.4, Y37E11AL.6, Y37E11AR.6, Y37E11AR.7, Y37E11B.7, Y37E3.13, Y37E3.19, Y37F4.3, Y37H2A.10, Y37H2A.11, Y37H2A.13, Y38C1AA.9, Y38C1AB.1, Y38C1AB.5, Y38C1BA.3, Y38C9B.1, Y38E10A.1, Y38E10A.10, Y38E10A.11, Y38E10A.12, Y38E10A.13, Y38E10A.15, Y38E10A.16, Y38E10A.25, Y38E10A.26, Y38E10A.29, Y38E10A.4, Y38E10A.5, Y38E10A.7, Y38E10A.9, Y38H6A.1, Y38H6A.2, Y38H6A.3, Y38H6A.5, Y38H6C.15, Y38H6C.19, Y38H6C.22, Y38H6C.23, Y38H6C.8, Y38H8A.1, Y39A1A.21, Y39A1A.24, Y39A1A.7, Y39A1B.1, Y39A1C.1, Y39A1C.4, Y39A3A.2, Y39A3A.5, Y39A3B.1, Y39A3CL.6, Y39B6A.48, Y39B6A.5, Y39B6A.9, Y39E4B.12, Y39E4B.13, Y39E4B.8, Y39F10A.1, Y39F10A.3, Y39F10B.1, Y39F10C.1, Y39F10C.2, Y39G10AL.1, Y39G8B.10, Y39G8B.7, Y39G8B.9, Y39G8C.4, Y39H10A.1, Y40B10B.1, Y40C5A.3, Y40H7A.10, Y40H7A.3, Y40H7A.4, Y41C4A.1, Y41C4A.16, Y41C4A.22, Y41C4A.6, Y41C4A.8, Y41D4A.1, Y41D4A.7, Y41D4B.10, Y41D4B.15, Y41D4B.16, Y41D4B.26, Y41D4B.6, Y41E3.1, Y41E3.22, Y41G9A.10, Y41G9A.5, Y42A5A.2, Y42A5A.3, Y42G9A.2, Y42H9AR.5, Y42H9B.1, Y43B11AR.1, Y43C5A.4, Y43F4A.1, Y43F8A.1, Y43F8A.2, Y43F8B.11, Y43F8B.18, Y43F8B.20, Y43F8B.3, Y43F8B.5, Y43F8C.1, Y43F8C.15, Y43F8C.16, Y43F8C.17, Y43F8C.2, Y43F8C.20, Y43F8C.23, Y43F8C.5, Y43F8C.7, Y43F8C.9, Y44A6B.3, Y44A6B.4, Y44E3A.2, Y44E3B.2, Y45F10A.5, Y45F10C.2, Y45F10C.4, Y45F10D.14, Y45F10D.6, Y45F3A.4, Y45G12C.11, Y45G5AM.6, Y46C8AL.1, Y46C8AL.2, Y46C8AL.3, Y46C8AL.4, Y46C8AL.5, Y46C8AL.8, Y46C8AL.9, Y46C8AR.1, Y46C8AR.3, Y46D2A.2, Y46E12A.1, Y46E12A.5, Y46G5A.14, Y46G5A.23, Y46G5A.29, Y46G5A.34, Y46G5A.39, Y46G5A.7, Y46H3B.1, Y46H3B.2, Y46H3C.7, Y46H3D.1, Y46H3D.8, Y47A7.2, Y47D3A.23, Y47D3B.12, Y47D3B.2, Y47D3B.3, Y47D3B.4, Y47D3B.6, Y47D7A.10, Y47D7A.11, Y47D7A.12, Y47D7A.2, Y47D7A.5, Y47D7A.6, Y47D7A.7, Y47D7A.9, Y47G6A.15, Y47G6A.29, Y47G6A.33, Y47H10A.2, Y47H9B.2, Y47H9C.1, Y48A6A.1, Y48A6B.4, Y48B6A.7, Y48B6A.8, Y48B6A.9, Y48D7A.1, Y48D7A.2, Y48E1B.16, Y48E1B.8, Y48E1B.9, Y48G10A.6, Y48G1BL.7, Y48G1BR.1, Y48G1C.13, Y48G8AL.12, Y49A3A.4, Y49E10.10, Y49E10.16, Y49E10.18, Y49E10.25, Y49E10.4, Y49F6B.12, Y49F6B.13, Y49F6B.6, Y49F6C.5, Y49F6C.6, Y49G5A.1, Y49G5B.1, Y4C6A.4, Y4C6B.7, Y50D4B.6, Y50D7A.13, Y50D7A.5, Y50E8A.1, Y50E8A.10, Y50E8A.4, Y50E8A.5, Y50E8A.7, Y51A2A.11, Y51A2A.12, Y51A2A.4, Y51A2A.6, Y51A2A.7, Y51A2B.4, Y51A2B.6, Y51A2B.9, Y51A2D.1, Y51A2D.10, Y51A2D.11, Y51A2D.14, Y51A2D.8, Y51A2D.9, Y51B9A.8, Y51F10.5, Y51F10.7, Y51H4A.1, Y51H4A.10, Y51H4A.12, Y51H4A.22, Y51H4A.32, Y51H4A.5, Y51H4A.8, Y51H4A.935, Y51H7C.1, Y51H7C.13, Y51H7C.15, Y51H7C.2, Y52B11A.5, Y52B11A.8, Y52B11B.1, Y53C10A.10, Y53C10A.15, Y53C12B.6, Y53C12B.7, Y53F4B.14, Y53F4B.18, Y53F4B.27, Y53G8AM.5, Y53G8AM.6, Y53G8AR.1, Y53H1A.4, Y53H1B.2, Y54E10A.17, Y54E10BL.2, Y54E2A.9, Y54F10AM.6, Y54F10BM.12, Y54F10BM.3, Y54G11A.1, Y54G2A.10, Y54G2A.14, Y54G2A.15, Y54G2A.28, Y54G2A.29, Y54G2A.32, Y54G2A.33, Y54G2A.39, Y54G2A.44, Y54G2A.45, Y54G2A.57, Y54G2A.6, Y54G2A.8, Y54G2A.9, Y55B1BL.1, Y55F3AM.11, Y55F3BR.11, Y55F3BR.2, Y55F3BR.8, Y56A3A.12, Y56A3A.14, Y57A10A.11, Y57A10A.23, Y57A10A.24, Y57A10B.3, Y57E12AR.1, Y57E12B.3, Y57G11A.2, Y57G11B.2, Y57G11B.3, Y57G11B.5, Y57G11C.1130, Y57G11C.18, Y57G11C.38, Y57G11C.39, Y57G11C.40, Y57G11C.41, Y57G11C.42, Y57G11C.52, Y57G11C.8, Y57G7A.1, Y57G7A.3, Y58G8A.5, Y59A8B.19, Y59A8B.20, Y59A8B.26, Y59C2A.1, Y59E9AL.1, Y59E9AL.6, Y59E9AR.10, Y59E9AR.4, Y59E9AR.9, Y59H11AM.4, Y59H11AR.5, Y5F2A.1, Y5F2A.2, Y5H2A.1, Y5H2A.3, Y5H2B.5, Y60A3A.21, Y60A3A.23, Y62E10A.13, Y62E10A.19, Y62E10A.3, Y62F5A.9, Y62H9A.3, Y62H9A.4, Y62H9A.5, Y62H9A.6, Y64G10A.1, Y64G10A.2, Y64G10A.7, Y64H9A.2, Y65A5A.2, Y65B4A.2, Y65B4BL.6, Y65B4BR.1, Y65B4BR.2, Y65B4BR.6, Y66A7A.6, Y66A7A.9, Y66H1A.5, Y67A10A.10, Y67A10A.11, Y67A10A.2, Y67D8B.5, Y67D8C.7, Y67D8C.8, Y68A4B.1, Y69A2AL.1, Y69A2AR.22, Y69A2AR.5, Y69F12A.3, Y69H2.10, Y69H2.3, Y6E2A.8, Y6G8.14, Y6G8.15, Y6G8.16, Y70C5C.2, Y70C5C.3, Y70C5C.5, Y70D2A.2, Y71A12B.6, Y71F9AM.8, Y71F9B.15, Y71F9B.9, Y71G12B.18, Y71G12B.20, Y71G12B.21, Y71G12B.4, Y71G12B.5, Y71H2B.4, Y73B3B.3, Y73B6A.3, Y73B6BL.1, Y73B6BL.24, Y73B6BL.25, Y73B6BL.34, Y73B6BL.35, Y73B6BL.37, Y73B6BL.44, Y73C8C.12, Y73C8C.2, Y73C8C.4, Y73E7A.8, Y73F4A.1, Y73F4A.2, Y73F4A.3, Y73F8A.10, Y73F8A.18, Y73F8A.22, Y73F8A.23, Y73F8A.26, Y73F8A.35, Y73F8A.6, Y73F8A.8, Y73F8A.9, Y74C10AR.2, Y74C9A.2, Y75B12B.11, Y75B12B.13, Y75B12B.8, Y75B7AR.1, Y75B8A.11, Y75B8A.20, Y75B8A.3, Y75B8A.44, Y75B8A.9, Y76A2B.2, Y76B12C.3, Y76B12C.8, Y77E11A.15, Y7A5A.9, Y80D3A.10, Y81G3A.4, Y82E9BR.4, Y87G2A.15, Y87G2A.16, Y8A9A.2, Y8A9A.6, Y8G1A.1, Y94A7B.11, Y95B8A.2, Y97E10AL.2, Y97E10B.1, ZC101.2, ZC13.4, ZC15.2, ZC15.5, ZC15.8, ZC155.5, ZC168.2, ZC168.5, ZC204.1, ZC204.17, ZC204.6, ZC21.3, ZC21.8, ZC239.22, ZC250.1, ZC328.5, ZC334.1, ZC334.10, ZC334.11, ZC334.13, ZC334.2, ZC334.3, ZC334.7, ZC334.8, ZC334.9, ZC373.2, ZC373.6, ZC373.7, ZC374.2, ZC376.8, ZC412.10, ZC412.3, ZC412.6, ZC412.7, ZC412.8, ZC412.9, ZC434.3, ZC449.1, ZC449.2, ZC455.10, ZC482.2, ZC482.4, ZC487.4, ZC487.5, ZC504.1, ZC513.8, ZC64.2, ZC84.1, ZC84.6, ZK1010.4, ZK1025.2, ZK1025.7, ZK1025.8, ZK1037.1, ZK1037.10, ZK105.1, ZK105.3, ZK105.6, ZK1055.5, ZK1067.7, ZK1098.9, ZK112.1, ZK112.3, ZK1193.1, ZK1225.1, ZK1225.4, ZK1225.5, ZK1248.1, ZK1248.16, ZK1251.11, ZK1251.2, ZK1290.1, ZK1290.10, ZK1290.11, ZK1290.12, ZK1290.7, ZK1290.8, ZK1307.2, ZK1320.10, ZK1320.2, ZK1320.3, ZK1321.1, ZK154.1, ZK154.4, ZK177.2, ZK180.5, ZK180.6, ZK218.1, ZK218.11, ZK218.3, ZK218.5, ZK218.7, ZK250.2, ZK262.2, ZK262.3, ZK265.2, ZK265.7, ZK265.8, ZK285.2, ZK287.3, ZK287.4, ZK287.8, ZK337.1, ZK337.5, ZK353.10, ZK353.5, ZK354.3, ZK355.1, ZK355.4, ZK355.5, ZK377.1, ZK381.2, ZK384.1, ZK384.2, ZK39.3, ZK39.4, ZK39.6, ZK39.7, ZK39.8, ZK39.9, ZK402.2, ZK430.8, ZK455.4, ZK455.5, ZK470.6, ZK488.10, ZK488.7, ZK507.4, ZK512.10, ZK512.7, ZK525.1, ZK546.15, ZK546.5, ZK593.2, ZK596.1, ZK6.10, ZK6.11, ZK6.3, ZK6.7, ZK616.9, ZK617.2, ZK622.5, ZK643.6, ZK643.8, ZK662.2, ZK662.6, ZK666.11, ZK666.3, ZK666.5, ZK666.6, ZK666.7, ZK669.1, ZK673.1, ZK673.9, ZK675.4, ZK678.5, ZK682.4, ZK688.1, ZK688.4, ZK688.8, ZK75.1, ZK75.3, ZK813.1, ZK813.2, ZK813.7, ZK816.1, ZK816.4, ZK822.2, ZK822.4, ZK829.3, ZK836.1, ZK84.1, ZK84.3, ZK84.6, ZK849.6, ZK856.4, ZK856.6, ZK856.7, ZK858.3, ZK863.2, ZK863.9, ZK867.3, ZK896.1, ZK896.4, ZK896.5, ZK896.6, ZK896.7, ZK897.1, ZK909.6, ZK970.7, ZK973.11, ZK973.4, ZK994.1, ZK994.3

**putative secreted proteins without domain (1593)**

AH6.3, B0034.1, B0034.4, B0205.10, B0205.12, B0205.13, B0207.2, B0213.17, B0213.3, B0213.4, B0213.5, B0213.6, B0222.11, B0228.1, B0228.8, B0238.15, B0250.2, B0252.8, B0261.5, B0280.7, B0294.1, B0310.6, B0334.13, B0344.2, B0379.2, B0379.7, B0393.9, B0403.3, B0403.5, B0410.3, B0416.2, B0432.11, B0432.14, B0457.2, B0478.3, B0496.11, B0507.5, B0513.4, B0524.5, B0554.1, B0563.10, B0563.9, BE10.4, C01A2.7, C01A2.9, C01B10.6, C01C4.1, C01F1.5, C01G10.15, C01G10.16, C01G10.17, C01G10.18, C01G10.4, C01G10.5, C01G10.6, C01G12.10, C01G12.11, C01G12.13, C01G12.2, C01G12.6, C01G12.9, C01G6.3, C01G6.9, C01H6.8, C02A12.4, C02B4.4, C02E7.6, C02E7.7, C02F12.3, C02F4.4, C02F5.14, C03A7.12, C03A7.13, C03B1.6, C03G5.10, C03G5.11, C03G5.12, C03G5.13, C03G5.2, C03G5.8, C03G5.9, C04E6.13, C04F12.5, C04G6.10, C04G6.7, C04H5.7, C04H5.8, C05B5.1, C05B5.11, C05B5.3, C05C10.8, C05D12.3, C05D2.8, C05D9.4, C05D9.9, C05E11.6, C05E11.7, C05E11.8, C05E4.7, C05E7.1, C05E7.2, C05E7.3, C06A12.8, C06C3.10, C06C3.4, C06C3.9, C06E1.5, C06E1.6, C06E2.9, C06E4.2, C06E7.2, C06E7.6, C06E7.88, C06G1.2, C07A12.2, C07B5.3, C07E3.10, C07G3.10, C08B6.10, C08B6.6, C08E3.14, C08E8.11, C09B7.2, C09D4.2, C09E7.2, C09G1.5, C09G12.17, C09G12.5, C09G5.8, C09G9.8, C09H10.5, C10C5.7, C10F3.1, C10F3.7, C10G11.10, C11H1.7, C12D12.1, C12D5.3, C12D5.5, C12D8.15, C13A10.2, C13A2.2, C13C12.2, C13C4.7, C14A4.9, C14B1.9, C14C6.3, C14F11.7, C15B12.4, C15F1.2, C15H11.10, C15H11.13, C15H9.11, C15H9.9, C16C8.10, C16C8.8, C16C8.9, C16D9.8, C16D9.9, C17B7.12, C17C3.13, C17C3.18, C17C3.2, C17C3.4, C17F3.3, C17F4.12, C17F4.2, C17F4.3, C17F4.7, C17G1.5, C17G10.5, C17H12.11, C18A11.2, C18D1.3, C18F10.2, C18H7.7, C18H9.1, C23G10.11, C23H5.8, C24A1.1, C24A11.5, C24A3.2, C25A8.2, C25D7.15, C25D7.5, C25E10.11, C25E10.13, C25F6.6, C25F6.8, C25F9.8, C25G6.4, C25H3.15, C25H3.5, C26B9.2, C26B9.3, C26D10.6, C26F1.1, C26F1.10, C27A2.5, C27A2.8, C27B7.9, C27C12.3, C27D6.12, C27F2.9, C28C12.1, C28D4.10, C28H8.2, C28H8.7, C29E4.14, C29E4.17, C29F3.3, C29F5.8, C29G2.6, C30E1.4, C30F2.3, C30F2.4, C30F8.3, C30G7.4, C30H6.10, C31H1.2, C31H1.5, C31H1.7, C31H5.7, C32A9.1, C32E12.3, C32E8.12, C32E8.4, C33A12.19, C33A12.2, C33B4.5, C33G3.5, C33G8.2, C33G8.3, C33G8.4, C34B7.1, C34D4.10, C34D4.11, C34E7.4, C34F11.8, C35A11.2, C35A11.3, C35A5.11, C35A5.6, C35B1.4, C35B1.7, C35C5.8, C35E7.7, C36B1.8, C37A5.8, C37H5.10, C37H5.11, C37H5.4, C38C10.6, C38C3.10, C38C3.6, C38C6.3, C38C6.5, C38C6.6, C38H2.2, C39B10.7, C39B5.5, C39E6.1, C39E9.8, C40A11.10, C40C9.3, C40H5.1, C41C4.9, C41G11.1, C41G6.13, C41G7.6, C42D4.13, C43F9.11, C43F9.7, C43G2.3, C44B11.4, C44B12.1, C44B12.3, C44B12.5, C44B12.9, C44C1.2, C44C1.6, C44E4.8, C44H9.5, C45B2.1, C45B2.2, C45B2.3, C45E5.4, C45G9.10, C45G9.12, C45G9.13, C45G9.6, C46C2.5, C46C2.6, C46F2.1, C47D2.1, C47E12.14, C47E12.9, C47E8.11, C47F8.7, C48B4.12, C48B4.13, C48D1.5, C48E7.7, C49A1.1, C49C3.20, C49C3.9, C49C8.6, C49G9.2, C49H3.12, C50D2.1, C50D2.6, C50E3.15, C50E3.6, C50F2.10, C50F2.4, C50F2.9, C50F4.8, C50F7.2, C50F7.5, C50F7.9, C50H2.10, C51E3.10, C52E2.2, C53A5.2, C53D6.4, C53D6.8, C54C6.5, C54C8.12, C54C8.2, C54C8.6, C54C8.9, C54D1.7, C54D10.13, C54D10.2, C54D10.9, C54D2.2, C54D2.6, C54F6.12, C54F6.15, C54F6.5, C54F6.6, C55A1.4, C55A1.6, C55A1.7, C55C3.3, C55C3.5, C56G2.4, C56G3.2, CC4.2, CD4.11, D1007.13, D1007.14, D1007.18, D1007.19, D1009.4, D1022.2, D1025.4, D1025.6, D1025.7, D1025.8, D1025.9, D1054.10, D1054.11, D1081.10, D1081.12, D1086.10, D1086.11, D1086.12, D1086.17, D1086.18, D1086.2, D1086.5, D1086.6, D1086.7, D2005.2, D2007.1, D2024.4, D2062.13, D2062.6, D2092.8, D2096.10, D2096.13, D2096.5, D2096.6, D2096.9, DY3.5, E01G4.6, E01H11.3, E02A10.4, E02H4.7, E02H9.1, E02H9.7, E02H9.9, E03D2.1, E03D2.2, E03H12.5, E03H12.7, E04A4.6, E04D5.5, E04F6.6, E04F6.8, E04F6.9, EGAP4.1, EGAP5.1, F01D4.9, F01F1.15, F01G10.6, F02D8.5, F02H6.6, F07C4.7, F07F6.5, F07G6.10, F07G6.2, F08B12.4, F08B4.4, F08F3.1, F08F3.10, F08G12.11, F08G12.8, F08G2.8, F09A5.3, F09B12.1, F09B9.4, F09C6.10, F09E10.1, F09E5.14, F09E5.16, F09E8.8, F09F7.8, F09F9.2, F09F9.3, F10C1.3, F10D2.8, F10E9.12, F10F2.9, F10G2.2, F11A5.17, F11C7.5, F11E6.4, F11E6.9, F11F1.6, F11G11.14, F12A10.1, F12A10.7, F13A2.1, F13A2.5, F13B6.1, F13B6.3, F13B9.2, F13C5.3, F13D12.3, F13E9.10, F13E9.12, F13E9.15, F13E9.2, F13E9.3, F13E9.9, F13G11.3, F13G3.12, F13H10.9, F14B6.1, F14B8.2, F14B8.4, F14D2.5, F14D7.10, F14D7.14, F14D7.5, F14D7.7, F14F11.2, F14F3.4, F14F3.5, F14F8.8, F14F9.2, F14H3.14, F14H3.5, F14H8.8, F15B9.11, F15D4.8, F15E11.11, F15E6.10, F15E6.3, F15E6.4, F15G9.5, F15H10.10, F15H10.5, F15H10.6, F15H10.9, F16B3.3, F16B4.4, F16B4.7, F16C3.2, F16C3.4, F16F9.2, F17B5.9, F17C11.2, F17E9.11, F17E9.15, F17E9.2, F17E9.3, F17E9.4, F18A1.7, F18A12.3, F18C5.5, F18E9.2, F18E9.3, F18E9.4, F18E9.7, F19B10.6, F19F10.8, F19H8.2, F20A1.1, F20A1.10, F20A1.6, F20A1.8, F20B10.3, F20B6.4, F21A3.8, F21C10.11, F21C10.5, F21C10.8, F21D12.2, F21D9.3, F21E9.4, F21E9.6, F21F8.5, F21F8.6, F21H12.7, F21H7.10, F22B3.5, F22B7.1, F22B7.4, F22D3.4, F22D6.15, F22E5.7, F22F4.1, F23C8.11, F23D12.7, F23F1.4, F23F12.12, F23H11.6, F23H11.7, F25E2.2, F25H8.5, F26A10.1, F26B1.1, F26B1.8, F26C11.4, F26D10.13, F26D11.2, F26D11.4, F26D2.16, F26F12.8, F26F2.8, F26G1.1, F27B10.1, F27E5.7, F28B1.1, F28B1.2, F28B1.9, F28C6.10, F28C6.5, F28E10.5, F28F9.3, F29B9.5, F29C4.2, F29C4.4, F29D10.1, F29D10.2, F30A10.11, F30A10.13, F30A10.14, F30H5.2, F30H5.5, F31A3.1, F31A9.4, F31D4.9, F31E8.5, F31F4.11, F31F6.8, F31F7.3, F32B4.5, F32B5.2, F32D1.11, F32D8.11, F32G8.3, F32H5.3, F33A8.10, F33A8.2, F33A8.7, F33D11.8, F33D4.6, F33E2.7, F33H1.6, F34D6.7, F34D6.8, F34D6.9, F34H10.2, F35A5.2, F35B12.3, F35B12.7, F35C11.1, F35C11.4, F35C5.1, F35C5.10, F35C5.3, F35D11.1, F35D11.9, F35D2.1, F35D2.2, F35F10.5, F35G2.3, F35G2.5, F35H10.2, F35H8.1, F36D1.10, F36D1.12, F36D1.5, F36D1.7, F36D3.14, F36F12.3, F36F12.7, F36F2.7, F36F2.8, F36H1.11, F36H12.1, F36H12.17, F36H12.4, F36H12.5, F36H9.7, F37A8.4, F37C12.10, F37C4.8, F38A6.4, F38B2.2, F38B2.6, F38E11.7, F39C12.4, F39F10.5, F39G3.3, F40A3.1, F40E10.5, F40F4.2, F40F4.3, F40F4.4, F40F4.8, F40F8.4, F40G12.2, F40G9.5, F40G9.6, F40G9.7, F40H3.2, F40H3.3, F41B4.2, F41C3.6, F41C6.3, F41C6.4, F41C6.6, F41D3.7, F41E6.11, F41E6.12, F41E6.15, F41E6.8, F41E7.4, F41E7.5, F41E7.7, F41E7.8, F41F3.3, F41F3.8, F41G3.1, F41G3.16, F42A10.6, F42A10.7, F42A6.2, F42A8.1, F42A9.4, F42F12.1, F42F12.10, F42F12.6, F42F12.7, F42F12.8, F42F12.9, F42G9.4, F43C11.3, F44B9.10, F44D12.6, F44F4.15, F45B8.3, F45D3.3, F45D3.4, F45E12.6, F45E4.5, F45E4.8, F46B6.10, F46B6.13, F46C3.6, F46C5.1, F46C5.10, F46C8.1, F46E10.2, F46F11.3, F46F11.7, F46F2.3, F46F5.15, F46G11.2, F46G11.4, F46G11.6, F47B8.13, F47B8.14, F47E1.1, F47G3.1, F47G9.6, F48B9.3, F48B9.4, F48C1.8, F48C1.9, F48C11.3, F48F7.4, F49C5.11, F49C5.7, F49D11.11, F49E10.2, F49E12.2, F49H12.4, F49H6.12, F49H6.15, F49H6.8, F52A8.3, F52B11.6, F52D1.3, F52D2.5, F52E1.14, F52E4.6, F52G3.4, F53B6.8, F53B7.7, F53E10.3, F53E10.4, F53F1.4, F53F1.5, F53F4.13, F53F4.17, F53F8.7, F53G12.9, F53G2.2, F53G2.3, F53H4.2, F53H4.3, F54B11.10, F54B11.4, F54B3.4, F54B8.1, F54B8.5, F54D12.11, F54D12.9, F54D5.3, F54D5.4, F54E4.3, F54E7.6, F54F11.1, F54F7.2, F54F7.3, F54F7.9, F55A12.6, F55B11.2, F55B11.3, F55B11.4, F55C12.7, F55F3.4, F55F8.8, F55H12.4, F56A8.9, F56C3.8, F56C4.1, F56C4.4, F56C9.8, F56D2.8, F56D3.1, F56D5.6, F56D6.8, F56D6.9, F56F11.1, F56F4.8, F56G4.7, F56H6.6, F56H9.2, F57A8.8, F57C2.4, F57C9.2, F57E7.1, F57E7.2, F57E7.4, F57G8.7, F57H12.3, F57H12.6, F58A4.1, F58B4.3, F58B4.4, F58B4.6, F58B4.7, F58D5.3, F58E10.7, F58E2.2, F58E6.13, F58E6.4, F58E6.7, F58F9.10, F58F9.11, F58F9.6, F58F9.8, F58F9.9, F58G1.4, F58G1.8, F58H1.2, F58H10.1, F58H7.1, F59A1.16, F59A1.6, F59A6.12, F59A7.11, F59A7.2, F59A7.5, F59B1.2, F59B10.2, F59B10.5, F59B2.12, F59C6.16, F59D6.1, F59E11.5, F59E11.7, F59F5.4, H01G02.1, H01M10.2, H01M10.3, H02F09.3, H05L03.3, H06I04.7, H12D21.1, H12D21.12, H12D21.13, H12D21.14, H12D21.15, H12D21.2, H12D21.3, H12D21.6, H12I13.6, H14A12.6, H14A12.7, H14N18.2, H17B01.5, H22K11.3, H23N18.5, H29C22.1, H31B20.2, H31G24.1, H32K16.2, H32K21.1, H34I24.1, H34I24.2, H34I24.3, H35N09.1, H37A05.4, H39E23.2, H43E16.1, K01A12.4, K01A6.4, K01A6.5, K01A6.7, K01A6.8, K01D12.10, K01D12.11, K01D12.12, K01D12.14, K01D12.5, K01D12.8, K01D12.9, K02A2.5, K02A6.1, K02A6.4, K02B12.6, K02B12.9, K02B7.3, K02C4.2, K02D3.1, K02D7.4, K02E11.10, K02E11.3, K02E11.4, K02E11.5, K02E11.6, K02E11.7, K02E2.4, K02F2.5, K02F3.3, K02F3.9, K02G10.4, K03B4.4, K03B4.7, K03B8.11, K03B8.14, K03D3.2, K03D3.5, K04C2.5, K04F1.8, K04F1.9, K04H8.3, K06A4.7, K06A9.1, K06B9.1, K06G5.1, K07A1.4, K07C11.3, K07C11.8, K07C5.9, K07D4.6, K07D4.9, K07G6.1, K07H8.11, K07H8.7, K08B4.2, K08B4.7, K08C9.1, K08C9.10, K08C9.2, K08C9.6, K08D12.4, K08D12.7, K08F4.5, K08H2.10, K08H2.9, K09A9.8, K09B11.10, K09C6.3, K09C6.9, K09C8.6, K09C8.7, K09C8.9, K09D9.9, K09E4.6, K09H11.4, K09H11.9, K10C2.8, K10C3.4, K10C9.1, K10C9.9, K10D3.6, K10G6.2, K10H10.10, K10H10.4, K11D12.1, K11H12.10, K11H12.6, K11H12.7, K11H3.5, K12B6.9, K12H6.10, K12H6.9, M01B2.8, M01D7.5, M01E11.4, M01G12.10, M02D8.6, M02E1.2, M02H5.8, M03B6.3, M03D4.3, M03F8.6, M04B2.6, M04D8.1, M04D8.2, M04D8.3, M05B5.7, M05D6.9, M110.8, M110.9, M116.2, M153.5, M176.10, M176.5, M199.9, M6.4, M70.2, M70.4, M79.4, R01E6.7, R01H10.4, R02D5.10, R02D5.7, R02D5.9, R03A10.2, R03C1.1, R04B3.3, R04D3.2, R04D3.3, R05A10.1, R05A10.2, R05A10.3, R05A10.5, R05D3.9, R05D7.7, R05G9R.1, R05H5.7, R06C7.4, R06F6.11, R06F6.7, R07B1.11, R07B1.8, R07E3.6, R07E3.7, R08B4.5, R08C7.1, R09A1.5, R09B5.12, R09E10.13, R09E10.8, R09F10.8, R09F10.9, R102.11, R11.2, R11.3, R11A5.6, R11D1.12, R11D1.13, R11D1.3, R11D1.4, R11G10.4, R12C12.10, R12C12.8, R12E2.14, R12E2.15, R12E2.6, R12E2.7, R12E2.8, R13A1.1, R13A1.5, R13D11.10, R13F6.2, R13H4.8, R148.3, R155.5, R160.5, R173.4, R52.4, R53.8, T01B10.2, T01B6.4, T01B7.8, T01B7.9, T01D1.3, T01D3.1, T01E8.8, T02B11.3, T02B11.4, T02B11.9, T02C12.5, T02D1.7, T02D1.9, T02E1.8, T02E9.5, T02E9.6, T02H6.10, T02H6.3, T02H6.6, T02H6.8, T03D8.7, T03F6.10, T03G11.10, T04B2.8, T04B8.1, T04C12.3, T04F3.4, T04F3.5, T04F8.8, T04G9.7, T05A10.6, T05A7.7, T05A8.2, T05A8.6, T05C1.1, T05D4.4, T05E12.8, T05G5.4, T05H10.3, T06A1.7, T06C10.4, T06E4.10, T06E4.12, T06E4.14, T06E4.8, T06G6.12, T06G6.6, T07A9.15, T07C12.10, T07C12.13, T07C12.15, T07D10.1, T07D10.6, T07E3.2, T07H3.7, T08B2.12, T08B6.9, T08G3.11, T08G3.6, T08G5.12, T08H10.3, T09A5.14, T09D3.3, T10B10.6, T10B9.9, T10D4.15, T10G3.1, T11F8.5, T11F9.13, T11F9.22, T12A7.5, T12D8.5, T12D8.9, T13A10.1, T13A10.5, T13C2.2, T13C2.3, T13C5.3, T13C5.7, T13F2.4, T13F3.6, T13F3.8, T14A8.2, T16A1.3, T16A1.4, T16G1.2, T16H12.9, T17A3.12, T17H7.7, T19A5.3, T19B10.12, T19B10.2, T19B10.9, T19C3.2, T19C4.7, T19D12.1, T19D7.3, T19H12.12, T19H12.3, T19H5.7, T20B12.5, T20B3.14, T20B6.3, T20D4.16, T20F10.2, T21C12.4, T21C9.9, T21D12.14, T21E3.2, T21G5.6, T22A3.3, T22B11.1, T22C1.12, T22C8.6, T22D1.2, T22E5.1, T22F7.4, T22H6.5, T22H6.7, T23B12.11, T23B3.5, T23B7.3, T23E7.4, T23F6.1, T23F6.2, T23F6.5, T23H2.4, T24D8.3, T24D8.4, T24D8.5, T24D8.6, T24F1.5, T25B6.3, T25B9.11, T25B9.3, T25D10.1, T25E12.14, T25E4.1, T25G12.11, T26A8.3, T26C11.9, T26C5.4, T26C5.5, T26E3.10, T26E3.6, T26E4.10, T26E4.2, T26E4.3, T26E4.5, T26E4.9, T27E4.4, T27E7.1, T28A11.4, T28C6.1, T28D6.3, T28D6.5, T28D9.12, T28H10.4, VC5.3, VK10D6R.1, VZK822L.2, W01A8.7, W01A8.8, W01B11.5, W01F3.2, W02B12.13, W02B3.7, W02D7.10, W02D7.12, W02D9.7, W03C9.1, W03D2.1, W03D2.5, W03D2.9, W03D8.1, W03D8.11, W03F11.5, W03F9.11, W04A4.2, W04A4.3, W04C9.2, W04G3.10, W04G3.12, W04G3.13, W04G3.2, W04G3.3, W04G3.8, W05B10.3, W05E7.3, W05F2.7, W06A11.1, W06A7.5, W07E11.3, W07E11.4, W07G1.7, W07G4.7, W08A12.2, W08A12.3, W08E12.2, W08E12.3, W08E12.4, W08E12.5, W08E12.6, W08F4.5, W08F4.7, W09C2.10, W09C2.7, W09C2.8, W09C2.9, W09C3.2, W09C3.7, W09C3.8, W09C5.11, W09D6.4, W09G12.9, W10G11.19, Y102A11A.5, Y102A5C.27, Y102A5C.40, Y102E9.3, Y102E9.5, Y102E9.6, Y105C5A.17, Y105C5A.8, Y105C5B.18, Y106G6D.6, Y106G6D.8, Y106G6H.1, Y110A2AL.4, Y110A2AL.6, Y110A2AL.7, Y111B2A.2, Y113G7A.15, Y116A8B.1, Y116A8C.29, Y116A8C.3, Y116A8C.33, Y116A8C.43, Y116A8C.44, Y116F11A.3, Y116F11B.13, Y116F11B.8, Y119C1B.6, Y11D7A.5, Y12A6A.1, Y12A6A.2, Y17D7C.2, Y17G9A.2, Y17G9A.3, Y18H1A.8, Y19D10B.3, Y19D10B.4, Y1A5A.2, Y22D7AL.13, Y22D7AL.6, Y22D7AR.10, Y22F5A.4, Y22F5A.5, Y23B4A.2, Y23H5B.3, Y23H5B.8, Y26G10.5, Y26G10.6, Y32G9A.12, Y32G9A.2, Y32G9A.5, Y34B4A.10, Y37A1B.7, Y37D8A.19, Y37D8A.3, Y37E11B.7, Y37E3.19, Y37F4.3, Y37H2A.13, Y38C1AB.1, Y38C1AB.5, Y38E10A.1, Y38H6A.1, Y38H6A.2, Y38H6A.5, Y38H6C.15, Y38H6C.22, Y38H6C.23, Y38H8A.1, Y39A1A.21, Y39A1A.24, Y39A1A.7, Y39A1C.1, Y39A3A.5, Y39A3B.1, Y39B6A.5, Y39B6A.9, Y39E4B.13, Y39F10A.1, Y39F10C.2, Y39G10AL.1, Y39H10A.1, Y40C5A.3, Y41C4A.1, Y41C4A.22, Y41C4A.6, Y41C4A.8, Y41D4A.7, Y41D4B.26, Y41D4B.6, Y41E3.22, Y41G9A.10, Y41G9A.5, Y42A5A.3, Y42G9A.2, Y42H9AR.5, Y43B11AR.1, Y43C5A.4, Y43F8A.1, Y43F8A.2, Y43F8B.18, Y43F8C.1, Y43F8C.15, Y43F8C.16, Y43F8C.17, Y43F8C.2, Y43F8C.20, Y43F8C.23, Y43F8C.5, Y43F8C.7, Y43F8C.9, Y45F10A.5, Y45F10D.14, Y45F10D.6, Y45F3A.4, Y45G5AM.6, Y46E12A.1, Y46E12A.5, Y46G5A.14, Y46G5A.23, Y46G5A.34, Y46H3C.7, Y46H3D.1, Y47A7.2, Y47D3B.12, Y47D3B.2, Y47D3B.3, Y47D7A.10, Y47D7A.11, Y47D7A.12, Y47D7A.2, Y47D7A.6, Y47D7A.7, Y47D7A.9, Y47G6A.15, Y47G6A.29, Y47G6A.33, Y48A6B.4, Y48B6A.9, Y48D7A.1, Y48G10A.6, Y48G1BR.1, Y48G1C.13, Y48G8AL.12, Y49E10.10, Y49F6B.12, Y49F6B.13, Y49F6B.6, Y49F6C.6, Y49G5B.1, Y4C6A.4, Y4C6B.7, Y50D7A.13, Y50D7A.5, Y50E8A.1, Y50E8A.10, Y50E8A.5, Y51A2A.12, Y51A2A.4, Y51A2A.6, Y51A2D.14, Y51F10.7, Y51H4A.1, Y51H4A.10, Y51H4A.22, Y51H4A.32, Y51H4A.8, Y51H4A.935, Y51H7C.1, Y51H7C.13, Y51H7C.15, Y52B11A.8, Y52B11B.1, Y53C12B.7, Y53F4B.27, Y53G8AM.6, Y53G8AR.1, Y53H1A.4, Y53H1B.2, Y54F10AM.6, Y54F10BM.12, Y54G11A.1, Y54G2A.10, Y54G2A.15, Y54G2A.28, Y54G2A.32, Y54G2A.44, Y54G2A.57, Y55B1BL.1, Y55F3BR.11, Y57A10A.23, Y57G11B.2, Y57G11B.3, Y57G11B.5, Y57G11C.1130, Y57G11C.18, Y57G11C.38, Y57G11C.40, Y57G11C.41, Y57G11C.52, Y57G11C.8, Y57G7A.1, Y58G8A.5, Y59A8B.19, Y59A8B.20, Y59A8B.26, Y59E9AL.6, Y59E9AR.10, Y59E9AR.9, Y59H11AM.4, Y5H2A.1, Y60A3A.21, Y60A3A.23, Y62E10A.19, Y62E10A.3, Y62F5A.9, Y62H9A.3, Y62H9A.4, Y62H9A.5, Y62H9A.6, Y64G10A.1, Y64G10A.2, Y64H9A.2, Y65A5A.2, Y65B4BL.6, Y65B4BR.2, Y66A7A.9, Y66H1A.5, Y67A10A.11, Y67A10A.2, Y67D8B.5, Y67D8C.7, Y67D8C.8, Y69A2AR.22, Y69F12A.3, Y6E2A.8, Y71F9AM.8, Y71F9B.15, Y71G12B.21, Y71G12B.5, Y71H2B.4, Y73B3B.3, Y73B6A.3, Y73B6BL.35, Y73B6BL.44, Y73C8C.12, Y73F8A.10, Y73F8A.26, Y73F8A.6, Y74C10AR.2, Y74C9A.2, Y75B12B.11, Y75B12B.13, Y75B12B.8, Y75B7AR.1, Y75B8A.11, Y75B8A.44, Y76A2B.2, Y76B12C.8, Y7A5A.9, Y80D3A.10, Y81G3A.4, Y82E9BR.4, Y95B8A.2, ZC15.5, ZC15.8, ZC155.5, ZC204.17, ZC204.6, ZC21.3, ZC21.8, ZC250.1, ZC328.5, ZC334.13, ZC373.2, ZC373.6, ZC412.10, ZC412.3, ZC412.6, ZC412.7, ZC412.8, ZC412.9, ZC434.3, ZK1098.9, ZK112.3, ZK1225.4, ZK1225.5, ZK1290.1, ZK1290.11, ZK1290.8, ZK1307.2, ZK1320.10, ZK1320.2, ZK1320.3, ZK1321.1, ZK154.1, ZK154.4, ZK180.5, ZK180.6, ZK265.7, ZK285.2, ZK287.3, ZK337.5, ZK353.10, ZK353.5, ZK354.3, ZK39.9, ZK470.6, ZK507.4, ZK512.10, ZK512.7, ZK525.1, ZK593.2, ZK622.5, ZK643.8, ZK662.2, ZK662.6, ZK666.11, ZK675.4, ZK688.1, ZK688.4, ZK813.2, ZK813.7, ZK816.1, ZK816.4, ZK829.3, ZK84.1, ZK84.3, ZK849.6, ZK856.4, ZK856.6, ZK856.7, ZK867.3, ZK909.6, ZK973.4

**all TM containing proteins (5458)**

F27C8.1, F07C3.7, F52H2.2, T13A10.10, C55C2.5, T11F9.4, F54D12.3, F28F9.4, Y53H1C.1, C24F3.5, F12B6.1, Y39D8C.1, Y53C10A.9, K11G12.2, K11G12.7, K03F8.2, ZK973.5, T09A5.3, ZC504.2, C40C9.2, R02E12.8, D2092.3, R01E6.4, T05C12.2, F25G6.4, F25G6.3, F53E10.2, F28F8.1, C31H5.3, R06A4.10, F27B3.2, F48E3.7, F59B1.9, F17C8.1, C10F3.3, C44F1.5, T01C2.1, C04A11.4, ZK154.7, T14B1.2, T05H4.13, F45H10.1, C05E11.4, F49E11.3, M195.3, C05E11.5, VF36H2L.1, ZC434.6, C42D8.8, K08D9.3, F32A5.5, C01G6.1, Y69E1A.7, F40F9.9, C35A5.1, C32C4.2, M02F4.8, K02G10.7, K07A1.16, ZK1321.3, ZK525.2, F31A9.3, C15F1.6, T18H9.2, F26D10.9, C38C6.2, B0207.12, R11G10.1, Y71F9AR.1, F56C11.1, K04F10.4, F23H11.5, Y39E4B.9, B0464.4, Y79H2A.1, C23H4.1, R173.1, C01G6.8, W01C8.6, T13F2.8, C56A3.7, C54D2.5, T10B9.10, C15B12.7, ZK112.7, F25F2.2, F08B4.2, R05H10.6, F59C12.1, C45G7.5, B0034.3, F18H3.5, F35E8.11, Y47H9C.4, C48B4.4, F08F1.5, T07C4.8, ZK512.3, C18C4.2, F27E11.3, ZC416.8, F56H1.1, T25G3.2, F48A11.1, C48D1.3, C09F12.1, C01C10.1, ZK563.4, T05A10.2, C01C10.4, T27D12.2, B0491.8, E04F6.11, T06F4.2, C07H4.2, R07B7.1, F07B10.1, C01G8.2, ZC190.1, R09B5.2, R09B5.3, R09B5.8, R09B5.9, R09B5.10, F14H8.6, F38E11.12, ZK632.6, C48E7.5, F54B11.1, T13B5.4, Y18H1A.12, W07A12.5, F38A3.1, F33A8.9, Y11D7A.11, F59F3.2, C34F6.2, C34F6.3, F57B9.4, F11C7.4, Y73B6BL.7, MTCE.21, Y23H5A.5, Y76A2A.2, F25D7.1, C02C2.3, R13A5.1, C47G2.1, M142.2, C54G4.8, F57B10.10, F29C4.1, Y55D5A.5, C05D2.1, F31F6.5, T13C5.1, B0240.3, T23G5.5, C47C12.6, K03B8.9, E02H4.1, T26H10.1, T21B6.1, F55A12.4, F56D1.5, C10F3.2, T11F9.11, C15H11.4, ZK816.5, C04F6.5, F27D9.6, F49E8.5, B0035.2, C01G10.12, C01G8.4, C04A2.7, C56C10.13, K02G10.8, T04A8.9, Y39C12A.8, Y63D3A.6, F15A8.5, K09G1.4, T23F1.7, M01E10.2, F46E10.9, F22B7.10, C43C3.3, Y48B6A.4, ZK512.6, F13G3.8, B0365.3, Y105E8A.7, H30A04.1, C26D10.5, F16B3.1, C46F4.1, F58A3.2, C48A7.1, Y37A1B.11, R07A4.1, C50H2.2, F56H11.4, F11E6.5, D2024.3, C40H1.4, F41H10.7, F41H10.8, F56H11.3, Y47D3A.30, Y53F4B.2, F32D8.6, M01D7.6, ZK809.4, F09B9.3, H35N03.1, F12F3.1, F15B9.2, Y67H2A.8, W02A2.1, W08D2.4, T13F2.1, W06D12.3, VZK822L.1, F10D2.9, C04F12.10, F48F5.5, F43G9.6, F41G3.4, F13B9.8, F02D10.5, F09B12.6, F15B9.7, K08C7.2, K08C7.5, F53F4.5, C24A11.8, F31B12.3, EGAP9.2, ZK1248.14, ZC482.1, C15B12.5, F47D12.1, Y40H4A.1, AH6.1, R134.2, R134.1, ZK970.5, ZK970.6, B0024.6, F52E1.4, C49H3.1, ZK455.2, F08B1.2, F23H12.6, ZC412.2, ZC239.7, W03F11.2, ZK896.8, C17F4.6, F21H7.9, F22E5.3, T03D8.5, T26C12.4, Y105C5B.2, C06A12.4, Y37A1B.15, C09G5.1, C45B2.4, F09C12.1, H04J21.3, F11A5.10, F25F8.2, ZC317.3, C27H5.8, C06E1.4, B0280.12, K10D3.1, C06A8.9, ZC196.7, F41B4.4, C43H6.9, F22A3.3, C12D12.2, K08F4.4, T22E5.2, Y53C12A.2, R05G6.6, W03G1.1, F44F4.6, Y46H3A.6, Y45F10D.3, T15D6.2, T01H8.5, C05C12.3, F54D1.5, C14F11.3, ZC504.5, K09E4.5, ZK622.2, C30H6.6, F43E2.4, F57A10.3, W04C9.1, W09D6.6, Y48G8AL.11, Y50E8A.16, ZK484.2, W02B9.1, C18E3.8, C13G3.2, T10H10.1, C09G4.1, K02G10.6, B0244.2, K07A1.8, T04G9.3, F54G8.3, C16E9.4, F08G12.10, F22F4.2, F26D11.10, R09F10.4, C36H8.2, K02B2.4, ZK792.2, ZK792.3, T18H9.5, W04D2.3, ZK770.3, Y8G1A.2, F07A5.1, R12E2.9, R12E2.5, R12E2.4, C18H7.2, T16H5.1, T23H4.1, Y47G6A.1, Y47G6A.2, R03E9.4, M02A10.2, K04G11.5, F33D4.2, W03D8.6, T22C1.7, Y77E11A.4, F08F1.1, M176.6, M176.7, F20C5.2, C25B8.1, M60.5, Y54G9A.3, C53C9.3, Y54G2A.25, Y73C8B.4, B0457.1, B0286.2, W01G7.5, ZK1067.1, C56G2.6, H19M22.2, F09E8.7, C35C5.5, F36H1.4, R107.8, Y71F9B.5, C16B8.1, C03B1.12, ZK945.9, K09H9.6, T21C9.5, F29D11.1, T21E3.3, W09C2.3, Y67D8C.10, F14D12.4, T01C8.7, F16F9.5, C52B9.9, F54F7.5, T07C4.7, ZC506.4, F45H11.4, F13G3.9, Y34D9B.1, F43C9.4, R06B9.6, K06C4.6, Y54E10BR.7, T07H6.2, T23D8.1, C29F5.4, C52A11.4, F57C12.5, F57C12.4, E03G2.2, F21G4.2, F14F4.3, F20B6.3, Y43F8C.12, Y75B8A.26, K08E5.3, K07D8.1, F31F6.6, R107.1, K08E5.2, K03B8.2, K03B8.5, C11D2.6, C27F2.2, F02E8.6, F09G8.4, Y113G7A.4, C10G8.5, ZC168.1, F35C12.2, Y32F6B.2, C07A9.4, C07A9.11, C13D9.7, C13D9.8, Y97E10B.7, F56F3.2, Y87G2A.14, F48G7.3, B0395.1, B0495.4, C54F6.13, F14B8.1, F57C7.2, F58E1.6, Y18D10A.6, ZK822.3, B0213.2, F20B10.1, F07F6.6, T01C3.10, C15H9.1, T23H2.1, C39E6.6, T05A1.1, C08B11.4, T01B11.7, F28H7.10, T09A12.3, T10B10.7, Y40C5A.2, F52F12.1, ZK455.8, R01E6.1, T01C4.2, Y102E9.1, C53B7.5, C08H9.5, ZK938.5, B0334.11, C06G8.2, K04E7.2, F56F4.5, B0212.5, M88.6, F54F2.1, ZK1058.2, K09C8.1, F46C3.1, T28D6.9, F18G5.2, K05B2.5, F14B8.3, ZK381.4, K08E7.9, C34G6.4, ZK455.7, F42E11.1, C05A9.1, T21E8.1, T21E8.2, T21E8.3, C47A10.1, C54D1.1, DH11.3, F22E10.1, F22E10.2, F22E10.3, T24H7.1, EGAP2.3, E01H11.1, Y73F8A.1, K10F12.3, Y55F3AL.1, K04B12.1, C44B7.8, C44B7.9, C54G10.3, T02D1.5, T10H9.5, ZK256.1, T05G5.11, C18E9.3, C24A8.3, D1044.3, F35D11.2, F59B10.1, T16A1.7, W01C9.3, Y39E4B.3, Y73B6BR.1, F08B12.2, M03F8.2, ZK675.1, F21H12.4, Y110A2AL.8, F07C3.1, C24B5.3, C32E8.8, C41D7.2, C45B2.7, C53C11.3, C54A12.1, F44F4.4, F54G8.5, F55F8.1, F56C11.2, K07A3.2, K07C10.1, R09H10.4, T07H8.6, T21H3.2, Y18D10A.7, Y38F1A.3, Y39A1B.2, Y53F4B.28, Y65B4BR.3, Y80D3A.7, ZK270.1, F46G10.5, T24C2.1, F53F4.6, W06A7.3, F08F3.3, B0240.1, T14A8.1, K09E2.4, Y42H9B.2, T11F8.3, C16C10.7, C03C10.3, C16D9.2, F26F4.3, C48B4.2, Y116A8C.14, Y116A8C.16, Y54E10A.14, ZK377.2, C18F3.2, Y47D3B.7, K11D9.2, T10H9.2, M01D7.2, D2013.8, F57C7.3, F45D3.5, W02D7.7, F55A11.3, F35H12.3, F59C12.2, C02D4.2, K02F2.6, Y22D7AR.13, C09B7.1, H06I04.2, C54H2.5, F07H5.2, R186.5, C04F5.1, ZK520.2, Y51A2D.19, F08B12.3, C32D5.2, K11G12.4, K11G12.3, Y69A2AR.4, Y54E5B.1, D1037.2, H21P03.3, F53H8.4, Y22D7AL.8, T10H9.4, F23H12.1, C30A5.5, W03G9.1, F55H12.1, T13B5.1, Y46G5A.30, M01G5.5, ZK1010.9, ZK829.10, C49C3.1, Y32F6A.2, T03F7.1, T25B6.7, T08A9.3, F31E8.2, T23H2.2, H34C03.1, ZK524.1, C17D12.6, AC3.10, T02E1.1, F25H8.7, K01A11.4, F42G8.11, Y66H1B.4, C52E12.3, AH6.4, AH6.6, AH6.7, AH6.8, AH6.10, AH6.11, AH6.12, AH6.14, F44F4.5, F44F4.13, F44F4.7, F49E12.5, F35C5.2, F28C12.1, F28C12.2, F28C12.4, F28C12.5, F28C12.7, T06G6.1, T06G6.2, T26E3.9, T19D12.8, F18C5.1, F18C5.6, F18C5.8, Y40H7A.6, C56C10.5, B0304.5, B0304.6, B0304.7, B0304.8, B0304.9, T21H8.2, T21H8.3, T21H8.4, C27D6.10, C27D6.9, C27D6.8, C27D6.6, R05H5.6, F37C12.17, F37C12.15, F37C12.16, F23F12.10, F33H1.5, R05H5.1, K10B4.5, ZK863.5, T19E7.5, C06G8.4, B0547.4, T09D3.1, Y45F10B.14, F53F4.9, C39H7.6, C39H7.7, C04E6.10, C04E6.9, Y2H9A.2, F53F1.10, F53F1.11, Y38A10A.1, W06G6.3, Y40H7A.5, F07C4.4, T02B5.4, T26E4.12, M01B2.2, F07C4.3, F07C4.5, F07C4.8, T19H12.5, T19H12.4, F32G8.1, R186.2, R04D3.6, R04D3.12, R04D3.8, F17A2.12, F17A2.6, R04D3.7, R04D3.9, F17A2.8, F17A2.7, F17A2.10, F17A2.11, F17A2.9, R04B5.8, F15A2.4, F15A2.3, F13G3.2, K02A2.2, E04F6.14, E04F6.13, E04F6.1, C13B7.3, C01B4.5, F13A7.2, Y22D7AR.8, C39B5.12, Y39A3B.4, C39B5.11, F09C6.7, H04J21.2, F48F5.4, C13B7.4, B0495.1, C41C4.2, M02B1.1, C18F10.4, C18F10.5, C18F10.6, T12A2.12, T12A2.11, T12A2.13, C18F10.8, T12A2.9, T12A2.10, T04A8.1, T04A8.2, T23F11.5, F26B1.6, C34C6.1, F15A4.4, F15A4.7, Y105C5A.11, ZC204.4, Y25C1A.9, Y25C1A.10, Y25C1A.11, Y25C1A.12, T09D3.5, C10G8.1, T09D3.7, T09D3.2, T09D3.6, W02F12.7, T07H8.5, T21C9.7, F21F8.1, Y51A2D.12, K04C1.6, K04C1.1, T19C4.8, T19C4.2, T19C4.4, T19C4.3, T19C4.9, F31E9.2, C51F7.2, F32H5.5, C53A5.8, Y43B11AR.5, Y43B11AR.2, Y40H7A.8, T02B11.1, W09D12.3, W09D12.2, C24B9.10, C24B9.14, R05D8.6, C24B9.7, C24B9.15, C24B9.11, C24B9.12, ZK678.6, T05C3.8, T05C3.7, ZC317.4, ZC317.5, F09E5.4, T11F9.18, C05E4.14, K04F1.5, K09D9.6, K04F1.16, K09D9.7, K09D9.8, Y38A10A.3, R09F10.6, C50B6.6, F55C5.9, C47E8.2, C50B6.5, F19G12.5, C02E7.3, C02E7.2, C02E7.4, C02E7.5, C54D10.6, W05H5.4, ZC404.5, ZC404.12, W06D12.7, Y26G10.2, M02H5.9, M02H5.10, M02H5.11, T03D3.4, R11G11.9, C31B8.11, C06A8.7, Y40D12A.3, Y54G11A.12, Y54G11A.15, B0334.7, T03F7.2, T03F7.3, T03F7.4, C06C3.6, C10G11.4, C10G11.2, C10G11.3, R08H2.7, T09F5.5, R05D8.3, Y49F6A.2, W10G11.10, W10G11.9, T21B4.8, ZK6.9, T27E7.8, T21B4.6, T21B4.5, T21B4.7, T21B4.9, T10D4.5, ZC204.5, T21B4.14, C45B11.4, T04C12.2, F26F12.6, DC2.6, DC2.2, C04F2.1, DC2.1, W03F9.6, F09G2.7, H27D07.6, C44C3.9, F25E5.14, F25E5.13, F21H7.11, F49H6.4, F21H7.14, C46E10.7, C46E10.10, F40D4.11, F21H7.7, T27A1.1, T19C9.4, F08E10.6, T19C9.2, Y68A4A.7, Y61B8A.2, Y61B8A.1, K03D7.6, Y102A5C.21, T27C5.1, R08H2.5, F08E10.3, C18B10.8, F37B4.1, Y47G7B.1, F14F9.7, F14F9.1, Y102A5C.31, T27C5.5, Y59A8B.3, Y59A8B.4, T01E8.7, F36D3.6, Y70C5C.4, T08G3.12, F57E7.3, Y6E2A.6, T08G3.5, T08G3.3, F26D2.4, K08G2.12, K08G2.5, K08G2.13, F20E11.12, F40D4.3, C41G6.9, Y38H6C.12, F57G8.3, C49G7.2, R08H2.3, Y60A3A.5, Y60A3A.6, Y60A3A.4, F40D4.1, K02E2.3, ZK228.8, ZK228.7, F57G8.1, ZK228.6, ZK228.5, Y60A3A.3, D1054.12, C50H11.7, F36G9.2, F10G2.8, C17E7.2, ZC132.7, F47D2.9, F47D2.10, R52.7, ZK697.11, F07C4.13, R03H4.9, F20E11.10, E03D2.3, Y102A5C.15, ZK262.1, C43D7.6, ZK262.11, D1065.4, D1065.5, T22F3.6, T22F3.5, T20B3.4, T20B3.3, T20B3.5, Y113G7B.2, C06B8.10, C06B8.6, F47C12.5, F47C12.10, F47C12.3, C35D6.2, C35D6.1, C04F2.4, C03G6.11, Y113G7A.1, F07C4.14, F08E10.1, Y68A4A.9, T26H5.3, F37B4.13, F37B4.6, F37B4.3, C03G6.9, F31F4.9, F31F4.18, C31B8.13, H05B21.2, C49D10.3, R08H2.4, T19C9.3, F28B1.8, T06E6.6, T06E6.7, K03D7.4, T06E6.8, T06E6.9, F31F4.6, C54F6.1, T03E6.5, F37B4.5, W02H5.6, F37B4.4, C32B5.2, C03G6.7, F21A3.1, K05D4.6, T09F5.8, F11A5.2, F11A5.1, T21D12.5, C47A10.2, C47A10.3, Y59A8A.4, C47A10.9, C47A10.7, C47A10.10, C47A10.11, Y94A7B.3, Y94A7B.1, K08G2.8, ZK1037.8, Y94A7B.4, C17F4.4, Y94A7B.5, K08G2.9, Y94A7B.6, Y94A7B.8, Y94A7B.7, Y94A7B.9, Y49F6A.3, T27A1.7, F36G9.8, F36G9.9, C05E4.4, F28D9.2, T09E8.5, T06E6.4, Y102A5C.29, C54E10.4, T22H2.3, T22H2.1, M01G12.13, M01G12.1, F15H9.2, F15H9.4, F15H9.3, Y22F5A.2, Y69E1A.6, AC3.1, T24A6.4, C41G6.11, C41G6.10, C41G6.14, ZK697.4, B0454.3, B0454.4, B0454.10, B0454.2, T10D4.12, Y27F2A.4, Y27F2A.7, F33H12.5, D2062.2, D2062.8, F33H12.2, Y27F2A.3, T10D4.9, T10D4.10, K07E8.9, K07E8.11, F22E5.4, ZC239.9, ZC239.19, ZC239.8, ZC239.10, T10D4.8, F22E5.16, Y47G7B.3, F34D6.6, F34D6.5, F39E9.3, F13A7.8, F28B1.6, Y60A3A.22, Y102A5C.32, Y61B8B.1, D2062.3, C14C6.10, F33H12.4, D2062.9, Y27F2A.2, ZK829.8, C27A7.7, F31F4.16, F31F4.8, T28A11.10, T28A11.9, T20C4.1, F14H3.1, T28A11.12, R13D7.3, C03G6.3, Y40B10B.2, T06A1.2, R05D8.2, Y45G12C.14, Y45G12C.15, F28H7.11, F28H7.1, R09B5.7, C05E4.11, C05E4.10, ZK262.10, T01G5.3, F22B8.1, F07G11.5, T07C12.1, F48G7.1, T02B11.5, F38H12.1, F38H12.2, Y73F8A.3, T03D3.11, T03D3.6, T03D3.12, T03D3.2, Y49C4A.6, F37B4.11, Y45G12A.1, T03D3.14, T28H11.2, T28H11.3, K12B6.5, D1022.6, W07G4.6, T26H2.8, F36D3.3, K11D12.3, C13D9.2, C13D9.1, T01G5.4, C13D9.3, T05B11.2, T05B11.6, C33A12.14, C33A12.13, C33A12.11, C33A12.10, C33A12.8, C28C12.13, R07B5.7, Y45F10B.11, Y45F10B.6, Y45F10B.5, F53B2.4, T02D1.3, Y45F10B.4, T04A11.10, T04A11.9, T04A11.8, T04A11.7, C08F11.4, F36G9.5, F36G9.6, F31F4.13, F31F4.14, T04A11.12, C38C3.2, C38C3.1, C50C10.2, C50C10.3, C50C10.4, C50C10.8, C33D9.4, R07B5.6, C50C10.1, R07B5.4, F46B6.11, R07B5.3, C55A1.8, T08G3.8, Y32B12B.7, F36D1.3, T03G11.2, R13F6.3, C14A4.15, Y43F8C.19, F15E6.7, F48D6.2, C52B9.5, T22B7.5, F53F1.7, F53F1.8, F53F1.9, H25K10.3, T04B2.4, Y73B6BL.39, Y105C5B.4, Y105C5B.6, Y105C5B.10, C06E7.7, H04M03.6, H04M03.8, H04M03.9, Y73B6BL.10, Y73B6BL.11, Y73B6BL.40, Y73B6BL.41, T13A10.14, T13A10.6, T13A10.7, T13A10.9, T13A10.12, T13A10.13, F13A7.3, F57G8.8, Y75B12B.7, T03D3.3, ZK105.2, F37B4.8, H10D18.3, R08F11.5, F59D6.5, ZC482.8, ZC482.6, M01B2.11, C41G6.2, C41G6.15, C41G6.8, C41G6.3, T10H4.5, T10H4.6, T10H4.8, T05E12.4, C06B8.9, T10H4.3, F49A5.8, C41G6.7, T05G11.3, F26D2.11, K10G4.9, K03D7.2, K03D7.11, T26H5.5, F36D3.13, T08G3.10, F14F8.7, Y116F11B.5, F19B2.3, Y52E8A.5, F14F8.10, F14F8.11, F14F8.5, F14F8.6, K10G4.2, F26D2.9, F40D4.8, T05G11.7, Y32B12C.2, T05G11.6, F36G9.16, F36G9.1, H24D24.2, H24D24.1, T11F1.1, H25K10.7, Y37A1B.10, Y38H6C.2, Y57G7A.4, F18E3.2, F18E3.4, F18E3.5, F18E3.6, F20E11.6, T03E6.6, W06G6.13, W06G6.6, W06G6.8, T11F1.5, ZK262.7, ZK262.6, Y43F8A.4, C25F9.1, F57G8.4, T06G6.7, K09D9.13, Y46H3C.3, K04F1.4, H06H21.1, H06H21.2, K04F1.2, ZC204.15, K04F1.3, Y46H3C.2, Y46H3C.1, ZK697.13, ZK697.12, ZK697.5, R11G11.13, R11G11.5, ZK488.9, M01G12.4, K12D9.9, ZK1037.9, K12D9.4, C44C3.6, C44C3.5, K12D9.7, K12D9.5, K12D9.3, H27D07.5, C44C3.11, C44C3.1, C33G8.1, C44C3.2, T24A6.14, T05B4.5, T05B4.6, T05B4.7, K12D9.10, H27D07.4, C44C3.3, C44C3.7, C03A7.3, H27D07.2, H05B21.3, H27D07.3, H05B21.4, F59E11.1, R03H4.2, R03H4.3, Y49C4A.5, Y49C4A.4, F07G11.8, C14C6.1, C14C6.9, R13D7.6, R10D12.4, Y55F3AM.2, C44B12.4, C44B12.8, T07H8.1, F55B12.8, C47A10.8, T05A12.1, R13H7.1, F31F4.4, F31F4.3, F31F4.2, C24B9.16, R05D8.4, R05D8.5, K06B4.9, F41H8.4, R13D11.9, T01C4.3, T01C4.4, T01C4.5, T01C4.6, C03A7.5, C03A7.6, T06E6.3, F59B1.7, C06B3.11, T10C6.3, T10C6.4, K01B6.2, E02C12.2, E02C12.3, T26H8.2, Y43B11AL.2, T10H4.9, T27C5.2, Y102A5C.22, Y102A5C.24, C03G6.2, C29F3.6, T07H8.3, R13D7.4, K09D9.10, K07C6.6, K07C6.7, K07C6.8, K07C6.9, K07C6.10, K07C6.11, F41F3.7, F41F3.6, K12G11.5, K03B4.5, C01G10.3, C01G10.2, F43A11.4, F43A11.6, F35B12.1, F38B7.4, F38B7.7, F38B7.8, F43A11.1, F43A11.3, H12C20.5, F10G2.6, F59B1.3, F41G3.11, T01D3.4, F19B10.7, F19B10.8, F40H7.4, F40H7.5, F40H7.7, F40H7.8, F40H7.2, C46E10.6, T24E12.4, T24E12.8, F56H9.1, F07F6.9, B0507.11, W05B10.5, C14C11.5, T21B4.12, F49C5.1, F49C5.2, C04E12.8, F35F10.8, F32H5.6, F55B12.7, F55B12.9, F09F3.1, F09F3.2, F09F3.4, F09F3.7, F09F3.11, F09F3.12, F09F3.13, F54C1.9, F08C6.4, F32A6.5, F52D10.5, Y71H9A.3, F41G4.3, Y71H9A.2, C42D4.5, C50C10.7, M7.13, C50B6.10, T23D5.12, T23D5.10, F22B8.5, T23D5.11, F57A10.1, R03G8.5, B0391.4, C24B9.8, T08G3.2, T08G3.1, Y73C8A.1, T23D5.6, T23D5.7, C05E4.2, C55A1.5, F44G3.11, T23D5.1, F25E5.12, T27C4.3, C54F6.10, T19H12.7, C24B9.1, C50B6.12, T23D5.2, F37B4.12, C50H11.12, R13D7.1, T23D5.9, C42D4.4, T09F5.3, C31B8.6, F07C4.1, C34D4.8, C45H4.12, F26D2.7, T06C12.3, T06C12.2, T06C12.1, F14F8.1, C17B7.1, T28A11.1, Y73C8C.5, K10C9.6, Y73C8C.6, Y73C8C.11, T23F1.4, F26D2.1, C14H10.4, C01B4.10, W06D12.4, Y40H7A.1, F52D2.9, T26H2.6, B0213.8, B0213.7, F59E11.13, F59E11.15, F59E11.14, F59E11.16, R08H2.2, F59A1.4, F58G4.2, F59A1.3, F59A1.14, F07C3.8, T10H4.2, F10A3.8, F10A3.6, Y6E2A.2, R08H2.13, F44G3.1, K05D4.2, F10A3.13, F10A3.5, F10A3.15, F10D2.4, Y39H10B.1, F59B1.1, F07B10.3, F07B10.2, F57A8.3, C13B7.5, C13B7.2, R02C2.5, W03D2.10, T22H6.4, T22H6.3, T05E12.1, F58G4.5, C09H5.9, C09H5.6, C05E4.6, F21F8.10, F21F8.9, ZC513.9, ZC513.11, C09H5.3, F47G9.2, C09H5.4, C09H5.5, F58G4.7, F58G4.6, M01D1.1, Y32B12B.5, T03E6.4, T03E6.1, Y9C9A.7, Y45G12C.12, Y9C9A.10, Y9C9A.11, Y9C9A.9, Y9C9A.18, ZK697.10, T08B6.3, E03H12.1, Y9C9A.3, Y9C9A.2, F55B12.6, T08B6.6, Y9C9A.6, Y9C9A.4, T08B6.7, T01G5.5, Y17G9A.5, Y17G9A.1, Y17G9A.7, Y17G9A.6, T18H9.4, R11D1.5, T10H9.6, R11D1.6, C12D8.12, T13F3.1, R08C7.7, F26D10.8, F40F9.4, C18B10.7, C50C10.6, T01G6.3, ZK285.1, Y68A4A.3, F20E11.4, F10D2.1, C02E7.9, W06H8.7, F26G5.4, F26G5.2, F26G5.5, C02E7.13, C31A11.9, Y102A5C.28, C42D4.9, Y46H3D.2, Y46H3D.3, F49C5.6, K10C9.8, C07G3.6, C07G3.4, C07G3.3, C07G3.5, C17E7.11, T16A9.2, C06C6.3, T10C6.1, C06C6.2, K02H11.3, R09E12.4, K02H11.2, K02H11.7, C50H11.9, F32A7.7, W09D6.3, B0213.9, C55A1.3, C06B3.10, C06B3.9, F41B5.8, F10A3.9, C45H4.15, T01G6.9, C01B4.1, C01B4.3, T24A6.6, W09D6.2, C05E4.13, B0391.12, F34D6.3, R09G11.1, DY3.7, F01G12.2, K02E10.8, C44H4.2, F35C8.4, F48F7.2, F55A11.2, T01B11.3, F14D12.6, C40C9.5, R02C2.3, AC7.1, F33C8.1, T27E9.1, H02I12.3, F40F9.2, F08F1.7, M03F4.3, ZK721.1, B0563.4, T11F9.2, C30H6.2, F56D6.6, F36H1.2, H13N06.5, F31D5.3, R13F6.4, R13A1.2, Y6B3B.10, Y76A2A.1, F54D8.2, D2013.10, C56E6.1, F36F2.5, ZC84.2, H13N06.6, C38C10.1, ZC395.3, C07F11.1, Y37A1B.13, C15F1.3, F15A2.2, ZC21.2, R06B10.4, Y71A12B.4, C02F5.8, C02F5.11, Y39E4B.4, F53B2.2, Y45F10B.1, T23D8.2, F33C8.3, C25G6.2, T14B4.4, B0563.2, T14G10.6, Y39B6A.6, F39C12.3, F53B6.1, F01E11.4, C02F12.1, F59G1.2, D2092.7, B0198.1, F21C3.1, T12C9.3, M110.2, ZK1067.5, B0334.2, F17C8.5, F22B7.7, ZC410.4, ZK1251.8, K04A8.4, F20A1.7, F29F11.4, R04F11.4, K01D12.4, F52E4.4, C44E12.3, C24A3.6, C40C9.1, T01B4.1, T01B4.2, F19D8.1, F55C5.3, M04B2.5, C33D12.3, C52B9.6, F46A9.3, F36A2.4, Y47D3B.5, F53C11.6, W06D12.5, K06B4.12, F31D4.7, R12G8.2, C48E7.9, C24H11.8, T28A8.1, W06D12.2, F32H5.7, Y71H9A.1, Y71H2AM.22, Y39B6A.19, D1022.1, K03E6.5, T02C5.5, B0273.4, R07D5.1, R13A1.4, R12H7.1, F57H12.2, T08G11.5, ZK637.8, F21F3.5, T19B4.7, T20G5.6, T21C12.1, T07A5.2, T06H11.1, Y110A7A.3, F56A8.7, K11C4.5, T07A5.6, Y37D8A.13, W01A11.3, F54B11.3, C30A5.7, C46F11.1, C30D11.1, C41C4.5, F11C3.2, F56B6.5, ZK563.1, M03A1.1, T22C8.8, T17A3.1, F59F3.1, F59F3.5, R10E11.8, R10E11.2, Y38F2AL.4, T01H3.1, F35H10.4, VW02B12L.1, C26H9A.1, Y56A3A.32, Y53C12A.1, C18A11.5, F54F7.4, K10C3.3, ZK546.1, C10C6.2, T24D1.4, T04A8.12, W02D3.6, T26A5.1, cTel55X.1, AC3.2, AC3.5, AC3.7, AC3.8, AH6.2, AH10.2, B0024.3, B0024.13, B0024.14, B0198.2, B0198.3, B0240.2, B0240.4, B0250.6, B0250.9, B0250.10, B0272.2, B0284.3, B0285.6, B0331.2, B0334.5, B0334.6, B0391.3, B0393.5, B0393.8, B0395.2, B0399.1, B0399.2, B0464.3, B0464.6, B0491.1, B0491.4, B0513.9, B0564.3, B0564.4, BE10.2, C01A2.2, C01A2.3, C01F6.2, C01G6.2, C01G10.1, C01G12.7, C01G12.8, C01H6.6, C02C6.2, C03A3.1, C03A3.2, C03E10.1, C03E10.3, C03H12.1, C04A11.1, C04F12.7, C04H5.3, C05A9.2, C05B5.2, C05B5.4, C05B5.8, C05C10.7, C05C12.4, C05C12.6, C05D12.1, C05E7.4, C05G5.1, C05G5.3, C06A1.2, C06B3.1, C06B3.2, C06B8.4, C06B8.7, C06C6.1, C06C6.7, C06G8.1, C06H2.4, C06H5.6, C06H5.7, C07A9.8, C07C7.1, C07H4.1, C08B6.1, C08B6.2, C08B6.5, C08B6.11, C08B6.12, C08B11.8, C08E8.1, C08F8.4, C08F8.9, C08F11.1, C08F11.2, C08F11.3, C08F11.8, C08F11.9, C08F11.13, C08H9.3, C08H9.4, C08H9.7, C08H9.10, C08H9.11, C08H9.12, C08H9.13, C08H9.15, C09F9.1, C09F9.2, C09F12.2, C09F12.3, C09G9.5, C10C5.1, C10C6.5, C10C6.6, C10C6.7, C11E4.3, C11E4.8, C11G10.1, C11H1.2, C12D8.5, C12D8.6, C12D8.13, C12D8.14, C12D8.16, C12D8.17, C13B4.1, C13C4.5, C13C4.6, C14A4.3, C14A4.6, C14A4.7, C14A4.12, C14A6.2, C14A6.5, C14A6.6, C14A6.7, C14A6.8, C14B1.2, C14C10.1, C14C10.6, C14H10.1, C15A7.1, C15A7.2, C15A11.4, C15A11.7, C15C6.1, C15C8.1, C15C8.5, C15C8.6, C15H11.2, C15H11.11, C16C10.1, C16C10.5, C16C10.12, C16D6.2, C16D6.3, C17D12.1, C17D12.3, C17E4.3, C17E4.4, C17E4.9, C17G1.1, C17G1.2, C17G1.3, C18B12.1, C18B12.2, C18B12.4, C18B12.6, C18D1.2, C18D1.4, C18D4.9, C18D11.1, C18D11.2, C18D11.6, C18E9.2, C18E9.5, C18E9.9, C18E9.10, C23H4.2, C23H4.3, C23H4.4, C23H4.7, C24F3.1, C24H11.4, C25A1.5, C25D7.10, C25G4.10, C26C6.9, C26G2.1, C26G2.2, C27A7.1, C27A7.6, C27C12.4, C27C12.5, C27D8.4, C29A12.4, C29E6.2, C29E6.4, C29F4.2, C29F4.3, C30F2.5, C30H6.5, C30H6.9, C31A11.1, C31A11.3, C31A11.4, C31A11.5, C31A11.6, C31A11.7, C31E10.1, C31E10.6, C31E10.7, C31E10.8, C31G12.1, C32A3.3, C32C4.1, C32C4.3, C32H11.2, C33A11.2, C33A12.6, C33D3.5, C33G3.3, C34B4.3, C34B4.5, C34C12.4, C34D1.4, C34F6.7, C34F6.10, C34F6.11, C35A5.2, C35A5.3, C35A5.5, C35A5.7, C35C5.2, C35C5.10, C36A4.5, C36A4.10, C36B1.6, C36B1.12, C36E8.3, C36H8.1, C37A5.1, C37A5.2, C37A5.4, C37E2.2, C37E2.3, C38C6.4, C38C10.2, C38D4.1, C38D9.5, C38H2.3, C39B10.1, C39B10.2, C39E9.7, C39E9.10, C41C4.7, C41G6.12, C41G6.16, C43C3.2, C43F9.4, C43F9.6, C43F9.9, C43F9.10, C44B9.3, C44C10.3, C44C10.9, C44C10.10, C44H4.1, C44H4.8, C44H9.1, C44H9.6, C44H9.7, C44H9.8, C45B11.5, C46C2.2, C46F11.5, C47A4.2, C47A10.4, C47A10.6, C47B2.1, C47D12.3, C47D12.5, C47E8.1, C47E8.3, C47E12.2, C47G2.3, C47G2.4, C48B4.6, C48B4.7, C48B4.8, C48B4.9, C48B4.10, C48B4.11, C49A1.2, C49A1.3, C49A1.10, C49F5.3, C49F5.5, C49F8.2, C49G9.1, C50B6.11, C50B8.4, C50B8.5, C50B8.6, C50F4.14, C50H2.1, C50H2.12, C50H2.13, C51E3.1, C51E3.2, C51E3.3, C51E3.4, C51E3.5, C51E3.6, C53A5.1, C53A5.5, C53A5.10, C53A5.13, C53B4.1, C53B4.3, C53B4.6, C53C7.1, C53D6.3, C53D6.5, C54C6.4, C54D10.4, C54D10.5, C54E10.3, C54G4.4, C54G4.5, C55A1.1, C55A1.12, C55A1.14, C55A1.15, C55A6.11, C56A3.3, C56A3.4, C56G7.2, D1046.3, D1046.4, D1046.5, D1053.4, D2005.6, D2030.12, D2085.6, D2089.2, DY3.8, E01G6.3, E03H4.7, E04D5.2, E04D5.3, F01D4.1, F01D4.2, F01D4.7, F01D5.10, F01G4.5, F01G4.6, F02A9.1, F02C12.1, F02D10.6, F02E9.7, F07A5.3, F07B10.4, F07B10.5, F07B10.6, F07C6.2, F07H5.6, F07H5.7, F08A10.1, F08B12.1, F08E10.2, F08G5.2, F08G5.5, F08G12.5, F09A5.1, F09A5.2, F09B9.1, F09B12.2, F09B12.3, F09E8.2, F10A3.1, F10A3.7, F10A3.12, F10A3.16, F10B5.9, F10C2.7, F10F2.4, F11A5.5, F11A5.8, F11A5.9, F11A5.13, F11A5.15, F11A10.5, F11A10.6, F11C1.2, F11C1.3, F11E6.6, F11E6.8, F11E6.10, F13A7.13, F13B12.2, F13B12.3, F13B12.7, F13D2.2, F13D2.3, F13D12.5, F13D12.8, F13D12.10, F13E6.3, F13E6.5, F13E9.11, F13G3.7, F13G11.2, F13H10.3, F13H10.4, F13H10.5, F14B6.2, F14B6.5, F14D7.6, F14D7.8, F14D7.9, F14E5.1, F14E5.2, F14E5.3, F14F3.3, F14F4.1, F14F7.4, F14F8.3, F14F8.4, F14F8.12, F14F11.1, F14H3.2, F14H3.10, F15A4.1, F15A4.3, F15A4.5, F15A4.8, F15B9.6, F15D4.3, F15H10.4, F15H10.7, F15H10.8, F16A11.3, F16B12.1, F16C3.1, F16D3.6, F16D3.7, F17B5.2, F17C11.6, F17C11.7, F17C11.12, F17H10.2, F17H10.4, F18C12.4, F18E2.4, F19B2.8, F19B6.4, F19C6.5, F19H6.4, F19H8.5, F20C5.4, F20D1.1, F20D1.7, F20D1.8, F20D1.10, F20E11.1, F20E11.2, F20G2.6, F21A3.7, F21A10.2, F21D9.2, F21G4.1, F21H7.3, F21H7.12, F22B5.3, F22B5.4, F22B5.6, F22B5.10, F22B8.3, F22E10.5, F23A7.1, F23B2.3, F23B2.7, F23D12.1, F23D12.6, F23H12.2, F23H12.3, F23H12.7, F23H12.8, F25B3.2, F25D1.2, F25D1.4, F25D7.2, F25D7.5, F25H5.2, F25H5.8, F26A3.6, F26D2.10, F26D10.11, F26E4.2, F26E4.6, F26E4.11, F26F2.7, F27D4.7, F27E5.3, F27E5.5, F28C6.4, F28D1.6, F28D1.8, F28D1.9, F28D1.11, F28G4.1, F28G4.5, F28H7.6, F28H7.9, F29F11.2, F30A10.4, F30A10.6, F30A10.12, F30F8.9, F31B9.1, F31C3.4, F31C3.6, F31E9.5, F32A7.3, F32A11.1, F32A11.7, F32B6.9, F32D8.15, F32D8.5, F32D8.10, F32D8.14, F32G8.4, F32H5.4, F33A8.5, F33H2.8, F34D10.2, F34D10.3, F35B12.9, F35C5.11, F35C11.5, F35E2.1, F35E2.6, F35E12.4, F35H8.4, F35H8.6, F36A2.7, F36A2.9, F36D1.2, F36D1.8, F36D3.5, F36F2.4, F36G3.3, F36G9.3, F36G9.7, F36G9.12, F36G9.13, F36H1.5, F36H1.9, F36H1.10, F36H2.1, F36H2.2, F37B12.1, F37H8.2, F37H8.4, F38A1.11, F38B2.3, F38B7.2, F38C2.4, F39B2.8, F40D4.5, F40D4.6, F40D4.7, F40D4.9, F40E10.6, F40F8.3, F40F9.1, F40F9.5, F40F12.3, F40G12.1, F40G12.8, F41D3.2, F41D3.4, F41D3.5, F41D3.10, F41E7.1, F41E7.2, F41E7.3, F42A8.3, F42D1.3, F42E11.2, F42E11.3, F42F12.3, F42G4.7, F43D9.1, F43G9.2, F43G9.3, F43G9.8, F43G9.13, F44D12.2, F44D12.9, F44F4.1, F44F4.3, F44F4.9, F44G3.5, F44G3.7, F44G3.10, F44G4.2, F44G4.3, F44G4.6, F44G4.8, F45E6.1, F45E10.2, F45H10.4, F45H10.5, F46B3.11, F46B6.2, F46B6.9, F46C3.2, F46C5.2, F46C5.7, F46C5.8, F46F2.4, F46F3.2, F46G10.2, F47A4.1, F47B8.1, F47B8.2, F47B8.5, F47B8.7, F47B8.9, F47B8.10, F47B10.3, F47B10.5, F47B10.8, F47G9.1, F47G9.3, F48C5.1, F48C11.2, F48F5.6, F48F7.8, F49A5.2, F49A5.3, F49A5.5, F49A5.7, F49A5.9, F49B2.3, F49B2.6, F49C12.1, F49C12.4, F49C12.6, F49C12.12, F49C12.13, F49E11.2, F49E12.7, F49E12.9, F49E12.10, F49E12.12, F49H6.3, F49H6.11, F49H6.13, F52A8.1, F52B5.1, F52B11.3, F52D10.1, F52D10.4, F53A2.1, F53A2.2, F53B6.4, F53B6.6, F53B6.9, F53B7.2, F53B7.4, F53B7.5, F53C11.2, F53F1.1, F53F4.1, F53F4.2, F53F8.2, F53F8.6, F53H2.2, F54B3.2, F54B8.6, F54B8.7, F54B8.8, F54B8.9, F54B8.10, F54B8.11, F54B8.12, F54B11.8, F54C8.6, F54C9.7, F54D1.6, F54E4.2, F54F7.6, F54F11.3, F54F12.1, F54F12.2, F55A11.7, F55B12.2, F55D12.1, F55D12.6, F55F3.3, F55G11.1, F55H2.5, F55H12.3, F56A8.1, F56A8.3, F56A12.2, F56C4.2, F56D5.9, F56D5.10, F56G4.1, F56H6.11, F56H6.12, F56H11.2, F57A8.2, F57A8.4, F57A8.7, F57A10.4, F57B1.1, F57B1.5, F57B1.6, F57B7.1, F57C7.4, F57F5.3, F57G4.1, F57G8.5, F57G9.1, F57G9.2, F57G9.3, F57G9.4, F58D2.2, F58D5.2, F58D5.8, F58D12.1, F58E6.6, F58E6.8, F58E6.11, F58E10.6, F58G1.7, F58G6.2, F58G6.3, F58G6.4, F58G6.8, F58G11.1, F58G11.4, F58H1.5, F58H1.6, F59A1.10, F59A1.11, F59A1.12, F59A1.13, F59A1.15, F59B2.2, F59B2.13, F59B10.3, F59B10.4, F59B10.6, F59C6.2, F59C6.3, F59C6.11, F59D12.1, F59D12.2, F59F3.4, F59F4.2, F59F4.3, F59F4.4, F59F5.1, F59F5.3, H01G02.3, H04D03.1, H09F14.1, H12D21.5, H12D21.9, H12D21.11, H12I19.1, H12I19.2, H12I19.4, H12I19.5, H19J13.1, H19N07.4, H32K16.1, JC8.5, JC8.12, K01A6.6, K01A11.3, K01B6.3, K01D12.1, K01D12.6, K01H12.2, K02B9.3, K02B12.3, K02E2.1, K02E11.1, K02E11.2, K03A11.4, K03A11.5, K03D3.1, K03D3.4, K03D7.9, K03H1.5, K03H1.9, K03H1.12, K04D7.2, K04D7.4, K04D7.6, K04G2.9, K04G2.10, K04G11.1, K05C4.2, K05C4.10, K05C4.11, K05D4.3, K05D4.8, K05D4.9, K06A4.2, K06A4.4, K06B4.3, K06B4.4, K07A1.1, K07A1.13, K07A12.2, K07F5.6, K07F5.12, K07F5.15, K07G5.5, K08C7.1, K08D8.1, K08D8.2, K08E4.3, K08E4.5, K08E4.6, K08E7.4, K08F4.3, K08F8.7, K08F9.1, K08F9.3, K08G2.7, K08H10.6, K09A9.3, K09A9.6, K09C8.2, K09E4.2, K09E9.1, K09E9.2, K09E9.3, K09G1.1, K09G1.2, K10C8.2, K10C8.4, K10D6.1, K10D6.2, K10D11.5, K10G9.1, K10H10.1, K11D9.3, K11H3.7, K12D12.4, K12G11.1, K12G11.2, M01B2.3, M01B2.4, M01B2.6, M01B2.7, M01B2.9, M01E5.1, M01F1.4, M01F1.5, M01G12.6, M02B1.2, M02B1.3, M02B1.4, M03B6.2, M03B6.4, M03B6.5, M03C11.5, M04B2.7, M04C7.4, M04D8.4, M04D8.5, M04D8.6, M04D8.7, M04D8.8, M05B5.1, M05B5.2, M05B5.6, M05D6.5, M05D6.6, M18.8, M28.1, M28.2, M28.4, M28.8, M28.9, M79.2, M88.1, M117.1, M153.2, M162.3, M162.5, M176.4, M176.11, M199.1, MTCE.3, MTCE.4, MTCE.11, MTCE.12, MTCE.16, MTCE.23, MTCE.25, MTCE.26, MTCE.31, MTCE.34, MTCE.35, R02D5.3, R02D5.6, R03A10.6, R03D7.8, R03E1.2, R03E1.3, R04B5.1, R04B5.9, R04D3.1, R04D3.10, R04F11.1, R05D7.1, R05D7.3, R05D11.5, R05D11.9, R05H5.5, R07A4.2, R07B1.3, R07B1.9, R07B5.5, R07B7.4, R07B7.5, R07B7.6, R07B7.8, R07B7.9, R07B7.10, R07B7.12, R07D5.2, R07E3.1, R07E3.3, R07E3.4, R07E3.5, R07E5.7, R07E5.13, R07E5.17, R07H5.4, R08B4.4, R09A8.5, R09D1.1, R09D1.2, R09D1.3, R09D1.5, R09D1.6, R09D1.10, R09D1.11, R09D1.12, R09E10.5, R09H10.7, R10D12.1, R10D12.5, R10D12.6, R10D12.7, R10D12.9, R10D12.11, R10D12.17, R10E4.3, R10E4.6, R10E4.9, R10E8.5, R10E9.3, R10E11.7, R10E11.9, R10E12.2, R10H10.4, R11.4, R11D1.7, R11H6.2, R11H6.3, R12H7.4, R13.3, R13G10.4, R13H4.7, R31.2, R102.6, R102.8, R166.2, R186.6, T01C3.5, T01C3.11, T01D3.5, T01G1.2, T01G5.2, T01G5.6, T01G9.1, T01G9.3, T01H3.3, T01H3.4, T01H10.1, T01H10.2, T01H10.3, T01H10.5, T01H10.6, T01H10.7, T02B5.1, T02B5.3, T02C1.2, T02D1.4, T02D1.6, T02E1.6, T02E1.7, T02E9.1, T02E9.3, T02G6.4, T03D8.6, T03E6.2, T03E6.8, T03F6.6, T03F7.5, T03F7.6, T04A11.3, T04C12.1, T04C12.7, T04C12.8, T04F8.1, T04F8.2, T04F8.4, T04F8.9, T04H1.1, T04H1.7, T04H1.8, T05A1.3, T05A1.5, T05C12.4, T05C12.9, T05C12.11, T05D4.3, T05E11.2, T05E11.5, T05E12.2, T05F1.1, T05G5.5, T05G11.2, T06C12.8, T06C12.9, T06C12.10, T06C12.11, T06C12.12, T06D8.3, T06D8.5, T06D8.7, T06D8.9, T06E4.5, T06E4.7, T06E4.9, T06E6.5, T06E6.11, T06E8.1, T06E8.2, T07A5.3, T07C5.1, T07C12.6, T07D4.1, T07D10.2, T07F10.1, T07F10.4, T07G12.2, T07G12.5, T07G12.8, T08D2.2, T08D10.2, T08D10.3, T08G3.7, T09A5.11, T09A5.12, T09A5.15, T09B9.2, T09B9.5, T09E8.3, T09E8.4, T09E11.4, T09E11.5, T09E11.6, T09E11.7, T09F5.2, T10B9.1, T10B9.3, T10B9.5, T10B10.5, T10B10.8, T10C6.2, T10C6.6, T10G3.2, T10G3.4, T11A5.2, T11A5.3, T11A5.5, T11F9.1, T11F9.12, T11G6.2, T11G6.3, T11G6.4, T12A7.7, T12G3.4, T12G3.7, T13F2.5, T13F3.7, T13H5.6, T13H5.8, T13H10.2, T14C1.1, T14D7.2, T14G8.4, T14G10.7, T15D6.5, T15D6.11, T16A9.5, T16G12.4, T16G12.5, T16G12.8, T16H12.8, T18D3.3, T18D3.5, T19A6.1, T19A6.3, T19A6.4, T19B10.5, T19B10.8, T19B10.10, T19C4.1, T19C9.1, T19H5.1, T19H5.2, T19H5.3, T19H5.4, T20B3.13, T20D3.6, T20D3.8, T20D3.11, T20G5.4, T20G5.14, T21B4.1, T21B4.4, T21B6.5, T21B10.4, T21B10.6, T21C9.1, T21C9.3, T21C9.11, T21C9.12, T21C9.13, T22A3.6, T22C1.3, T22C1.9, T22C8.1, T22C8.7, T22G5.4, T22G5.5, T22H2.6, T23D5.8, T23F1.3, T23F1.5, T23G4.4, T23G7.2, T23G11.1, T23G11.6, T23G11.10, T24A11.2, T24B1.1, T24F1.2, T24F1.4, T24F1.7, T25B9.7, T25E12.7, T25E12.8, T25E12.9, T25E12.10, T25E12.11, T25E12.13, T26C5.3, T26E4.4, T26E4.14, T26E4.15, T26G10.5, T26H2.2, T26H2.7, T26H8.4, T27C5.8, T27C5.10, T27D1.3, T27D12.1, T27E7.3, T27E7.4, T27E7.5, T27E7.9, T27E9.5, T27E9.9, T27F6.6, T27F6.7, T28B8.5, T28C6.3, T28C6.8, T28F3.4, T28F3.5, T28F3.9, T28F4.2, VB0393L.2, VC27A7L.1, VF39H2L.1, W01A8.3, W01A8.4, W01B6.3, W01B6.8, W01D2.3, W01D2.4, W01D2.5, W01D2.6, W02A2.5, W02B8.1, W02B8.3, W02B8.4, W02D9.2, W02D9.8, W02D9.9, W03C9.6, W03C9.8, W03G11.2, W04D2.2, W04E12.2, W04E12.3, W04E12.4, W04E12.5, W04E12.9, W04G3.1, W04G3.6, W04G5.5, W05B5.2, W05E10.1, W05E10.2, W05H5.1, W05H5.3, W05H5.5, W05H5.6, W05H5.7, W05H12.1, W06A7.2, W06A7.4, W06D4.2, W06D12.1, W06F12.2, W06G6.1, W06G6.7, W06H3.1, W07A8.1, W07A8.5, W07A12.6, W07A12.7, W07G1.2, W07G1.5, W07G1.6, W07G4.5, W08D2.3, W08D2.5, W08G11.5, W09D10.2, W09D10.5, W09G3.8, Y2H9A.4, Y6B3B.3, Y6B3B.5, Y6B3B.7, Y6B3B.11, Y6E2A.1, Y6E2A.9, Y6G8.4, Y7A5A.1, Y7A9A.1, Y9C2UA.1, Y11D7A.3, Y11D7A.9, Y15E3A.4, Y17D7A.4, Y17G7B.19, Y18D10A.4, Y18D10A.11, Y18D10A.12, Y18D10A.23, Y26D4A.3, Y26D4A.5, Y26G10.1, Y32B12B.1, Y32B12B.3, Y32F6A.4, Y32F6B.1, Y37A1A.2, Y37A1A.3, Y37A1C.1, Y37D8A.5, Y37D8A.6, Y37D8A.8, Y37D8A.10, Y37D8A.12, Y37D8A.16, Y37D8A.17, Y37D8A.22, Y37D8A.26, Y37H2C.1, Y37H2C.4, Y38F1A.2, Y38F1A.4, Y38F1A.7, Y38F1A.8, Y38H6C.16, Y38H6C.17, Y38H6C.20, Y38H6C.21, Y38H8A.7, Y39A1A.8, Y39A1A.20, Y39A1A.22, Y39B6A.8, Y39B6A.10, Y39B6A.18, Y39B6A.27, Y39B6A.29, Y39B6A.30, Y39B6A.32, Y39B6A.41, Y39C12A.5, Y39C12A.6, Y39C12A.7, Y39E4A.1, Y39E4A.2, Y39E4B.5, Y39E4B.7, Y39G8B.3, Y39G8B.4, Y40H7A.9, Y40H7A.11, Y41C4A.11, Y41C4A.13, Y41C4A.17, Y41C4A.18, Y41E3.3, Y41E3.8, Y41E3.12, Y41E3.14, Y41E3.15, Y42A5A.1, Y43C5A.3, Y43D4A.3, Y43D4A.4, Y43F4B.7, Y43F8A.3, Y43F8A.5, Y43F8B.10, Y43F8C.11, Y44A6B.1, Y44A6B.2, Y44A6D.6, Y44A6E.1, Y45F10B.13, Y45F10D.15, Y46G5A.2, Y46G5A.5, Y46G5A.8, Y46G5A.17, Y46G5A.22, Y46G5A.26, Y47D3A.5, Y47D3A.11, Y47D3A.32, Y47D3B.11, Y47H9A.1, Y47H9C.2, Y48A6B.1, Y48A6B.6, Y48B6A.10, Y48C3A.5, Y48C3A.11, Y48E1B.2, Y48E1B.14, Y49A3A.1, Y49E10.11, Y49E10.20, Y50E8A.11, Y51A2B.2, Y51A2D.4, Y51A2D.5, Y51A2D.18, Y51B9A.6, Y51B9A.7, Y51H4A.16, Y51H4A.21, Y51H4A.25, Y52B11A.1, Y52B11A.7, Y53C10A.5, Y53C12A.3, Y53F4B.12, Y53F4B.25, Y53H1B.1, Y53H1C.3, Y54E2A.1, Y54E2A.5, Y54E2A.7, Y54E2A.10, Y54E2A.12, Y54E5A.1, Y54E5A.2, Y54E5A.8, Y54G9A.4, Y54G9A.5, Y54G9A.9, Y54G11A.14, Y54G11B.1, Y56A3A.2, Y56A3A.22, Y57A10A.3, Y57A10A.10, Y57A10A.14, Y57A10A.28, Y57A10A.35, Y57A10B.1, Y57A10B.4, Y57A10B.5, Y57A10C.1, Y57A10C.3, Y57A10C.4, Y57A10C.7, Y57A10C.8, Y57A10C.9, Y57A10C.10, Y57G11B.7, Y57G11C.2, Y57G11C.4, Y57G11C.15, Y57G11C.17, Y57G11C.23, Y57G11C.31, Y57G11C.37, Y57G11C.44, Y57G11C.45, Y57G11C.46, Y57G11C.49, Y59A8A.3, Y59A8B.21, Y60A3A.14, Y60A3A.19, Y62E10A.4, Y62E10A.10, Y62H9A.1, Y62H9A.10, Y62H9A.11, Y62H9A.12, Y63D3A.8, Y64G10A.6, Y64G10A.8, Y66A7A.7, Y66D12A.11, Y66D12A.13, Y66D12A.21, Y66D12A.24, Y67A10A.1, Y67A10A.3, Y67A10A.8, Y67A10A.9, Y67H2A.4, Y67H2A.5, Y68A4A.2, Y68A4A.6, Y69E1A.1, Y69E1A.3, Y69H2.1, Y69H2.2, Y69H2.9, Y69H2.11, Y69H2.12, Y69H2.14, Y70C5A.2, Y70D2A.1, Y70G10A.2, Y70G10A.3, Y71A12B.11, Y71A12C.2, Y73F8A.2, Y73F8A.5, Y73F8A.11, Y73F8A.30, Y75B8A.5, Y75B8A.16, Y75B8A.33, Y75B8A.34, Y75B12A.2, Y75B12B.10, Y76A2B.4, Y76A2B.6, Y79H2A.2, Y80D3A.5, Y80D3A.8, Y87G2A.13, Y87G2A.18, Y102A5B.1, Y102A5C.23, Y102A5C.25, Y102A5C.33, Y105C5A.23, Y105C5B.1, Y105C5B.8, Y105C5B.16, Y105C5B.17, Y105C5B.23, Y105E8A.3, Y105E8A.12, Y105E8A.27, Y106G6G.4, Y106G6H.8, Y106G6H.16, Y111B2A.19, Y111B2A.20, Y113G7A.5, Y113G7A.10, Y113G7A.14, Y113G7B.9, Y113G7B.11, Y113G7B.12, Y113G7C.1, Y116A8B.5, Y116A8C.8, Y116A8C.9, Y116A8C.23, Y116A8C.40, ZC84.4, ZC84.7, ZC101.3, ZC374.1, ZC376.1, ZC376.2, ZC376.3, ZC410.5, ZC412.1, ZC443.5, ZC443.6, ZC455.3, ZC455.4, ZC455.5, ZC455.6, ZC455.7, ZC455.8, ZC455.9, ZC455.11, ZC482.3, ZC482.5, ZC482.7, ZC506.3, ZC518.1, ZC518.4, ZK218.4, ZK265.5, ZK287.2, ZK287.9, ZK455.3, ZK512.1, ZK512.11, ZK550.1, ZK550.2, ZK593.3, ZK596.3, ZK632.10, ZK637.1, ZK637.3, ZK643.3, ZK666.12, ZK678.4, ZK757.1, ZK757.4, ZK792.1, ZK792.7, ZK795.2, ZK795.4, ZK809.8, ZK822.5, ZK829.9, ZK836.3, ZK849.4, ZK849.5, ZK858.1, ZK858.5, ZK858.6, ZK858.7, ZK863.1, ZK863.8, ZK892.3, ZK892.5, ZK896.9, ZK899.2, ZK930.2, ZK930.6, ZK938.6, ZK938.7, ZK945.1, ZK970.1, ZK971.1, ZK1010.5, ZK1010.6, ZK1010.8, ZK1025.3, ZK1037.3, ZK1037.6, ZK1037.11, ZK1053.2, ZK1053.6, ZK1067.4, ZK1073.2, ZK1086.1, ZK1086.2, ZK1251.3, ZK1307.7, ZK1320.5, ZK1321.2, D2023.1, C06G8.3, C18D4.5, ZK675.3, AH9.1, AH9.4, B0034.5, B0041.5, B0205.2, B0207.8, B0207.9, B0207.10, B0212.1, B0212.2, B0212.4, B0218.1, B0218.2, B0222.1, B0222.2, B0222.3, B0222.10, B0228.6, B0238.3, B0238.5, B0238.6, B0238.8, B0244.4, B0244.5, B0244.6, B0244.7, B0244.8, B0244.9, B0244.10, B0244.11, B0252.1, B0252.3, B0303.4, B0303.8, B0303.11, B0304.3, B0310.1, B0310.3, B0310.5, B0336.11, B0348.1, B0348.2, B0361.7, B0361.8, B0361.11, B0410.2, B0414.1, B0416.1, B0416.3, B0416.5, B0432.1, B0454.5, B0454.6, B0495.7, B0495.9, B0496.5, B0496.6, B0511.2, B0511.12, B0511.13, B0511.14, B0546.3, B0546.5, B0554.5, B0554.7, B0563.6, C01B4.7, C01B4.8, C01B4.9, C01B10.4, C01B10.7, C01B10.9, C01B10.10, C01B12.3, C01B12.4, C01B12.5, C01C10.3, C01F1.2, C01F1.4, C01G5.4, C02A12.2, C02A12.3, C02A12.5, C02A12.6, C02B8.5, C02C2.4, C02C2.5, C02D5.2, C02E7.1, C02E11.1, C02F5.5, C02F5.13, C02H6.1, C02H7.2, C03A7.9, C03A7.10, C03A7.11, C03B1.13, C03B8.1, C03F11.1, C03F11.3, C03G6.13, C03G6.16, C03H5.2, C03H5.6, C03H5.7, C04C3.1, C04C3.2, C04C3.6, C04C3.7, C04E6.2, C04E6.4, C04E12.4, C04E12.5, C04E12.9, C04F5.2, C04F5.4, C04F5.5, C04F5.6, C04F5.7, C04F6.2, C04G6.11, C05D9.2, C05D9.7, C05D10.3, C05D11.7, C05E11.1, C05E11.2, C05E11.3, C06A5.10, C06E1.1, C06E1.3, C06E1.11, C06G3.4, C06G4.4, C06G4.5, C07D10.3, C07G3.9, C07H6.9, C08A9.3, C08D8.1, C08F1.6, C08F1.8, C08F1.10, C08G5.1, C08G5.6, C08G9.1, C08G9.2, C09B8.3, C09B8.8, C09B9.1, C09B9.2, C09B9.3, C09B9.7, C09D4.1, C09D4.6, C09E7.1, C09E8.1, C09E8.3, C09F5.1, C09F5.2, C09G12.2, C09G12.3, C09G12.6, C09H5.2, C10A4.2, C10A4.3, C10A4.4, C10A4.5, C10A4.6, C10A4.7, C10E2.5, C10E2.6, C10H11.3, C10H11.4, C10H11.5, C10H11.6, C12D5.11, C12D12.3, C13A2.1, C13A2.5, C13A2.9, C13A10.1, C13B7.1, C13B9.4, C13D9.4, C13D9.5, C13D9.6, C13D9.9, C13F10.1, C13F10.5, C14B9.3, C14C11.4, C14E2.5, C14F5.1, C14F5.4, C15B12.2, C15C7.1, C15C7.6, C15H9.3, C15H9.4, C15H9.5, C16B8.4, C16C4.6, C16C4.7, C16C4.13, C16C8.17, C16D9.4, C16D9.6, C16D9.7, C17A2.3, C17A2.5, C17B7.3, C17B7.5, C17E7.3, C17E7.9, C17E7.12, C17E7.13, C17F3.1, C17G10.7, C17H11.1, C17H11.6, C17H12.2, C17H12.4, C17H12.10, C18A3.2, C18A3.4, C18A3.9, C18A3.10, C18A11.3, C18B2.6, C18B10.1, C18B10.2, C18B10.3, C18B10.4, C18B10.5, C18C4.3, C18G1.1, C18G1.8, C18H2.1, C18H2.3, C18H2.4, C18H2.5, C18H7.6, C18H7.8, C18H9.5, C23G10.6, C23G10.10, C23H3.2, C23H5.7, C24A8.1, C24A11.2, C24B5.1, C24B9.2, C24B9.4, C24B9.5, C24B9.6, C24B9.9, C24D10.2, C24D10.4, C24D10.5, C24G6.2, C24G7.1, C24G7.2, C24G7.4, C24H10.1, C25A11.1, C25B8.4, C25B8.5, C25B8.7, C25E10.3, C25E10.4, C25E10.5, C25F6.4, C25F6.7, C25G6.5, C25H3.9, C26B2.7, C26B2.8, C26B9.1, C26F1.6, C27A12.9, C27D6.3, C27F2.6, C27F2.8, C27H5.4, C27H5.6, C28C12.11, C28H8.4, C29F5.5, C29F9.8, C29G2.4, C29G2.5, C29H12.2, C30A5.10, C30B5.2, C30B5.5, C30B5.7, C30E1.2, C30E1.3, C30E1.6, C30E1.7, C30F12.3, C30F12.5, C30F12.6, C30G4.4, C30G12.4, C31B8.1, C32D5.7, C32F10.4, C33C12.4, C33D12.2, C33F10.1, C33F10.11, C33G8.5, C33H5.1, C33H5.11, C33H5.14, C33H5.18, C34B2.8, C34B2.9, C34B2.10, C34B2.11, C34D4.4, C34D10.1, C34G6.6, C34H3.1, C35A11.1, C35A11.4, C35B1.3, C35D10.1, C35D10.3, C35D10.8, C35D10.10, C36B7.3, C36B7.4, C36B7.6, C36C5.2, C36C5.3, C36C5.6, C36C5.7, C36C5.8, C36C5.9, C36C5.10, C36C5.11, C37C3.7, C37C3.11, C37C3.12, C39B5.1, C39D10.2, C39D10.6, C39F7.1, C39H7.4, C39H7.5, C40A11.8, C41D11.9, C41G11.4, C42C1.1, C42C1.7, C42C1.8, C42C1.12, C42C1.16, C42D4.2, C42D8.1, C43G2.1, C43G2.2, C43G2.4, C43H6.6, C43H6.7, C44B7.3, C44B7.4, C44B7.6, C44B7.7, C44B7.11, C44C1.1, C45E1.4, C45G9.11, C45H4.1, C45H4.3, C45H4.6, C45H4.7, C45H4.8, C45H4.9, C45H4.10, C45H4.11, C45H4.13, C45H4.16, C46A5.2, C46C11.2, C46C11.3, C46E10.2, C46F4.2, C46G7.1, C48A7.2, C48B6.5, C48B6.9, C48B6.10, C48C5.1, C48C5.3, C48E7.1, C48E7.6, C48E7.8, C49A9.7, C49A9.8, C49C8.2, C49D10.4, C49D10.7, C49D10.8, C49G7.1, C50A2.3, C50D2.2, C50D2.9, C50E3.7, C50E3.8, C50E3.9, C50E10.3, C50E10.5, C50E10.6, C50E10.7, C50E10.8, C50E10.9, C50E10.10, C50E10.11, C50F2.7, C50F7.1, C50H11.2, C50H11.3, C50H11.4, C50H11.5, C50H11.8, C50H11.10, C50H11.11, C50H11.13, C50H11.14, C52B9.4, C52B11.3, C52D10.12, C52E2.7, C53C11.2, C53D5.1, C53D5.2, C53D5.5, C54A12.2, C54E4.4, C54F6.4, C54F6.7, C54F6.11, C54G6.3, C54G7.2, C55B7.6, C55C2.3, C55C2.4, C55C3.2, C55C3.6, C56E6.5, C56G2.1, C56G3.1, CD4.1, D1007.3, D1007.5, D1007.10, D1007.15, D1009.1, D1009.3, D1014.2, D1044.2, D1065.1, D1069.1, D1069.4, D1073.1, D1079.1, D2024.5, D2062.4, D2062.5, D2062.7, D2063.2, D2092.1, D2092.4, D2092.5, D2096.2, E02D9.1, E02H9.6, E03D2.4, E03H12.3, E03H12.6, E04A4.5, E04F6.4, E04F6.10, E_BE45912.2, F01E11.1, F01E11.3, F01E11.5, F01F1.14, F01G12.1, F02C9.1, F02C9.3, F02C9.4, F02E8.2, F02E8.5, F02E11.1, F02E11.2, F02G3.1, F07B7.14, F07C3.2, F07C3.9, F07C3.10, F07E5.8, F07F6.2, F07F6.7, F07F6.8, F07G6.1, F07G11.1, F07G11.3, F08C6.5, F08F1.3, F08F3.8, F08F8.6, F08F8.8, F09C12.6, F09E5.11, F09E10.6, F09F7.1, F09F9.1, F09F9.4, F09G2.1, F09G2.3, F09G2.5, F09G2.6, F09G2.8, F09G8.9, F10C5.2, F10D2.2, F10D2.5, F10D2.6, F10D2.7, F10D2.11, F10D2.12, F10D7.1, F10D7.2, F10E7.2, F10E7.3, F10E7.6, F10E7.9, F10E7.10, F10E9.1, F10E9.10, F10G7.5, F10G7.7, F11C7.1, F11D5.3, F11D5.5, F11G11.4, F11G11.5, F11G11.9, F11H8.2, F12A10.4, F12B6.2, F12B6.3, F12D9.1, F13A2.6, F13B6.2, F13B9.6, F13C5.1, F13C5.5, F13D11.1, F13H6.5, F13H8.4, F13H8.11, F14B8.5, F14B8.6, F14B8.7, F14D2.6, F14D12.5, F14F9.6, F14H12.6, F14H12.7, F14H12.8, F15A8.4, F15B10.1, F15E6.2, F15E6.8, F15E11.10, F16B4.10, F16F9.1, F16H11.1, F16H11.3, F17E9.5, F17E9.7, F17E9.8, F18A1.1, F18A12.2, F18A12.5, F18A12.8, F18C5.10, F18E3.1, F18E3.10, F18F11.3, F18F11.5, F18G5.4, F19C7.5, F19C7.6, F20A1.2, F20A1.3, F20A1.4, F20B4.6, F20D6.10, F20D12.7, F21C10.1, F21C10.3, F21C10.4, F21C10.12, F21D12.3, F21E9.2, F21F3.3, F21F3.6, F21F3.7, F21F8.2, F21F8.11, F21H12.2, F22E5.9, F22E5.11, F22F7.1, F22F7.3, F22F7.4, F23F1.6, F23F12.3, F23H11.9, F25B4.7, F25E2.1, F25E5.2, F25G6.7, F26A1.6, F26A1.7, F26A1.8, F26F4.4, F26F4.9, F26F12.4, F26F12.5, F26G1.6, F26G5.10, F27C1.2, F27D9.2, F27D9.7, F27E11.1, F27E11.2, F28A10.3, F28A10.7, F28A12.1, F28A12.3, F28B3.5, F28B3.6, F28B3.9, F28B3.10, F28B4.1, F28B4.4, F28E10.2, F28F9.2, F28H1.4, F29A7.4, F29B9.7, F29B9.8, F29B9.11, F30B5.6, F30B5.7, F31D5.1, F31D5.2, F31D5.4, F31D5.5, F31E3.6, F31E8.1, F31E8.4, F31F4.7, F31F4.15, F31F4.17, F31F7.2, F32B5.4, F32D1.3, F33D4.4, F33D4.7, F33D11.5, F33D11.9, F33D11.11, F33D11.12, F34D6.4, F35A5.5, F35C8.5, F35C8.7, F35D2.3, F35D2.4, F35D11.3, F35D11.5, F35F10.2, F35F10.9, F35F10.10, F35G8.1, F35H10.10, F35H12.6, F36D4.4, F36H5.4, F36H5.5, F36H5.10, F36H9.4, F36H9.5, F36H9.6, F36H12.15, F37A4.1, F37A4.3, F37A4.4, F37B4.7, F37B4.9, F37C4.1, F37C4.2, F37C4.3, F37C4.4, F37C4.7, F37C12.2, F37E3.2, F38A5.5, F38A5.8, F38A5.9, F38A5.10, F38A5.11, F38A5.12, F38A5.14, F38B6.3, F38B6.6, F38E1.6, F38E1.8, F38E1.9, F38H12.5, F39B3.2, F39E9.12, F39G3.1, F39G3.4, F39G3.5, F39G3.6, F39H12.4, F40A3.2, F40A3.4, F40A3.5, F40A3.7, F40B5.2, F40E12.2, F40G9.1, F40G9.8, F40G9.9, F40H6.1, F41A4.1, F41B4.3, F41C3.2, F41C3.4, F41C3.8, F41C6.7, F41D9.5, F41E6.14, F41F3.1, F41H8.1, F41H8.2, F41H10.5, F41H10.11, F42A6.1, F42A9.3, F42A9.6, F42A9.9, F42C5.2, F42C5.6, F42G8.5, F42G8.6, F42G8.9, F42G8.10, F42G9.6, F43B10.1, F43B10.2, F43C11.6, F44A2.2, F44B9.5, F44C8.7, F44E2.10, F44E7.7, F44E7.9, F45C12.5, F45C12.6, F45E4.6, F45E4.7, F45E4.11, F45F2.1, F45F2.5, F45F2.6, F45F2.7, F46C8.3, F46F5.6, F46F5.9, F46F5.16, F46G11.1, F47B3.3, F47B7.2, F47C10.6, F47D2.1, F47D2.3, F47D2.4, F47D2.5, F47D2.6, F47D2.7, F47D2.8, F47D12.6, F47E1.2, F47E1.4, F47F6.3, F47G6.3, F48A11.4, F48B9.2, F48C1.1, F48C1.2, F48C1.3, F48C1.4, F48C1.6, F48E3.2, F48E3.6, F48G7.6, F49E7.2, F49F1.3, F49F1.12, F49H12.6, F52C6.4, F52E1.9, F52F10.2, F52F10.3, F52F10.4, F52F10.5, F52H2.4, F52H2.6, F53A3.1, F53A9.4, F53A9.5, F53B1.2, F53B1.3, F53B1.8, F53B3.5, F53C3.3, F53C3.4, F53C3.5, F53C3.6, F53C3.13, F53G12.3, F53G12.8, F53H8.3, F54C1.1, F54C1.8, F54D7.2, F54D7.3, F54E2.5, F54E2.6, F54E7.1, F54E7.5, F54F2.9, F55A3.1, F55A4.1, F55A4.3, F55A4.8, F55A4.10, F55A12.2, F55C12.4, F55C12.5, F55D10.3, F55D10.4, F55D10.5, F55E10.1, F55E10.2, F55E10.4, F55E10.5, F55E10.6, F55E10.7, F55F8.6, F55F8.9, F55G1.1, F55G1.6, F55G1.12, F55G1.15, F56A4.7, F56A4.10, F56A4.11, F56A4.12, F56A11.4, F56A11.5, F56A11.6, F56B3.6, F56B3.7, F56B3.11, F56C3.3, F56C3.4, F56C3.5, F56C3.6, F56C3.7, F56C9.3, F56C11.5, F56D1.2, F56D6.4, F56D6.5, F56E10.3, F56F4.1, F56F4.3, F56F11.5, F56H1.2, F56H1.3, F57B10.5, F57C9.6, F57F4.2, F57F10.1, F57H12.4, F58A6.2, F58A6.5, F58A6.6, F58A6.10, F58A6.11, F58D7.1, F58E2.9, F58F6.5, F58F6.6, F58F12.2, F58G4.4, F58H7.3, F59A3.1, F59A3.4, F59A3.7, F59A7.3, F59B1.4, F59B1.6, F59D6.6, F59E12.8, F59G1.1, F59H6.4, H03E18.2, H04J21.1, H04M03.2, H04M03.11, H04M03.12, H06H21.10, H06I04.5, H10E21.1, H10E21.2, H10E21.5, H11E01.2, H14E04.3, H14N18.4, H16O14.1, H17B01.1, H20E11.1, H20J04.1, H20J04.6, H20J04.7, H22D07.1, H22K11.4, H23L24.4, H23N18.1, H23N18.2, H23N18.3, H24O09.1, H27M09.5, H32C10.3, H34P18.1, H35N09.2, H41C03.3, K01A2.1, K01A2.3, K01A2.4, K01A2.6, K01A2.8, K01A2.9, K01A12.3, K02A2.3, K02D7.5, K02E7.4, K02E7.10, K02E7.11, K02E10.4, K02E10.5, K02F3.6, K02F3.8, K02F6.3, K02F6.4, K02G10.1, K02G10.3, K02G10.5, K03A1.2, K03C7.3, K03E5.1, K03H6.1, K03H6.4, K03H6.5, K04A8.2, K04A8.10, K04E7.1, K04F1.1, K04F1.12, K05F1.1, K05F1.6, K05F1.8, K06A1.2, K06A1.3, K06A5.2, K06A9.3, K06C4.8, K06C4.9, K06C4.17, K06H6.3, K07B1.4, K07B1.5, K07E3.7, K07E8.5, K07H8.2, K07H8.5, K08B4.3, K08B4.4, K08B5.1, K08D9.1, K08D9.6, K08D10.10, K08D10.11, K08F11.5, K09C4.1, K09C4.4, K09C4.5, K09C6.4, K09C6.5, K09C6.6, K09D9.3, K09D9.11, K09E10.1, K09E10.2, K09F5.1, K09F5.4, K09F6.3, K09F6.4, K09H11.6, K10B4.2, K10B4.4, K10C2.2, K10C2.3, K10C9.4, K10D2.5, K11C4.2, K11D12.5, K11D12.8, K11G9.5, K11G12.6, K11H12.3, K11H12.8, K12B6.2, K12B6.4, K12B6.6, K12C11.3, K12H4.5, K12H6.2, K12H6.6, M01A10.3, M01A12.3, M01E11.1, M01G5.3, M01H9.4, M02A10.1, M02B7.4, M02H5.2, M02H5.12, M03A1.3, M03E7.2, M03E7.3, M03E7.5, M03F4.4, M03F8.1, M04G7.1, M04G7.3, M60.4, M60.6, M70.1, M70.3, PDB1.1, R01B10.3, R01B10.4, R01B10.5, R01B10.6, R01H2.3, R02E12.6, R02F2.8, R02F11.2, R02F11.3, R03E9.2, R03E9.3, R03G5.3, R03G5.7, R03H4.1, R03H4.4, R03H4.5, R03H10.2, R04A9.1, R04E5.2, R05C11.3, R05D3.2, R05F9.7, R05G9.2, R05G9.3, R06A10.1, R06B10.1, R08C7.2, R08F11.1, R08F11.2, R08F11.6, R09B5.4, R09B5.6, R09B5.11, R09E12.1, R09E12.2, R09F10.1, R09F10.5, R09H3.1, R10A10.1, R10F2.1, R10H1.2, R11E3.2, R11F4.2, R11G1.1, R11G1.6, R11G11.3, R11G11.12, R12B2.7, R12C12.3, R12C12.4, R12C12.6, R12C12.9, R12E2.2, R13A1.9, R13A5.4, R13A5.9, R13D7.10, R13D11.1, R13D11.3, R13D11.4, R13D11.6, R13F6.5, R13H7.2, R57.1, R105.1, R106.2, R119.1, R144.5, R144.6, R144.11, R148.4, R151.1, R151.6, R155.1, R155.2, R155.3, R155.4, R160.3, R160.4, R160.6, R173.3, R193.3, SSSD1.1, T01A4.1, T01B10.5, T01B11.1, T01B11.4, T01C8.2, T01D1.5, T02B11.6, T02C5.1, T02C5.3, T02G5.3, T02G5.12, T02G5.14, T03D3.1, T03F1.12, T03G11.4, T04A6.1, T04A6.2, T04B8.5, T04C9.2, T04G9.5, T05A7.8, T05A8.7, T05B4.1, T05B4.8, T05E7.4, T05H4.1, T05H4.4, T05H4.5, T06A4.2, T06C10.2, T06D4.4, T06F4.1, T07A9.12, T07D1.3, T07D3.6, T07E3.6, T07F8.2, T07F12.2, T07H6.1, T07H8.7, T08B1.1, T08B2.3, T08B6.4, T08H10.4, T09A12.5, T09B4.1, T09B4.2, T09B4.5, T09H2.1, T10E9.4, T10E9.5, T10E9.6, T10E9.8, T10E10.3, T10H9.1, T10H9.3, T11F8.1, T11F8.2, T12A2.2, T12A2.15, T12B3.2, T12B3.3, T12F5.2, T13C2.6, T13C2.7, T13C5.6, T13G4.3, T14B4.3, T14B4.5, T14E8.1, T14E8.3, T14G11.1, T14G12.6, T15B7.2, T15B7.6, T15B7.8, T15B7.11, T15B7.12, T15B7.13, T15B7.14, T15B7.16, T16A1.2, T17A3.10, T19C3.1, T19C3.4, T19D2.3, T19D7.1, T19D7.5, T19D7.7, T19D12.4, T19D12.7, T19D12.9, T19D12.10, T19F4.1, T19H12.1, T19H12.6, T19H12.8, T19H12.9, T19H12.10, T19H12.11, T20B12.9, T20D4.1, T20D4.2, T20D4.13, T20D4.18, T20F5.5, T20H9.6, T21D9.2, T21D12.9, T21E12.2, T21E12.5, T21F2.1, T21G5.2, T21H3.4, T21H3.5, T22B2.2, T22B2.3, T22B2.5, T22B11.2, T22B11.4, T22D1.4, T22D1.8, T22D1.11, T22D1.12, T22E5.6, T22E7.1, T22E7.2, T22F3.7, T22F3.8, T22F3.10, T22F3.11, T22F7.1, T22H9.2, T23B3.2, T23B3.4, T23B3.6, T23B12.5, T23C6.5, T23F2.3, T23F2.5, T24A6.5, T24A6.10, T24A6.13, T24C4.4, T24C4.7, T24C12.1, T24C12.4, T24D8.1, T24E12.5, T24H7.5, T25D1.1, T25D3.4, T25D10.2, T25E4.2, T25F10.3, T25F10.4, T25G12.6, T26A5.4, T26A8.1, T26A8.2, T26C12.2, T27A1.4, T27A1.5, T27C4.1, T27C4.2, T27C10.1, T27C10.2, T27E4.6, T27F7.3, T28A11.6, T28A11.7, T28A11.15, T28B4.2, T28B4.4, T28C12.1, T28C12.2, T28D9.3, T28D9.4, T28D9.7, T28F2.7, T28F12.1, T28H11.8, W01A11.2, W01B11.1, W01B11.2, W02B3.6, W02C12.1, W02C12.2, W02D3.4, W02D7.3, W02D7.8, W02D7.11, W02F12.2, W02H5.4, W02H5.5, W03A5.2, W03B1.2, W03B1.7, W03B1.8, W03D2.6, W03D8.3, W03D8.9, W03D8.10, W03F8.6, W03F9.4, W03F11.4, W03G9.3, W04B5.2, W04B5.6, W04C9.3, W04C9.6, W05E7.2, W05F2.2, W05F2.3, W05G11.2, W05H7.2, W05H9.1, W06B11.3, W06H8.4, W07E6.3, W07E6.5, W09B6.5, W09B7.3, W10C4.1, W10C8.3, W10C8.5, W10D9.1, W10G11.5, W10G11.16, Y4C6A.2, Y4C6A.3, Y4C6B.2, Y4C6B.3, Y4C6B.4, Y4C6B.5, Y5H2B.1, Y5H2B.4, Y5H2B.7, Y9C9A.5, Y9C9A.8, Y9C9A.15, Y9C9A.17, Y9C12A.1, Y14H12A.1, Y17G9A.4, Y19D10A.1, Y19D10A.2, Y19D10A.4, Y19D10A.5, Y19D10A.8, Y19D10A.10, Y19D10A.11, Y19D10A.12, Y19D10A.13, Y19D10B.5, Y22D7AL.1, Y22D7AL.3, Y22D7AL.11, Y22D7AL.12, Y22D7AL.15, Y22D7AR.2, Y22D7AR.7, Y22D7AR.14, Y23H5B.2, Y23H5B.4, Y24D9A.6, Y24D9A.7, Y25C1A.7, Y32G9A.8, Y32H12A.1, Y32H12A.5, Y32H12A.6, Y34B4A.7, Y34D9A.2, Y34D9A.8, Y34F4.1, Y34F4.2, Y34F4.5, Y35H6.1, Y37E3.16, Y37E11AL.1, Y37E11AL.2, Y37E11AL.5, Y37E11AR.1, Y37E11AR.5, Y37F4.5, Y38C1AA.1, Y38C1AA.5, Y38C1AA.7, Y38C1AA.8, Y38C1AB.2, Y38C1AB.6, Y38C1BA.1, Y38C9B.2, Y38F2AL.1, Y38F2AR.2, Y38F2AR.3, Y38F2AR.7, Y38F2AR.9, Y39A3A.3, Y39A3B.2, Y39A3B.3, Y39A3B.5, Y39A3CL.3, Y39D8A.1, Y39D8B.1, Y39D8B.3, Y39G10AR.5, Y39G10AR.6, Y39G10AR.16, Y39G10AR.22, Y39H10A.2, Y40B10A.3, Y40B10A.5, Y40B10A.9, Y40C5A.4, Y40D12A.2, Y41D4A.4, Y41D4A.8, Y41D4B.1, Y41D4B.24, Y41G9A.2, Y41G9A.4, Y42G9A.6, Y42H9AR.2, Y43H11AL.2, Y45G12B.2, Y45G12C.1, Y45G12C.5, Y45G12C.6, Y45G12C.7, Y45G12C.8, Y45G12C.9, Y45G12C.10, Y46B2A.3, Y46H3A.1, Y46H3D.4, Y47D7A.14, Y47D9A.3, Y47D9A.5, Y47G6A.3, Y47G6A.7, Y47G7B.2, Y48G1BL.5, Y48G1BM.9, Y48G1C.5, Y48G8AL.13, Y48G9A.10, Y49C4A.1, Y49C4A.2, Y49C4A.3, Y49C4A.8, Y49F6B.9, Y49F6B.11, Y50D4A.2, Y50D4B.4, Y50D4B.5, Y50D4B.7, Y51F10.3, Y51F10.4, Y51H7BR.3, Y51H7BR.4, Y51H7BR.7, Y51H7C.10, Y52D5A.1, Y52E8A.4, Y53G8AM.4, Y53G8AM.7, Y53G8AR.7, Y53G8B.2, Y53G8B.3, Y53G8B.4, Y54E10BL.3, Y54E10BR.1, Y54E10BR.5, Y54F10AL.1, Y54F10AM.7, Y54F10BM.6, Y54G2A.2, Y54G2A.4, Y54G2A.11, Y54G2A.13, Y54G2A.16, Y54G2A.18, Y54G2A.35, Y54G2A.36, Y55B1BM.1, Y55D5A.6, Y55F3AR.1, Y55F3AR.2, Y55F3BL.2, Y55F3BR.4, Y55F3BR.7, Y55F3C.2, Y55F3C.3, Y55F3C.7, Y55F3C.8, Y55H10A.1, Y55H10A.2, Y57E12AL.1, Y57E12AL.4, Y57E12AM.1, Y57E12B.1, Y57E12B.4, Y57G7A.6, Y57G7A.7, Y57G7A.8, Y58A7A.1, Y58A7A.5, Y58G8A.1, Y58G8A.4, Y59C2A.2, Y59E1A.1, Y59E9AL.4, Y59H11AL.1, Y59H11AR.2, Y59H11AR.4, Y60C6A.1, Y61A9LA.1, Y64H9A.1, Y65B4BL.3, Y65B4BL.4, Y65B4BL.7, Y67D8C.4, Y67D8C.6, Y67D8C.9, Y69A2AR.6, Y69A2AR.9, Y69A2AR.14, Y69A2AR.15, Y69A2AR.19, Y69A2AR.23, Y69A2AR.27, Y69A2AR.31, Y71D11A.1, Y71D11A.5, Y71F9AL.6, Y71F9AL.7, Y71F9AM.6, Y71F9B.1, Y71F9B.3, Y71F9B.8, Y71F9B.14, Y71G10AR.1, Y71G12A.4, Y71G12B.3, Y71G12B.7, Y71G12B.16, Y71G12B.23, Y71G12B.25, Y71G12B.26, Y71H2AL.2, Y71H2AM.2, Y71H2AM.4, Y71H2AM.9, Y71H2AM.10, Y71H2AR.2, Y73B3A.1, Y73B6BL.14, Y73B6BL.19, Y73B6BL.22, Y73B6BL.26, Y73B6BL.31, Y73B6BL.36, Y73C8C.9, Y73E7A.3, Y73E7A.6, Y74C10AL.2, Y74C10AR.3, Y74E4A.1, Y75B7AL.1, Y75B7AL.2, Y76B12C.1, Y76G2A.2, Y77E11A.12, Y81B9A.3, Y82E9BL.1, Y82E9BL.2, Y82E9BL.3, Y82E9BL.5, Y82E9BL.6, Y82E9BR.3, Y82E9BR.16, Y92H12BR.3, Y94H6A.5, Y95B8A.4, Y97E10AL.1, Y97E10AR.2, Y97E10AR.6, Y97E10B.2, Y97E10B.3, Y97E10B.4, Y97E10B.9, Y97E10B.10, Y102A11A.1, Y102A11A.6, Y102A11A.7, Y102A11A.8, Y108G3AL.2, Y110A2AL.12, Y110A2AR.1, Y110A7A.11, Y119C1B.1, Y119C1B.3, Y119C1B.5, Y119D3B.3, Y119D3B.5, ZC8.1, ZC13.1, ZC13.2, ZC21.6, ZC53.4, ZC132.3, ZC132.8, ZC132.9, ZC142.1, ZC142.2, ZC155.4, ZC155.7, ZC190.2, ZC190.5, ZC190.6, ZC190.8, ZC196.5, ZC196.8, ZC196.9, ZC239.5, ZC239.16, ZC239.17, ZC250.3, ZC262.3, ZC266.1, ZC266.2, ZC317.2, ZC328.1, ZC328.3, ZC404.10, ZC404.11, ZC404.13, ZC449.4, ZC449.5, ZC477.3, ZC487.2, ZC513.3, ZC513.5, ZC581.3, ZK6.6, ZK6.8, ZK40.1, ZK54.1, ZK54.3, ZK84.2, ZK84.4, ZK105.4, ZK154.6, ZK180.1, ZK180.3, ZK185.2, ZK353.2, ZK353.4, ZK355.2, ZK370.4, ZK370.7, ZK370.8, ZK402.1, ZK418.3, ZK418.5, ZK418.6, ZK418.7, ZK470.1, ZK484.5, ZK546.4, ZK563.2, ZK563.6, ZK616.6, ZK682.2, ZK682.5, ZK686.3, ZK688.2, ZK688.7, ZK697.1, ZK697.3, ZK697.7, ZK721.4, ZK770.1, ZK783.1, ZK813.5, ZK1055.3, ZK1055.4, ZK1236.7, ZK1290.13, F08A10.2, R01H2.7, F53F4.16, C26D10.7, C53D6.10, C09G12.10, C09G12.11, C09G12.16, F56D6.7, C17F4.10, C17G1.8, Y57G7A.12, F11E6.11, F17C11.13, F14F8.13, T09F5.16, C35D6.9, C35D6.10, Y7A9C.7, F08H9.12, Y7A9C.9, Y7A9C.8, K03D3.11, W05H5.8, T11A5.7, R144.12, Y54G2A.38, Y59E9AL.7, Y73B6BL.42, F20D6.12, F11D5.7, B0403.6, C07A9.12, Y79H2A.12, T21C12.8, F40G12.15, C24A8.6, C08B6.13, ZK856.14, R02E4.2, W02B12.15, F27E5.8, F49C5.9, Y38E10A.28, R11G10.3, Y57A10C.11, F57G9.7, C41C4.10, T14D7.3, T28F3.3, D2021.2, W02B8.6, Y54G9A.10, C45H4.18, C02A12.8, C02A12.9, C02A12.10, R09E12.8, F30B5.8, ZK265.9, Y19D10A.15, F20E11.15, F54B8.16, C31A11.10, F58G6.9, F47B8.12, C06B3.12, C06B3.13, C06B3.14, F26G5.12, C36B1.13, C35C5.11, T28B8.6, C39H7.8, C39H7.9, C23H4.8, F23D12.8, F42D1.4, H03G16.6, M163.9, M163.10, C12D8.18, C12D8.19, C30G7.2, C30G7.5, F43D2.6, F57B1.8, Y6G8.6, Y51A2D.21, Y102A5C.36, F41G3.18, Y56A3A.36, C53D6.11, F32A7.8, F36H2.4, F38E11.13, M117.6, Y45F10C.6, Y45F10D.16, Y87G2A.19, Y50E8A.17, W02A2.9, C32C4.7, B0035.18, C09E7.10, C25B8.8, D1022.9, D2062.12, F52B10.3, K04C2.7, C33H5.19, T20B3.15, ZK381.8, Y71G12B.33, C33C12.11, K10G6.5, C25H3.16, F02A9.7, R12B2.8, F40H6.6, C06A5.12, C08F1.11, C08G5.7, F53F10.8, T01D1.7, W10C8.6, Y18H1A.15, Y47G6A.31, Y47G6A.32, C06G4.6, ZK688.10, F23F12.13, C18H7.10, C18H7.11, Y55F3C.9, Y55F3C.10, Y18H1A.14, W09G12.10, Y77E11A.16, R05C11.4, F56D6.10, F56D6.11, F56D6.12, F56D6.13, F56D6.14, F49F1.14, ZK185.4, ZK185.5, F29B9.12, C09B9.8, W02H5.10, Y39H10B.2, Y45G5AL.2, D2063.4, H23N18.6, F14F9.8, C13A2.12, R13D7.11, ZK105.8, F38G1.3, ZC13.10, C36C9.6, C14E2.7, T26C11.8, F23G4.1, T03G11.9, K03D3.12, C31B8.16, T27A3.8, R03H4.6, ZK678.8, F55A11.5, F54H12.7, Y47D7A.16, Y51H7BR.8, C23H5.11, Y71G10AR.4, T13F3.9, F33G12.7, B0303.16, T24C4.8, C36E6.8, K09F5.6, AH9.6, F22G12.8, Y19D10A.17, C50E3.16, C35B8.4, Y119C1B.12, F58F12.4, Y71H2AM.25, ZK688.11, C53C11.5, T23E7.5, T23E7.6, ZK1290.15, K09C6.10, F18E9.8, F13H10.8, F47E1.5, ZK1053.7, F59C6.14, ZC262.9, ZC262.10, C02G6.3, T10D4.14, M01D7.9, R09B5.13, F40F8.12, Y43C5A.7, K07A1.17, K12C11.6, K12C11.7, Y71A12B.17, F07C6.6, K03D3.14, R01H2.8, F13E9.16, Y110A7A.21, Y26D4A.17, Y49E10.29, C29E4.15, F54H12.8, ZK180.7, Y73B6BL.47, Y27F2A.10, Y27F2A.11, T03E6.9, W06G6.15, C15A7.4, K09H9.8, F09F9.5, Y54G2A.52, K10H10.12, C25F9.13, Y37H2A.14, F15B9.10, C10A4.9, F08A8.8, T03F6.9, F57B1.9, D1081.11, ZK39.10, T04C12.9, T12B5.14, F55F10.3, C14A6.12, C14A6.13, C10A4.10, K06B4.15, Y70C5B.2, M03A1.8, F09C6.16, T27F6.10, F07B10.7, C37A5.11, C23H5.12, C41G7.8, C25A1.16, F40G9.15, F38B7.10, F27D4.8, F21C3.7, ZC443.7, F53F4.18, T26H8.5, T20F5.8, Y26G10.4, M106.8, K11E4.6, Y44A6D.7, F58E6.12, C45B11.8, K04G2.12, M04D5.3, C41G7.9, K07A12.8, K01A2.12, Y2H9A.6, F58H1.8, C45B11.9, F16H6.11, F38B7.11, F40G9.18, Y34F4.6, M03F8.7, C18H7.12, C49C3.21, F47G3.4, C25H3.17, C48D1.9, Y105C5A.1269, Y105C5A.1270, F36H2.6, K07E3.9, Y116A8C.463, ZK1098.12, F59A3.13, B0025.5, Y23H5B.12, Y36E3A.2, C30B5.9, T11F9.21, T25E12.16, ZK783.7, Y39A1A.27, Y41C4A.21, F37C12.21, C12D8.20, K08F9.5, K08F9.6, Y70C5A.4, C18H7.13, T27B2.1, M03C11.9

**serpentine receptors (1469)**

AC3.1, AH6.10, AH6.11, AH6.12, AH6.14, AH6.4, AH6.6, AH6.7, AH6.8, B0205.2, B0212.2, B0213.7, B0213.8, B0213.9, B0238.3, B0238.5, B0238.6, B0238.8, B0244.6, B0250.10, B0250.6, B0304.5, B0304.6, B0304.7, B0304.8, B0304.9, B0334.7, B0391.12, B0391.3, B0391.4, B0414.1, B0454.10, B0454.2, B0454.3, B0454.4, B0495.1, B0496.5, B0507.11, B0547.4, C01B4.1, C01B4.10, C01B4.3, C01B4.5, C01G10.2, C01G10.3, C02A12.10, C02A12.2, C02A12.3, C02A12.5, C02A12.6, C02A12.9, C02E7.13, C02E7.2, C02E7.3, C02E7.4, C02E7.5, C02E7.9, C03A7.10, C03A7.3, C03A7.5, C03A7.6, C03A7.9, C03G6.11, C03G6.16, C03G6.2, C03G6.3, C03G6.7, C03G6.9, C03H5.7, C04C3.1, C04C3.7, C04E12.8, C04E12.9, C04E6.10, C04E6.2, C04E6.9, C04F2.1, C04F2.4, C04F5.4, C04F5.5, C04F5.6, C05E4.10, C05E4.11, C05E4.13, C05E4.14, C05E4.2, C05E4.4, C05E4.6, C06A8.7, C06B3.1, C06B3.10, C06B3.11, C06B3.12, C06B3.13, C06B3.14, C06B3.9, C06B8.10, C06B8.4, C06B8.6, C06B8.9, C06C3.6, C06C6.1, C06C6.2, C06C6.3, C06E7.7, C06G8.4, C07D10.3, C07G3.3, C07G3.4, C07G3.5, C07G3.6, C08B6.12, C08B6.13, C08F11.4, C08F11.9, C09E7.1, C09G12.10, C09G12.11, C09G12.16, C09G12.2, C09G12.3, C09G12.6, C09H5.3, C09H5.4, C09H5.5, C09H5.6, C09H5.9, C10G11.2, C10G11.3, C10G11.4, C10G8.1, C12D5.11, C12D8.12, C13B7.1, C13B7.2, C13B7.3, C13B7.4, C13B7.5, C13D9.1, C13D9.2, C13D9.3, C13D9.4, C13D9.5, C13D9.6, C14A4.15, C14C11.5, C14C6.1, C14C6.10, C14C6.9, C14H10.4, C15H9.3, C16D9.7, C17B7.1, C17E7.11, C17E7.2, C17E7.3, C17F4.10, C17F4.4, C18B10.1, C18B10.2, C18B10.3, C18B10.4, C18B10.5, C18B10.7, C18B10.8, C18D4.5, C18D4.9, C18F10.4, C18F10.5, C18F10.6, C18F10.8, C18H7.8, C24A8.6, C24B9.1, C24B9.10, C24B9.11, C24B9.12, C24B9.14, C24B9.15, C24B9.16, C24B9.2, C24B9.4, C24B9.5, C24B9.6, C24B9.7, C24B9.8, C24H11.4, C25E10.3, C25F9.1, C25F9.13, C27A7.7, C27D6.10, C27D6.6, C27D6.8, C27D6.9, C28C12.13, C29F3.6, C29F9.8, C29G2.4, C29G2.5, C30B5.7, C30G7.5, C31A11.10, C31A11.3, C31A11.4, C31A11.6, C31A11.9, C31B8.11, C31B8.13, C31B8.6, C31E10.1, C32B5.2, C32H11.2, C33A12.10, C33A12.11, C33A12.13, C33A12.14, C33A12.8, C33D9.4, C33G8.1, C33G8.5, C34C6.1, C34D4.8, C35A11.1, C35A5.7, C35D6.1, C35D6.10, C35D6.2, C35D6.9, C36C5.10, C36C5.11, C36C5.2, C36C5.3, C36C5.6, C36C5.7, C36C5.8, C36C5.9, C38C3.1, C38C3.2, C38C6.4, C39B5.1, C39B5.11, C39B5.12, C39H7.5, C39H7.6, C39H7.7, C39H7.8, C39H7.9, C41C4.2, C41G6.10, C41G6.11, C41G6.12, C41G6.14, C41G6.15, C41G6.16, C41G6.2, C41G6.3, C41G6.7, C41G6.8, C41G6.9, C42C1.1, C42D4.4, C42D4.5, C42D4.9, C43D7.6, C44B12.4, C44B12.8, C44C3.1, C44C3.11, C44C3.2, C44C3.3, C44C3.5, C44C3.6, C44C3.7, C44C3.9, C45B11.4, C45H4.1, C45H4.10, C45H4.11, C45H4.12, C45H4.15, C45H4.16, C45H4.18, C45H4.3, C45H4.6, C45H4.7, C45H4.8, C45H4.9, C46E10.10, C46E10.6, C46E10.7, C46F4.1, C47A10.10, C47A10.11, C47A10.2, C47A10.3, C47A10.4, C47A10.6, C47A10.7, C47A10.8, C47A10.9, C47E8.2, C48B6.5, C49D10.3, C49G7.2, C50B6.10, C50B6.12, C50B6.5, C50B6.6, C50C10.1, C50C10.2, C50C10.3, C50C10.4, C50C10.6, C50C10.7, C50C10.8, C50E10.10, C50E10.11, C50E10.3, C50E10.5, C50E10.6, C50E10.7, C50E10.8, C50E10.9, C50H11.10, C50H11.11, C50H11.12, C50H11.14, C50H11.2, C50H11.3, C50H11.4, C50H11.5, C50H11.7, C50H11.9, C51E3.1, C51E3.2, C51E3.3, C51E3.4, C51E3.5, C51F7.2, C52B9.5, C53A5.10, C53A5.8, C53B7.5, C54C6.4, C54D10.6, C54E10.4, C54F6.1, C54F6.10, C54F6.11, C54F6.7, C55A1.1, C55A1.12, C55A1.14, C55A1.15, C55A1.3, C55A1.5, C55A1.8, C56C10.5, D1054.12, D1065.4, D1065.5, D1069.4, D2023.1, D2062.2, D2062.3, D2062.8, D2062.9, DC2.1, DC2.2, DC2.6, E02C12.2, E02C12.3, E03D2.3, E03D2.4, E03H12.1, E03H12.6, E04F6.1, E04F6.13, E04F6.14, F01D4.7, F07B10.2, F07B10.3, F07B10.4, F07B10.6, F07B10.7, F07C3.8, F07C4.1, F07C4.13, F07C4.14, F07C4.3, F07C4.4, F07C4.5, F07C4.8, F07F6.9, F07G11.5, F07G11.8, F08A10.2, F08E10.1, F08E10.2, F08E10.3, F08E10.6, F08H9.12, F09C12.6, F09C6.16, F09C6.7, F09E5.4, F09F3.1, F09F3.11, F09F3.12, F09F3.13, F09F3.2, F09F3.4, F09F3.7, F09G2.7, F10A3.12, F10A3.13, F10A3.15, F10A3.16, F10A3.5, F10A3.6, F10A3.7, F10A3.8, F10A3.9, F10D2.1, F10D2.4, F10G2.6, F10G2.8, F10G7.7, F11A5.1, F11A5.2, F13A7.13, F13A7.2, F13A7.3, F13A7.8, F13G3.2, F14F8.1, F14F8.10, F14F8.11, F14F8.12, F14F8.13, F14F8.3, F14F8.4, F14F8.5, F14F8.6, F14F8.7, F14F9.1, F14F9.7, F14H3.1, F15A2.3, F15A2.4, F15A4.1, F15A4.3, F15A4.4, F15A4.7, F15E11.10, F15E6.7, F15H9.2, F15H9.3, F15H9.4, F16B4.10, F17A2.10, F17A2.11, F17A2.12, F17A2.6, F17A2.7, F17A2.8, F17A2.9, F18C5.1, F18C5.6, F18C5.8, F18E2.4, F18E3.1, F18E3.10, F18E3.2, F18E3.4, F18E3.5, F18E3.6, F19B10.7, F19B10.8, F19B2.3, F19B2.8, F19G12.5, F20A1.3, F20D6.12, F20E11.1, F20E11.10, F20E11.12, F20E11.15, F20E11.2, F20E11.4, F20E11.6, F21A3.1, F21F8.1, F21F8.10, F21F8.9, F21H7.11, F21H7.14, F21H7.7, F22B8.1, F22B8.3, F22B8.5, F22E5.16, F22E5.4, F23F12.10, F25E2.1, F25E5.12, F25E5.13, F25E5.14, F26B1.6, F26D10.8, F26D2.1, F26D2.11, F26D2.4, F26D2.7, F26D2.9, F26F12.6, F26G5.10, F26G5.12, F26G5.2, F26G5.4, F26G5.5, F27E5.5, F28B1.6, F28B1.8, F28C12.1, F28C12.2, F28C12.4, F28C12.5, F28C12.7, F28D9.2, F28H7.1, F28H7.11, F28H7.9, F30B5.6, F30B5.8, F31E9.2, F31E9.5, F31F4.13, F31F4.14, F31F4.16, F31F4.18, F31F4.2, F31F4.3, F31F4.4, F31F4.6, F31F4.8, F31F4.9, F32A7.7, F32G8.1, F32H5.5, F32H5.6, F33H1.5, F33H12.2, F33H12.4, F33H12.5, F34D6.5, F34D6.6, F35B12.1, F35C5.2, F35F10.2, F35F10.8, F35F10.9, F36D1.2, F36D1.3, F36D3.13, F36D3.3, F36D3.6, F36G9.1, F36G9.16, F36G9.2, F36G9.5, F36G9.6, F36G9.8, F36G9.9, F36H9.6, F37B4.1, F37B4.11, F37B4.12, F37B4.13, F37B4.3, F37B4.4, F37B4.5, F37B4.6, F37B4.8, F37B4.9, F37C12.15, F37C12.16, F37C12.17, F38B7.4, F38B7.7, F38B7.8, F38E1.6, F38E1.8, F38H12.1, F38H12.2, F38H12.5, F39E9.3, F40A3.7, F40D4.1, F40D4.11, F40D4.3, F40D4.5, F40D4.6, F40D4.7, F40D4.8, F40D4.9, F40F9.4, F40G12.1, F40G12.15, F40G12.8, F40H7.2, F40H7.4, F40H7.5, F40H7.7, F40H7.8, F41B5.8, F41F3.6, F41F3.7, F41G3.11, F41H8.1, F41H8.2, F41H8.4, F42D1.3, F43A11.1, F43A11.3, F43A11.4, F43A11.6, F44F4.13, F44F4.5, F44F4.7, F44G3.1, F44G3.11, F44G3.5, F46B3.11, F46B6.11, F47B8.12, F47B8.7, F47B8.9, F47C12.10, F47C12.3, F47C12.5, F47D2.1, F47D2.10, F47D2.3, F47D2.4, F47D2.5, F47D2.6, F47D2.7, F47D2.8, F47D2.9, F47G9.2, F48D6.2, F48F5.4, F48G7.1, F48G7.6, F49A5.8, F49C5.1, F49C5.2, F49C5.6, F49C5.9, F49E12.5, F49H6.11, F49H6.4, F52D2.9, F52F10.5, F53B2.4, F53F1.10, F53F1.11, F53F1.7, F53F1.8, F53F1.9, F53F4.9, F53F8.2, F54B8.10, F54B8.11, F54B8.12, F54B8.16, F54B8.6, F54B8.7, F54B8.8, F54B8.9, F54E2.6, F54E4.2, F54F11.3, F55B12.6, F55B12.7, F55B12.8, F55B12.9, F55C5.9, F56A4.7, F56D5.10, F56D6.4, F56D6.5, F56D6.7, F56H9.1, F57A10.1, F57A8.3, F57B7.1, F57E7.3, F57G8.1, F57G8.3, F57G8.4, F57G8.8, F57G9.1, F57G9.2, F57G9.4, F57G9.7, F58A6.10, F58A6.11, F58A6.6, F58D7.1, F58E10.6, F58E2.9, F58G4.2, F58G4.5, F58G4.6, F58G4.7, F58G6.2, F59A1.14, F59A1.3, F59A1.4, F59A7.3, F59B1.1, F59B1.3, F59B1.4, F59B1.7, F59D6.5, F59E11.1, F59E11.13, F59E11.14, F59E11.15, F59E11.16, H04J21.2, H04M03.6, H04M03.8, H04M03.9, H05B21.2, H05B21.3, H05B21.4, H06H21.1, H06H21.2, H10D18.3, H12C20.5, H12I19.1, H12I19.2, H24D24.1, H24D24.2, H24O09.1, H25K10.3, H25K10.7, H27D07.2, H27D07.3, H27D07.4, H27D07.5, H27D07.6, H34P18.1, K01A12.3, K01B6.2, K02A2.2, K02E11.2, K02E2.3, K02H11.2, K02H11.3, K02H11.7, K03B4.5, K03D3.1, K03D3.11, K03D3.12, K03D3.14, K03D3.4, K03D7.11, K03D7.2, K03D7.4, K03D7.6, K04C1.1, K04C1.6, K04F1.16, K04F1.2, K04F1.3, K04F1.4, K04F1.5, K05D4.2, K05D4.3, K05D4.6, K05D4.8, K05D4.9, K06B4.9, K06C4.17, K07C6.10, K07C6.11, K07C6.6, K07C6.7, K07C6.8, K07C6.9, K07E8.11, K07E8.5, K07E8.9, K08B5.1, K08D9.1, K08G2.12, K08G2.13, K08G2.5, K08G2.7, K08G2.8, K08G2.9, K09C6.4, K09C6.5, K09D9.10, K09D9.13, K09D9.6, K09D9.7, K09D9.8, K10B4.2, K10B4.5, K10C9.4, K10C9.6, K10C9.8, K10G4.2, K10G4.9, K11D12.3, K11E4.6, K12B6.5, K12D9.10, K12D9.3, K12D9.4, K12D9.5, K12D9.7, K12D9.9, K12G11.5, M01B2.11, M01B2.2, M01B2.3, M01B2.4, M01B2.7, M01B2.9, M01D1.1, M01G12.1, M01G12.13, M01G12.4, M01G12.6, M02H5.10, M02H5.11, M02H5.12, M02H5.2, M02H5.9, M03F8.7, M162.3, M199.1, M7.13, R02C2.5, R03G8.5, R03H4.2, R03H4.3, R03H4.4, R03H4.9, R04B5.8, R04D3.10, R04D3.12, R04D3.6, R04D3.7, R04D3.8, R04D3.9, R05D8.2, R05D8.3, R05D8.4, R05D8.5, R05D8.6, R05F9.7, R05H5.1, R05H5.6, R07B5.3, R07B5.4, R07B5.5, R07B5.6, R07B5.7, R08C7.7, R08F11.2, R08F11.5, R08H2.13, R08H2.2, R08H2.3, R08H2.4, R08H2.5, R08H2.7, R09B5.7, R09E12.1, R09E12.2, R09E12.4, R09E12.8, R09F10.6, R10D12.11, R10D12.17, R10D12.4, R10E11.7, R10E8.5, R10H1.2, R11D1.5, R11D1.6, R11G10.3, R11G11.13, R11G11.5, R11G11.9, R13D11.1, R13D11.3, R13D11.6, R13D11.9, R13D7.1, R13D7.10, R13D7.3, R13D7.4, R13D7.6, R13F6.3, R13H4.7, R13H7.1, R186.2, R52.7, T01C4.3, T01C4.4, T01C4.5, T01C4.6, T01D3.4, T01E8.7, T01G5.3, T01G5.4, T01G5.5, T01G5.6, T01G6.3, T01G6.9, T02B11.1, T02B11.5, T02B5.4, T02D1.3, T03D3.11, T03D3.12, T03D3.14, T03D3.2, T03D3.3, T03D3.4, T03D3.6, T03E6.1, T03E6.2, T03E6.4, T03E6.5, T03E6.6, T03E6.8, T03E6.9, T03F7.2, T03F7.3, T03F7.4, T03G11.2, T04A11.10, T04A11.12, T04A11.7, T04A11.8, T04A11.9, T04A8.1, T04A8.2, T04B2.4, T04C12.2, T05A12.1, T05A7.8, T05B11.2, T05B11.6, T05B4.5, T05B4.6, T05B4.7, T05C3.7, T05C3.8, T05E12.1, T05E12.2, T05E12.4, T05G11.2, T05G11.3, T05G11.6, T05G11.7, T06A1.2, T06C10.2, T06C12.1, T06C12.11, T06C12.2, T06C12.3, T06E4.7, T06E6.11, T06E6.3, T06E6.4, T06E6.6, T06E6.7, T06E6.8, T06E6.9, T06G6.1, T06G6.2, T06G6.7, T07C12.1, T07C12.6, T07H8.1, T07H8.3, T07H8.5, T07H8.7, T08B6.3, T08B6.6, T08B6.7, T08G3.1, T08G3.10, T08G3.12, T08G3.2, T08G3.3, T08G3.5, T08G3.8, T09D3.1, T09D3.2, T09D3.5, T09D3.6, T09D3.7, T09E8.5, T09F5.16, T09F5.3, T09F5.5, T09F5.8, T10C6.1, T10C6.2, T10C6.3, T10C6.4, T10D4.10, T10D4.12, T10D4.14, T10D4.5, T10D4.8, T10D4.9, T10E10.3, T10G3.4, T10H4.2, T10H4.3, T10H4.5, T10H4.6, T10H4.8, T10H4.9, T10H9.1, T10H9.6, T11A5.2, T11A5.3, T11A5.7, T11F1.1, T11F1.5, T11F9.18, T12A2.10, T12A2.11, T12A2.12, T12A2.13, T12A2.9, T12A7.7, T13A10.12, T13A10.13, T13A10.14, T13A10.6, T13A10.7, T13A10.9, T13F2.5, T13F3.1, T14C1.1, T15B7.11, T15B7.12, T15B7.13, T16A9.2, T16H12.8, T18H9.4, T19B10.10, T19C4.2, T19C4.3, T19C4.4, T19C4.8, T19C4.9, T19C9.1, T19C9.2, T19C9.3, T19C9.4, T19D12.8, T19E7.5, T19H12.4, T19H12.5, T19H12.7, T20B3.3, T20B3.4, T20B3.5, T20C4.1, T20D4.1, T20D4.18, T20D4.2, T21B4.1, T21B4.12, T21B4.14, T21B4.5, T21B4.6, T21B4.7, T21B4.8, T21B4.9, T21C9.7, T21D12.5, T21H8.2, T21H8.3, T21H8.4, T22B7.5, T22F3.5, T22F3.6, T22G5.4, T22H2.1, T22H2.3, T22H6.3, T22H6.4, T23D5.1, T23D5.10, T23D5.11, T23D5.12, T23D5.2, T23D5.6, T23D5.7, T23D5.8, T23D5.9, T23F1.3, T23F1.4, T23F11.5, T24A6.10, T24A6.13, T24A6.14, T24A6.4, T24A6.5, T24A6.6, T24E12.4, T24E12.8, T25E12.11, T25E12.13, T26E3.9, T26E4.12, T26E4.14, T26E4.15, T26H2.6, T26H2.8, T26H5.3, T26H5.5, T26H8.2, T26H8.4, T26H8.5, T27A1.7, T27C10.1, T27C10.2, T27C4.3, T27C5.1, T27C5.10, T27C5.2, T27C5.5, T27D1.3, T27E7.4, T27E7.5, T27E7.8, T27E7.9, T28A11.1, T28A11.10, T28A11.12, T28A11.15, T28A11.7, T28A11.9, T28H11.2, T28H11.3, VC27A7L.1, W01D2.4, W02F12.7, W02H5.6, W03D2.10, W03F9.6, W04D2.2, W05B10.5, W05E7.2, W05H5.1, W05H5.4, W05H5.5, W05H5.6, W05H5.7, W05H5.8, W06D12.4, W06D12.7, W06G6.13, W06G6.3, W06G6.6, W06G6.8, W06H8.7, W07A8.1, W07A8.5, W07G1.2, W07G1.6, W07G4.6, W08G11.5, W09D12.2, W09D12.3, W09D6.2, W09D6.3, W10G11.10, W10G11.9, Y102A5C.15, Y102A5C.21, Y102A5C.22, Y102A5C.23, Y102A5C.24, Y102A5C.25, Y102A5C.28, Y102A5C.29, Y102A5C.31, Y102A5C.32, Y102A5C.33, Y105C5A.11, Y105C5B.1, Y105C5B.10, Y105C5B.4, Y105C5B.6, Y113G7A.1, Y113G7B.2, Y113G7B.9, Y116A8C.40, Y116F11B.5, Y119D3B.3, Y119D3B.5, Y17G9A.1, Y17G9A.5, Y17G9A.6, Y17G9A.7, Y19D10A.1, Y19D10A.13, Y19D10A.15, Y19D10A.17, Y19D10A.2, Y22D7AR.8, Y22F5A.2, Y25C1A.10, Y25C1A.11, Y25C1A.12, Y25C1A.9, Y26G10.1, Y26G10.2, Y26G10.4, Y27F2A.10, Y27F2A.11, Y27F2A.2, Y27F2A.3, Y27F2A.4, Y27F2A.7, Y2H9A.2, Y32B12B.3, Y32B12B.5, Y32B12B.7, Y32B12C.2, Y32H12A.1, Y37A1B.10, Y37H2C.4, Y38A10A.1, Y38A10A.3, Y38C9B.2, Y38H6C.12, Y38H6C.2, Y39A3B.4, Y39C12A.5, Y39C12A.6, Y39C12A.7, Y39G10AR.22, Y39G8B.3, Y39G8B.4, Y39H10B.1, Y40B10A.3, Y40B10B.2, Y40D12A.3, Y40H7A.1, Y40H7A.5, Y40H7A.6, Y40H7A.8, Y40H7A.9, Y41E3.12, Y41E3.14, Y41E3.15, Y43B11AL.2, Y43B11AR.2, Y43B11AR.5, Y43F8A.4, Y43F8C.19, Y44A6B.1, Y44A6B.2, Y45F10B.11, Y45F10B.14, Y45F10B.4, Y45F10B.5, Y45F10B.6, Y45G12A.1, Y45G12C.10, Y45G12C.12, Y45G12C.14, Y45G12C.15, Y45G12C.5, Y45G12C.6, Y45G12C.7, Y45G12C.8, Y45G12C.9, Y46H3A.1, Y46H3C.1, Y46H3C.2, Y46H3C.3, Y46H3D.2, Y46H3D.3, Y47G7B.1, Y47G7B.3, Y48A6B.1, Y48C3A.11, Y49C4A.1, Y49C4A.2, Y49C4A.3, Y49C4A.4, Y49C4A.5, Y49C4A.6, Y49F6A.2, Y49F6A.3, Y49F6B.11, Y51A2D.12, Y52E8A.5, Y54G11A.12, Y54G11A.15, Y54G11B.1, Y54G2A.38, Y54G9A.10, Y55F3AM.2, Y55F3C.10, Y55F3C.2, Y55F3C.8, Y57A10B.4, Y57A10B.5, Y57A10C.3, Y57A10C.4, Y57A10C.7, Y57A10C.8, Y57G11C.46, Y57G7A.12, Y57G7A.4, Y57G7A.7, Y59A8A.4, Y59A8B.3, Y59A8B.4, Y5H2B.4, Y5H2B.7, Y60A3A.22, Y60A3A.3, Y60A3A.4, Y60A3A.5, Y60A3A.6, Y61B8A.1, Y61B8A.2, Y61B8B.1, Y62E10A.4, Y62H9A.10, Y64G10A.8, Y68A4A.2, Y68A4A.3, Y68A4A.6, Y68A4A.7, Y68A4A.9, Y69A2AR.15, Y69E1A.6, Y6E2A.1, Y6E2A.2, Y6E2A.6, Y6G8.4, Y70C5A.2, Y70C5C.4, Y73B6BL.10, Y73B6BL.11, Y73B6BL.39, Y73B6BL.40, Y73B6BL.41, Y73C8A.1, Y73C8C.11, Y73C8C.5, Y73C8C.6, Y73C8C.9, Y73F8A.3, Y75B12B.10, Y75B12B.7, Y77E11A.16, Y7A9C.7, Y7A9C.8, Y7A9C.9, Y94A7B.1, Y94A7B.3, Y94A7B.4, Y94A7B.5, Y94A7B.6, Y94A7B.7, Y94A7B.8, Y94A7B.9, Y97E10B.10, Y97E10B.2, Y97E10B.3, Y97E10B.4, Y97E10B.9, Y9C9A.10, Y9C9A.11, Y9C9A.15, Y9C9A.17, Y9C9A.18, Y9C9A.2, Y9C9A.3, Y9C9A.4, Y9C9A.5, Y9C9A.6, Y9C9A.7, Y9C9A.8, Y9C9A.9, ZC132.3, ZC132.7, ZC142.1, ZC142.2, ZC204.15, ZC204.4, ZC204.5, ZC239.10, ZC239.19, ZC239.8, ZC239.9, ZC317.4, ZC317.5, ZC404.10, ZC404.11, ZC404.12, ZC404.13, ZC404.5, ZC443.7, ZC455.11, ZC455.7, ZC455.8, ZC455.9, ZC482.6, ZC482.8, ZC513.11, ZC513.9, ZK1037.11, ZK1037.3, ZK1037.8, ZK1037.9, ZK105.2, ZK105.4, ZK105.8, ZK228.5, ZK228.6, ZK228.7, ZK228.8, ZK262.1, ZK262.10, ZK262.11, ZK262.6, ZK262.7, ZK265.5, ZK285.1, ZK488.9, ZK6.9, ZK678.4, ZK678.6, ZK697.10, ZK697.11, ZK697.12, ZK697.13, ZK697.4, ZK697.5, ZK697.7, ZK721.4, ZK829.8, ZK863.5

**TM proteins with additional domains (2680)**

AC3.10, AC3.2, AC3.5, AC3.7, AC3.8, AC7.1, AH6.1, AH6.2, AH9.1, B0024.13, B0024.14, B0024.3, B0024.6, B0034.3, B0035.18, B0035.2, B0198.1, B0198.3, B0207.12, B0212.1, B0212.4, B0212.5, B0213.2, B0218.1, B0218.2, B0222.1, B0222.10, B0222.2, B0222.3, B0240.1, B0240.2, B0240.3, B0240.4, B0244.10, B0244.2, B0244.5, B0244.7, B0244.8, B0244.9, B0250.9, B0252.1, B0252.3, B0272.2, B0273.4, B0280.12, B0284.3, B0285.6, B0286.2, B0303.11, B0303.4, B0304.3, B0310.5, B0331.2, B0334.11, B0334.2, B0334.5, B0334.6, B0336.11, B0348.1, B0348.2, B0361.11, B0361.7, B0361.8, B0365.3, B0393.5, B0395.1, B0395.2, B0399.1, B0399.2, B0410.2, B0416.1, B0416.5, B0454.6, B0457.1, B0464.3, B0464.4, B0464.6, B0491.1, B0491.4, B0491.8, B0495.4, B0495.7, B0511.12, B0511.13, B0511.2, B0513.9, B0546.5, B0554.5, B0554.7, B0563.2, B0563.4, B0563.6, B0564.3, B0564.4, BE10.2, C01A2.3, C01B10.10, C01B10.4, C01B10.7, C01B10.9, C01B12.3, C01B12.5, C01B4.7, C01B4.8, C01B4.9, C01C10.1, C01C10.3, C01C10.4, C01F1.2, C01F1.4, C01G10.1, C01G10.12, C01G12.8, C01G5.4, C01G6.1, C01G6.8, C01G8.2, C01G8.4, C01H6.6, C02A12.8, C02B8.5, C02C2.3, C02C2.4, C02C2.5, C02C6.2, C02D4.2, C02D5.2, C02E11.1, C02E7.1, C02F12.1, C02F5.11, C02F5.13, C02F5.8, C02H6.1, C02H7.2, C03A3.2, C03A7.11, C03B1.12, C03B1.13, C03B8.1, C03C10.3, C03F11.1, C03F11.3, C03G6.13, C03H5.2, C03H5.6, C04A11.4, C04A2.7, C04C3.2, C04E12.4, C04E12.5, C04F12.10, C04F5.1, C04F5.7, C04F6.5, C04H5.3, C05A9.1, C05C12.3, C05D10.3, C05D11.7, C05D12.1, C05D2.1, C05D9.2, C05E11.1, C05E11.4, C05E11.5, C05G5.1, C06A12.4, C06A8.9, C06B3.2, C06B8.7, C06C6.7, C06E1.3, C06E1.4, C06G3.4, C06G4.5, C06G8.1, C06G8.2, C06G8.3, C06H2.4, C06H5.6, C07A9.11, C07A9.12, C07A9.4, C07A9.8, C07F11.1, C07G3.9, C07H4.2, C08B11.4, C08B11.8, C08B6.1, C08D8.1, C08E8.1, C08F11.8, C08F8.4, C08G5.1, C08G9.2, C08H9.10, C08H9.11, C08H9.12, C08H9.13, C08H9.3, C08H9.4, C08H9.5, C08H9.7, C09B7.1, C09B9.3, C09B9.8, C09D4.1, C09E8.3, C09F12.1, C09F5.2, C09F9.2, C09G4.1, C09G5.1, C09H5.2, C10C6.2, C10C6.5, C10C6.6, C10E2.6, C10F3.2, C10F3.3, C10G8.5, C10H11.3, C10H11.4, C10H11.5, C10H11.6, C11D2.6, C11E4.3, C12D12.2, C12D8.14, C12D8.16, C12D8.17, C12D8.18, C12D8.19, C12D8.5, C12D8.6, C13A2.1, C13A2.5, C13A2.9, C13B4.1, C13B9.4, C13C4.5, C13C4.6, C13D9.7, C13D9.8, C13D9.9, C13F10.5, C13G3.2, C14A4.3, C14A6.2, C14B9.3, C14C10.1, C14C11.4, C14F11.3, C14F5.1, C14F5.4, C14H10.1, C15A11.7, C15A7.1, C15A7.2, C15A7.4, C15B12.5, C15B12.7, C15C7.1, C15F1.3, C15F1.6, C15H11.2, C15H11.4, C15H9.1, C15H9.4, C15H9.5, C16B8.1, C16B8.4, C16C10.1, C16C10.12, C16C10.5, C16C10.7, C16C4.13, C16C4.6, C16C8.17, C16D6.2, C16D9.2, C16D9.4, C16E9.4, C17A2.5, C17B7.3, C17B7.5, C17D12.1, C17D12.3, C17D12.6, C17E4.3, C17E4.9, C17F4.6, C17G1.3, C17G1.8, C17H11.1, C17H11.6, C17H12.2, C17H12.4, C18A11.5, C18A3.2, C18B12.2, C18B12.4, C18B12.6, C18B2.6, C18C4.2, C18C4.3, C18D1.2, C18D1.4, C18D11.2, C18E3.8, C18E9.10, C18E9.2, C18E9.3, C18F3.2, C18G1.8, C18H2.1, C18H2.3, C18H2.4, C18H2.5, C18H7.11, C18H7.2, C18H9.5, C23G10.6, C23H4.1, C23H4.2, C23H4.3, C23H4.4, C23H4.7, C23H4.8, C23H5.7, C24A11.8, C24A3.6, C24A8.1, C24A8.3, C24B5.1, C24B5.3, C24F3.1, C24F3.5, C24G6.2, C24G7.1, C24G7.2, C24G7.4, C24H10.1, C24H11.8, C25A1.5, C25B8.1, C25B8.4, C25B8.5, C25B8.7, C25E10.5, C25F6.4, C25F6.7, C25G4.10, C25G6.2, C25G6.5, C25H3.9, C26B2.8, C26C6.9, C26D10.5, C26D10.7, C26F1.6, C26G2.1, C26H9A.1, C27A12.9, C27A7.1, C27C12.4, C27C12.5, C27D8.4, C27F2.2, C27H5.8, C28H8.4, C29A12.4, C29E6.2, C29E6.4, C29F4.2, C29F5.4, C29H12.2, C30A5.10, C30A5.5, C30A5.7, C30B5.2, C30B5.5, C30B5.9, C30D11.1, C30F12.6, C30G12.4, C30H6.2, C30H6.5, C30H6.6, C31A11.1, C31A11.5, C31A11.7, C31E10.6, C31E10.7, C31E10.8, C31H5.3, C32A3.3, C32C4.1, C32C4.2, C32C4.7, C32D5.2, C32E8.8, C33A11.2, C33A12.6, C33D12.2, C33D12.3, C33G3.3, C33H5.1, C33H5.11, C33H5.14, C33H5.18, C33H5.19, C34B2.10, C34B2.8, C34B4.5, C34D10.1, C34D4.4, C34F6.10, C34F6.2, C34F6.3, C34F6.7, C34G6.4, C34G6.6, C34H3.1, C35A11.4, C35A5.1, C35A5.2, C35A5.3, C35A5.5, C35C5.11, C35C5.2, C35C5.5, C35D10.1, C36B1.12, C36B7.6, C36E8.3, C36H8.1, C36H8.2, C37A5.1, C37A5.2, C37A5.4, C37C3.12, C37C3.7, C37E2.2, C37E2.3, C38C10.1, C38C10.2, C38C6.2, C38D9.5, C39B10.2, C39E6.6, C39E9.10, C39E9.7, C40C9.1, C40C9.2, C40C9.5, C40H1.4, C41C4.10, C41C4.5, C41C4.7, C41D11.9, C41D7.2, C41G11.4, C41G7.9, C42C1.16, C42C1.7, C42C1.8, C42D4.2, C42D8.8, C43C3.2, C43C3.3, C43F9.10, C43F9.6, C43F9.9, C43G2.1, C43G2.2, C43G2.4, C43H6.6, C43H6.7, C43H6.9, C44B7.11, C44B7.3, C44B7.6, C44B7.8, C44B7.9, C44C10.3, C44E12.3, C44F1.5, C44H4.1, C44H4.2, C44H9.1, C45B2.4, C45B2.7, C45G7.5, C46A5.2, C46C11.2, C46C2.2, C46F11.1, C46F4.2, C47A10.1, C47A4.2, C47B2.1, C47C12.6, C47D12.3, C47E12.2, C47G2.1, C47G2.3, C47G2.4, C48A7.1, C48A7.2, C48B4.2, C48B4.4, C48C5.1, C48D1.3, C48E7.5, C48E7.8, C48E7.9, C49A1.2, C49A1.3, C49A9.7, C49A9.8, C49C3.1, C49C3.21, C49D10.4, C49D10.7, C49D10.8, C49F5.5, C49F8.2, C49G7.1, C49H3.1, C50B6.11, C50D2.2, C50D2.9, C50E3.16, C50F4.14, C50F7.1, C50H2.1, C50H2.12, C50H2.2, C51E3.6, C52A11.4, C52B11.3, C52B9.4, C52B9.6, C52B9.9, C52D10.12, C52E12.3, C52E2.7, C53A5.13, C53A5.5, C53B4.1, C53B4.3, C53B4.6, C53C11.3, C53C7.1, C53C9.3, C53D5.2, C53D5.5, C53D6.3, C54A12.1, C54A12.2, C54D1.1, C54D10.4, C54D2.5, C54F6.13, C54F6.4, C54G10.3, C54G4.4, C54G4.8, C54G7.2, C54H2.5, C55B7.6, C55C2.5, C56A3.3, C56A3.4, C56A3.7, C56C10.13, C56E6.1, C56E6.5, C56G2.1, C56G2.6, C56G3.1, C56G7.2, cTel55X.1, D1007.5, D1009.1, D1009.3, D1014.2, D1022.1, D1022.6, D1037.2, D1044.2, D1044.3, D1046.3, D1046.4, D1046.5, D1065.1, D1073.1, D2013.10, D2013.8, D2021.2, D2024.3, D2062.12, D2063.2, D2085.6, D2089.2, D2092.1, D2092.3, D2092.5, D2092.7, D2096.2, DH11.3, DY3.7, E01G6.3, E01H11.1, E02D9.1, E02H4.1, E03G2.2, E03H12.3, E03H4.7, E04A4.5, E04D5.2, E04D5.3, E04F6.11, E04F6.4, EGAP2.3, EGAP9.2, E_BE45912.2, F01D4.1, F01D4.2, F01D5.10, F01E11.1, F01E11.4, F01E11.5, F01G12.1, F01G12.2, F01G4.5, F01G4.6, F02C12.1, F02C9.3, F02C9.4, F02D10.5, F02E11.1, F02E8.2, F02E8.5, F02E8.6, F02E9.7, F02G3.1, F07A5.1, F07A5.3, F07B10.1, F07B10.5, F07B7.14, F07C3.1, F07C3.10, F07C3.7, F07E5.8, F07F6.6, F07F6.7, F07F6.8, F07G11.1, F07G11.3, F07G6.1, F07H5.2, F08A10.1, F08B1.2, F08B12.1, F08B12.2, F08B12.3, F08B4.2, F08C6.4, F08F1.1, F08F1.5, F08F1.7, F08F3.3, F08F8.8, F08G12.10, F08G12.5, F08G5.2, F08G5.5, F09A5.1, F09A5.2, F09B12.2, F09B12.3, F09B12.6, F09B9.1, F09B9.3, F09C12.1, F09E5.11, F09E8.2, F09E8.7, F09F9.4, F09G2.1, F09G2.3, F09G2.5, F09G2.6, F09G2.8, F09G8.4, F09G8.9, F10A3.1, F10C2.7, F10C5.2, F10D2.11, F10D2.12, F10D2.2, F10D2.5, F10D2.6, F10D2.7, F10D2.9, F10D7.2, F10E7.10, F10E7.2, F10E7.9, F10F2.4, F10G7.5, F11A10.5, F11A5.10, F11A5.5, F11A5.8, F11A5.9, F11C1.3, F11C3.2, F11C7.1, F11C7.4, F11D5.3, F11D5.7, F11E6.5, F11E6.6, F11E6.8, F11G11.9, F11H8.2, F12A10.4, F12B6.1, F12B6.2, F12B6.3, F12D9.1, F12F3.1, F13A2.6, F13B12.2, F13B9.6, F13B9.8, F13D11.1, F13D2.2, F13D2.3, F13E6.3, F13E6.5, F13G3.7, F13G3.8, F13G3.9, F13H10.3, F13H10.4, F13H10.5, F13H8.4, F14B6.5, F14B8.1, F14B8.3, F14B8.7, F14D12.4, F14D12.5, F14D12.6, F14D2.6, F14D7.6, F14E5.1, F14E5.2, F14E5.3, F14F11.1, F14F3.3, F14F4.1, F14F4.3, F14H3.10, F14H3.2, F14H8.6, F15A2.2, F15A4.8, F15A8.5, F15B10.1, F15B9.2, F15B9.7, F15D4.3, F15E6.2, F15H10.4, F15H10.7, F15H10.8, F16A11.3, F16B12.1, F16B3.1, F16C3.1, F16D3.6, F16D3.7, F16F9.1, F16F9.5, F16H11.1, F16H11.3, F17B5.2, F17C11.12, F17C11.7, F17C8.1, F17C8.5, F17E9.7, F17E9.8, F18A12.5, F18A12.8, F18F11.3, F18F11.5, F18G5.4, F18H3.5, F19B6.4, F19D8.1, F19H6.4, F19H8.5, F20A1.7, F20B10.1, F20B4.6, F20B6.3, F20C5.2, F20C5.4, F20D1.1, F20D1.10, F20D1.7, F20D1.8, F21A10.2, F21A3.7, F21C10.1, F21C10.12, F21C10.3, F21C10.4, F21C3.1, F21D12.3, F21F3.3, F21F3.5, F21F3.7, F21F8.11, F21F8.2, F21G4.1, F21G4.2, F21H12.4, F21H7.9, F22A3.3, F22B5.10, F22B5.3, F22B7.10, F22B7.7, F22E10.1, F22E10.2, F22E10.3, F22E10.5, F22E5.11, F22E5.3, F22F4.2, F22F7.1, F22F7.3, F22F7.4, F23B2.3, F23D12.6, F23F1.6, F23F12.13, F23F12.3, F23H11.5, F23H11.9, F23H12.1, F23H12.2, F23H12.6, F23H12.8, F25B4.7, F25D1.2, F25D1.4, F25D7.1, F25D7.2, F25D7.5, F25E5.2, F25F2.2, F25F8.2, F25G6.3, F25G6.4, F25G6.7, F25H5.8, F26A1.8, F26A3.6, F26D10.11, F26D10.9, F26D11.10, F26D2.10, F26E4.11, F26E4.6, F26F2.7, F26F4.3, F26F4.4, F26F4.9, F26G1.6, F27B3.2, F27C1.2, F27C8.1, F27D4.7, F27D9.2, F27D9.6, F27D9.7, F27E11.1, F27E11.2, F27E11.3, F28A10.7, F28A12.1, F28B3.10, F28B3.6, F28B3.9, F28C6.4, F28D1.11, F28D1.8, F28D1.9, F28E10.2, F28F8.1, F28F9.4, F28G4.1, F28G4.5, F28H1.4, F28H7.10, F28H7.6, F29C4.1, F29D11.1, F29F11.2, F29F11.4, F30A10.4, F30A10.6, F30B5.7, F30F8.9, F31A9.3, F31B12.3, F31B9.1, F31C3.4, F31D4.7, F31D5.1, F31D5.2, F31D5.3, F31D5.4, F31D5.5, F31E8.2, F31E8.4, F31F4.15, F31F4.7, F31F6.5, F31F6.6, F31F7.2, F32A11.7, F32A5.5, F32A6.5, F32A7.3, F32B6.9, F32D1.3, F32D8.14, F32D8.15, F32D8.5, F32D8.6, F32G8.4, F32H5.4, F32H5.7, F33A8.5, F33A8.9, F33C8.1, F33C8.3, F33D11.11, F33D11.12, F33D11.5, F33D11.9, F33D4.2, F33D4.4, F33D4.7, F34D10.2, F34D6.3, F34D6.4, F35A5.5, F35C11.5, F35C12.2, F35C8.4, F35C8.5, F35C8.7, F35D11.2, F35D11.3, F35D2.3, F35D2.4, F35E12.4, F35E2.1, F35E2.6, F35F10.10, F35G8.1, F35H10.10, F35H10.4, F35H12.3, F35H8.6, F36A2.4, F36A2.9, F36D1.8, F36D3.5, F36D4.4, F36F2.4, F36F2.5, F36G3.3, F36G9.12, F36G9.13, F36G9.3, F36H1.10, F36H1.2, F36H1.4, F36H1.5, F36H1.9, F36H2.1, F36H2.2, F36H5.5, F36H9.4, F37A4.1, F37A4.4, F37B4.7, F37C12.2, F37C4.1, F37C4.2, F37C4.3, F37E3.2, F37H8.4, F38A1.11, F38A3.1, F38A5.10, F38A5.11, F38A5.12, F38A5.14, F38A5.5, F38A5.9, F38B2.3, F38B6.6, F38B7.10, F38B7.11, F38C2.4, F38E1.9, F38E11.12, F39B2.8, F39B3.2, F39C12.3, F39E9.12, F39G3.1, F39G3.4, F39G3.5, F39G3.6, F39H12.4, F40A3.2, F40A3.5, F40B5.2, F40E10.6, F40E12.2, F40F9.1, F40F9.2, F40F9.5, F40F9.9, F40G9.1, F40G9.18, F40G9.9, F41A4.1, F41B4.4, F41C3.2, F41C3.4, F41C6.7, F41D3.10, F41D3.2, F41D3.4, F41D3.5, F41D9.5, F41E6.14, F41E7.1, F41E7.2, F41E7.3, F41G3.4, F41G4.3, F41H10.11, F41H10.5, F41H10.7, F41H10.8, F42A8.3, F42C5.2, F42E11.1, F42E11.2, F42F12.3, F42G8.10, F42G8.11, F42G8.6, F42G8.9, F42G9.6, F43C9.4, F43D9.1, F43E2.4, F43G9.13, F43G9.2, F43G9.3, F43G9.6, F44A2.2, F44B9.5, F44C8.7, F44D12.2, F44D12.9, F44E7.7, F44E7.9, F44F4.1, F44F4.4, F44F4.6, F44G3.10, F44G4.8, F45C12.5, F45C12.6, F45D3.5, F45E10.2, F45E4.11, F45E4.7, F45E6.1, F45F2.1, F45F2.5, F45F2.6, F45F2.7, F45H10.1, F45H11.4, F46A9.3, F46C3.1, F46C5.8, F46E10.9, F46F3.2, F46G10.5, F46G11.1, F47A4.1, F47B3.3, F47B7.2, F47B8.10, F47B8.5, F47C10.6, F47D12.1, F47E1.2, F47E1.4, F47F6.3, F47G3.4, F47G9.1, F47G9.3, F48A11.1, F48C1.1, F48C1.2, F48C11.2, F48C5.1, F48E3.2, F48E3.7, F48F5.5, F48F7.2, F48F7.8, F48G7.3, F49A5.2, F49A5.3, F49A5.5, F49A5.7, F49A5.9, F49B2.6, F49C12.1, F49C12.13, F49C12.4, F49C12.6, F49E11.3, F49E12.10, F49E12.9, F49E8.5, F49H12.6, F49H6.13, F49H6.3, F52B11.3, F52B5.1, F52C6.4, F52D10.1, F52D10.4, F52D10.5, F52E1.4, F52E4.4, F52F10.3, F52F10.4, F52F12.1, F52H2.2, F52H2.4, F52H2.6, F53A9.5, F53B1.2, F53B1.8, F53B2.2, F53B3.5, F53B6.1, F53B6.4, F53B6.6, F53B6.9, F53B7.2, F53B7.4, F53B7.5, F53C11.2, F53C11.6, F53C3.13, F53C3.3, F53C3.4, F53C3.5, F53C3.6, F53E10.2, F53F1.1, F53F10.8, F53F4.5, F53G12.3, F53H2.2, F53H8.3, F53H8.4, F54B11.1, F54B11.3, F54C1.1, F54D1.5, F54D1.6, F54D12.3, F54D7.3, F54D8.2, F54E2.5, F54E7.1, F54F12.1, F54F2.1, F54F2.9, F54F7.4, F54F7.5, F54G8.3, F54G8.5, F55A11.2, F55A11.3, F55A11.5, F55A11.7, F55A12.4, F55A3.1, F55A4.1, F55A4.10, F55A4.8, F55B12.2, F55C12.5, F55C5.3, F55D10.3, F55D10.5, F55D12.6, F55E10.6, F55E10.7, F55F3.3, F55F8.1, F55F8.9, F55G1.12, F55G1.6, F55H12.1, F55H12.3, F55H2.5, F56A11.5, F56A4.10, F56A4.11, F56A4.12, F56A8.1, F56A8.3, F56A8.7, F56B3.11, F56B3.6, F56B3.7, F56B6.5, F56C11.1, F56C11.2, F56C3.6, F56C9.3, F56D1.2, F56D1.5, F56D5.9, F56E10.3, F56F3.2, F56F4.1, F56F4.3, F56F4.5, F56G4.1, F56H1.1, F56H1.2, F56H1.3, F56H11.3, F56H11.4, F56H6.11, F56H6.12, F57A10.3, F57A8.2, F57A8.4, F57A8.7, F57B1.1, F57B1.8, F57B1.9, F57B10.10, F57B10.5, F57B9.4, F57C12.4, F57C12.5, F57C7.2, F57C7.3, F57C7.4, F57C9.6, F57F10.1, F57G4.1, F57G8.5, F57H12.2, F57H12.4, F58A3.2, F58A6.5, F58E1.6, F58E6.12, F58F6.5, F58F6.6, F58G11.1, F58G11.4, F58G4.4, F58G6.3, F58G6.4, F58G6.8, F58G6.9, F58H1.6, F58H7.3, F59A1.10, F59A1.13, F59A3.1, F59A3.4, F59B1.9, F59B10.1, F59B2.13, F59B2.2, F59C12.1, F59C12.2, F59C6.11, F59C6.2, F59D12.1, F59D6.6, F59F3.1, F59F3.2, F59F3.4, F59F3.5, F59F4.2, F59F4.4, F59F5.1, F59F5.3, F59G1.1, F59G1.2, F59H6.4, H02I12.3, H04J21.1, H04J21.3, H04M03.2, H06H21.10, H06I04.2, H06I04.5, H09F14.1, H10E21.2, H10E21.5, H11E01.2, H12I19.4, H12I19.5, H13N06.5, H13N06.6, H14N18.4, H16O14.1, H17B01.1, H19J13.1, H19M22.2, H19N07.4, H20E11.1, H20J04.1, H20J04.6, H21P03.3, H22D07.1, H22K11.4, H23L24.4, H23N18.1, H23N18.2, H23N18.3, H30A04.1, H32C10.3, H32K16.1, H35N03.1, H41C03.3, JC8.5, K01A11.4, K01A2.1, K01A2.8, K01D12.1, K01D12.4, K01D12.6, K01H12.2, K02A2.3, K02B12.3, K02B2.4, K02D7.5, K02E10.4, K02E10.8, K02E11.1, K02E7.10, K02F2.6, K02F3.6, K02F3.8, K02F6.3, K02F6.4, K02G10.1, K02G10.5, K02G10.6, K02G10.7, K02G10.8, K03A1.2, K03A11.4, K03B8.2, K03B8.5, K03B8.9, K03E5.1, K03E6.5, K03F8.2, K03H1.5, K03H6.1, K03H6.5, K04A8.10, K04A8.2, K04A8.4, K04B12.1, K04D7.2, K04D7.4, K04E7.2, K04F1.1, K04F1.12, K04F10.4, K04G11.5, K04G2.9, K05B2.5, K05C4.11, K05C4.2, K05F1.1, K05F1.6, K06A1.3, K06A4.4, K06A5.2, K06A9.3, K06B4.12, K06C4.6, K06C4.8, K06C4.9, K06H6.3, K07A1.16, K07A1.8, K07A12.2, K07A3.2, K07B1.4, K07B1.5, K07C10.1, K07D8.1, K07E3.7, K07F5.12, K07F5.15, K07F5.6, K07G5.5, K07H8.2, K08B4.3, K08B4.4, K08C7.1, K08C7.2, K08C7.5, K08D9.3, K08D9.6, K08E4.6, K08E5.2, K08E5.3, K08E7.9, K08F11.5, K08F4.3, K08F4.4, K08F9.1, K08F9.3, K08H10.6, K09A9.3, K09A9.6, K09C4.1, K09C4.4, K09C4.5, K09C8.1, K09D9.11, K09E10.1, K09E10.2, K09E2.4, K09E4.2, K09E4.5, K09E9.1, K09E9.2, K09F5.1, K09F6.3, K09F6.4, K09G1.1, K09G1.4, K09H9.6, K10B4.4, K10C2.3, K10C3.3, K10C8.2, K10D11.5, K10D2.5, K10D3.1, K10D6.1, K10D6.2, K10F12.3, K10G9.1, K10H10.1, K11C4.2, K11C4.5, K11D12.5, K11D9.2, K11D9.3, K11G12.2, K11G12.3, K11G12.4, K11G12.6, K11G12.7, K11G9.5, K11H12.3, K11H3.7, K12B6.2, K12C11.3, K12C11.6, K12C11.7, K12G11.1, K12G11.2, K12H6.6, M01A10.3, M01B2.6, M01D7.2, M01D7.6, M01E10.2, M01E11.1, M01E5.1, M01F1.5, M01G5.3, M01G5.5, M02A10.2, M02B1.1, M02B1.3, M02B7.4, M02F4.8, M03A1.1, M03A1.3, M03A1.8, M03B6.2, M03C11.5, M03E7.5, M03F4.3, M03F8.2, M04B2.5, M04G7.1, M05B5.1, M05D6.5, M110.2, M117.1, M142.2, M153.2, M162.5, M176.4, M176.6, M176.7, M18.8, M195.3, M28.1, M60.5, M70.1, M70.3, M79.2, M88.1, M88.6, MTCE.11, MTCE.12, MTCE.21, MTCE.23, MTCE.25, MTCE.26, MTCE.31, MTCE.34, MTCE.35, MTCE.4, PDB1.1, R01B10.4, R01B10.5, R01B10.6, R01E6.1, R01E6.4, R01H2.3, R02C2.3, R02D5.6, R02E12.6, R02E12.8, R02F11.2, R02F11.3, R02F2.8, R03A10.6, R03D7.8, R03E1.2, R03E1.3, R03E9.2, R03E9.3, R03E9.4, R03G5.3, R03H4.1, R03H4.5, R03H4.6, R04B5.9, R04D3.1, R04E5.2, R04F11.1, R04F11.4, R05C11.3, R05D11.5, R05D3.2, R05D7.3, R05G6.6, R05H10.6, R05H5.5, R06A4.10, R06B10.1, R06B10.4, R06B9.6, R07A4.1, R07B1.3, R07B7.1, R07B7.10, R07B7.12, R07B7.4, R07B7.5, R07B7.6, R07B7.8, R07B7.9, R07D5.1, R07E3.1, R07E3.3, R07E3.4, R07E3.5, R07E5.13, R07E5.7, R08B4.4, R08C7.2, R08F11.1, R08F11.6, R09B5.10, R09B5.11, R09B5.13, R09B5.2, R09B5.3, R09B5.4, R09B5.6, R09B5.8, R09B5.9, R09D1.1, R09D1.10, R09D1.11, R09D1.12, R09D1.2, R09D1.3, R09D1.5, R09D1.6, R09E10.5, R09F10.1, R09F10.4, R09H10.4, R105.1, R106.2, R107.1, R107.8, R10D12.1, R10D12.5, R10D12.6, R10D12.7, R10D12.9, R10E11.2, R10E11.8, R10E4.9, R10F2.1, R11E3.2, R11F4.2, R11G1.1, R11G1.6, R11G10.1, R11G11.12, R11H6.2, R11H6.3, R12C12.3, R12C12.6, R12E2.2, R12E2.4, R12E2.5, R12E2.9, R12G8.2, R12H7.1, R13.3, R134.1, R134.2, R13A1.2, R13A1.4, R13A1.9, R13A5.1, R13A5.4, R13A5.9, R13D11.4, R13D7.11, R13F6.4, R13F6.5, R13G10.4, R13H7.2, R144.6, R151.6, R155.1, R155.2, R155.3, R155.4, R166.2, R173.1, R173.3, R186.5, R186.6, R31.2, R57.1, SSSD1.1, T01A4.1, T01B11.3, T01B11.4, T01B11.7, T01B4.1, T01B4.2, T01C2.1, T01C3.10, T01C4.2, T01C8.7, T01D3.5, T01G5.2, T01G9.3, T01H10.1, T01H10.2, T01H10.3, T01H10.5, T01H10.6, T01H10.7, T01H3.1, T01H3.3, T01H3.4, T01H8.5, T02B11.6, T02B5.1, T02B5.3, T02C1.2, T02C5.1, T02C5.3, T02C5.5, T02D1.5, T02D1.6, T02E1.7, T02E9.1, T02E9.3, T02G5.12, T03D3.1, T03D8.5, T03D8.6, T03F1.12, T03F6.6, T03F7.1, T03G11.4, T04A11.3, T04A8.12, T04A8.9, T04B8.5, T04F8.1, T04F8.2, T04F8.4, T04G9.3, T04G9.5, T04H1.1, T04H1.7, T04H1.8, T05A1.1, T05A1.3, T05A1.5, T05A10.2, T05B4.1, T05B4.8, T05C12.2, T05C12.9, T05E11.2, T05E11.5, T05F1.1, T05G5.5, T05H4.1, T05H4.13, T05H4.4, T05H4.5, T06A4.2, T06C12.10, T06C12.8, T06C12.9, T06D4.4, T06D8.3, T06D8.5, T06D8.7, T06D8.9, T06E4.5, T06E6.5, T06E8.1, T06F4.2, T06H11.1, T07A5.2, T07A5.3, T07A5.6, T07C4.7, T07C4.8, T07C5.1, T07D1.3, T07D10.2, T07D4.1, T07E3.6, T07F10.1, T07F10.4, T07F12.2, T07F8.2, T07G12.2, T07G12.5, T07H6.1, T07H6.2, T07H8.6, T08A9.3, T08B1.1, T08D10.2, T08D2.2, T08G11.5, T08G3.7, T08H10.4, T09A12.3, T09A12.5, T09A5.11, T09A5.12, T09A5.3, T09B4.1, T09B9.2, T09E11.4, T09E11.5, T09E11.6, T09E11.7, T09E8.3, T09E8.4, T09H2.1, T10B10.5, T10B10.7, T10B10.8, T10B9.1, T10B9.10, T10B9.3, T10B9.5, T10C6.6, T10E9.4, T10E9.6, T10H10.1, T10H9.2, T10H9.4, T10H9.5, T11F8.3, T11F9.11, T11F9.12, T11F9.2, T11F9.21, T11F9.4, T11G6.2, T11G6.3, T11G6.4, T12A2.15, T12A2.2, T12B3.2, T12B3.3, T12B5.14, T12C9.3, T12G3.4, T13A10.10, T13B5.1, T13B5.4, T13C2.6, T13C5.1, T13C5.6, T13F2.1, T13F2.8, T13G4.3, T13H10.2, T13H5.8, T14A8.1, T14B1.2, T14B4.4, T14D7.2, T14D7.3, T14E8.1, T14E8.3, T14G10.6, T14G10.7, T14G12.6, T15B7.16, T15B7.2, T15D6.11, T15D6.2, T15D6.5, T16A1.2, T16A1.7, T16A9.5, T16G12.5, T16H5.1, T17A3.1, T18D3.3, T18H9.2, T18H9.5, T19A6.1, T19A6.3, T19A6.4, T19B10.5, T19B10.8, T19B4.7, T19C3.1, T19C3.4, T19D12.10, T19D12.4, T19D12.9, T19D7.1, T19D7.7, T19F4.1, T19H12.1, T19H12.10, T19H12.11, T19H12.6, T19H12.8, T19H12.9, T19H5.1, T19H5.2, T19H5.3, T20B12.9, T20B3.13, T20B3.15, T20D3.6, T20D3.8, T20D4.13, T20G5.4, T20G5.6, T20H9.6, T21B10.6, T21B4.4, T21B6.1, T21B6.5, T21C12.1, T21C9.1, T21C9.12, T21C9.3, T21D12.9, T21E3.3, T21E8.1, T21E8.2, T21E8.3, T21F2.1, T21H3.2, T21H3.4, T22A3.6, T22B11.2, T22C1.3, T22C1.7, T22C8.7, T22C8.8, T22D1.11, T22D1.12, T22D1.4, T22E5.2, T22E7.1, T22E7.2, T22F3.10, T22F3.11, T22F3.7, T22F3.8, T22F7.1, T22G5.5, T22H2.6, T22H9.2, T23B12.5, T23B3.2, T23B3.4, T23C6.5, T23D8.1, T23D8.2, T23F1.5, T23F1.7, T23F2.3, T23F2.5, T23G11.6, T23G4.4, T23G5.5, T23G7.2, T23H2.1, T23H2.2, T23H4.1, T24B1.1, T24C2.1, T24C4.4, T24C4.7, T24C4.8, T24D1.4, T24D8.1, T24F1.2, T24H7.1, T24H7.5, T25B6.7, T25B9.7, T25D10.2, T25D3.4, T25E12.10, T25E12.7, T25E12.8, T25E12.9, T25F10.3, T25G12.6, T25G3.2, T26A5.1, T26A5.4, T26A8.2, T26C12.4, T26E4.4, T26H10.1, T26H2.2, T26H2.7, T27A1.4, T27A1.5, T27B2.1, T27C5.8, T27D12.1, T27D12.2, T27E4.6, T27E9.1, T27E9.5, T27E9.9, T27F6.6, T27F7.3, T28A8.1, T28B4.4, T28B8.5, T28C12.1, T28C12.2, T28D6.9, T28D9.3, T28D9.7, T28F2.7, T28F3.3, T28F3.4, T28F3.5, T28F3.9, T28F4.2, T28H11.8, VF36H2L.1, VF39H2L.1, VW02B12L.1, VZK822L.1, W01A11.2, W01A8.3, W01A8.4, W01B11.2, W01B6.3, W01C8.6, W01C9.3, W01D2.3, W01G7.5, W02A2.1, W02A2.5, W02A2.9, W02B12.15, W02B3.6, W02B8.3, W02B8.4, W02B8.6, W02B9.1, W02C12.1, W02D3.4, W02D3.6, W02D7.7, W02D9.2, W02F12.2, W02H5.4, W02H5.5, W03A5.2, W03B1.7, W03B1.8, W03C9.6, W03D8.10, W03D8.6, W03D8.9, W03F11.2, W03F11.4, W03F9.4, W03G1.1, W03G11.2, W03G9.1, W04B5.2, W04C9.1, W04C9.3, W04C9.6, W04D2.3, W04G3.6, W04G5.5, W05B5.2, W05E10.1, W05G11.2, W05H12.1, W05H5.3, W06A7.3, W06A7.4, W06D12.1, W06D12.2, W06D12.3, W06D12.5, W06G6.1, W06G6.7, W06H3.1, W07A12.5, W07A12.6, W07A12.7, W07G1.5, W08D2.4, W08D2.5, W09B7.3, W09C2.3, W09D10.2, W09D6.6, W09G3.8, W10C4.1, W10C8.5, W10C8.6, W10G11.16, W10G11.5, Y102A11A.6, Y102A11A.8, Y102A5B.1, Y105C5A.23, Y105C5B.16, Y105C5B.2, Y105C5B.23, Y105E8A.12, Y105E8A.3, Y106G6H.8, Y108G3AL.2, Y110A2AL.12, Y110A2AL.8, Y110A2AR.1, Y110A7A.11, Y110A7A.3, Y111B2A.19, Y111B2A.20, Y113G7A.4, Y113G7A.5, Y113G7B.12, Y113G7C.1, Y116A8B.5, Y116A8C.14, Y116A8C.16, Y116A8C.463, Y116A8C.9, Y119C1B.3, Y119C1B.5, Y11D7A.11, Y11D7A.3, Y11D7A.9, Y15E3A.4, Y17D7A.4, Y17G7B.19, Y18D10A.12, Y18D10A.23, Y18D10A.6, Y18D10A.7, Y18H1A.12, Y18H1A.14, Y19D10A.10, Y19D10A.11, Y19D10A.12, Y19D10A.4, Y19D10A.5, Y19D10A.8, Y19D10B.5, Y22D7AL.11, Y22D7AL.15, Y22D7AL.8, Y22D7AR.13, Y22D7AR.14, Y22D7AR.2, Y22D7AR.7, Y23H5A.5, Y23H5B.4, Y25C1A.7, Y2H9A.4, Y32F6A.2, Y32F6A.4, Y32F6B.1, Y32F6B.2, Y32G9A.8, Y32H12A.5, Y34D9A.2, Y34D9A.8, Y34D9B.1, Y34F4.1, Y37A1A.2, Y37A1A.3, Y37A1B.11, Y37A1B.13, Y37A1C.1, Y37D8A.10, Y37D8A.13, Y37D8A.17, Y37D8A.26, Y37D8A.5, Y37D8A.6, Y37D8A.8, Y37E11AL.5, Y37E11AR.1, Y37E11AR.5, Y37E3.16, Y38C1AA.1, Y38C1AA.5, Y38F1A.2, Y38F1A.3, Y38F1A.8, Y38F2AL.1, Y38F2AL.4, Y38F2AR.2, Y38F2AR.7, Y38F2AR.9, Y38H6C.16, Y38H6C.17, Y38H6C.20, Y39A1A.22, Y39A1A.8, Y39A1B.2, Y39A3A.3, Y39A3B.2, Y39A3B.5, Y39B6A.18, Y39B6A.19, Y39B6A.27, Y39B6A.29, Y39B6A.30, Y39B6A.41, Y39B6A.6, Y39B6A.8, Y39C12A.8, Y39D8A.1, Y39D8B.1, Y39D8B.3, Y39D8C.1, Y39E4A.2, Y39E4B.3, Y39E4B.4, Y39E4B.5, Y39E4B.7, Y39E4B.9, Y39G10AR.6, Y39H10A.2, Y40C5A.2, Y40D12A.2, Y40H4A.1, Y40H7A.11, Y41C4A.11, Y41D4A.4, Y41D4A.8, Y41D4B.24, Y41E3.3, Y41G9A.4, Y42A5A.1, Y42G9A.6, Y42H9AR.2, Y42H9B.2, Y43F4B.7, Y43F8A.3, Y43F8A.5, Y43F8B.10, Y43F8C.12, Y43H11AL.2, Y44A6E.1, Y45F10B.1, Y45F10D.3, Y45G12B.2, Y45G12C.1, Y45G5AL.2, Y46B2A.3, Y46G5A.17, Y46G5A.2, Y46G5A.26, Y46G5A.30, Y46G5A.5, Y46G5A.8, Y46H3A.6, Y46H3D.4, Y47D3A.11, Y47D3A.30, Y47D3B.11, Y47D3B.5, Y47D3B.7, Y47D7A.14, Y47D7A.16, Y47G6A.1, Y47G6A.2, Y47G6A.31, Y47G6A.7, Y47H9A.1, Y47H9C.2, Y47H9C.4, Y48A6B.6, Y48B6A.10, Y48B6A.4, Y48E1B.14, Y48E1B.2, Y48G1BM.9, Y48G1C.5, Y48G8AL.11, Y48G8AL.13, Y48G9A.10, Y49A3A.1, Y49C4A.8, Y49E10.11, Y49E10.20, Y49F6B.9, Y4C6A.2, Y4C6A.3, Y4C6B.2, Y4C6B.3, Y4C6B.4, Y4C6B.5, Y50D4B.4, Y50D4B.5, Y50D4B.7, Y50E8A.16, Y50E8A.17, Y51A2B.2, Y51A2D.18, Y51A2D.19, Y51A2D.4, Y51A2D.5, Y51B9A.6, Y51F10.4, Y51H4A.25, Y52B11A.7, Y52D5A.1, Y52E8A.4, Y53C10A.5, Y53C10A.9, Y53C12A.1, Y53C12A.2, Y53C12A.3, Y53F4B.12, Y53F4B.2, Y53F4B.25, Y53F4B.28, Y53G8AM.4, Y53G8AR.7, Y53G8B.2, Y53G8B.4, Y53H1B.1, Y53H1C.1, Y54E10A.14, Y54E10BR.1, Y54E10BR.5, Y54E10BR.7, Y54E2A.1, Y54E2A.12, Y54E5A.1, Y54E5B.1, Y54F10AL.1, Y54F10AM.7, Y54F10BM.6, Y54G2A.18, Y54G2A.2, Y54G2A.25, Y54G2A.35, Y54G2A.4, Y54G9A.3, Y54G9A.4, Y55B1BM.1, Y55D5A.5, Y55D5A.6, Y55F3AL.1, Y55F3AR.1, Y55F3AR.2, Y55F3BL.2, Y55F3BR.4, Y55F3C.3, Y55F3C.7, Y55H10A.1, Y56A3A.2, Y56A3A.22, Y56A3A.32, Y56A3A.36, Y57A10A.10, Y57A10A.28, Y57A10A.3, Y57A10A.35, Y57A10B.1, Y57A10C.10, Y57E12AL.1, Y57E12AM.1, Y57G11C.15, Y57G11C.17, Y57G11C.2, Y57G11C.23, Y57G11C.31, Y57G11C.37, Y57G11C.4, Y57G11C.44, Y57G11C.49, Y57G7A.6, Y58A7A.1, Y58G8A.1, Y58G8A.4, Y59A8B.21, Y59C2A.2, Y59E1A.1, Y59E9AL.4, Y59E9AL.7, Y59H11AL.1, Y59H11AR.2, Y59H11AR.4, Y5H2B.1, Y60A3A.14, Y60A3A.19, Y60C6A.1, Y61A9LA.1, Y62E10A.10, Y63D3A.6, Y63D3A.8, Y64G10A.6, Y65B4BR.3, Y66D12A.13, Y66D12A.21, Y66H1B.4, Y67A10A.1, Y67A10A.3, Y67A10A.8, Y67A10A.9, Y67D8C.10, Y67D8C.9, Y67H2A.4, Y67H2A.8, Y69A2AR.19, Y69A2AR.31, Y69A2AR.4, Y69A2AR.6, Y69E1A.1, Y69E1A.3, Y69E1A.7, Y69H2.1, Y69H2.11, Y69H2.12, Y69H2.14, Y69H2.2, Y6B3B.10, Y6B3B.11, Y6B3B.3, Y6B3B.5, Y6E2A.9, Y70D2A.1, Y70G10A.2, Y70G10A.3, Y71A12B.4, Y71A12C.2, Y71D11A.1, Y71D11A.5, Y71F9AM.6, Y71F9AR.1, Y71F9B.3, Y71F9B.5, Y71F9B.8, Y71G10AR.1, Y71G10AR.4, Y71G12B.23, Y71G12B.25, Y71H2AM.2, Y71H2AM.22, Y71H2AM.25, Y71H2AM.4, Y71H2AM.9, Y71H2AR.2, Y71H9A.1, Y71H9A.2, Y71H9A.3, Y73B6BL.19, Y73B6BL.26, Y73B6BL.31, Y73B6BL.36, Y73B6BL.42, Y73B6BL.7, Y73B6BR.1, Y73C8B.4, Y73E7A.3, Y73E7A.6, Y73F8A.1, Y73F8A.11, Y73F8A.2, Y73F8A.30, Y74C10AL.2, Y74C10AR.3, Y74E4A.1, Y75B7AL.1, Y75B8A.26, Y75B8A.5, Y76A2A.2, Y76A2B.6, Y76B12C.1, Y77E11A.4, Y7A5A.1, Y7A9A.1, Y80D3A.5, Y80D3A.7, Y80D3A.8, Y82E9BR.16, Y82E9BR.3, Y87G2A.13, Y87G2A.14, Y87G2A.18, Y87G2A.19, Y8G1A.2, Y94H6A.5, Y97E10AR.2, Y97E10AR.6, Y97E10B.7, ZC101.3, ZC13.1, ZC155.4, ZC155.7, ZC168.1, ZC190.1, ZC190.2, ZC196.5, ZC196.7, ZC196.8, ZC196.9, ZC21.2, ZC239.16, ZC239.17, ZC239.5, ZC239.7, ZC250.3, ZC262.10, ZC262.3, ZC262.9, ZC317.3, ZC328.1, ZC328.3, ZC374.1, ZC376.1, ZC376.2, ZC376.3, ZC395.3, ZC410.4, ZC412.1, ZC412.2, ZC416.8, ZC434.6, ZC443.5, ZC443.6, ZC455.3, ZC455.4, ZC455.5, ZC455.6, ZC482.1, ZC482.3, ZC482.5, ZC482.7, ZC504.2, ZC504.5, ZC506.3, ZC506.4, ZC513.5, ZC518.1, ZC8.1, ZC84.2, ZC84.4, ZK1010.6, ZK1010.9, ZK1025.3, ZK1037.6, ZK1053.6, ZK1053.7, ZK1055.4, ZK1058.2, ZK1067.1, ZK1067.4, ZK1067.5, ZK1086.1, ZK112.7, ZK1236.7, ZK1248.14, ZK1251.3, ZK1251.8, ZK1307.7, ZK1321.2, ZK1321.3, ZK154.7, ZK180.1, ZK180.3, ZK185.2, ZK185.4, ZK185.5, ZK256.1, ZK265.9, ZK270.1, ZK287.2, ZK370.4, ZK370.7, ZK377.2, ZK381.4, ZK381.8, ZK40.1, ZK418.3, ZK418.5, ZK418.6, ZK418.7, ZK455.2, ZK455.3, ZK455.7, ZK455.8, ZK484.2, ZK512.3, ZK512.6, ZK520.2, ZK524.1, ZK525.2, ZK54.1, ZK550.1, ZK550.2, ZK563.1, ZK563.2, ZK563.4, ZK563.6, ZK6.6, ZK6.8, ZK616.6, ZK622.2, ZK632.10, ZK632.6, ZK637.1, ZK637.8, ZK643.3, ZK675.1, ZK675.3, ZK678.8, ZK682.2, ZK682.5, ZK686.3, ZK688.2, ZK757.1, ZK757.4, ZK770.1, ZK770.3, ZK783.1, ZK792.1, ZK792.2, ZK792.3, ZK792.7, ZK795.4, ZK809.4, ZK813.5, ZK816.5, ZK822.3, ZK822.5, ZK829.10, ZK829.9, ZK849.4, ZK849.5, ZK858.1, ZK858.5, ZK858.6, ZK858.7, ZK892.3, ZK896.8, ZK896.9, ZK899.2, ZK930.2, ZK938.5, ZK938.6, ZK938.7, ZK945.1, ZK945.9, ZK970.1, ZK970.5, ZK970.6, ZK973.5

**TM proteins without recognizable domains (1309)**

AH10.2, AH9.4, AH9.6, B0025.5, B0034.5, B0041.5, B0198.2, B0207.10, B0207.8, B0207.9, B0228.6, B0244.11, B0244.4, B0303.16, B0303.8, B0310.1, B0310.3, B0393.8, B0403.6, B0416.3, B0432.1, B0454.5, B0495.9, B0496.6, B0511.14, B0546.3, C01A2.2, C01B12.4, C01F6.2, C01G12.7, C01G6.2, C02F5.5, C02G6.3, C03A3.1, C03E10.1, C03E10.3, C03H12.1, C04A11.1, C04C3.6, C04E6.4, C04F12.7, C04F5.2, C04F6.2, C04G6.11, C05A9.2, C05B5.2, C05B5.4, C05B5.8, C05C10.7, C05C12.4, C05C12.6, C05D9.7, C05E11.2, C05E11.3, C05E7.4, C05G5.3, C06A1.2, C06A5.10, C06A5.12, C06E1.1, C06E1.11, C06G4.4, C06G4.6, C06H5.7, C07C7.1, C07H4.1, C07H6.9, C08A9.3, C08B6.11, C08B6.2, C08B6.5, C08F1.10, C08F1.11, C08F1.6, C08F1.8, C08F11.1, C08F11.13, C08F11.2, C08F11.3, C08F8.9, C08G5.6, C08G5.7, C08G9.1, C08H9.15, C09B8.3, C09B8.8, C09B9.1, C09B9.2, C09B9.7, C09D4.6, C09E7.10, C09E8.1, C09F12.2, C09F12.3, C09F5.1, C09F9.1, C09G9.5, C10A4.10, C10A4.2, C10A4.3, C10A4.4, C10A4.5, C10A4.6, C10A4.7, C10A4.9, C10C5.1, C10C6.7, C10E2.5, C11E4.8, C11G10.1, C11H1.2, C12D12.3, C12D8.13, C12D8.20, C13A10.1, C13A2.12, C13F10.1, C14A4.12, C14A4.6, C14A4.7, C14A6.12, C14A6.13, C14A6.5, C14A6.6, C14A6.7, C14A6.8, C14B1.2, C14C10.6, C14E2.5, C14E2.7, C15A11.4, C15B12.2, C15C6.1, C15C7.6, C15C8.1, C15C8.5, C15C8.6, C15H11.11, C16C4.7, C16D6.3, C16D9.6, C17A2.3, C17E4.4, C17E7.12, C17E7.13, C17E7.9, C17F3.1, C17G1.1, C17G1.2, C17G10.7, C17H12.10, C18A11.3, C18A3.10, C18A3.4, C18A3.9, C18B12.1, C18D11.1, C18D11.6, C18E9.5, C18E9.9, C18G1.1, C18H7.10, C18H7.12, C18H7.13, C18H7.6, C23G10.10, C23H3.2, C23H5.11, C23H5.12, C24A11.2, C24B9.9, C24D10.2, C24D10.4, C24D10.5, C25A1.16, C25A11.1, C25B8.8, C25D7.10, C25E10.4, C25H3.16, C25H3.17, C26B2.7, C26B9.1, C26G2.2, C27A7.6, C27D6.3, C27F2.6, C27F2.8, C27H5.4, C27H5.6, C28C12.11, C29E4.15, C29F4.3, C29F5.5, C30E1.2, C30E1.3, C30E1.6, C30E1.7, C30F12.3, C30F12.5, C30F2.5, C30G4.4, C30G7.2, C30H6.9, C31B8.1, C31B8.16, C31G12.1, C32C4.3, C32D5.7, C32F10.4, C33C12.11, C33C12.4, C33D3.5, C33F10.1, C33F10.11, C34B2.11, C34B2.9, C34B4.3, C34C12.4, C34D1.4, C34F6.11, C35B1.3, C35B8.4, C35C5.10, C35D10.10, C35D10.3, C35D10.8, C36A4.10, C36A4.5, C36B1.13, C36B1.6, C36B7.3, C36B7.4, C36C9.6, C36E6.8, C37A5.11, C37C3.11, C38D4.1, C38H2.3, C39B10.1, C39D10.2, C39D10.6, C39F7.1, C39H7.4, C40A11.8, C41G7.8, C42C1.12, C42D8.1, C43F9.4, C44B7.4, C44B7.7, C44B9.3, C44C1.1, C44C10.10, C44C10.9, C44H4.8, C44H9.6, C44H9.7, C44H9.8, C45B11.5, C45B11.8, C45B11.9, C45E1.4, C45G9.11, C45H4.13, C46C11.3, C46E10.2, C46F11.5, C46G7.1, C47D12.5, C47E8.1, C47E8.3, C48B4.10, C48B4.11, C48B4.6, C48B4.7, C48B4.8, C48B4.9, C48B6.10, C48B6.9, C48C5.3, C48D1.9, C48E7.1, C48E7.6, C49A1.10, C49C8.2, C49F5.3, C49G9.1, C50A2.3, C50B8.4, C50B8.5, C50B8.6, C50E3.7, C50E3.8, C50E3.9, C50F2.7, C50H11.13, C50H11.8, C50H2.13, C53A5.1, C53C11.2, C53C11.5, C53D5.1, C53D6.10, C53D6.11, C53D6.5, C54D10.5, C54E10.3, C54E4.4, C54G4.5, C54G6.3, C55A6.11, C55C2.3, C55C2.4, C55C3.2, C55C3.6, CD4.1, D1007.10, D1007.15, D1007.3, D1022.9, D1053.4, D1069.1, D1079.1, D1081.11, D2005.6, D2024.5, D2030.12, D2062.4, D2062.5, D2062.7, D2063.4, D2092.4, DY3.8, E02H9.6, E04F6.10, F01E11.3, F01F1.14, F02A9.1, F02A9.7, F02C9.1, F02D10.6, F02E11.2, F07C3.2, F07C3.9, F07C6.2, F07C6.6, F07F6.2, F07H5.6, F07H5.7, F08A8.8, F08C6.5, F08F1.3, F08F3.8, F08F8.6, F09E10.6, F09F7.1, F09F9.1, F09F9.5, F10B5.9, F10D7.1, F10E7.3, F10E7.6, F10E9.1, F10E9.10, F11A10.6, F11A5.13, F11A5.15, F11C1.2, F11D5.5, F11E6.10, F11E6.11, F11G11.4, F11G11.5, F13B12.3, F13B12.7, F13B6.2, F13C5.1, F13C5.5, F13D12.10, F13D12.5, F13D12.8, F13E9.11, F13E9.16, F13G11.2, F13H10.8, F13H6.5, F13H8.11, F14B6.2, F14B8.5, F14B8.6, F14D7.8, F14D7.9, F14F7.4, F14F9.6, F14F9.8, F14H12.6, F14H12.7, F14H12.8, F15A4.5, F15A8.4, F15B9.10, F15B9.6, F15E6.8, F16H6.11, F17C11.13, F17C11.6, F17E9.5, F17H10.2, F17H10.4, F18A1.1, F18A12.2, F18C12.4, F18C5.10, F18E9.8, F18G5.2, F19C6.5, F19C7.5, F19C7.6, F20A1.2, F20A1.4, F20D12.7, F20D6.10, F20G2.6, F21C3.7, F21D9.2, F21E9.2, F21F3.6, F21H12.2, F21H7.12, F21H7.3, F22B5.4, F22B5.6, F22E5.9, F22G12.8, F23A7.1, F23B2.7, F23D12.1, F23D12.8, F23G4.1, F23H12.3, F23H12.7, F25B3.2, F25H5.2, F25H8.7, F26A1.6, F26A1.7, F26E4.2, F26F12.4, F26F12.5, F27D4.8, F27E5.3, F27E5.8, F28A10.3, F28A12.3, F28B3.5, F28B4.1, F28B4.4, F28D1.6, F28F9.2, F29A7.4, F29B9.11, F29B9.12, F29B9.7, F29B9.8, F30A10.12, F31C3.6, F31E3.6, F31E8.1, F31F4.17, F32A11.1, F32A7.8, F32B5.4, F32D8.10, F33G12.7, F33H2.8, F34D10.3, F35B12.9, F35C5.11, F35D11.5, F35E8.11, F35H12.6, F35H8.4, F36A2.7, F36G9.7, F36H12.15, F36H2.4, F36H2.6, F36H5.10, F36H5.4, F36H9.5, F37A4.3, F37B12.1, F37C12.21, F37C4.4, F37C4.7, F37H8.2, F38A5.8, F38B6.3, F38B7.2, F38E11.13, F38G1.3, F40A3.4, F40F12.3, F40F8.12, F40F8.3, F40G9.15, F40G9.8, F40H6.1, F40H6.6, F41B4.3, F41C3.8, F41F3.1, F41G3.18, F42A6.1, F42A9.3, F42A9.6, F42A9.9, F42C5.6, F42D1.4, F42E11.3, F42G4.7, F42G8.5, F43B10.1, F43B10.2, F43C11.6, F43D2.6, F43G9.8, F44E2.10, F44F4.3, F44F4.9, F44G3.7, F44G4.2, F44G4.3, F44G4.6, F45E4.6, F45H10.4, F45H10.5, F46B6.2, F46B6.9, F46C3.2, F46C5.2, F46C5.7, F46C8.3, F46F2.4, F46F5.16, F46F5.6, F46F5.9, F46G10.2, F47B10.3, F47B10.5, F47B10.8, F47B8.1, F47B8.2, F47D12.6, F47E1.5, F47G6.3, F48A11.4, F48B9.2, F48C1.3, F48C1.4, F48C1.6, F48E3.6, F48F5.6, F49B2.3, F49C12.12, F49E11.2, F49E12.12, F49E12.7, F49E7.2, F49F1.12, F49F1.14, F49F1.3, F52A8.1, F52B10.3, F52E1.9, F52F10.2, F53A2.1, F53A2.2, F53A3.1, F53A9.4, F53B1.3, F53F4.1, F53F4.16, F53F4.18, F53F4.2, F53F4.6, F53F8.6, F53G12.8, F54B11.8, F54B3.2, F54C1.8, F54C1.9, F54C8.6, F54C9.7, F54D7.2, F54E7.5, F54F12.2, F54F7.6, F54H12.7, F54H12.8, F55A12.2, F55A4.3, F55C12.4, F55D10.4, F55D12.1, F55E10.1, F55E10.2, F55E10.4, F55E10.5, F55F10.3, F55F8.6, F55G1.1, F55G1.15, F55G11.1, F56A11.4, F56A11.6, F56A12.2, F56C11.5, F56C3.3, F56C3.4, F56C3.5, F56C3.7, F56C4.2, F56D6.10, F56D6.11, F56D6.12, F56D6.13, F56D6.14, F56D6.6, F56F11.5, F56H11.2, F57A10.4, F57B1.5, F57B1.6, F57F4.2, F57F5.3, F57G9.3, F58A6.2, F58D12.1, F58D2.2, F58D5.2, F58D5.8, F58E6.11, F58E6.6, F58E6.8, F58F12.2, F58F12.4, F58G1.7, F58H1.5, F58H1.8, F59A1.11, F59A1.12, F59A1.15, F59A3.13, F59A3.7, F59B1.6, F59B10.3, F59B10.4, F59B10.6, F59C6.14, F59C6.3, F59D12.2, F59E12.8, F59F4.3, H01G02.3, H03E18.2, H03G16.6, H04D03.1, H04M03.11, H04M03.12, H10E21.1, H12D21.11, H12D21.5, H12D21.9, H14E04.3, H20J04.7, H23N18.6, H27M09.5, H34C03.1, H35N09.2, JC8.12, K01A11.3, K01A2.12, K01A2.3, K01A2.4, K01A2.6, K01A2.9, K01A6.6, K01B6.3, K02B9.3, K02E10.5, K02E2.1, K02E7.11, K02E7.4, K02G10.3, K03A11.5, K03C7.3, K03D7.9, K03H1.12, K03H1.9, K03H6.4, K04C2.7, K04D7.6, K04E7.1, K04G11.1, K04G2.10, K04G2.12, K05C4.10, K05F1.8, K06A1.2, K06A4.2, K06B4.15, K06B4.3, K06B4.4, K07A1.1, K07A1.13, K07A1.17, K07A12.8, K07E3.9, K07H8.5, K08D10.10, K08D10.11, K08D8.1, K08D8.2, K08E4.3, K08E4.5, K08E7.4, K08F8.7, K08F9.5, K08F9.6, K09C6.10, K09C6.6, K09C8.2, K09D9.3, K09E9.3, K09F5.4, K09F5.6, K09G1.2, K09H11.6, K09H9.8, K10C2.2, K10C8.4, K10G6.5, K10H10.12, K11D12.8, K11H12.8, K12B6.4, K12B6.6, K12D12.4, K12H4.5, K12H6.2, M01A12.3, M01D7.9, M01F1.4, M01H9.4, M02A10.1, M02B1.2, M02B1.4, M03B6.4, M03B6.5, M03C11.9, M03E7.2, M03E7.3, M03F4.4, M03F8.1, M04B2.7, M04C7.4, M04D5.3, M04D8.4, M04D8.5, M04D8.6, M04D8.7, M04D8.8, M04G7.3, M05B5.2, M05B5.6, M05D6.6, M106.8, M117.6, M163.10, M163.9, M176.11, M28.2, M28.4, M28.8, M28.9, M60.4, M60.6, MTCE.16, MTCE.3, R01B10.3, R01H2.7, R01H2.8, R02D5.3, R02E4.2, R03G5.7, R03H10.2, R04A9.1, R04B5.1, R05C11.4, R05D11.9, R05D7.1, R05G9.2, R05G9.3, R06A10.1, R07A4.2, R07B1.9, R07D5.2, R07E5.17, R07H5.4, R09A8.5, R09F10.5, R09G11.1, R09H10.7, R09H3.1, R102.6, R102.8, R10A10.1, R10E11.9, R10E12.2, R10E4.3, R10E4.6, R10E9.3, R10H10.4, R11.4, R119.1, R11D1.7, R11G11.3, R12B2.7, R12B2.8, R12C12.4, R12C12.9, R12H7.4, R144.11, R144.12, R144.5, R148.4, R151.1, R160.3, R160.4, R160.6, R193.3, T01B10.5, T01B11.1, T01C3.11, T01C3.5, T01C8.2, T01D1.5, T01D1.7, T01G1.2, T01G9.1, T02D1.4, T02E1.1, T02E1.6, T02G5.14, T02G5.3, T02G6.4, T03F6.9, T03F7.5, T03F7.6, T03G11.9, T04A6.1, T04A6.2, T04C12.1, T04C12.7, T04C12.8, T04C12.9, T04C9.2, T04F8.9, T05A8.7, T05C12.11, T05C12.4, T05D4.3, T05E7.4, T05G5.11, T06C12.12, T06E4.9, T06E8.2, T06F4.1, T07A9.12, T07D3.6, T07G12.8, T08B2.3, T08B6.4, T08D10.3, T09A5.15, T09B4.2, T09B4.5, T09B9.5, T09F5.2, T10E9.5, T10E9.8, T10G3.2, T10H9.3, T11A5.5, T11F8.1, T11F8.2, T11F9.1, T12F5.2, T12G3.7, T13C2.7, T13F3.7, T13F3.9, T13H5.6, T14B4.3, T14B4.5, T14G11.1, T14G8.4, T15B7.14, T15B7.6, T15B7.8, T16G12.4, T16G12.8, T17A3.10, T18D3.5, T19C4.1, T19D12.7, T19D2.3, T19D7.5, T19H5.4, T20D3.11, T20F5.5, T20F5.8, T20G5.14, T21B10.4, T21C12.8, T21C9.11, T21C9.13, T21C9.5, T21D9.2, T21E12.2, T21E12.5, T21G5.2, T21H3.5, T22B11.4, T22B2.2, T22B2.3, T22B2.5, T22C1.9, T22C8.1, T22D1.8, T22E5.6, T23B3.6, T23E7.5, T23E7.6, T23G11.1, T23G11.10, T24A11.2, T24C12.1, T24C12.4, T24E12.5, T24F1.4, T24F1.7, T25D1.1, T25E12.16, T25E4.2, T25F10.4, T26A8.1, T26C11.8, T26C12.2, T26C5.3, T26G10.5, T27A1.1, T27A3.8, T27C4.1, T27C4.2, T27E7.3, T27F6.10, T27F6.7, T28A11.6, T28B4.2, T28B8.6, T28C6.3, T28C6.8, T28D9.4, T28F12.1, VB0393L.2, W01A11.3, W01B11.1, W01B6.8, W01D2.5, W01D2.6, W02B8.1, W02C12.2, W02D7.11, W02D7.3, W02D7.8, W02D9.8, W02D9.9, W02H5.10, W03B1.2, W03C9.8, W03D2.6, W03D8.3, W03F8.6, W03G9.3, W04B5.6, W04E12.2, W04E12.3, W04E12.4, W04E12.5, W04E12.9, W04G3.1, W05E10.2, W05F2.2, W05F2.3, W05H7.2, W05H9.1, W06A7.2, W06B11.3, W06D4.2, W06F12.2, W06G6.15, W06H8.4, W07E6.3, W07E6.5, W07G4.5, W08D2.3, W09B6.5, W09D10.5, W09G12.10, W10C8.3, W10D9.1, Y102A11A.1, Y102A11A.7, Y102A5C.36, Y102E9.1, Y105C5A.1269, Y105C5A.1270, Y105C5B.17, Y105C5B.8, Y105E8A.27, Y105E8A.7, Y106G6G.4, Y106G6H.16, Y110A7A.21, Y113G7A.10, Y113G7A.14, Y113G7B.11, Y116A8C.23, Y116A8C.8, Y119C1B.1, Y119C1B.12, Y14H12A.1, Y17G9A.4, Y18D10A.11, Y18D10A.4, Y18H1A.15, Y22D7AL.1, Y22D7AL.12, Y22D7AL.3, Y23H5B.12, Y23H5B.2, Y24D9A.6, Y24D9A.7, Y26D4A.17, Y26D4A.3, Y26D4A.5, Y2H9A.6, Y32B12B.1, Y32H12A.6, Y34B4A.7, Y34F4.2, Y34F4.5, Y34F4.6, Y35H6.1, Y36E3A.2, Y37A1B.15, Y37D8A.12, Y37D8A.16, Y37D8A.22, Y37E11AL.1, Y37E11AL.2, Y37F4.5, Y37H2A.14, Y37H2C.1, Y38C1AA.7, Y38C1AA.8, Y38C1AB.2, Y38C1AB.6, Y38C1BA.1, Y38E10A.28, Y38F1A.4, Y38F1A.7, Y38F2AR.3, Y38H6C.21, Y38H8A.7, Y39A1A.20, Y39A1A.27, Y39A3B.3, Y39A3CL.3, Y39B6A.10, Y39B6A.32, Y39E4A.1, Y39G10AR.16, Y39G10AR.5, Y39H10B.2, Y40B10A.5, Y40B10A.9, Y40C5A.4, Y41C4A.13, Y41C4A.17, Y41C4A.18, Y41C4A.21, Y41D4B.1, Y41E3.8, Y41G9A.2, Y43C5A.3, Y43C5A.7, Y43D4A.3, Y43D4A.4, Y43F8C.11, Y44A6D.6, Y44A6D.7, Y45F10B.13, Y45F10C.6, Y45F10D.15, Y45F10D.16, Y46G5A.22, Y47D3A.32, Y47D3A.5, Y47D9A.3, Y47D9A.5, Y47G6A.3, Y47G6A.32, Y47G7B.2, Y48C3A.5, Y48G1BL.5, Y49E10.29, Y50D4A.2, Y50E8A.11, Y51A2D.21, Y51B9A.7, Y51F10.3, Y51H4A.16, Y51H4A.21, Y51H7BR.3, Y51H7BR.4, Y51H7BR.7, Y51H7BR.8, Y51H7C.10, Y52B11A.1, Y53G8AM.7, Y53G8B.3, Y53H1C.3, Y54E10BL.3, Y54E2A.10, Y54E2A.5, Y54E2A.7, Y54E5A.2, Y54E5A.8, Y54G11A.14, Y54G2A.11, Y54G2A.13, Y54G2A.16, Y54G2A.36, Y54G2A.52, Y54G9A.5, Y54G9A.9, Y55F3BR.7, Y55F3C.9, Y55H10A.2, Y57A10A.14, Y57A10C.1, Y57A10C.11, Y57A10C.9, Y57E12AL.4, Y57E12B.1, Y57E12B.4, Y57G11B.7, Y57G11C.45, Y57G7A.8, Y58A7A.5, Y59A8A.3, Y62H9A.1, Y62H9A.11, Y62H9A.12, Y64H9A.1, Y65B4BL.3, Y65B4BL.4, Y65B4BL.7, Y66A7A.7, Y66D12A.11, Y66D12A.24, Y67D8C.4, Y67D8C.6, Y67H2A.5, Y69A2AR.14, Y69A2AR.23, Y69A2AR.27, Y69A2AR.9, Y69H2.9, Y6B3B.7, Y6G8.6, Y70C5A.4, Y70C5B.2, Y71A12B.11, Y71A12B.17, Y71F9AL.6, Y71F9AL.7, Y71F9B.1, Y71F9B.14, Y71G12A.4, Y71G12B.16, Y71G12B.26, Y71G12B.3, Y71G12B.33, Y71G12B.7, Y71H2AL.2, Y71H2AM.10, Y73B3A.1, Y73B6BL.14, Y73B6BL.22, Y73B6BL.47, Y73F8A.5, Y75B12A.2, Y75B7AL.2, Y75B8A.16, Y75B8A.33, Y75B8A.34, Y76A2A.1, Y76A2B.4, Y76G2A.2, Y77E11A.12, Y79H2A.1, Y79H2A.12, Y79H2A.2, Y81B9A.3, Y82E9BL.1, Y82E9BL.2, Y82E9BL.3, Y82E9BL.5, Y82E9BL.6, Y92H12BR.3, Y95B8A.4, Y97E10AL.1, Y9C12A.1, Y9C2UA.1, ZC13.10, ZC13.2, ZC132.8, ZC132.9, ZC190.5, ZC190.6, ZC190.8, ZC21.6, ZC266.1, ZC266.2, ZC317.2, ZC410.5, ZC449.4, ZC449.5, ZC477.3, ZC487.2, ZC513.3, ZC518.4, ZC53.4, ZC581.3, ZC84.7, ZK1010.5, ZK1010.8, ZK1053.2, ZK1055.3, ZK1073.2, ZK1086.2, ZK1098.12, ZK1290.13, ZK1290.15, ZK1320.5, ZK154.6, ZK180.7, ZK218.4, ZK287.9, ZK353.2, ZK353.4, ZK355.2, ZK370.8, ZK39.10, ZK402.1, ZK470.1, ZK484.5, ZK512.1, ZK512.11, ZK54.3, ZK546.1, ZK546.4, ZK593.3, ZK596.3, ZK637.3, ZK666.12, ZK688.10, ZK688.11, ZK688.7, ZK697.1, ZK697.3, ZK721.1, ZK783.7, ZK795.2, ZK809.8, ZK836.3, ZK84.2, ZK84.4, ZK856.14, ZK863.1, ZK863.8, ZK892.5, ZK930.6, ZK971.1
