# Supplementary material for: A survey of putative secreted and transmembrane proteins encoded in the C. elegans genome
Source: BMC Genomics. 2012 Jul 23;13:333. doi: 10.1186/1471-2164-13-333 (PMC3534327; doi:10.1186/1471-2164-13-333)
Supplement: Additional file 3 — Lists of putative organelle proteins. [file 1471-2164-13-333-S3.doc]

**Proteins identified as localized to various organelles**

To identify putative organelle proteins we therefore identified homologs of yeast or mouse organelle proteins, where corresponding experimental evidence exists. Mouse organelle proteins with manual experimental evidence were obtained from QuickGO (Binns et al., 2009). Yeast organelle proteins with experimental evidence were obtained from the yeast GO (Gene Ontology) slim data set ([http://www.yeastgenome.org](http://www.yeastgenome.org/)). Inparanoid (Ostlund et al., 2010) was used to identify the *C. elegans* homologs of these genes.

**golgi proteins (514)**

B0024.12, B0024.15, B0205.4, B0212.4, B0218.3, B0244.2, B0261.2, B0272.2, B0281.8, B0285.5, B0303.7, B0336.2, B0416.6, B0432.8, B0464.6, B0507.2, B0511.1, C01G8.5, C03D6.4, C03E10.4, C03G6.19, C03H5.2, C04A11.4, C04F12.10, C05A9.1, C05D10.2, C05D11.4, C05E4.3, C06E1.7, C06G3.10, C07H4.2, C07H6.3, C08A9.1, C08B11.1, C08B11.5, C09B8.4, C09B8.7, C09D8.1, C09G12.1, C09G4.3, C09H6.2, C10C6.6, C10F3.6, C10H11.8, C12D8.10, C13B9.2, C13B9.3, C13G3.3, C15H9.5, C16C2.4, C18B12.2, C18D11.2, C18E9.10, C18G1.8, C18H9.7, C23F12.1, C24H11.7, C25A1.4, C25A1.8, C25B8.3, C25F6.2, C25F6.7, C26H9A.1, C27C12.7, C27H5.4, C27H6.2, C28C12.5, C28C12.7, C28G1.5, C28G1.6, C29H12.2, C30F12.1, C31H5.6, C32D5.9, C32F10.1, C33A11.2, C33D12.1, C33D12.7, C33D9.1, C33H5.14, C34C6.5, C34D4.4, C34G6.4, C35C5.6, C35D10.4, C36B1.1, C36F7.1, C39F7.4, C41C4.10, C42D8.8, C43E11.11, C43E11.8, C43G2.2, C44B7.7, C44C1.3, C44C1.4, C45E1.1, C47A10.1, C49C8.5, C49H3.9, C50F4.14, C51E3.7, C52B11.2, C52B9.4, C52E12.4, C52E4.5, C54G10.4, C55B7.2, C56C10.9, D1014.3, D2013.1, D2021.1, D2030.1, DC2.3, DH11.3, E01F3.1, EGAP9.2, EGAP9.3, F01G4.2, F02C9.3, F02E8.6, F07A5.4, F07F6.4, F08A8.5, F08B4.6, F08F8.8, F08H9.5, F08H9.6, F08H9.7, F08H9.8, F08H9.9, F10B5.8, F10D11.1, F10D7.3, F10F2.1, F10G7.1, F11A10.4, F11A10.6, F11A5.5, F11A6.1, F11H8.4, F12B6.1, F12F6.6, F13B10.1, F13B9.1, F13E6.1, F13E6.6, F14B4.2, F14E5.2, F16B4.8, F17B5.6, F18A12.8, F20B6.2, F20C5.2, F20C5.4, F20D6.3, F20D6.4, F21A10.2, F21D5.2, F21F3.3, F22E10.1, F22E10.2, F22E10.3, F22E10.5, F23H12.1, F25B3.3, F25H8.3, F28A10.9, F28B3.5, F28B4.2, F28F8.6, F28H6.1, F29D11.1, F29G9.3, F30A10.6, F32A6.3, F32B6.8, F32D8.14, F32H2.5, F33D11.12, F33G12.5, F35D6.1, F35G12.1, F35G12.3, F35H10.4, F35H12.3, F35H12.4, F36D4.2, F36D4.3, F36F2.4, F36H2.1, F38E11.5, F39B2.3, F39G3.7, F40E10.6, F41C3.4, F41D3.6, F42E11.1, F43C11.7, F43D9.3, F44A6.1, F44C8.7, F45E1.7, F45G2.4, F45H11.4, F45H7.2, F46C5.8, F47B7.2, F47G9.4, F48E3.1, F52C12.4, F53F10.4, F53G12.1, F54C9.10, F54E7.7, F54F2.1, F54F7.1, F55A11.2, F55A12.7, F55A4.1, F55A4.8, F55A8.2, F55B12.3, F55H2.5, F57A8.2, F57C12.4, F57C12.5, F57F5.1, F57H12.1, F58F12.1, F58F6.4, F58H1.1, F59B10.1, F59B2.7, F59C6.7, F59D12.4, F59E10.3, F59F3.1, F59F3.5, H17B01.1, H20J04.5, H26D21.1, H32C10.3, JC8.10, JC8.3, K02A4.2, K02B12.7, K02B2.1, K02G10.8, K02H8.1, K03E5.1, K03E6.1, K03E6.3, K04F10.4, K04G2.6, K05B2.4, K06A1.1, K06H6.3, K06H6.6, K07A1.8, K07C11.9, K07C5.8, K08E5.3, K08E7.9, K08F8.3, K08H10.4, K08H10.9, K09B11.9, K09E9.2, K10B2.3, K11C4.3, K11C4.4, K11D2.3, K12H4.8, M01D7.2, M01E11.1, M01F1.1, M02A10.2, M02B1.1, M05D6.2, M110.5, M163.3, M88.6, PAR2.4, R01H10.8, R05G6.10, R05H10.2, R05H5.5, R06B9.6, R06C7.7, R07E4.4, R08C7.2, R09B5.11, R107.6, R119.5, R11A5.1, R11A5.7, R11H6.2, R12B2.2, R12B2.5, R12E2.1, R12H7.2, R153.1, R166.1, R173.1, T01B7.3, T01D3.2, T01D3.5, T03F1.12, T03F6.3, T04D1.4, T05A8.4, T05B11.3, T05C12.7, T05E7.1, T05G5.8, T06A4.1, T06A4.3, T07A5.1, T07A5.2, T07D3.4, T09A5.2, T10B11.6, T10F2.5, T10H9.4, T13F2.3, T13F2.8, T13H5.1, T13H5.2, T14G10.5, T17A3.1, T17E9.2, T18H9.7, T19B4.7, T19E7.3, T20G5.1, T21B6.5, T21C9.2, T21D12.9, T21E12.4, T21E8.1, T21E8.2, T21E8.3, T22C8.8, T23D8.8, T23H2.5, T24B1.1, T24D1.1, T24F1.1, T25D10.2, T25G12.2, T25G12.4, T25G12.7, T27A1.5, T27A1.6, T27A10.7, T27C10.6, T28A8.2, T28F2.1, VW02B12L.1, W02B3.4, W02C12.3, W02F12.2, W03C9.3, W03D8.8, W04G5.2, W05H7.3, W06E11.5, W06H8.8, W07B8.5, W07G4.2, W07G4.3, W08D2.1, W09B6.1, W09D10.1, W09G10.4, W09G3.8, Y105C5B.28, Y105E8A.10, Y105E8A.26, Y105E8A.9, Y105E8B.3, Y105E8B.5, Y106G6E.4, Y108G3AL.1, Y110A2AL.14, Y110A7A.12, Y110A7A.20, Y110A7A.6, Y111B2A.16, Y111B2A.17, Y111B2A.22, Y113G7A.3, Y113G7B.16, Y113G7B.24, Y116F11B.12, Y18D10A.13, Y18D10A.6, Y18D10A.9, Y18H1A.9, Y22D7AL.11, Y22F5A.3, Y25C1A.5, Y32F6B.3, Y37A1B.1, Y39A1A.1, Y39B6A.2, Y39G8C.1, Y40B1A.4, Y41C4A.4, Y41D4B.12, Y41E3.7, Y43C5A.2, Y43F4B.7, Y43F8A.3, Y45G12B.2, Y45G5AM.9, Y46E12BL.1, Y46G5A.5, Y47D3A.17, Y47D3B.11, Y47G6A.11, Y47G6A.18, Y47G6A.19, Y47G6A.7, Y47G6A.9, Y47H9C.2, Y48E1B.13, Y48E1C.2, Y48G1A.6, Y48G1C.2, Y48G8AR.1, Y48G9A.4, Y49A3A.1, Y49E10.11, Y49E10.20, Y49E10.23, Y51H4A.3, Y51H7C.6, Y53C12A.3, Y54E10BR.2, Y54E5B.1, Y54F10AM.4, Y54G11A.2, Y54G2A.17, Y54G2A.18, Y55F3AM.14, Y55F3BL.2, Y57A10A.16, Y57E12AL.1, Y57G11C.10, Y57G11C.22, Y57G11C.24, Y57G11C.4, Y59A8B.25, Y59A8B.7, Y59A8B.8, Y59A8B.9, Y59E9AL.7, Y5H2B.1, Y60A3A.19, Y65B4A.3, Y65B4BR.4, Y66H1A.3, Y67D8C.10, Y6B3A.1, Y6B3B.5, Y6D11A.2, Y71F9AL.17, Y71F9AM.4, Y71F9AM.5, Y71G12B.27, Y71H10A.2, Y71H2B.10, Y73B6BL.4, Y73E7A.7, Y76A2A.2, Y76A2B.1, Y76A2B.6, Y87G2A.4, Y92H12A.1, Y95B8A.10, Y97E10AR.6, ZC116.3, ZC155.7, ZC250.3, ZC434.6, ZC434.9, ZC518.2, ZC8.4, ZK1098.10, ZK1098.5, ZK1240.3, ZK1240.5, ZK1240.6, ZK1240.8, ZK1240.9, ZK20.6, ZK256.1, ZK328.4, ZK337.2, ZK370.7, ZK455.7, ZK512.5, ZK546.1, ZK563.2, ZK637.8, ZK783.1, ZK849.2, ZK856.1, ZK856.5, ZK858.3, ZK896.9, ZK930.1

**endoplasmic reticulum proteins (571)**

B0035.14, B0222.6, B0222.7, B0222.8, B0250.9, B0272.2, B0334.11, B0336.11, B0348.4, B0361.8, B0454.6, B0491.1, B0511.1, B0511.12, B0511.13, BE10.2, C01B12.1, C01B4.8, C01H6.4, C02C2.4, C02C6.3, C02F5.9, C04F12.10, C05B10.1, C05D10.3, C06A1.1, C06A1.6, C06A1.7, C06B3.4, C06B3.5, C06E1.3, C06G3.9, C07A12.4, C07A4.3, C07H4.2, C08B11.8, C08E8.2, C08F8.4, C08H9.2, C08H9.3, C09D4.4, C09G4.1, C10C6.5, C10C6.6, C12C8.1, C12D5.7, C14A4.3, C14A6.2, C14B1.1, C14B9.2, C15C8.4, C15F1.6, C15H11.9, C15H9.4, C15H9.6, C16C10.12, C18B12.2, C18D1.2, C18E9.11, C18E9.2, C18H7.3, C18H9.3, C18H9.5, C23H3.4, C24F3.6, C25A1.5, C25E10.2, C27A12.9, C27A7.5, C27H5.5, C28H8.4, C29F4.1, C30F12.1, C31E10.7, C32D5.9, C32F10.1, C33F10.14, C33H5.18, C34B2.10, C34C6.5, C34F6.10, C34F6.2, C34F6.3, C34H4.4, C35A5.3, C36A4.1, C36A4.2, C36A4.3, C36A4.6, C38C10.2, C39E9.7, C39F7.4, C40H1.4, C41C4.4, C41C4.8, C41G7.9, C42C1.15, C43G2.1, C44C10.1, C45H4.17, C45H4.2, C46A5.3, C46H11.2, C47B2.4, C47E12.3, C48A7.1, C49C8.4, C50C3.9, C50D2.2, C50D2.4, C50F7.10, C50H11.15, C52D10.13, C53B4.4, C53B4.5, C53B4.6, C54D1.5, C54G7.2, C55B6.2, C56G2.6, D1009.1, D1014.3, D2013.8, D2024.2, D2024.3, D2024.8, D2045.9, D2062.1, D2085.6, D2092.5, D2096.2, E01F3.1, E02H9.5, E03E2.1, E03G2.4, EGAP7.1, F01G4.2, F02C12.5, F02E11.1, F08C6.2, F09B9.3, F09E5.2, F10C2.7, F10D2.9, F10D7.3, F10F2.1, F11A10.1, F11A5.12, F11A5.9, F11E6.5, F11G11.11, F11G11.12, F12B6.2, F13D12.6, F13H10.4, F14D12.5, F14F7.2, F14F7.3, F14H12.1, F15A2.1, F15B10.1, F15C11.2, F15H10.1, F15H10.2, F17C11.7, F19B6.1, F19B6.2, F19C7.7, F20C5.4, F21F3.3, F21F8.11, F22E10.5, F23F1.6, F23H12.4, F25B4.6, F25D7.1, F25D7.2, F25G6.5, F25G6.7, F25G6.8, F26B1.4, F26E4.11, F26H9.8, F27C8.6, F28B12.3, F28C6.4, F28D1.9, F29D11.1, F30A10.6, F30B5.1, F31C3.1, F32A5.3, F32B4.6, F32D8.14, F32D8.6, F33D11.11, F33D11.9, F33D4.2, F35B12.2, F35C8.5, F35G2.4, F35H12.3, F36A4.10, F36A4.6, F36H1.1, F38A1.8, F38A3.1, F38A3.2, F38B6.5, F39H11.5, F40E10.3, F40F9.6, F41B5.2, F41B5.3, F41B5.4, F41B5.7, F41C3.2, F41C3.4, F41C3.5, F41C6.5, F41H10.7, F41H10.8, F42A6.4, F42A9.5, F42G9.2, F43D9.3, F43E2.8, F43G6.5, F43H9.2, F44A6.1, F44B9.5, F44C8.1, F44E7.7, F45D3.5, F45E4.11, F46C3.1, F46C8.2, F46C8.6, F46E10.9, F48E3.3, F48F5.5, F49E12.10, F49E12.9, F49H12.6, F52B11.4, F52D1.1, F52H3.1, F53F4.5, F53G12.7, F54A5.3, F54C9.4, F54D1.3, F54F2.9, F54H5.3, F55A11.3, F55A3.1, F55A4.1, F55B12.3, F55C10.2, F55C10.3, F56A4.10, F56A4.11, F56A4.12, F56A8.7, F56B6.4, F56H11.3, F56H11.4, F57A8.2, F57B1.3, F57B1.4, F57B10.10, F57B7.3, F57C2.5, F58F6.2, F59A1.13, F59E12.12, F59E12.4, F59E12.5, H02I12.8, H06O01.1, H11E01.2, H13N06.5, H14E04.1, H17B01.4, H19N07.4, H20J04.2, H24K24.5, H27M09.4, H37A05.1, H43I07.3, K01G5.6, K02B12.3, K02D7.3, K02G10.6, K05C4.1, K05D4.4, K06G5.2, K07A1.16, K07B1.4, K07E3.8, K07G5.1, K08C7.1, K08C7.2, K08C7.5, K08C9.4, K08D12.1, K08D12.3, K08F4.3, K08H10.4, K09A9.6, K09E4.2, K09E9.2, K10B2.2, K11C4.5, K11D9.2, K11G9.5, K12H4.4, M01A10.3, M01E11.1, M02B7.4, M03F4.7, M05B5.4, M110.1, M110.7, M162.5, M18.1, M195.1, M4.1, R01B10.4, R05D7.4, R05F9.1, R05H5.5, R06C1.2, R07E3.5, R08C7.2, R08F11.3, R10D12.1, R10D12.12, R151.6, R186.3, R74.3, T01G1.4, T01H3.3, T02B11.6, T03F6.6, T03G11.4, T04G9.5, T04H1.1, T05A8.4, T05E11.3, T05E11.5, T05E11.6, T05F1.1, T05G5.3, T05G5.5, T05H4.1, T05H4.4, T05H4.5, T06E8.1, T07A9.3, T07H6.2, T07H6.3, T09A5.11, T09B4.1, T09B9.1, T09B9.2, T09E8.3, T10B9.1, T10B9.10, T10B9.2, T10B9.3, T10B9.4, T10B9.5, T10B9.7, T10B9.8, T10E10.1, T10E10.2, T10E10.5, T10E10.6, T10E10.7, T11F9.9, T12A2.2, T13B5.4, T13F2.8, T13H5.8, T14B4.6, T14B4.7, T14G10.7, T14G8.3, T15B7.2, T15B7.3, T15H9.6, T15H9.7, T19B10.1, T19B10.8, T19D12.10, T19D12.9, T20F5.2, T21B4.2, T21D12.2, T22C1.3, T22C1.7, T22D1.4, T22D1.9, T22F3.10, T22F3.11, T22F3.7, T22F3.8, T22G5.5, T24D1.4, T25E12.5, T26A5.1, T26A5.4, T27D12.1, T27E4.2, T27E4.8, T27E4.9, T27E9.5, T27F6.6, T27F7.3, T28C6.4, T28F2.5, T28F3.3, T28F3.4, T28H11.8, VZK822L.1, W01A11.2, W01B6.7, W02B12.15, W02D3.1, W02D7.7, W02F12.2, W03F8.5, W03G11.1, W04C9.6, W05B2.1, W05B2.5, W05B2.6, W05G11.3, W06A7.3, W06D12.3, W07A12.5, W08F4.3, W09B12.1, W09B6.1, W09G3.8, Y105E8A.3, Y105E8B.8, Y106G6E.6, Y110A2AL.12, Y110A2AM.3, Y110A7A.11, Y11D7A.11, Y15E3A.4, Y17D7A.4, Y17G9B.3, Y18D10A.17, Y18D10A.19, Y18H1A.11, Y19D10A.10, Y19D10A.11, Y19D10A.4, Y19D10A.8, Y32F6A.3, Y32F6B.3, Y32H12A.5, Y34D9A.10, Y37A1B.12, Y37A1B.13, Y38A10A.5, Y38A8.2, Y38C1BA.3, Y38F2AR.2, Y38H6C.16, Y39A1A.19, Y39D8C.1, Y40D12A.2, Y41C4A.16, Y41C4A.19, Y41D4A.2, Y41E3.2, Y42G9A.6, Y42H9B.1, Y43F8A.3, Y46H3A.2, Y46H3A.3, Y47D3A.11, Y47D3A.17, Y47D3B.10, Y47D3B.11, Y47H9C.2, Y47H9C.5, Y48G8AL.11, Y49A3A.1, Y49C4A.9, Y49F6B.10, Y4C6B.3, Y4C6B.4, Y50D4C.4, Y51A2D.19, Y51B9A.6, Y51H4A.9, Y52B11A.3, Y53F4B.2, Y54E10BL.2, Y54E10BL.4, Y54E10BR.1, Y54E10BR.5, Y54G2A.18, Y54G2A.23, Y55B1BM.1, Y55F3BL.2, Y56A3A.21, Y56A3A.36, Y57A10A.11, Y57G11C.15, Y57G11C.17, Y57G7A.10, Y59A8B.25, Y59A8B.8, Y59E9AL.7, Y5H2B.6, Y60A3A.10, Y60A3A.14, Y62E10A.10, Y63D3A.6, Y66H1A.2, Y66H1B.4, Y67D2.1, Y67D2.2, Y67D8C.10, Y6B3B.11, Y71F9AM.4, Y71F9AM.6, Y71F9B.3, Y71H10A.1, Y73B6BL.34, Y73F8A.1, Y80D3A.5, Y87G2A.9, ZC101.3, ZC116.3, ZC373.7, ZC410.3, ZC434.6, ZC449.6, ZC506.1, ZC506.3, ZC513.5, ZC513.8, ZC8.1, ZK1010.7, ZK1058.2, ZK1058.4, ZK1098.10, ZK1290.3, ZK1307.8, ZK1320.4, ZK1320.7, ZK1321.3, ZK180.3, ZK180.4, ZK265.9, ZK370.4, ZK40.1, ZK54.1, ZK616.6, ZK632.6, ZK637.5, ZK682.2, ZK686.3

**“vesicle” proteins (833)**

B0024.10, B0024.12, B0024.13, B0024.15, B0025.1, B0025.2, B0034.3, B0035.4, B0035.5, B0207.6, B0218.3, B0244.2, B0261.2, B0272.4, B0280.1, B0281.8, B0303.9, B0336.2, B0361.10, B0361.7, B0395.3, B0414.2, B0454.6, B0464.6, B0491.1, B0511.6, B0513.9, C01B10.9, C01F1.6, C01F6.6, C01G8.2, C01G8.9, C01H6.4, C02C6.1, C02D5.2, C02F4.2, C02F5.9, C03C11.2, C04F12.10, C04F5.3, C05D10.3, C05D11.2, C05D9.1, C05E11.1, C05E4.9, C06A1.1, C06G8.2, C07A4.3, C07G1.5, C07H6.7, C08B11.5, C08G5.4, C08H9.2, C08H9.3, C09D4.4, C09E9.1, C09G12.1, C09G12.8, C09H6.2, C10C6.5, C10C6.6, C10H11.8, C11H1.4, C12D5.7, C13B9.3, C13C4.5, C13F10.5, C13G3.3, C14B9.8, C14F11.1, C15B12.1, C15B12.7, C15B12.8, C15F1.4, C15F1.6, C15H11.3, C15H11.6, C15H11.7, C15H9.4, C15H9.8, C16A3.2, C17C3.1, C17C3.3, C17G1.7, C17H12.14, C18A3.6, C18B12.4, C18C4.10, C18D11.2, C18E3.2, C18E9.10, C18E9.2, C18F3.2, C18H9.3, C23H3.4, C24H11.7, C25B8.3, C25D7.6, C25D7.7, C25E10.2, C25F6.2, C25F6.7, C27A7.5, C27F2.8, C27H2.2, C27H6.1, C27H6.2, C28A5.4, C28C12.10, C28C12.5, C28C12.7, C28G1.3, C28G1.5, C28G1.6, C28H8.4, C28H8.9, C29H12.2, C30A5.5, C30F12.1, C30F8.2, C31B8.8, C31E10.7, C31H5.6, C32D5.3, C32D5.9, C32E12.5, C32E8.10, C33A11.2, C33C12.3, C33C12.8, C33H5.10, C33H5.14, C33H5.9, C34C12.5, C34C6.6, C34D10.1, C34D10.2, C34D4.4, C34E10.4, C34E10.7, C34E11.1, C34F6.8, C34G6.7, C35B8.2, C36A4.9, C36B1.1, C36B1.12, C36B1.4, C36F7.4, C37F5.1, C37H5.13, C39E9.10, C39E9.11, C39E9.13, C39F7.4, C40C9.5, C40H1.4, C41C4.5, C41C4.7, C41C4.8, C42D8.2, C42D8.8, C43E11.8, C44B7.8, C44B7.9, C44C1.3, C44C1.4, C44C1.5, C44E4.3, C45H4.17, C45H4.2, C46H11.11, C46H11.2, C47B2.2, C47B2.4, C47C12.6, C47E12.8, C47G2.5, C48A7.2, C48B4.1, C48D1.3, C49C8.4, C49C8.5, C49H3.5, C50D2.2, C50H11.1, C50H11.15, C52B9.4, C52E12.2, C54H2.5, C55B6.2, C55C3.1, C56A3.6, C56C10.1, C56C10.9, CC4.3, CD4.6, D1009.1, D1009.3, D1037.4, D1054.2, D1086.9, D2013.10, D2024.3, D2024.7, D2030.10, D2045.7, D2045.9, D2062.1, D2089.5, D2096.3, DC2.3, E02H4.1, E03H4.8, F01D4.4, F01F1.4, F01G10.10, F01G4.1, F02C9.3, F02E11.1, F02E8.3, F02E8.6, F02E9.7, F07B10.1, F07F6.4, F08A8.1, F08A8.2, F08A8.3, F08A8.4, F08B12.2, F08B4.5, F08F1.5, F09A5.1, F09B12.2, F09B12.3, F09B9.3, F09B9.5, F09C3.1, F09E5.8, F09G2.4, F10B5.4, F10B5.8, F10D2.9, F10D7.3, F10F2.1, F10G7.1, F10G8.3, F11A10.1, F11A10.6, F11E6.1, F11E6.5, F11H8.4, F12F6.6, F13B12.5, F13B9.1, F13B9.8, F13D12.6, F13E6.1, F13H10.4, F15A2.6, F15C11.2, F16B3.1, F16B4.8, F16F9.5, F17C11.7, F17C11.8, F17E5.2, F17H10.3, F18C12.2, F19B6.2, F20B10.1, F20B6.2, F20D12.2, F21F3.3, F21G4.2, F21H11.2, F22G12.4, F23C8.5, F23F1.6, F23H12.1, F25C8.1, F25D7.1, F25D7.3, F25E2.3, F25H2.9, F26E4.11, F26H9.6, F27D9.1, F27E5.1, F28B3.8, F28B4.2, F28C1.2, F28C6.4, F28D1.9, F28F8.6, F29D11.1, F29G9.3, F30A10.3, F30A10.6, F30F8.8, F31E8.2, F32A5.6, F32A6.3, F32A7.3, F32B6.8, F32D8.14, F32D8.6, F33D11.9, F35C8.7, F35E8.11, F35G2.4, F36D4.2, F36F2.4, F36H1.1, F36H2.1, F36H9.3, F37A4.7, F37B12.3, F37H8.5, F38A1.8, F38A6.2, F38B7.1, F38E11.5, F39B2.3, F39G3.7, F39H11.3, F39H11.5, F40E10.3, F40F8.9, F41B5.2, F41B5.3, F41B5.4, F41B5.7, F41C3.3, F41C3.4, F41C3.5, F41E6.5, F41E7.6, F41F3.2, F41G3.4, F41H10.11, F41H10.7, F41H10.8, F42A9.5, F42G8.11, F42G9.7, F42H10.7, F43C11.7, F43D9.3, F43E2.7, F43G9.6, F43H9.2, F44B9.5, F44C4.5, F44C8.1, F45D3.5, F45E1.7, F45G2.4, F45H11.4, F45H7.2, F46C3.1, F46C5.8, F46E10.8, F46F11.5, F47B7.2, F47G4.3, F47G4.7, F47G9.1, F48F5.5, F49E11.1, F49E12.10, F49E12.9, F52C12.4, F52E1.13, F52F12.1, F53A2.4, F53C11.3, F53F10.4, F53F10.5, F53F10.8, F53G12.1, F53G2.7, F53H8.1, F54B3.1, F54C9.1, F54D8.3, F54F2.8, F54G8.3, F54H5.3, F55A12.7, F55A3.1, F55A4.1, F55A4.5, F55C7.7, F55F3.1, F56B6.4, F56D2.7, F56E3.3, F56F3.1, F56F3.4, F56H11.3, F56H11.4, F57A8.2, F57B10.10, F57C12.4, F57C12.5, F57F10.1, F57F5.1, F57H12.1, F58A3.2, F58F9.7, F59A6.1, F59A7.9, F59B8.2, F59D12.4, F59D6.7, F59E10.3, F59E12.4, F59E12.5, F59F4.1, F59G1.3, H06I04.2, H16O14.1, H17B01.1, H17B01.4, H18N23.2, H24K24.5, H25P06.2, H32C10.3, H34C03.2, H39E23.3, H43I07.3, JC8.10, K01A11.4, K02A4.2, K02B2.1, K02D7.2, K02F2.6, K02F3.6, K02H8.1, K03E6.1, K03E6.3, K04E7.2, K04F1.15, K05B2.4, K05C4.1, K05D4.4, K06A1.1, K07C5.5, K07D4.3, K08C7.2, K08C7.5, K08D12.1, K08D12.2, K09A9.2, K09B11.9, K09C8.4, K09E9.2, K10B3.10, K10D2.6, K10H10.2, K11C4.4, K11D12.9, K11D2.2, K11D2.3, K11G12.3, K11G12.4, K11H3.1, K12H4.4, K12H6.3, M01A10.2, M01D7.2, M01E11.1, M01F1.7, M02A10.2, M02B7.4, M02B7.5, M03A1.1, M03F8.2, M04C9.5, M106.5, M110.5, M88.5, PAR2.3, PAR2.4, R01H10.1, R01H2.5, R02D3.1, R02E12.6, R04B3.2, R05D11.3, R05D3.7, R05F9.12, R05G6.5, R06C1.3, R06C7.3, R06C7.6, R06F6.2, R06F6.9, R07B1.10, R07B1.12, R07B7.11, R07C3.4, R07E4.4, R07H5.1, R08E5.2, R08F11.3, R09B5.11, R09E10.3, R10D12.12, R10E12.1, R11A5.1, R11A5.2, R11A5.7, R12A1.2, R12C12.2, R12H7.2, R13A1.4, R13A5.1, R151.6, R160.1, R166.1, R173.1, R186.3, T01C8.1, T01C8.7, T01D3.2, T01D3.5, T01G1.3, T01H3.1, T02D1.5, T03D8.1, T03E6.7, T03F1.1, T03F1.12, T03F6.3, T03F6.5, T04C10.1, T05A7.10, T05A7.5, T05B11.3, T05C12.3, T05E11.6, T05E7.1, T05F1.1, T05G5.10, T05G5.5, T05G5.8, T05H10.1, T06A4.1, T06A4.3, T06G6.9, T07A5.2, T07A9.10, T07C4.11, T07C4.9, T08A9.3, T08B1.6, T08D2.1, T09A5.11, T09E8.2, T09E8.3, T10A3.1, T10F2.3, T10F2.5, T10G3.5, T10H9.2, T10H9.4, T11F8.3, T12A2.2, T12E12.4, T13C2.6, T13F2.1, T13F2.3, T13F2.8, T14D7.3, T14F9.3, T14G10.5, T14G10.7, T15B7.2, T15H9.3, T17E9.1, T18D3.3, T19E7.3, T20B3.1, T20B5.1, T20D3.7, T20F5.2, T20G5.1, T20G5.2, T21C9.2, T21E12.4, T22C1.3, T22C8.5, T22D1.4, T22D2.1, T22G5.5, T23D8.2, T23F11.3, T23G5.5, T23H2.5, T24C12.2, T24D1.4, T24F1.1, T25B9.9, T25E12.5, T26A5.9, T27A1.5, T27C10.6, T27C4.4, T27E9.3, T27F6.6, T28H10.3, VM106R.1, VZK822L.1, W01C8.6, W01C9.4, W02B12.10, W02B12.8, W02D3.1, W02D7.7, W02D9.1, W02D9.2, W02F12.2, W02F12.5, W02G9.1, W03C9.3, W03C9.4, W03D8.8, W05H7.3, W06B4.3, W06D12.3, W06D4.5, W06H12.1, W06H8.1, W07A12.4, W07B8.5, W07G4.3, W08D2.4, W08G11.4, W09B6.1, W09D10.1, W09D10.4, W09G10.4, W09G3.8, W09H1.5, W10D5.1, Y102A11A.6, Y105E8A.22, Y105E8A.9, Y105E8B.9, Y106G6H.7, Y110A2AL.12, Y110A2AM.3, Y110A7A.11, Y110A7A.12, Y110A7A.14, Y110A7A.6, Y111B2A.8, Y113G7A.3, Y113G7B.16, Y116A8C.12, Y116A8C.14, Y116A8C.16, Y116A8C.36, Y119D3B.17, Y17D7A.4, Y17G7B.12, Y18D10A.17, Y18D10A.5, Y18H1A.9, Y25C1A.5, Y32F6B.3, Y32H12A.2, Y37D8A.23, Y37E3.15, Y38A10A.5, Y38A8.2, Y38C1AA.3, Y38C1BA.2, Y38F1A.6, Y39A1A.1, Y39A1A.19, Y39B6A.19, Y39B6A.33, Y40G12A.1, Y40G12A.2, Y40H4A.1, Y41D4A.6, Y41D4B.13, Y41D4B.19, Y43C5A.2, Y43F4B.4, Y43F4B.7, Y45G5AM.9, Y46E12BL.1, Y47D3A.15, Y47D3A.17, Y47D3A.22, Y47D3A.29, Y47D3B.10, Y47D3B.11, Y47G6A.11, Y47G6A.19, Y47G6A.7, Y47H9C.2, Y47H9C.4, Y48A6A.1, Y48B6A.12, Y48E1B.1, Y48G10A.1, Y48G1A.5, Y48G1C.2, Y48G1C.4, Y48G8AL.14, Y48G9A.4, Y49C4A.9, Y49E10.11, Y49E10.20, Y4C6B.6, Y50D7A.7, Y51H1A.1, Y51H4A.3, Y53C12A.3, Y53F4B.2, Y53G8B.4, Y54E10BL.4, Y54E10BR.2, Y54E10BR.5, Y54E5A.4, Y54F10AM.4, Y54F10AM.8, Y54F10AR.1, Y54G11A.13, Y54G11A.5, Y54G11A.6, Y54G2A.18, Y55D5A.3, Y55D5A.5, Y55F3AM.12, Y55F3AM.14, Y56A3A.13, Y56A3A.36, Y57A10C.6, Y57G11C.15, Y57G11C.4, Y57G11C.47, Y57G7A.10, Y59E9AL.7, Y5H2B.6, Y60A3A.10, Y60A3A.19, Y60A3A.7, Y60A3A.9, Y62E10A.10, Y63D3A.6, Y65B4BL.5, Y66H1A.2, Y66H1A.3, Y67H2A.7, Y69A2AR.30, Y69A2AR.4, Y69A2AR.6, Y6B3A.1, Y6B3B.11, Y71F9AL.17, Y71G12B.11, Y71H10A.2, Y71H2B.10, Y73B3A.5, Y73B6BL.21, Y73B6BL.29, Y73B6BL.32, Y73E7A.9, Y75B7AL.3, Y76A2A.2, Y76A2B.3, Y76A2B.6, Y77E11A.13, Y77E11A.4, Y87G2A.14, Y87G2A.2, Y87G2A.9, Y92C3B.3, Y97E10AR.7, ZC116.3, ZC190.1, ZC376.5, ZC404.9, ZC434.9, ZC449.6, ZC518.2, ZC8.1, ZC8.4, ZK1086.1, ZK1098.10, ZK1098.5, ZK1128.5, ZK1128.8, ZK1240.3, ZK1240.5, ZK1240.6, ZK1240.8, ZK1240.9, ZK1248.3, ZK1307.5, ZK1320.1, ZK180.3, ZK180.4, ZK256.1, ZK328.4, ZK328.5, ZK370.3, ZK512.5, ZK524.2, ZK593.4, ZK616.6, ZK632.6, ZK637.8, ZK669.2, ZK669.3, ZK686.3, ZK688.9, ZK721.1, ZK757.4, ZK770.1, ZK783.2, ZK795.4, ZK809.7, ZK856.8, ZK899.8, ZK930.1, ZK945.2

**mitochondrial proteins (1368)**

AC3.2, B0024.9, B0035.16, B0035.3, B0041.5, B0041.6, B0205.11, B0205.6, B0212.1, B0218.6, B0218.8, B0228.5, B0250.5, B0261.2, B0261.4, B0272.3, B0303.15, B0303.3, B0334.4, B0334.5, B0334.8, B0336.4, B0361.10, B0361.5, B0365.1, B0395.3, B0432.2, B0432.4, B0432.8, B0454.6, B0478.1, B0491.3, B0511.8, B0513.5, B0513.9, B0546.1, BE0003N10.1, C01A2.3, C01A2.4, C01B10.9, C01B4.8, C01B4.9, C01F1.2, C01G10.10, C01G10.7, C01G8.4, C02B10.1, C02C2.4, C02C6.1, C02F4.2, C02F5.3, C02G6.1, C02G6.2, C03G5.1, C03G6.19, C03H5.4, C04A2.3, C04C3.3, C04E12.7, C04E6.11, C04F12.10, C04F6.3, C04G2.6, C05C10.1, C05C10.3, C05C10.4, C05C8.1, C05D10.3, C05D11.1, C05D11.10, C05D11.11, C05D11.12, C05D9.5, C05E4.1, C05E4.3, C05G5.4, C06A1.1, C06A8.1, C06E1.10, C06G3.11, C06G3.7, C06H2.1, C07B5.4, C07E3.2, C07G1.8, C07H4.2, C08A9.1, C08B6.8, C08F8.2, C09B8.6, C09F9.3, C09H10.3, C10C5.6, C10C6.6, C10E2.6, C10G11.5, C11E4.1, C11E4.2, C13B9.2, C13G5.1, C14A4.14, C14A4.2, C14A6.1, C14A6.2, C14B9.2, C14C10.1, C14F11.1, C14F5.1, C15B12.7, C15F1.6, C15F1.7, C15H11.4, C15H11.7, C15H9.1, C15H9.7, C16A3.10, C16A3.5, C16C10.1, C16C10.10, C16C10.11, C17C3.1, C17C3.3, C17G1.7, C17G10.8, C17H12.14, C18A3.5, C18A3.6, C18B12.4, C18C4.10, C18D1.2, C18E9.4, C18E9.6, C18F3.2, C18H9.3, C18H9.5, C23G10.2, C23H3.7, C23H4.6, C24A11.9, C24A3.4, C25A1.12, C25A1.13, C25A1.7, C25B8.1, C25B8.3, C25H3.9, C26E6.12, C26E6.4, C26E6.6, C26F1.7, C27A7.1, C27A7.3, C27H6.4, C27H6.9, C28C12.5, C28C12.7, C28F5.4, C29A12.3, C29E4.10, C29E4.12, C29E4.8, C29F3.1, C29H12.1, C30C11.1, C30F12.2, C30F12.7, C30H6.6, C31E10.7, C31H5.6, C32D5.3, C32D5.6, C32E12.5, C32F10.8, C33A12.1, C33A12.7, C33F10.12, C33F10.14, C33H5.18, C34B2.6, C34B2.7, C34B2.8, C34C12.6, C34C12.8, C34C6.4, C34D4.14, C34E10.1, C34E10.10, C34E10.11, C34E10.4, C34E10.6, C34F6.8, C35A5.3, C35B8.3, C35D10.1, C35D10.4, C35D10.5, C36A4.9, C36B1.4, C37E2.1, C37F5.1, C37H5.13, C37H5.2, C37H5.3, C37H5.8, C38C10.2, C38C6.2, C39B5.6, C39E9.7, C39F7.4, C41C4.10, C41C4.8, C41D11.8, C41G7.9, C42C1.10, C42C1.5, C43E11.7, C43H6.3, C44B12.2, C44B7.10, C44B7.4, C44B7.8, C44B7.9, C44C1.5, C44E4.6, C45B11.1, C45B11.3, C45G3.3, C46F11.2, C46F4.2, C46H11.3, C47B2.7, C47C12.4, C47D12.6, C47E12.1, C47E12.2, C47E12.4, C47E12.8, C47E8.5, C47G2.3, C48B4.1, C48B4.4, C48E7.3, C49F8.2, C49G7.11, C49H3.10, C50B6.9, C50B8.1, C50B8.3, C50D2.2, C50D2.9, C50F4.12, C50F4.14, C50F7.4, C50H11.1, C52E12.2, C52E4.4, C53B7.4, C54F6.14, C54G10.4, C54G4.8, C55B7.4, C55F2.1, C56C10.8, C56G2.1, CD4.3, CD4.6, D1005.1, D1014.1, D1022.3, D1022.4, D1022.5, D1025.2, D1037.3, D1037.4, D1046.3, D1053.1, D1054.2, D1065.1, D2007.4, D2013.5, D2023.2, D2023.5, D2023.6, D2024.7, D2030.2, D2030.4, D2030.5, D2063.1, D2085.1, D2089.5, D2096.1, D2096.4, DC2.5, DH11.1, DY3.1, E01H11.1, E02A10.1, E02H1.2, E02H1.8, E04A4.4, E04A4.5, E04A4.7, E04F6.15, E04F6.2, E04F6.5, E04F6.7, EEED8.8, EEED8.9, EGAP2.3, F01D5.7, F01D5.8, F01F1.2, F01F1.6, F01G4.2, F01G4.6, F02A9.4, F02E11.1, F02E8.1, F02E8.5, F02H6.5, F07A5.7, F07H5.9, F08A8.1, F08A8.2, F08A8.3, F08A8.4, F08B4.1, F08F3.2, F08F3.4, F08F8.8, F09E10.3, F09E5.15, F09E5.8, F09F7.4, F09G8.3, F10C2.6, F10C2.7, F10D11.1, F10E7.7, F10G7.2, F10G7.4, F10G8.9, F11A10.1, F11A10.3, F11A5.9, F11C1.5, F11D5.3, F11G11.7, F11H8.4, F12B6.1, F12B6.2, F12F6.5, F13B10.1, F13B9.8, F13D12.2, F13D12.4, F13D12.6, F13G3.11, F13G3.7, F14B4.2, F14B8.7, F14F4.3, F15A2.2, F15A4.11, F15A4.12, F15D3.1, F15D3.6, F15D3.7, F15D4.1, F15D4.3, F15H10.3, F17A9.4, F17A9.5, F17C11.9, F17E5.2, F17H10.1, F19B6.4, F19C6.1, F19H8.1, F20C5.2, F20D1.9, F20D6.11, F20D6.3, F20D6.4, F20H11.3, F21D5.8, F21F8.11, F21H11.2, F22B7.5, F22B8.7, F22D6.3, F22D6.4, F22E10.5, F22F7.1, F22F7.2, F23B12.5, F23B12.9, F23C8.5, F23F1.6, F23F12.6, F23H11.3, F23H11.9, F23H12.2, F25B4.1, F25B4.6, F25B4.7, F25B5.3, F25B5.6, F25C8.1, F25E2.3, F25E2.4, F25G6.7, F25H2.11, F25H2.4, F25H2.5, F25H2.6, F25H2.9, F25H5.3, F25H5.6, F25H9.7, F26C11.1, F26D10.3, F26D10.9, F26E4.9, F26F2.7, F26F4.10, F26H11.5, F26H9.4, F26H9.6, F27C1.7, F27D4.1, F27D4.5, F27D9.1, F27D9.5, F27D9.6, F28A10.6, F28B3.9, F28C6.6, F28C6.8, F28F8.2, F28H1.3, F28H6.3, F29B9.10, F29C12.4, F29C4.1, F29C4.6, F30A10.10, F30A10.5, F30A10.6, F30F8.9, F31C3.3, F31E8.2, F32A5.8, F32A6.3, F32A7.4, F32B4.2, F32B4.6, F32B5.1, F32B6.2, F32B6.8, F32D1.2, F32D8.12, F32H2.5, F33A8.5, F33D4.5, F33D4.8, F33H1.2, F33H2.6, F35G12.1, F35G12.10, F35G12.2, F35G2.4, F36A4.7, F36D4.3, F36H1.6, F36H12.11, F36H9.3, F37B4.2, F37C12.3, F37C12.7, F37C12.9, F37F2.2, F37F2.3, F38A5.13, F38B2.4, F38B6.4, F38H4.8, F39B2.11, F39B2.7, F39G3.1, F39H2.3, F40E10.3, F40F9.10, F40F9.6, F40G9.2, F41C3.2, F41C3.3, F41C3.5, F41E6.5, F41E7.6, F41G3.4, F42A8.2, F42A9.2, F42D1.2, F42G8.12, F42G9.5, F42H10.6, F43C1.2, F43C1.6, F43E2.5, F43E2.7, F43G9.1, F43G9.3, F43H9.2, F44C4.5, F44E2.6, F44E7.4, F44E7.7, F44E7.9, F44G3.2, F45E4.11, F45E4.9, F45F2.5, F45F2.6, F45F2.7, F45G2.3, F45G2.8, F45G2.9, F45H10.1, F45H10.2, F45H10.3, F45H11.5, F46A8.10, F46B6.6, F46C5.9, F46E10.10, F46G10.3, F46G10.7, F46G11.1, F46H5.3, F46H5.4, F47B10.1, F47D12.4, F47G4.3, F48E8.3, F49E10.1, F49E8.3, F49E8.5, F49H12.6, F52A8.5, F52C9.3, F52D1.1, F52D10.3, F52E1.13, F52E1.7, F52E4.1, F52F12.1, F52F12.7, F52H2.6, F52H3.2, F53A2.7, F53A3.7, F53C11.3, F53E10.1, F53E10.6, F53F10.3, F53F4.10, F53G12.1, F53H8.3, F54A3.5, F54B3.3, F54C4.1, F54C8.1, F54C9.1, F54C9.6, F54D5.12, F54D5.14, F54D5.7, F54D8.2, F54D8.3, F54E12.2, F54E7.1, F54F2.2, F54H12.1, F55A11.4, F55A4.1, F55B12.4, F55C5.5, F55F10.1, F55G1.5, F56A11.5, F56A4.10, F56A4.11, F56A4.12, F56B3.11, F56B3.5, F56B3.8, F56C11.3, F56C9.3, F56D1.3, F56D12.1, F56D2.6, F56D5.3, F56H1.5, F56H1.6, F57A10.3, F57B10.10, F57B10.7, F57B9.4, F57C7.2, F57F4.1, F57F5.1, F58A4.5, F58A6.1, F58B3.5, F58E10.3, F58F12.1, F58F9.7, F58G11.1, F59A1.13, F59A2.3, F59A3.3, F59A6.6, F59A7.8, F59A7.9, F59B8.2, F59C6.12, F59C6.5, F59E11.2, F59F4.1, F59G1.7, H06I04.2, H06O01.2, H09G03.2, H11E01.2, H12D21.4, H12D21.7, H13N06.4, H14A12.2, H14E04.1, H17B01.1, H19J13.1, H21P03.1, H22K11.1, H24K24.3, H24K24.4, H25P06.1, H27A22.1, H28O16.1, H32C10.1, H34C03.1, H37N21.1, JC8.5, K01A2.5, K01A6.2, K01C8.6, K01C8.7, K01D12.6, K01G5.10, K01H12.2, K02A2.1, K02A4.1, K02B2.3, K02B7.4, K02C4.3, K02F3.2, K04C1.4, K04D7.2, K04D7.3, K04F1.15, K04G2.2, K04G2.5, K04G7.4, K05B2.4, K05B2.5, K05C4.7, K05F1.3, K05G3.3, K06A1.5, K06A5.6, K06H7.3, K06H7.9, K07A1.10, K07A12.3, K07A3.1, K07A3.3, K07B1.2, K07B1.3, K07C5.1, K07D4.3, K07E3.3, K07E3.4, K07E8.7, K07G5.5, K07H8.2, K08A8.3, K08B4.3, K08B4.4, K08C7.1, K08D10.2, K08D10.7, K08D10.8, K08E4.1, K08F11.4, K08F11.5, K08F4.1, K09A9.5, K09E4.3, K09H11.1, K10B3.7, K10B3.8, K10B3.9, K10D2.6, K10H10.2, K10H10.3, K10H10.6, K11C4.3, K11C4.4, K11D2.5, K11G12.3, K11G12.4, K11G12.5, K11G9.5, K11H12.1, K11H12.8, K11H3.1, K11H3.3, K11H3.6, K12D12.1, K12G11.3, K12G11.4, LLC1.3, M01B12.3, M01B2.10, M01B2.12, M01E5.2, M01E5.5, M01F1.3, M01F1.9, M02A10.2, M02B7.3, M03A1.1, M03A1.6, M03B6.2, M03C11.5, M03C11.8, M04B2.4, M05B5.4, M05D6.7, M106.1, M106.3, M110.4, M117.2, M142.6, M153.1, M162.5, M7.5, M88.1, M88.2, M88.6, M88.7, MTCE.11, MTCE.12, MTCE.16, MTCE.21, MTCE.23, MTCE.25, MTCE.26, MTCE.31, MTCE.34, MTCE.35, PAR2.1, PAR2.3, PDB1.1, R02D3.1, R02F11.3, R02F2.9, R03A10.4, R03G5.1, R03G5.5, R04E5.10, R04F11.2, R05D3.6, R05D7.4, R05G6.10, R05G6.5, R05G6.7, R05H10.5, R06A4.8, R06C1.2, R06C1.3, R06C7.3, R06C7.5, R06F6.4, R06F6.9, R07B1.12, R07B7.10, R07B7.4, R07B7.5, R07C3.4, R07E3.4, R07E3.5, R07E4.3, R07E4.6, R07E5.13, R07H5.2, R08C7.2, R08E5.2, R09B5.11, R09E10.3, R09H10.3, R106.2, R107.2, R107.7, R10D12.1, R10E4.5, R10H10.1, R10H10.6, R11.1, R119.2, R11A5.4, R11A8.5, R11D1.11, R11D1.9, R11F4.1, R11H6.1, R12C12.1, R12E2.12, R12H7.2, R13D11.4, R13H4.4, R13H9.1, R144.13, R151.7, R160.1, R17.2, R186.6, R53.4, R53.5, T01B11.2, T01B11.4, T01C8.1, T01E8.3, T01E8.6, T01H3.3, T01H8.1, T02B11.6, T02D1.5, T02E1.5, T02G5.12, T02G5.4, T02G5.8, T02G5.9, T02H6.11, T03D8.2, T03F1.10, T03F1.2, T03F1.3, T03F1.7, T03F6.1, T03F6.2, T03F7.1, T03F7.7, T04A8.11, T04A8.14, T04F8.1, T04H1.1, T04H1.4, T05A1.3, T05A12.2, T05C12.3, T05D4.1, T05E7.1, T05F1.10, T05F1.8, T05G5.10, T05G5.5, T05G5.6, T05G5.7, T05H10.2, T05H10.6, T05H4.12, T05H4.13, T05H4.4, T05H4.5, T06D8.3, T06D8.5, T06D8.6, T07A9.11, T07A9.2, T07C4.7, T07C4.8, T07D3.9, T08B1.3, T08B1.6, T08B2.7, T08D2.1, T08G11.1, T08G2.3, T09A12.2, T09A5.7, T09A5.9, T09B4.9, T09B9.2, T09E8.3, T09F3.2, T09F3.3, T10B10.2, T10B11.1, T10B11.2, T10B5.5, T10B5.8, T10E9.7, T10E9.9, T10F2.1, T10F2.2, T10H4.4, T10H9.4, T11F9.11, T11G6.1, T12A2.15, T12A7.1, T12D8.6, T12E12.4, T13C5.8, T13H5.5, T14B4.2, T14D7.1, T14G10.1, T14G11.3, T14G12.4, T16D1.2, T17E9.1, T18D3.9, T19B4.4, T19D12.10, T19D12.9, T19D2.2, T20B12.1, T20B3.1, T20D3.5, T20F5.3, T20G5.1, T20G5.2, T20H4.4, T20H4.5, T21B10.2, T21B6.5, T21C9.12, T21F4.1, T21H8.1, T22B11.5, T22F3.10, T22F3.11, T22F3.7, T22F3.8, T22H2.5, T22H6.2, T23B12.2, T23B12.3, T23B3.3, T23B5.4, T23F4.1, T23F4.3, T23G5.1, T23G5.5, T23H2.3, T23H2.5, T24C4.1, T24H7.1, T24H7.5, T25B9.1, T25B9.9, T25C8.1, T25D3.2, T25G12.5, T25G3.4, T26A5.3, T27C10.6, T27D12.1, T27E9.1, T27E9.2, T27E9.7, T27F6.5, T27F6.6, T28A11.11, T28A8.7, T28F3.1, T28F3.4, T28H11.8, VF11C1L.1, VW06B3R.1, W01A8.4, W01C9.4, W02A11.2, W02B12.15, W02B12.9, W02D3.12, W02D3.2, W02D3.6, W02F12.5, W02G9.1, W03B1.4, W03C9.3, W03D8.8, W03F8.3, W03G11.3, W04C9.6, W04D2.5, W04E12.6, W04E12.8, W04G3.4, W04G5.2, W05E10.4, W05G11.6, W06B11.2, W06B3.1, W06H3.1, W06H3.2, W06H3.3, W06H8.2, W07B8.5, W07E11.1, W08D2.1, W09B6.1, W09C3.4, W09C5.8, W09D10.2, W09D10.3, W09D10.4, W09D6.6, W09G3.7, W09H1.5, W10C8.2, W10C8.5, W10D5.2, W10D9.5, Y105C5B.28, Y105E8A.20, Y105E8A.22, Y105E8A.23, Y106G6E.4, Y106G6E.6, Y106G6H.5, Y106G6H.8, Y10G11A.1, Y110A7A.14, Y110A7A.19, Y110A7A.8, Y111B2A.8, Y113G7A.8, Y113G7A.9, Y113G7B.16, Y116A8C.27, Y119C1B.4, Y119D3B.14, Y119D3B.16, Y119D3B.17, Y15E3A.4, Y17G7B.10, Y17G7B.3, Y17G7B.7, Y17G9B.5, Y18D10A.13, Y18D10A.16, Y18D10A.19, Y18D10A.3, Y18D10A.5, Y18H1A.3, Y18H1A.6, Y19D10A.10, Y19D10A.11, Y19D10A.12, Y19D10A.4, Y19D10A.8, Y22D7AL.10, Y22D7AL.5, Y25C1A.13, Y25C1A.7, Y32B12A.3, Y32G9A.4, Y32H12A.3, Y32H12A.7, Y34D9A.1, Y34D9A.6, Y37D8A.1, Y37D8A.14, Y37D8A.18, Y37D8A.23, Y37E11AR.3, Y37E11AR.4, Y37E3.15, Y37E3.17, Y37E3.9, Y37H9A.6, Y38A8.1, Y38C1AA.1, Y38C1AA.11, Y38F1A.6, Y38F2AR.3, Y38F2AR.7, Y39A1A.11, Y39A1A.12, Y39A1A.15, Y39A1A.22, Y39A1A.6, Y39A3CR.4, Y39B6A.34, Y39B6A.39, Y39D8C.1, Y39E4A.3, Y39G10AL.3, Y39G10AR.3, Y39G8B.1, Y39G8B.2, Y40B10A.2, Y40B10A.6, Y40B10A.7, Y40B1A.4, Y40B1B.8, Y40H4A.1, Y41D4A.5, Y41D4A.6, Y41E3.18, Y41E3.4, Y42A5A.5, Y42G9A.6, Y43C5B.3, Y43F8C.8, Y43H11AL.2, Y44A6D.5, Y45F10D.11, Y45F10D.4, Y45F3A.9, Y45G12B.1, Y45G12B.3, Y45G12C.2, Y46G5A.17, Y46G5A.2, Y46G5A.31, Y46G5A.35, Y46G5A.5, Y46H3A.7, Y47D3A.16, Y47D3A.25, Y47D3A.29, Y47D3B.10, Y47G6A.10, Y47G6A.27, Y47G6A.8, Y48A6B.9, Y48B6A.11, Y48B6A.12, Y48B6A.3, Y48B6A.6, Y48C3A.17, Y48C3A.18, Y48E1B.13, Y48E1B.2, Y48E1B.5, Y48G1A.4, Y48G1C.12, Y48G1C.4, Y48G8AL.11, Y48G9A.3, Y48G9A.4, Y49A3A.1, Y49A3A.2, Y49A3A.5, Y49E10.2, Y49E10.22, Y49F6B.1, Y49F6B.4, Y4C6B.3, Y4C6B.4, Y50D4C.2, Y50D7A.10, Y50D7A.4, Y50D7A.6, Y50D7A.7, Y50D7A.9, Y50E8A.6, Y50E8A.9, Y51B9A.6, Y51H1A.3, Y51H4A.3, Y53C10A.12, Y53C10A.9, Y53F4B.39, Y53G8AL.2, Y53G8AR.2, Y53G8B.1, Y54E10A.14, Y54E10A.2, Y54E10A.7, Y54E10BL.5, Y54E10BR.3, Y54E5A.6, Y54F10AM.2, Y54F10AM.5, Y54F10BM.2, Y54G11A.13, Y54G11A.5, Y54G11A.6, Y54G11A.8, Y54G11A.9, Y54G2A.2, Y55B1BR.4, Y55D5A.5, Y55F3AM.1, Y55F3AM.14, Y55F3AM.8, Y55F3AR.1, Y56A3A.13, Y56A3A.19, Y56A3A.22, Y56A3A.28, Y56A3A.29, Y56A3A.32, Y57A10A.15, Y57A10A.29, Y57A10C.6, Y57G11A.4, Y57G11C.11, Y57G11C.12, Y57G11C.16, Y57G11C.22, Y57G11C.34, Y57G7A.10, Y60A3A.13, Y60A3A.7, Y60A3A.9, Y61A9LA.10, Y62E10A.6, Y63D3A.5, Y63D3A.6, Y63D3A.7, Y65B4BL.5, Y65B4BR.4, Y66D12A.22, Y66D12A.23, Y66D12A.7, Y66H1A.2, Y66H1A.3, Y67A10A.7, Y67D2.4, Y67H2A.1, Y67H2A.4, Y67H2A.7, Y69A2AR.18, Y69A2AR.4, Y70C5C.1, Y71F9AL.13, Y71F9AL.5, Y71F9B.2, Y71G12B.10, Y71G12B.24, Y71H10A.1, Y71H2AM.11, Y71H2AM.23, Y71H2AM.3, Y71H2AM.4, Y71H2AM.5, Y71H2AM.6, Y71H2AM.8, Y71H2AM.9, Y73B3A.5, Y73B6A.5, Y73B6BL.33, Y73C8C.10, Y73C8C.2, Y73F8A.27, Y74C10AR.3, Y75B12B.2, Y75B12B.5, Y75B8A.24, Y76A2B.3, Y76B12C.7, Y77E11A.1, Y82E9BR.3, Y87G2A.2, Y87G2A.5, Y87G2A.8, Y92C3B.3, Y92H12BR.7, Y92H12BR.8, Y94H6A.4, Y94H6A.5, Y94H6A.6, Y94H6A.8, Y9C9A.16, ZC116.2, ZC123.3, ZC262.5, ZC262.8, ZC302.1, ZC376.5, ZC395.2, ZC395.3, ZC395.6, ZC410.2, ZC410.7, ZC434.8, ZC477.3, ZC477.9, ZC518.3, ZC97.1, ZK1010.1, ZK1010.2, ZK1053.5, ZK1058.1, ZK1098.7, ZK1127.7, ZK1128.1, ZK121.1, ZK1236.1, ZK1248.14, ZK1320.1, ZK1320.9, ZK177.5, ZK185.5, ZK20.3, ZK265.9, ZK287.7, ZK370.5, ZK430.1, ZK430.3, ZK455.1, ZK520.5, ZK524.3, ZK54.1, ZK54.2, ZK546.17, ZK550.3, ZK550.4, ZK550.5, ZK550.6, ZK593.1, ZK616.3, ZK637.2, ZK652.10, ZK652.2, ZK652.9, ZK669.4, ZK675.2, ZK682.2, ZK686.3, ZK688.3, ZK697.8, ZK742.3, ZK742.4, ZK757.4, ZK783.2, ZK809.2, ZK816.5, ZK829.4, ZK829.6, ZK856.5, ZK892.4, ZK896.6, ZK896.7, ZK909.2, ZK930.1, ZK945.2, ZK970.2, ZK973.1, ZK973.10, ZK973.3
