# Supplementary material for: A survey of putative secreted and transmembrane proteins encoded in the C. elegans genome
Source: BMC Genomics. 2012 Jul 23;13:333. doi: 10.1186/1471-2164-13-333 (PMC3534327; doi:10.1186/1471-2164-13-333)
Supplement: Additional file 5 — Lists with family members of large secreted protein families. [file 1471-2164-13-333-S5.doc]

**Large families of putative secreted proteins**

Family members of large putative secreted proteins families corresponding to Table 1 and 2 in the main text.

Large families of putative secreted proteins with known domains

| family | no of genes | definition | all members | only members with SP |
| --- | --- | --- | --- | --- |
| small C-type lectins | 236 (184) | small proteins with a CL domain, excluding TM containing and large proteins with additional domains | B0218.6, B0218.8, B0365.5, B0365.6, B0432.12, B0454.7, C03E10.6, C03H5.1, C04H5.2, C07A9.1, C09D1.2, C14A6.1, C16A11.8, C25A1.8, C25G4.1, C29F3.4, C29F3.5, C30H6.1, C30H6.3, C30H6.4, C31G12.2, C33D9.2, C35D10.14, C35D10.15, C41H7.7, C42D4.11, C49A1.9, C49C3.11, C49C3.12, C49C3.13, C50E3.14, C54C8.7, C54D1.2, E03H4.10, EEED8.11, F07C4.2, F07C4.9, F08D12.4, F08G5.7, F08H9.5, F08H9.6, F08H9.7, F08H9.8, F08H9.9, F09G8.8, F10F2.5, F10F2.7, F10F2.8, F10G2.3, F11D11.1, F11D11.11, F11D11.14, F11D11.5, F15D3.2, F16H6.1, F16H6.2, F17B5.10, F17B5.3, F17B5.5, F17C11.5, F19F10.6, F19F10.7, F21H7.4, F22B3.10, F25B4.9, F26A1.11, F26A1.12, F26D10.12, F26D11.5, F26D11.9, F26D2.12, F26F2.6, F29D10.3, F31D4.4, F32E10.3, F33E2.3, F35C5.5, F35C5.6, F35C5.7, F35C5.8, F35C5.9, F35D11.10, F35D11.8, F36D3.10, F36F12.5, F36F12.6, F36G9.11, F36H5.6, F38A1.1, F38A1.10, F38A1.14, F38A1.4, F38A1.5, F38A1.7, F38C2.6, F40G9.10, F47C12.2, F47C12.4, F47F6.5, F47G4.1, F49A5.4, F49A5.7, F49H6.1, F49H6.2, F52E1.2, F56A4.2, F56D6.1, F56D6.15, F56D6.2, F56H6.8, F58A4.5, F59A7.1, H02K04.1, H02K04.2, H16D19.1, K01C8.8, K02F3.5, K03H6.7, K10B2.3, M02F4.7, M162.1, M162.2, M199.3, M199.4, M199.6, R06B10.3, R07C3.1, R07C3.12, R08C7.6, R13F6.8, T03F1.10, T05A7.2, T07D10.4, T07H3.4, T07H3.5, T09F5.9, T15B7.1, T19E7.1, T20B3.12, T20B3.16, T20D3.1, T26C12.6, T26E3.1, T27C5.7, T27D12.3, T27F6.2, W02D7.2, W04E12.6, W04E12.8, W04H10.4, W09G10.5, W09G10.6, W10G11.11, W10G11.12, W10G11.13, W10G11.14, W10G11.15, W10G11.5, W10G11.6, W10G11.7, Y102A5B.2, Y102A5B.3, Y102A5C.16, Y102A5C.17, Y116A8A.3, Y116A8A.8, Y116A8C.21, Y17D7B.6, Y17D7B.8, Y18D10A.10, Y18D10A.12, Y18D10A.24, Y19D10A.9, Y25C1A.1, Y25C1A.3, Y25C1A.4, Y26D4A.2, Y26D4A.4, Y26D4A.6, Y38E10A.4, Y38E10A.5, Y38H6C.8, Y39A1B.1, Y46C8AL.1, Y46C8AL.2, Y46C8AL.3, Y46C8AL.4, Y46C8AL.5, Y46C8AL.8, Y46C8AL.9, Y46C8AR.1, Y46C8AR.3, Y46H3B.1, Y46H3B.2, Y48E1B.16, Y48E1B.9, Y51A2A.1, Y51A2A.11, Y51A2A.7, Y52B11A.5, Y53H1A.3, Y54G2A.14, Y54G2A.33, Y54G2A.39, Y54G2A.6, Y54G2A.8, Y54G2A.9, Y55F3C.13, Y55F3C.5, Y59H11AR.5, Y60A3A.2, Y68A4B.1, Y68A4B.2, Y70C5C.2, Y70C5C.5, Y71A12B.5, Y71A12B.6, Y73C8C.2, ZC15.2, ZC15.6, ZK39.2, ZK39.3, ZK39.4, ZK39.5, ZK39.6, ZK39.7, ZK39.8, ZK666.3, ZK666.5, ZK666.6, ZK666.7, ZK673.9, ZK858.3, ZK863.9, ZK896.6, ZK896.7 | B0218.6, B0218.8, B0365.5, B0365.6, B0432.12, C03E10.6, C03H5.1, C04H5.2, C07A9.1, C14A6.1, C16A11.8, C25A1.8, C25G4.1, C29F3.4, C29F3.5, C30H6.1, C30H6.4, C35D10.14, C35D10.15, C41H7.7, C49C3.11, C49C3.13, C54C8.7, C54D1.2, E03H4.10, EEED8.11, F07C4.2, F07C4.9, F08G5.7, F08H9.5, F08H9.6, F08H9.7, F08H9.8, F08H9.9, F09G8.8, F10F2.8, F10G2.3, F11D11.1, F11D11.11, F11D11.14, F11D11.5, F16H6.1, F17B5.10, F17B5.3, F17B5.5, F17C11.5, F21H7.4, F25B4.9, F26A1.12, F26D10.12, F26D11.5, F26D11.9, F26F2.6, F29D10.3, F31D4.4, F32E10.3, F33E2.3, F35C5.5, F35C5.6, F35C5.7, F35C5.8, F35C5.9, F35D11.10, F35D11.8, F36G9.11, F36H5.6, F38A1.1, F38A1.10, F38A1.14, F38A1.4, F38C2.6, F40G9.10, F47C12.2, F47C12.4, F49A5.4, F49H6.1, F49H6.2, F52E1.2, F56A4.2, F56D6.1, F56D6.15, F56D6.2, F56H6.8, F58A4.5, F59A7.1, H02K04.1, H02K04.2, H16D19.1, K01C8.8, K02F3.5, K03H6.7, K10B2.3, M02F4.7, M162.2, M199.3, M199.4, M199.6, R06B10.3, R07C3.1, R08C7.6, R13F6.8, T03F1.10, T05A7.2, T07D10.4, T07H3.4, T07H3.5, T09F5.9, T15B7.1, T20B3.16, T20D3.1, T26C12.6, T27C5.7, T27D12.3, T27F6.2, W02D7.2, W04E12.6, W04E12.8, W09G10.5, W10G11.11, W10G11.12, W10G11.13, W10G11.14, W10G11.15, Y102A5B.2, Y102A5B.3, Y116A8A.3, Y116A8C.21, Y17D7B.6, Y17D7B.8, Y18D10A.10, Y18D10A.24, Y19D10A.9, Y25C1A.1, Y25C1A.3, Y25C1A.4, Y26D4A.2, Y26D4A.6, Y38E10A.4, Y38E10A.5, Y38H6C.8, Y39A1B.1, Y46C8AL.1, Y46C8AL.2, Y46C8AL.3, Y46C8AL.4, Y46C8AL.5, Y46C8AL.8, Y46C8AL.9, Y46C8AR.1, Y46C8AR.3, Y46H3B.1, Y46H3B.2, Y48E1B.16, Y48E1B.9, Y51A2A.11, Y51A2A.7, Y52B11A.5, Y54G2A.14, Y54G2A.33, Y54G2A.39, Y54G2A.6, Y54G2A.8, Y54G2A.9, Y59H11AR.5, Y68A4B.1, Y70C5C.2, Y70C5C.5, Y71A12B.6, Y73C8C.2, ZC15.2, ZK39.3, ZK39.4, ZK39.6, ZK39.7, ZK39.8, ZK666.3, ZK666.5, ZK666.6, ZK666.7, ZK673.9, ZK858.3, ZK863.9, ZK896.6, ZK896.7 |
| collagens | 168 (108) | collagens excluding excluding TM containing and BM collagens (cle-1, emb-2, let-9) | AC3.6, B0024.1, B0024.2, B0222.6, B0222.7, B0222.8, B0491.2, C01B12.1, C01H6.1, C09G5.3, C09G5.4, C09G5.5, C09G5.6, C12D8.8, C15A11.1, C15A11.5, C15A11.6, C18H7.3, C24F3.6, C27H5.5, C29E4.1, C29F4.1, C30F2.1, C31H2.2, C34D4.15, C34H4.4, C35B8.1, C39E9.3, C39E9.9, C44C10.1, C46A5.3, C50B6.4, C50D2.4, C52D10.13, C53B4.5, D1007.2, D2023.7, D2024.8, E03G2.3, E03G2.4, EGAP7.1, F02D10.1, F08G5.4, F09G8.6, F11G11.11, F11G11.12, F11H8.3, F12F6.9, F14F7.1, F14H12.1, F15A2.1, F15D3.3, F15H10.1, F15H10.2, F17C11.3, F17C8.2, F17E9.1, F19C7.7, F22D6.10, F23H12.4, F26B1.4, F26F12.1, F27C1.8, F29B9.9, F29C4.8, F30B5.1, F32G8.5, F33D11.3, F36A4.10, F36A4.6, F38A3.2, F38B6.5, F41C6.5, F41F3.4, F46B3.17, F46C8.2, F46C8.6, F52B11.4, F52F12.2, F53G12.7, F54B11.2, F54C9.4, F54D1.2, F54D1.3, F54D8.1, F55C10.2, F55C10.3, F56B3.1, F56D5.1, F57B1.3, F57B1.4, F57B7.3, F58F6.1, F58F6.2, F59E12.12, H06A10.2, H27M09.4, K01A2.7, K02D7.3, K03H9.2, K08C9.4, K09H9.3, K12D12.3, M01A12.1, M110.1, M18.1, M195.1, M199.5, R09A8.4, T01B7.7, T05A1.2, T06E4.4, T06E4.6, T07H6.3, T08B2.2, T10B10.1, T10E10.1, T10E10.2, T10E10.5, T10E10.6, T10E10.7, T11B7.3, T11F9.9, T14B4.6, T14B4.7, T15B7.3, T15B7.4, T15B7.5, T21B4.2, T21D12.2, T21D9.1, T28C6.4, T28C6.6, T28F2.6, T28F2.8, W01B6.7, W03G11.1, W05B2.1, W05B2.5, W05B2.6, W05G11.3, W08D2.6, W09G10.1, Y18H1A.12, Y18H1A.13, Y2H9A.3, Y38C1BA.3, Y39G8C.4, Y41C4A.16, Y41C4A.19, Y41D4A.2, Y41E3.2, Y42H9B.1, Y49F6B.10, Y51H4A.9, Y54E10BL.2, Y57A10A.11, Y73B6BL.34, Y77E11A.15, ZC373.7, ZC513.8, ZK1010.7, ZK1193.1, ZK1248.2, ZK1290.3, ZK265.2, ZK836.1, ZK863.2 | AC3.6, B0024.1, B0024.2, B0222.7, B0222.8, B0491.2, C01B12.1, C01H6.1, C09G5.4, C09G5.5, C09G5.6, C12D8.8, C15A11.5, C15A11.6, C18H7.3, C29E4.1, C29F4.1, C30F2.1, C31H2.2, C34H4.4, C35B8.1, C39E9.3, C39E9.9, C46A5.3, C50B6.4, C52D10.13, D2023.7, D2024.8, E03G2.3, E03G2.4, EGAP7.1, F02D10.1, F08G5.4, F09G8.6, F11H8.3, F12F6.9, F14F7.1, F14H12.1, F15A2.1, F17C11.3, F17C8.2, F17E9.1, F19C7.7, F22D6.10, F23H12.4, F26B1.4, F26F12.1, F27C1.8, F29B9.9, F30B5.1, F32G8.5, F33D11.3, F36A4.10, F41F3.4, F46B3.17, F52B11.4, F52F12.2, F54C9.4, F54D8.1, F55C10.2, F55C10.3, F56B3.1, F56D5.1, F57B7.3, F58F6.1, F58F6.2, H06A10.2, H27M09.4, K03H9.2, K08C9.4, K09H9.3, K12D12.3, M110.1, M18.1, T01B7.7, T06E4.4, T06E4.6, T07H6.3, T08B2.2, T10B10.1, T10E10.5, T11B7.3, T11F9.9, T14B4.7, T21D12.2, T21D9.1, T28C6.4, T28C6.6, W05B2.5, W05B2.6, W05G11.3, W08D2.6, Y18H1A.13, Y2H9A.3, Y38C1BA.3, Y39G8C.4, Y41C4A.16, Y42H9B.1, Y54E10BL.2, Y57A10A.11, Y73B6BL.34, Y77E11A.15, ZC373.7, ZC513.8, ZK1193.1, ZK265.2, ZK836.1, ZK863.2 |
| ShK proteins | 66 (52) | TF317956 and TF318899 | C03G6.13, C14C6.2, C14C6.5, C46H11.7, C46H11.8, C46H11.9, C49G7.3, C49G7.4, C54D10.3, E04D5.4, F01D5.1, F01D5.2, F01D5.3, F01D5.5, F07C4.10, F07C4.11, F07C4.6, F15A4.6, F16H6.3, F26D2.13, F26D2.14, F35E8.1, F35E8.10, F35E8.13, F35E8.2, F35E8.4, F35E8.6, F35E8.7, F35E8.9, F41G3.10, F41G3.19, F41G3.20, F41G3.21, F46B3.1, F46B3.2, F48G7.5, F48G7.7, F48G7.8, F49F1.1, F49F1.5, F49F1.6, F49F1.7, K03A11.6, M05D6.8, M163.11, M163.8, T05B4.10, T05B4.11, T05B4.12, T05B4.13, T05B4.3, T05B4.4, T05B4.8, T05B4.9, T06C12.14, T26E4.2, Y39G8B.10, Y39G8B.7, Y39G8B.9, Y46H3D.8, ZK218.1, ZK218.11, ZK218.3, ZK218.5, ZK218.7, ZK673.1 | C14C6.2, C14C6.5, C46H11.7, C46H11.8, C46H11.9, C49G7.3, C49G7.4, C54D10.3, E04D5.4, F01D5.1, F01D5.2, F01D5.3, F01D5.5, F07C4.10, F07C4.6, F15A4.6, F16H6.3, F26D2.13, F26D2.14, F35E8.1, F35E8.10, F35E8.13, F35E8.6, F35E8.7, F35E8.9, F41G3.20, F41G3.21, F46B3.1, F46B3.2, F48G7.7, F48G7.8, F49F1.5, F49F1.6, F49F1.7, K03A11.6, M05D6.8, M163.8, T05B4.10, T05B4.11, T05B4.9, T06C12.14, T26E4.2, Y39G8B.10, Y39G8B.7, Y39G8B.9, Y46H3D.8, ZK218.1, ZK218.11, ZK218.3, ZK218.5, ZK218.7, ZK673.1 |
| Transthyretin -like | 56 (46) | ttr-genes, TF315773 | B0334.1, C04G2.1, C12D8.4, C14C10.7, C33A12.15, C37C3.13, C40H1.5, C56A3.2, E02C12.13, E02C12.4, F09A5.9, F09F3.6, F10G7.11, F19F10.4, F21E9.3, F22A3.2, F22A3.7, F35G8.2, F36A4.8, F40F12.1, F46B3.18, F46B3.3, F46B3.4, F56F4.1, F56F4.2, F56F4.9, F58A3.5, F58B3.9, H01M10.3, H14N18.3, JC8.14, JC8.8, K03H1.3, K03H1.4, K03H1.6, R13A5.3, R13A5.6, R90.2, R90.3, R90.4, T04H1.3, T05A10.3, T07C12.7, T07C4.5, T08A9.11, T08A9.2, T19C3.9, T21C9.8, T28B4.3, Y44A6B.4, Y51A2D.10, Y51A2D.11, Y51A2D.9, Y5F2A.1, Y5F2A.2, ZC64.2 | B0334.1, C04G2.1, C12D8.4, C14C10.7, C33A12.15, C37C3.13, C56A3.2, E02C12.13, E02C12.4, F09F3.6, F10G7.11, F19F10.4, F21E9.3, F22A3.2, F22A3.7, F35G8.2, F36A4.8, F46B3.3, F46B3.4, F56F4.9, H01M10.3, H14N18.3, JC8.14, JC8.8, K03H1.3, K03H1.4, K03H1.6, R13A5.3, R13A5.6, R90.2, T04H1.3, T05A10.3, T07C12.7, T07C4.5, T08A9.11, T08A9.2, T19C3.9, T21C9.8, T28B4.3, Y44A6B.4, Y51A2D.10, Y51A2D.11, Y51A2D.9, Y5F2A.1, Y5F2A.2, ZC64.2 |
| RcpL-genes | 51 (38) | (TF317243 + others, excluding TM containing proteins like let-23, daf-2) | C04G2.11, C17E7.10, C30G4.6, C31E10.3, C41G6.6, F02C12.4, F11A5.7, F14D2.17, F14D2.6, F15E11.2, F15E11.3, F15E11.4, F15E11.5, F45C12.1, F45C12.16, F54G8.1, F56A4.9, F58E1.4, F58E1.7, F59D6.4, H25K10.5, H25K10.6, K04F1.10, K04F1.11, K04F1.12, K04F1.13, K04F1.14, K04F1.6, K04F1.7, K12D9.12, K12D9.14, T05A6.4, T11F1.2, T11F1.6, T11F1.7, T11F1.8, T11F1.9, T26E4.1, Y19D10A.7, Y19D10B.2, Y37A1B.8, Y37A1B.9, Y70C5C.3, Y73F8A.18, ZC482.2, ZC482.4, ZK1037.1, ZK355.1, ZK355.4, ZK355.5, ZK355.6 | C17E7.10, C30G4.6, C41G6.6, F02C12.4, F11A5.7, F15E11.2, F15E11.4, F15E11.5, F45C12.16, F54G8.1, F56A4.9, F58E1.4, F58E1.7, H25K10.5, K04F1.10, K04F1.11, K04F1.13, K04F1.14, K04F1.7, K12D9.12, K12D9.14, T05A6.4, T11F1.2, T11F1.6, T11F1.8, T11F1.9, T26E4.1, Y19D10A.7, Y37A1B.8, Y37A1B.9, Y70C5C.3, Y73F8A.18, ZC482.2, ZC482.4, ZK1037.1, ZK355.1, ZK355.4, ZK355.5 |
|  |  |  |  |  |
| small CUB proteins | 43 (38) | small proteins containing a CUB domain and no other domain | C17H12.6, C17H12.8, C32H11.1, C32H11.10, C32H11.12, C32H11.13, C32H11.3, C32H11.4, C32H11.9, C49G7.7, E02H4.4, F08G5.6, F10A3.4, F20G2.5, F35E12.10, F35E12.2, F35E12.5, F35E12.6, F35E12.7, F53C11.1, F54B11.11, F55G11.2, F55G11.4, F55G11.5, F55G11.6, F55G11.7, F55G11.8, H20E11.2, H20E11.3, K08D8.3, K08D8.4, K08D8.5, K08D8.6, K10D11.1, K10D11.2, K10D11.3, K10D11.4, K10D11.6, T05A7.3, T05E12.6, ZK896.1, ZK896.4, ZK896.5 | C17H12.6, C17H12.8, C32H11.1, C32H11.10, C32H11.12, C32H11.3, C32H11.4, C32H11.9, E02H4.4, F08G5.6, F10A3.4, F35E12.10, F35E12.2, F35E12.5, F35E12.6, F35E12.7, F53C11.1, F54B11.11, F55G11.2, F55G11.4, F55G11.5, F55G11.6, F55G11.7, F55G11.8, H20E11.2, H20E11.3, K08D8.3, K08D8.4, K08D8.5, K08D8.6, K10D11.1, K10D11.2, K10D11.3, K10D11.6, T05E12.6, ZK896.1, ZK896.4, ZK896.5 |
| insulins | 39 (37) | insulin genes | C06E2.8, C17C3.18, C17C3.19, C17C3.20, C17C3.4, F08G2.6, F13B12.5, F21E9.4, F41G3.16, F41G3.17, F52B11.6, F56F3.6, K02E2.4, M04D8.1, M04D8.2, M04D8.3, T08G5.12, T10D4.13, T10D4.4, T28B8.2, W09C5.4, Y39A3A.5, Y53H1A.4, Y8A9A.6, ZC334.1, ZC334.10, ZC334.11, ZC334.2, ZC334.3, ZC334.8, ZC334.9, ZK1251.11, ZK1251.2, ZK75.1, ZK75.2, ZK75.3, ZK84.3, ZK84.6, ZK84.7 | C06E2.8, C17C3.18, C17C3.19, C17C3.20, C17C3.4, F08G2.6, F13B12.5, F21E9.4, F41G3.16, F41G3.17, F52B11.6, F56F3.6, K02E2.4, M04D8.1, M04D8.2, M04D8.3, T08G5.12, T10D4.13, T10D4.4, T28B8.2, W09C5.4, Y39A3A.5, Y53H1A.4, Y8A9A.6, ZC334.1, ZC334.10, ZC334.11, ZC334.2, ZC334.3, ZC334.8, ZC334.9, ZK1251.11, ZK1251.2, ZK75.1, ZK75.3, ZK84.3, ZK84.6 |
| grl-genes and  grd-genes | 44/46 | grl- and grd-genes | C04E6.6, C23H5.9, C24G6.7, C26F1.5, C37C3.4, C56A3.1, E02A10.2, F02D8.2, F09D12.1, F11E6.2, F28A12.2, F32D1.4, F40C5.3, F41E6.2, F42C5.7, F46B3.5, F46H5.6, K02D7.6, K02E2.2, K03B8.7, K10C2.5, R02D3.6, R08B4.1, T01B10.1, T01B10.2, T02E9.2, T03D8.4, T05C3.4, T16G1.8, T18H9.1, T24A6.15, T24A6.18, T24A6.19, W03A5.3, W05E7.1, W05E7.3, Y47D7A.5, Y65B4BR.6, Y69A2AL.1, Y75B8A.20, Y87G2A.15, ZC168.5, ZC487.4, ZC487.5, ZK512.9, ZK643.8 | C04E6.6, C23H5.9, C24G6.7, C26F1.5, C37C3.4, C56A3.1, E02A10.2, F02D8.2, F09D12.1, F11E6.2, F28A12.2, F32D1.4, F40C5.3, F41E6.2, F42C5.7, F46B3.5, F46H5.6, K02D7.6, K02E2.2, K03B8.7, K10C2.5, R02D3.6, R08B4.1, T01B10.1, T01B10.2, T02E9.2, T03D8.4, T05C3.4, T16G1.8, T18H9.1, T24A6.15, T24A6.18, T24A6.19, W03A5.3, W05E7.3, Y47D7A.5, Y65B4BR.6, Y69A2AL.1, Y75B8A.20, Y87G2A.15, ZC168.5, ZC487.4, ZC487.5, ZK643.8 |
| scl-genes | 28 (27) | TF352352 and TF316148 | B0545.3, C39E9.2, C39E9.4, C39E9.5, C39E9.6, C50E3.10, F02E11.5, F08E10.7, F09E8.5, F11C7.3, F48E8.1, F49E11.10, F49E11.11, F49E11.4, F49E11.5, F49E11.6, F49E11.9, F58E2.5, H10D18.2, H10D18.4, T05A10.5, T12A7.3, T19C9.5, Y43F8B.5, Y51H7C.2, ZK384.1, ZK384.2, ZK6.3 | B0545.3, C39E9.2, C39E9.4, C39E9.5, C39E9.6, C50E3.10, F02E11.5, F08E10.7, F09E8.5, F11C7.3, F48E8.1, F49E11.10, F49E11.11, F49E11.4, F49E11.5, F49E11.6, F58E2.5, H10D18.2, H10D18.4, T05A10.5, T12A7.3, T19C9.5, Y43F8B.5, Y51H7C.2, ZK384.1, ZK384.2, ZK6.3 |
| Ctx genes | 29 (24) | Ctx domain containing genes | AC3.3, AC3.4, C03A7.14, C03A7.4, C03A7.7, C03A7.8, C14C11.8, C24B5.5, F07H5.8, F19G12.7, F35A5.4, M02G9.1, M02G9.2, M02G9.3, M195.2, R09B5.5, R09F10.2, R09F10.7, T01D1.6, T23F1.6, Y105C5A.3, Y105C5A.4, Y105C5A.5, Y105C5A.6, Y5H2A.3, Y5H2A.4, Y73F8A.8, Y73F8A.9, ZK1067.7 | AC3.3, AC3.4, C03A7.14, C03A7.4, C03A7.7, C03A7.8, C24B5.5, F19G12.7, F35A5.4, M02G9.2, M02G9.3, R09B5.5, R09F10.2, R09F10.7, T01D1.6, T23F1.6, Y105C5A.3, Y105C5A.4, Y105C5A.5, Y105C5A.6, Y5H2A.3, Y73F8A.8, Y73F8A.9, ZK1067.7 |
| VOMI | 20 (13) | Vitelline membrane outer layer protein I | C08G5.3, C18D4.4, C47A10.12, F28A10.8, F46F5.13, K07E8.1, R105.1, T02H6.4, T02H6.7, T02H6.9, T23F4.5, Y37E11AL.4, Y38H6C.19, Y39A3A.3, Y39F10C.1, Y40B10B.1, Y46B2A.3, Y54E2A.9, Y57G11A.2, Y9C9A.1 | C08G5.3, C18D4.4, F28A10.8, K07E8.1, T02H6.4, T02H6.7, T02H6.9, Y37E11AL.4, Y38H6C.19, Y39F10C.1, Y40B10B.1, Y54E2A.9, Y57G11A.2 |
| large KU/DC proteins | 12 (12) | TF315349 | C34F6.1, C54D10.10, F30H5.3, K10D3.4, T22F7.3, W01F3.3, W05B2.2, Y43F8B.3, Y55F3BR.2, ZC84.1, ZC84.6, ZK287.4 | C34F6.1, C54D10.10, F30H5.3, K10D3.4, T22F7.3, W01F3.3, W05B2.2, Y43F8B.3, Y55F3BR.2, ZC84.1, ZC84.6, ZK287.4 |
| spp-genes | 23 (21) | spp-genes | C28C12.5, C28C12.7, C48E7.10, C54G6.5, F08F1.6, F27C8.4, F32D8.9, K04A8.8, K04A8.9, K09F5.3, T07C4.4, T08A9.10, T08A9.12, T08A9.7, T08A9.8, T08A9.9, T22G5.7, T25C12.2, T25C12.4, T25D10.3, Y34D9A.11, ZK616.9, ZK867.3 | C28C12.5, C28C12.7, C48E7.10, C54G6.5, F08F1.6, F27C8.4, F32D8.9, K04A8.8, K09F5.3, T07C4.4, T08A9.12, T08A9.7, T08A9.8, T08A9.9, T22G5.7, T25C12.2, T25C12.4, T25D10.3, Y34D9A.11, ZK616.9, ZK867.3 |
| wrt-genes | 10 (10) | wrt-genes | B0344.2, C29F3.2, F38E11.7, F52E4.6, W03D2.5, ZK1037.10, ZK1290.12, ZK1290.8, ZK377.1, ZK678.5 | B0344.2, C29F3.2, F38E11.7, F52E4.6, W03D2.5, ZK1037.10, ZK1290.12, ZK1290.8, ZK377.1, ZK678.5 |

Large families of putative secreted proteins with novel domains

| family | no of genes | definition | all members | only members with SP |
| --- | --- | --- | --- | --- |
| DUF23 | 65 (42) | DUF23 domain containing proteins (TF315451, TF315323 and others) | C01G5.9, C05C8.8, C08B6.3, C13A2.1, C13A2.5, C13A2.6, C14C6.6, C14C6.7, C14C6.8, C17A2.2, C18G1.6, C18G1.7, C27A7.2, C31B8.7, C33H5.2, C35A5.5, C49A1.5, D1014.5, D1014.6, D1014.7, E03H4.5, F07G11.3, F07G11.4, F13G3.3, F18F11.4, F22F7.3, F22F7.4, F28H7.7, F36F12.1, F36F12.2, F39G3.2, F46F5.7, F49C12.5, F54D10.8, F55C10.4, F59C6.8, K02H11.4, K06H6.4, K08D9.2, K08D9.5, K08D9.6, M03F8.4, R05A10.6, R07B7.12, R08C7.4, T05B11.4, T06A1.1, T06A1.5, T09E11.8, T13H10.2, T15D6.10, T15D6.12, T22D1.1, Y105C5B.25, Y116F11B.9, Y18H1A.14, Y47D3B.1, Y47D3B.4, Y97E10B.1, ZK1025.4, ZK1025.7, ZK381.2, ZK381.8, ZK488.5, ZK488.6 | C01G5.9, C05C8.8, C08B6.3, C14C6.6, C14C6.7, C17A2.2, C18G1.6, C18G1.7, C31B8.7, C33H5.2, C49A1.5, D1014.5, D1014.6, E03H4.5, F07G11.4, F13G3.3, F18F11.4, F28H7.7, F36F12.1, F39G3.2, F46F5.7, F49C12.5, F54D10.8, F55C10.4, F59C6.8, K02H11.4, K08D9.2, M03F8.4, R05A10.6, R08C7.4, T05B11.4, T06A1.1, T06A1.5, T09E11.8, T15D6.10, T15D6.12, Y105C5B.25, Y116F11B.9, Y47D3B.4, Y97E10B.1, ZK1025.7, ZK381.2 |
| nsp-genes | 58 (41) | nsp-genes | C01G12.10, C01G12.11, C01G12.2, C01G12.6, C03G5.10, C03G5.11, C03G5.12, C03G5.13, C03G5.2, C03G5.8, C03G5.9, C24D10.7, C24D10.8, C40H5.1, D1025.4, D1025.6, D1025.7, D1025.8, D1025.9, F09F7.8, F11G11.8, F26A1.10, F35C5.10, F38A5.10, F38A5.12, F38A5.14, F38A5.5, F38A5.9, F42F12.1, F42F12.10, F42F12.6, F42F12.7, F42F12.8, F42F12.9, H04M03.2, H12D21.1, H12D21.12, H12D21.13, H12D21.14, H12D21.15, K07F5.5, T23B7.1, T27C10.7, W06A7.5, Y23H5B.9, Y38E10A.11, Y38E10A.12, Y38E10A.13, Y38E10A.15, Y38E10A.16, Y38E10A.25, Y38E10A.26, Y38E10A.29, Y43C5A.1, Y50E8A.17, ZC412.6, ZC412.7, ZK484.8 | C01G12.10, C01G12.11, C01G12.2, C01G12.6, C03G5.10, C03G5.11, C03G5.12, C03G5.13, C03G5.2, C03G5.8, C03G5.9, C40H5.1, D1025.4, D1025.6, D1025.7, D1025.8, D1025.9, F09F7.8, F35C5.10, F42F12.1, F42F12.10, F42F12.6, F42F12.7, F42F12.8, F42F12.9, H12D21.1, H12D21.12, H12D21.13, H12D21.14, H12D21.15, W06A7.5, Y38E10A.11, Y38E10A.12, Y38E10A.13, Y38E10A.15, Y38E10A.16, Y38E10A.25, Y38E10A.26, Y38E10A.29, ZC412.6, ZC412.7 |
| DUF19 | 51 (42) | DUF19 domain containing proteins (TF318149 and others) | C01B7.7, C03G6.5, C04E12.2, C17B7.2, C17B7.3, C17B7.4, C17B7.9, C36C5.12, C36C5.14, C36C5.15, C36C5.4, C36C5.5, D1086.1, D1086.3, D1086.8, F07B7.13, F07B7.14, F07B7.8, F10G2.1, F35F10.13, F38A6.5, F59E11.6, K06C4.1, K09H9.5, T08G5.15, T08G5.3, T10D4.1, T13F3.4, T20D4.10, T20D4.11, T20D4.12, T20D4.15, T20D4.17, T20D4.19, T20D4.20, T28A11.16, T28A11.18, T28A11.19, T28A11.2, T28A11.3, T28A11.5, W09B7.3, W10G11.1, W10G11.2, W10G11.3, W10G11.4, Y23H5B.1, Y43F8B.10, Y43F8B.11, Y43F8B.20, ZK105.1 | C01B7.7, C03G6.5, C04E12.2, C17B7.2, C17B7.4, C17B7.9, C36C5.12, C36C5.14, C36C5.15, C36C5.5, D1086.1, D1086.3, D1086.8, F07B7.13, F10G2.1, F35F10.13, F38A6.5, F59E11.6, K09H9.5, T08G5.15, T08G5.3, T10D4.1, T20D4.10, T20D4.11, T20D4.12, T20D4.17, T20D4.19, T20D4.20, T28A11.16, T28A11.18, T28A11.19, T28A11.2, T28A11.3, T28A11.5, W10G11.1, W10G11.2, W10G11.3, W10G11.4, Y23H5B.1, Y43F8B.11, Y43F8B.20, ZK105.1 |
| nlp-genes | 44 (41) | nlp-genes | B0213.17, B0213.2, B0213.3, B0213.4, B0213.5, B0213.6, B0464.3, C01A2.7, C01C4.1, C04H5.8, C33A12.2, C54C8.9, CC4.2, D1009.4, D2005.2, E03D2.1, E03D2.2, F18E9.2, F30H5.2, F33A8.2, F35B12.7, F35C11.1, F36H12.1, F37A8.4, F45E4.8, F48B9.4, F48C11.3, F59C6.6, K09C8.6, M01D7.5, T13A10.5, T19C4.7, T23E7.4, T24D8.3, T24D8.4, T24D8.5, T28H10.4, Y43F8C.1, Y43F8C.2, Y45F10A.5, Y47D3B.2, Y74C9A.2, Y80D3A.10, ZK1320.10 | B0213.17, B0213.3, B0213.4, B0213.5, B0213.6, C01A2.7, C01C4.1, C04H5.8, C33A12.2, C54C8.9, CC4.2, D1009.4, D2005.2, E03D2.1, E03D2.2, F18E9.2, F30H5.2, F33A8.2, F35B12.7, F35C11.1, F36H12.1, F37A8.4, F45E4.8, F48B9.4, F48C11.3, K09C8.6, M01D7.5, T13A10.5, T19C4.7, T23E7.4, T24D8.3, T24D8.4, T24D8.5, T28H10.4, Y43F8C.1, Y43F8C.2, Y45F10A.5, Y47D3B.2, Y74C9A.2, Y80D3A.10, ZK1320.10 |
| DUF130 | 41 (25) | DUF130 domain containing proteins (TF322011) | C52E2.3, F07E5.7, F16G10.1, F16G10.10, F16G10.11, F16G10.13, F16G10.14, F16G10.2, F16G10.3, F16G10.4, F16G10.6, F16G10.7, F16G10.8, F16G10.9, F22E5.2, F43C11.1, F43C11.11, F43C11.12, F43C11.2, F43C11.4, F43C11.5, F45C12.9, F47B7.3, F47B7.4, F47B7.5, F59A6.11, K07G6.1, R03H10.4, R03H10.5, R05D8.11, R52.4, R52.5, R52.6, T07H3.7, T10D4.1, T10D4.11, T10D4.7, T14G12.6, Y25C1A.2, Y27F2A.9, ZK250.2 | C52E2.3, F16G10.10, F16G10.11, F16G10.13, F16G10.14, F16G10.2, F16G10.3, F16G10.4, F16G10.8, F16G10.9, F43C11.1, F43C11.2, F43C11.4, F45C12.9, F47B7.3, F47B7.4, F59A6.11, R03H10.4, R03H10.5, R05D8.11, R52.5, R52.6, T10D4.1, Y25C1A.2, ZK250.2 |
| DUF13 | 35 (15) | DUF13 domain containing proteins (TF316021) | BE0003N10.3, C03G6.6, C04F1.1, C13A2.10, C13A2.4, C13A2.9, C17A2.7, C28H8.8, C31B8.12, C33G8.13, C33H5.1, E03H4.2, F07G11.1, F07G11.2, F07G11.7, F13A7.12, F15H10.8, F20D6.2, F21G4.3, F28A10.1, F47B8.5, F49C12.2, F49C12.4, K01D12.1, K01D12.3, R09H10.1, R09H10.2, T15D6.11, T15D6.8, W09C3.3, Y71G12B.18, Y82E9BL.12, ZK1025.3, ZK1086.3, ZK1290.7 | BE0003N10.3, C03G6.6, C04F1.1, C13A2.4, C17A2.7, C28H8.8, C33G8.13, E03H4.2, F13A7.12, F21G4.3, R09H10.1, R09H10.2, T15D6.8, Y71G12B.18, ZK1290.7 |
| DUF148 | 32 (31) | DUF148 domain containing proteins (TF316074, others) | C06A8.3, C28C12.3, C28C12.4, C32H11.11, C32H11.5, C32H11.6, C32H11.8, C42D4.1, F11F1.2, F11F1.4, F11F1.8, F13E9.14, F13E9.4, F13E9.8, F58G4.3, M7.10, M7.9, R11G11.6, R11G11.7, T05E11.8, T17H7.1, T23G4.5, Y57G11C.39, Y67A10A.10, ZC204.1, ZK488.10, ZK488.7, ZK596.1, ZK822.2, ZK822.4, ZK822.6, ZK970.7 | C06A8.3, C28C12.3, C28C12.4, C32H11.11, C32H11.5, C32H11.6, C32H11.8, C42D4.1, F11F1.2, F11F1.4, F11F1.8, F13E9.14, F13E9.4, F13E9.8, F58G4.3, M7.10, M7.9, R11G11.6, R11G11.7, T05E11.8, T17H7.1, T23G4.5, Y57G11C.39, Y67A10A.10, ZC204.1, ZK488.10, ZK488.7, ZK596.1, ZK822.2, ZK822.4, ZK970.7 |
| flp-genes | 28/30 |  | C03G5.7, C05E11.8, C18D1.3, C24A1.1, C25H3.5, C26F1.10, C36H8.3, C52D10.11, E01H11.3, F07D3.2, F15D4.8, F22B7.2, F23B2.5, F31F6.4, F33D4.3, F39H2.1, F49E10.3, K02G10.4, K04H4.7, M79.4, R03A10.2, R173.4, T06C10.4, T07D10.6, W07E11.2, W07E11.3, W07E11.4, Y37D8A.15, Y48D7A.2, ZK525.1 | C05E11.8, C18D1.3, C24A1.1, C25H3.5, C26F1.10, C36H8.3, C52D10.11, E01H11.3, F07D3.2, F15D4.8, F23B2.5, F31F6.4, F33D4.3, F39H2.1, F49E10.3, K02G10.4, K04H4.7, M79.4, R03A10.2, R173.4, T06C10.4, T07D10.6, W07E11.2, W07E11.3, W07E11.4, Y37D8A.15, Y48D7A.2, ZK525.1 |
| CW | 28 (21) | CW containing proteins, excluding CL containing proteins (TF322162 and others) | C17D12.9, C18A3.11, C27C7.5, C31B8.2, C50E3.2, E03H12.4, E03H12.9, F10A3.11, F11D11.6, F29A7.3, F29A7.8, F38A1.15, F54D12.1, F54D12.10, F54D12.2, F54D12.7, F54D12.8, K04H8.2, M199.7, T07D3.2, Y110A2AL.5, Y116A8C.1, Y17D7B.5, Y39A3A.2, Y44A6B.3, Y73F8A.22, Y73F8A.23, Y77E11A.8 | C18A3.11, C50E3.2, E03H12.4, E03H12.9, F10A3.11, F11D11.6, F29A7.3, F29A7.8, F54D12.1, F54D12.10, F54D12.7, F54D12.8, M199.7, T07D3.2, Y110A2AL.5, Y116A8C.1, Y17D7B.5, Y39A3A.2, Y44A6B.3, Y73F8A.22, Y73F8A.23 |
| DUF274 | 22 (15) | DUF274 domain containing proteins (TF315857) | B0024.4, B0554.6, C18H7.11, C30E1.8, C34H4.1, C34H4.2, C34H4.3, F11D11.3, F11D11.4, F39E9.1, F54E2.1, K11H12.11, K11H12.3, K11H12.4, Y41D4B.15, Y41D4B.16, Y41D4B.17, Y46D2A.1, Y46D2A.2, Y47H9C.1, ZK6.10, ZK6.11 | B0554.6, C30E1.8, C34H4.1, C34H4.2, C34H4.3, F11D11.3, F54E2.1, K11H12.11, K11H12.4, Y41D4B.15, Y41D4B.16, Y46D2A.2, Y47H9C.1, ZK6.10, ZK6.11 |
| small DB proteins | 18 (17) | small DB containing proteins withl no other domain | C42D4.3, C49F8.3, C56C10.4, F13H8.5, F18C5.9, F26G1.5, F26G1.9, F29C12.1, F35B3.4, F53F8.4, M03A1.7, R57.2, T20G5.12, T20G5.13, T22E5.3, Y57G11C.42, Y65B4BL.1, ZK1010.4 | C42D4.3, C49F8.3, C56C10.4, F13H8.5, F18C5.9, F26G1.5, F26G1.9, F29C12.1, F35B3.4, F53F8.4, M03A1.7, R57.2, T20G5.12, T20G5.13, T22E5.3, Y57G11C.42, ZK1010.4 |
| DUF229 | 15 (11) | DUF229 domain containing proteins (TF312922 and others) | F07C3.3, F10C2.3, F15H10.7, F28A10.2, F32D8.2, F32G8.2, K03A11.4, M03F8.5, R03G8.1, R03G8.3, T07G12.3, T23E1.1, Y38A10A.2, Y41E3.1, Y73F8A.35 | F10C2.3, F28A10.2, F32D8.2, F32G8.2, M03F8.5, R03G8.1, R03G8.3, T07G12.3, T23E1.1, Y41E3.1, Y73F8A.35 |
| DUF263 | 13 (11) | DUF263 domain containing proteins (TF315866) | C07G3.8, C12D5.10, C12D5.9, C50H11.17, F08D12.7, F40G12.4, F40G12.5, F40G12.6, K03B4.6, R13D7.2, Y73C8C.4, ZK105.5, ZK105.6 | C07G3.8, C12D5.10, C12D5.9, C50H11.17, F08D12.7, F40G12.4, F40G12.5, F40G12.6, R13D7.2, Y73C8C.4, ZK105.6 |
| DUF1647 | 14 (11) | DUF1647 domain containing proteins (TF320732) | C06B8.2, C16D9.4, C16D9.5, F22E5.1, F28G4.3, F32B4.1, F41D3.9, F46F5.10, R05A10.7, T15D6.9, Y27F2A.6, Y45G12C.1, ZK1053.1, ZK1225.1 | C06B8.2, C16D9.5, F22E5.1, F28G4.3, F32B4.1, F41D3.9, F46F5.10, R05A10.7, T15D6.9, Y27F2A.6, ZK1225.1 |
| DUF236 | 13 (10) | DUF236 domain containing proteins (TF315782) | C03C11.1, C04F12.6, C36F7.5, C47E12.10, F27C1.3, F42H11.1, F47B3.4, F47B3.5, K07A1.5, R08A2.1, T05E11.7, Y8G1A.1, ZC376.8 | C03C11.1, C36F7.5, C47E12.10, F27C1.3, F42H11.1, F47B3.4, K07A1.5, R08A2.1, Y8G1A.1, ZC376.8 |
| TF352284 | 13 (13) | TF352284 | D2096.10, D2096.13, D2096.5, E02H9.1, E02H9.9, F17E9.2, F17E9.3, F17E9.4, K04F1.8, K04F1.9, T26A8.3, Y18H1A.8, Y37H2A.13 | D2096.10, D2096.13, D2096.5, E02H9.1, E02H9.9, F17E9.2, F17E9.3, F17E9.4, K04F1.8, K04F1.9, T26A8.3, Y18H1A.8, Y37H2A.13 |
| DUF316 | 12 (10) | DUF316 domain containing proteins (TF320025, TF317169, others) | C46E10.1, D1081.3, F15H9.1, F35E2.5, F35E2.9, F36D1.6, R01H2.2, T04A6.3, T06G6.8, Y116A8A.4, Y116A8A.6, Y47H9B.2 | C46E10.1, F15H9.1, F35E2.5, F35E2.9, F36D1.6, R01H2.2, T04A6.3, Y116A8A.4, Y116A8A.6, Y47H9B.2 |
| DUF273 | 12 (7) | DUF273 domain containing proteins (TF315659) | C54C8.4, F25H2.7, F31F4.1, F32H2.11, F32H2.8, F41D3.11, F46F5.14, F46F5.4, T08G2.2, T26E4.7, ZK1055.2, ZK1055.4 | C54C8.4, F25H2.7, F31F4.1, F32H2.11, F41D3.11, F46F5.14, T08G2.2 |
| DUF271 | 11 (7) | DUF271 domain containing proteins (TF315382) | C26C6.4, F28G4.2, F28G4.4, F41C3.11, F49C12.1, F49C12.3, F53F4.7, Y48E1B.8, Y51H7C.12, Y57G11C.31, ZK455.5 | C26C6.4, F28G4.2, F28G4.4, F41C3.11, F53F4.7, Y48E1B.8, ZK455.5 |
| DUF268 | 11 (9) | DUF268 domain containing proteins (TF318673) | C13A2.3, C13A2.7, F32B5.3, F32D8.8, F46F5.12, F56H6.2, K04A8.1, K04A8.2, K06H6.1, W08G11.1, ZK1055.5 | C13A2.3, C13A2.7, F32B5.3, F32D8.8, F56H6.2, K04A8.1, K06H6.1, W08G11.1, ZK1055.5 |
| DUF870 | 8/11 | DUF870 domain containing proteins (TF322745) | B0416.7, F36A4.1, F36A4.2, F36A4.3, F36A4.4, F36A4.5, F47C12.11, F47C12.6, F47C12.7, F47C12.8, ZC239.22 | B0416.7, F36A4.1, F36A4.2, F36A4.4, F47C12.11, F47C12.6, F47C12.8, ZC239.22 |
| DUF672 | 7/10 | DUF672 domain containing proteins (TF315369) | F28A10.5, F38C2.4, K06H6.2, R05A10.8, Y105C5B.23, Y39F10A.3, Y40H7A.2, Y40H7A.3, Y40H7A.4, Y55F3AM.11 | F28A10.5, K06H6.2, R05A10.8, Y39F10A.3, Y40H7A.3, Y40H7A.4, Y55F3AM.11 |
| DUF1261 | 10/10 | DUF1261 domain containing proteins (TF315792) | F19C7.1, F49C12.14, F56C9.7, LLC1.2, T20D3.2, Y119D3B.13, Y34B4A.5, Y34B4A.6, Y34B4A.9, Y53G8AM.5 | F19C7.1, F49C12.14, F56C9.7, LLC1.2, T20D3.2, Y119D3B.13, Y34B4A.5, Y34B4A.6, Y34B4A.9, Y53G8AM.5 |
| TF319413 | 8/10 | TF319413 | C01B10.7, F22D6.15, K02E11.3, K02E11.4, K02E11.5, K02E11.6, K02E11.7, K02E11.9, T19H12.3, Y73B6A.3 | F22D6.15, K02E11.3, K02E11.4, K02E11.5, K02E11.6, K02E11.7, T19H12.3, Y73B6A.3 |
