# Supplementary material for: A survey of putative secreted and transmembrane proteins encoded in the C. elegans genome
Source: BMC Genomics. 2012 Jul 23;13:333. doi: 10.1186/1471-2164-13-333 (PMC3534327; doi:10.1186/1471-2164-13-333)
Supplement: Additional file 7 — Lists of transmembrane proteins grouped by domain structure. [file 1471-2164-13-333-S7.doc]

**TM proteins listed according to functional groups**

*For each group or subgroup the number of belonging members is listed in brackets followed by the (sub) group name. Group members are identified by their sequence names. Gene names (if avaialable) can be easily retrieved from GExplore (<http://genome.sfu.ca/gexplore/>).

**Smaller subgroups are defined by domains (pFAM ID or SMART ID), gene names, GO annotations, publications referenced, or a combination of the above.

**(2680) proteins with additional domains**

**(343) CHANNELS**

B0207.12, B0280.12, B0334.2, B0395.1, B0399.1, B0491.4, B0491.8, B0495.4, B0564.3, B0564.4, C01B12.3, C01B12.5, C01G6.1, C02C2.3, C03F11.1, C04C3.2, C04F5.1, C05C12.3, C06A8.9, C06E1.4, C07A9.11, C07A9.4, C07A9.8, C07H4.2, C09B9.3, C09G5.1, C10G8.5, C11D2.6, C11E4.3, C13D9.7, C13D9.8, C15A7.1, C15A7.4, C16E9.4, C18B2.6, C18H7.2, C23H5.7, C24A3.6, C24G7.1, C24G7.2, C24G7.4, C24H11.8, C25B8.1, C27C12.5, C27F2.2, C27H5.8, C29E6.2, C29F4.2, C29F5.4, C30D11.1, C31H5.3, C32C4.1, C32C4.2, C33D12.3, C33G3.3, C35A5.1, C35C5.5, C36H8.2, C37A5.1, C39B10.2, C40C9.1, C40C9.2, C41C4.5, C43F9.9, C43G2.4, C43H6.9, C44E12.3, C45B2.4, C46A5.2, C47C12.6, C48A7.1, C48E7.9, C49A1.2, C49A1.3, C50B6.11, C52B9.6, C53A5.5, C53C9.3, C53D6.3, C54D2.5, C54F6.13, D2062.12, D2092.3, E02H4.1, E04F6.11, F02D10.5, F07A5.1, F07B10.5, F07F6.6, F08A10.1, F08B12.3, F08G12.10, F09C12.1, F09E8.7, F09G2.5, F09G8.9, F11A5.10, F11C7.1, F11H8.2, F12B6.3, F12F3.1, F13G3.8, F14B8.1, F14F11.1, F14H3.2, F14H8.6, F15E6.2, F16B3.1, F16F9.5, F17C8.5, F17E9.7, F17E9.8, F18G5.4, F19D8.1, F20A1.7, F21A3.7, F21C3.1, F21F3.5, F22A3.3, F22B7.7, F22E5.11, F22F4.2, F23B2.3, F25D1.4, F25F8.2, F25G6.3, F25G6.4, F26A3.6, F26D11.10, F27B3.2, F28A12.1, F28F8.1, F28H7.10, F29F11.4, F31D4.7, F32A5.5, F32B6.9, F32D8.15, F32G8.4, F32H5.7, F33D11.5, F34D6.3, F35C12.2, F36A2.4, F36F2.5, F38E11.12, F40F9.9, F41B4.4, F41E7.1, F41E7.2, F44A2.2, F46A9.3, F46F3.2, F47A4.1, F48E3.7, F52E4.4, F53C11.6, F53E10.2, F54D1.5, F55C5.3, F55D10.5, F55G1.12, F57C7.2, F57G8.5, F58E1.6, F58G6.4, F58G6.8, F58H7.3, F59B1.9, F59F3.4, H35N03.1, K01A11.4, K01A2.8, K01D12.4, K02B2.4, K02G10.7, K03B8.9, K03F8.2, K04A8.4, K04G11.5, K06B4.12, K06C4.6, K07A1.16, K09C8.1, K10D3.1, K10D6.1, K11C4.5, K11G12.2, K11G12.7, K11H3.7, M02A10.2, M02F4.8, M04B2.5, M110.2, M60.5, R01E6.4, R02E12.8, R03E1.3, R03E9.4, R04F11.4, R06A4.10, R06B10.4, R07A4.1, R07B7.1, R07D5.1, R09F10.4, R11G10.1, R12E2.4, R12E2.5, R12E2.9, R12G8.2, R12H7.1, R13.3, R13A1.4, R13A1.9, R13A5.4, R186.5, T01B4.1, T01B4.2, T01C3.10, T01C8.7, T01H10.1, T01H10.2, T01H10.3, T01H10.5, T01H10.6, T01H10.7, T01H8.5, T02C5.5, T05B4.1, T05C12.2, T06A4.2, T06F4.2, T06H11.1, T08G11.5, T09A12.3, T09A5.3, T10B10.7, T12C9.3, T15B7.16, T16H5.1, T18H9.5, T19C3.1, T19D7.1, T20B12.9, T20G5.4, T21C12.1, T21C9.3, T21F2.1, T23H4.1, T24D8.1, T26H10.1, T27A1.4, T27D12.2, T27E9.9, T28A8.1, T28B8.5, T28D9.7, T28F2.7, T28F4.2, W04D2.3, W06D12.2, W06D12.5, W10G11.16, Y105C5B.16, Y110A7A.3, Y113G7A.4, Y113G7A.5, Y18D10A.6, Y32F6B.2, Y37A1B.11, Y37E11AR.1, Y39A3B.2, Y39B6A.19, Y40C5A.2, Y44A6E.1, Y45G5AL.2, Y46G5A.26, Y47D3B.5, Y47G6A.1, Y47G6A.2, Y48A6B.6, Y48B6A.4, Y51A2D.19, Y54G9A.3, Y55F3BR.4, Y55F3C.3, Y57A10A.10, Y57A10A.28, Y57A10A.35, Y57G11C.2, Y57G11C.44, Y57G11C.49, Y58G8A.1, Y69E1A.7, Y69H2.11, Y69H2.12, Y69H2.2, Y71A12B.4, Y71D11A.5, Y71H2AM.22, Y71H9A.1, Y73B6BL.19, Y73B6BL.26, Y73B6BL.42, Y73F8A.1, Y73F8A.11, Y73F8A.2, Y73F8A.30, Y74E4A.1, Y76B12C.1, Y8G1A.2, Y97E10B.7, ZC168.1, ZC196.7, ZC21.2, ZC239.16, ZC239.17, ZC239.5, ZC317.3, ZC410.4, ZC482.1, ZC482.5, ZC504.2, ZC518.1, ZC84.2, ZK1067.5, ZK1251.8, ZK1321.2, ZK1321.3, ZK512.3, ZK520.2, ZK525.2, ZK675.3, ZK688.2, ZK770.1, ZK770.3, ZK792.2, ZK792.3, ZK822.3, ZK849.4, ZK849.5, ZK973.5

**(484) TRANSPORTERS**

AH6.2, B0212.4, B0222.2, B0222.3, B0240.1, B0240.4, B0252.3, B0285.6, B0303.11, B0331.2, B0361.11, B0365.3, B0416.5, B0454.6, C01B4.7, C01B4.8, C01B4.9, C01G12.8, C02C2.4, C02E7.1, C03A3.2, C03B1.13, C03H5.2, C05A9.1, C05D10.3, C05E11.4, C05E11.5, C05G5.1, C06E1.3, C06G8.2, C06G8.3, C06H2.4, C06H5.6, C08D8.1, C09D4.1, C09H5.2, C10C6.5, C10C6.6, C10E2.6, C12D12.2, C13C4.5, C13C4.6, C14A6.2, C14C10.1, C14F5.4, C14H10.1, C15B12.7, C15H9.1, C16C10.1, C16C10.12, C17B7.5, C17E4.9, C18A3.2, C18C4.2, C18D1.2, C18E9.2, C18H9.5, C24F3.1, C24F3.5, C25E10.5, C26C6.9, C26H9A.1, C27C12.4, C29H12.2, C30H6.2, C30H6.6, C34G6.4, C35A11.4, C35A5.3, C38C10.2, C39E9.10, C39E9.7, C41C4.10, C42C1.16, C42C1.8, C43F9.6, C43G2.2, C44B7.6, C44B7.8, C44B7.9, C44C10.3, C46C11.2, C46C2.2, C47A10.1, C47A4.2, C47D12.3, C47E12.2, C47G2.3, C48A7.2, C48B4.4, C48D1.3, C49C3.1, C49F8.2, C50D2.2, C50E3.16, C50F4.14, C51E3.6, C52E12.3, C53B4.1, C53B4.3, C53B4.6, C54D1.1, C54G10.3, C55B7.6, C55C2.5, C56E6.1, C56E6.5, D1046.3, D1046.4, DH11.3, E03G2.2, E04A4.5, F01G12.1, F01G12.2, F01G4.6, F02C9.3, F02E11.1, F07C3.7, F08F1.5, F08F3.3, F09A5.1, F09G2.3, F10C2.7, F10D7.2, F10E7.9, F10G7.5, F11A5.9, F11D5.7, F12B6.1, F12B6.2, F13B12.2, F13G3.7, F13H10.3, F14B8.3, F14D12.5, F14D7.6, F14E5.1, F14F4.3, F15B10.1, F16H11.1, F16H11.3, F17C11.12, F19B6.4, F20B6.3, F21D12.3, F21F8.11, F21G4.1, F21G4.2, F22E10.1, F22E10.2, F22E10.3, F23F1.6, F23F12.13, F23F12.3, F23H12.2, F25B4.7, F25D1.2, F25G6.7, F27C1.2, F27C8.1, F27D9.2, F27E11.1, F27E11.2, F28F9.4, F30B5.7, F31C3.4, F31E8.4, F31F6.6, F32D8.6, F32H5.4, F35H10.4, F36G9.3, F36H1.10, F36H1.5, F36H1.9, F36H2.1, F36H2.2, F37B4.7, F37H8.4, F40F9.5, F41C3.2, F41C6.7, F41D9.5, F42E11.1, F43E2.4, F43G9.3, F44C8.7, F44D12.9, F44E7.7, F45E10.2, F45E4.11, F45E6.1, F47B8.10, F47E1.2, F47E1.4, F47G9.1, F48E3.2, F49C12.13, F49C12.6, F49E11.3, F49E8.5, F52B5.1, F52D10.1, F52F12.1, F52H2.2, F52H2.4, F53B1.8, F53H8.3, F54D12.3, F54E7.1, F55A4.8, F55F3.3, F55F8.9, F55H12.1, F56A4.10, F56A4.11, F56A4.12, F56C9.3, F56F4.3, F56F4.5, F57A10.3, F57A8.7, F57B10.5, F57C12.4, F57C12.5, F57F10.1, F58G11.4, F58G6.3, F58G6.9, F59A1.13, F59A3.4, F59B2.2, F59F5.1, H11E01.2, H13N06.5, H16O14.1, H17B01.1, H32K16.1, K01H12.2, K02A2.3, K02E11.1, K02G10.5, K04E7.2, K05B2.5, K05F1.6, K06H6.3, K07E3.7, K07G5.5, K07H8.2, K08C7.1, K08E4.6, K08E5.2, K08E7.9, K08F4.4, K08F9.1, K08H10.6, K09A9.3, K09C4.1, K09C4.4, K09C4.5, K09E9.1, K10G9.1, K10H10.1, K11D9.2, K11D9.3, K11G12.3, K11G12.4, K11G9.5, K12C11.3, K12C11.6, K12C11.7, K12G11.1, K12G11.2, M01F1.5, M01G5.5, M02B1.1, M03B6.2, M03F8.2, M117.1, M153.2, M162.5, M195.3, PDB1.1, R02E12.6, R02F11.3, R02F2.8, R03E9.3, R05C11.3, R05G6.6, R07B7.10, R07E3.4, R08F11.6, R09B5.11, R107.1, R10D12.1, R10E11.2, R10E11.8, R11E3.2, R13A1.2, R13A5.9, T01B11.4, T01B11.7, T01D3.5, T01H3.1, T01H3.3, T02B11.6, T02D1.5, T02G5.12, T03F7.1, T04B8.5, T04F8.1, T04G9.5, T04H1.1, T05A1.5, T07A5.3, T07G12.2, T07G12.5, T08B1.1, T09B9.2, T10C6.6, T10H9.5, T11F9.2, T11F9.4, T11G6.2, T11G6.3, T11G6.4, T12B3.2, T13A10.10, T13B5.1, T18D3.3, T19D12.10, T19D12.9, T20G5.6, T21B6.5, T21E8.1, T21E8.2, T21E8.3, T22E5.2, T22F3.10, T22F3.11, T22F3.7, T22F3.8, T22F7.1, T23G5.5, T23H2.1, T24H7.5, T25B6.7, T25D3.4, T26A5.1, T27A1.5, T27D12.1, T27E9.1, T28C12.1, T28C12.2, T28F3.3, T28F3.4, T28H11.8, VW02B12L.1, W01B11.2, W01B6.3, W01C8.6, W02D3.6, W03C9.6, W03G1.1, W03G9.1, W04C9.1, W04C9.6, W04G3.6, W05E10.1, W05H5.3, W08D2.5, W09C2.3, W09D10.2, W09D6.6, Y105E8A.12, Y105E8A.3, Y111B2A.19, Y111B2A.20, Y15E3A.4, Y18D10A.23, Y19D10A.10, Y19D10A.11, Y19D10A.12, Y19D10A.4, Y19D10A.5, Y19D10A.8, Y32F6A.2, Y32F6A.4, Y32F6B.1, Y37A1A.3, Y37A1C.1, Y37E3.16, Y38F2AL.4, Y38F2AR.2, Y38H6C.17, Y39B6A.41, Y39D8C.1, Y39E4A.2, Y39E4B.5, Y42G9A.6, Y43F4B.7, Y43F8A.5, Y43F8C.12, Y46G5A.30, Y47D3A.11, Y47G6A.7, Y48G8AL.11, Y49E10.11, Y4C6B.2, Y4C6B.3, Y4C6B.4, Y4C6B.5, Y50E8A.16, Y51A2D.18, Y51A2D.4, Y51A2D.5, Y51B9A.6, Y51F10.4, Y53C10A.9, Y53C12A.2, Y53F4B.12, Y53G8AR.7, Y53G8B.4, Y53H1C.1, Y54E10BR.7, Y54G2A.18, Y54G2A.4, Y54G9A.4, Y55F3BL.2, Y55H10A.1, Y57G11C.15, Y57G11C.23, Y58A7A.1, Y59A8B.21, Y59E9AL.4, Y59H11AR.2, Y59H11AR.4, Y61A9LA.1, Y66D12A.13, Y67D8C.10, Y69A2AR.4, Y6E2A.9, Y70G10A.3, Y71F9AM.6, Y71G10AR.1, Y71G10AR.4, Y71G12B.25, Y71H2AM.9, Y73B6BL.31, Y73B6BL.36, Y73E7A.3, Y74C10AR.3, Y75B8A.26, Y76A2A.2, Y82E9BR.16, ZC250.3, ZC395.3, ZC416.8, ZK1010.9, ZK1053.6, ZK180.3, ZK185.2, ZK185.5, ZK256.1, ZK287.2, ZK370.7, ZK455.7, ZK455.8, ZK484.2, ZK512.6, ZK54.1, ZK550.2, ZK563.1, ZK563.2, ZK6.6, ZK637.1, ZK637.8, ZK682.2, ZK809.4, ZK822.5, ZK829.10, ZK829.9, ZK892.3, ZK896.9

**(517) ENZYMES**

AC3.2, AC3.5, AC3.7, AC3.8, B0024.13, B0035.18, B0218.1, B0218.2, B0250.9, B0304.3, B0310.5, B0334.5, B0361.7, B0361.8, B0395.2, B0399.2, B0491.1, B0495.7, B0511.2, BE10.2, C01B10.10, C01B10.4, C01B10.7, C01B10.9, C01C10.3, C02A12.8, C02C2.5, C02H6.1, C03A7.11, C03C10.3, C04A11.4, C04F12.10, C04F5.7, C04F6.5, C05D12.1, C06B3.2, C07G3.9, C08B11.4, C08B11.8, C08B6.1, C08F11.8, C08F8.4, C08H9.10, C08H9.11, C08H9.12, C08H9.13, C08H9.3, C08H9.4, C08H9.7, C09F9.2, C10F3.2, C10H11.3, C10H11.4, C10H11.5, C10H11.6, C12D8.5, C13B4.1, C13D9.9, C14A4.3, C15F1.6, C15H11.4, C17A2.5, C17G1.3, C17H12.4, C18C4.3, C18G1.8, C18H2.4, C23G10.6, C23H4.2, C23H4.3, C23H4.4, C23H4.7, C25A1.5, C25H3.9, C27A12.9, C27A7.1, C27D8.4, C31A11.1, C31A11.5, C31A11.7, C32C4.7, C33A12.6, C33H5.11, C33H5.14, C33H5.18, C34B2.10, C34H3.1, C35A5.2, C35C5.11, C35C5.2, C36B1.12, C38C6.2, C40C9.5, C40H1.4, C42C1.7, C42D4.2, C43F9.10, C44B7.11, C44H9.1, C46F4.2, C48B4.2, C48E7.8, C49A9.8, C49D10.4, C49D10.7, C49D10.8, C49F5.5, C50D2.9, C52B9.9, C53D5.5, C54G4.8, C54G7.2, C56G2.6, D1009.1, D1022.1, D2024.3, D2063.2, D2085.6, DY3.7, E01G6.3, E03H4.7, E04F6.4, EGAP2.3, EGAP9.2, F01D4.1, F01D4.2, F01E11.1, F01G4.5, F08G5.2, F08G5.5, F09B12.3, F09B9.1, F09G2.6, F09G2.8, F10D2.11, F10D2.12, F10D2.2, F10D2.5, F10D2.6, F10D2.7, F10D2.9, F11A5.5, F11A5.8, F11E6.5, F12A10.4, F13D11.1, F13E6.5, F13H10.4, F13H10.5, F14B6.5, F14E5.3, F14F3.3, F14H3.10, F15A2.2, F15A4.8, F17B5.2, F17C11.7, F18A12.5, F18A12.8, F19H6.4, F20B4.6, F21F3.3, F21F8.2, F22E10.5, F22F7.1, F23H11.9, F26D10.9, F26E4.6, F26F4.3, F26G1.6, F27D9.6, F27D9.7, F28A10.7, F28B3.9, F28C6.4, F28D1.11, F28D1.9, F28G4.1, F28G4.5, F29F11.2, F30A10.4, F31F4.7, F33A8.5, F33D11.9, F33D4.4, F35C11.5, F35C8.5, F35E2.6, F35H8.6, F36A2.9, F36G9.12, F36H1.2, F37A4.1, F37C4.1, F37C4.2, F37C4.3, F39G3.1, F39G3.4, F39G3.5, F39G3.6, F41D3.10, F41D3.2, F41D3.4, F41D3.5, F41E6.14, F41H10.7, F41H10.8, F42F12.3, F42G8.6, F42G9.6, F44B9.5, F44F4.6, F45E4.7, F45H10.1, F46E10.9, F47B7.2, F47C10.6, F47F6.3, F48A11.1, F48C1.1, F48F5.5, F49B2.6, F49E12.10, F49E12.9, F49H12.6, F52F10.3, F52F10.4, F52H2.6, F53C11.2, F53C3.13, F53F4.5, F53G12.3, F53H8.4, F54C1.1, F54D8.2, F55A11.5, F55A12.4, F55D10.3, F55E10.6, F55H2.5, F56A11.5, F56B3.7, F56C11.1, F56D1.5, F56G4.1, F56H11.3, F56H11.4, F56H6.11, F56H6.12, F57B9.4, F59A1.10, F59A3.1, F59F4.4, F59G1.1, H06H21.10, H12I19.4, H12I19.5, H13N06.6, H14N18.4, H19N07.4, H21P03.3, H23N18.1, H23N18.2, H23N18.3, H41C03.3, K02E7.10, K02F3.6, K03A1.2, K03B8.2, K03B8.5, K04A8.10, K04D7.2, K04F1.1, K04F10.4, K07B1.4, K07B1.5, K08B4.3, K08B4.4, K08C7.2, K08C7.5, K08F9.3, K09A9.6, K09E10.1, K09E10.2, K09E4.2, K10C2.3, M01A10.3, M01B2.6, M01E11.1, M03A1.3, M03A1.8, M03C11.5, M79.2, M88.1, MTCE.11, MTCE.12, MTCE.21, MTCE.23, MTCE.25, MTCE.26, MTCE.31, MTCE.34, MTCE.35, MTCE.4, R01B10.4, R02C2.3, R03E9.2, R03H4.1, R03H4.5, R03H4.6, R04B5.9, R04D3.1, R07B7.4, R07B7.5, R07B7.6, R07B7.8, R07B7.9, R07E3.1, R07E3.5, R08F11.1, R09B5.6, R09D1.1, R09D1.10, R09D1.11, R09D1.2, R09D1.3, R09D1.5, R09D1.6, R09F10.1, R13D11.4, R155.1, R173.1, R173.3, R57.1, T01G5.2, T02B5.1, T02B5.3, T03D3.1, T03D8.6, T03G11.4, T04H1.7, T04H1.8, T05E11.5, T05G5.5, T05H4.1, T05H4.13, T05H4.4, T05H4.5, T06C12.10, T06C12.8, T06D4.4, T06D8.3, T06E8.1, T07C4.7, T07C5.1, T07D1.3, T07F10.1, T07H6.1, T07H6.2, T08D10.2, T08H10.4, T09A5.11, T09B4.1, T09E11.4, T09E11.5, T09E11.6, T09E11.7, T09H2.1, T10B10.8, T10B9.1, T10B9.10, T10B9.3, T10B9.5, T11F9.11, T12A2.2, T12B3.3, T12G3.4, T13C5.1, T13C5.6, T13F2.1, T14D7.2, T15D6.2, T15D6.5, T18H9.2, T19B10.8, T19D7.7, T19H12.1, T19H12.10, T19H12.11, T19H12.6, T19H12.9, T19H5.1, T19H5.2, T19H5.3, T20D3.8, T22B11.2, T22D1.11, T22D1.4, T22G5.5, T23F1.7, T23G4.4, T24D1.4, T25B9.7, T25G3.2, T26A5.4, T26A8.2, T26E4.4, T26H2.7, T27E4.6, T27E9.5, T27F7.3, T28D9.3, T28F3.5, T28F3.9, VF36H2L.1, VZK822L.1, W01A11.2, W01A8.4, W02A2.1, W02F12.2, W03B1.7, W03B1.8, W03F9.4, W06D12.3, W06G6.1, W07A12.6, W07A12.7, W08D2.4, W10C8.5, Y110A2AL.12, Y116A8C.14, Y116A8C.16, Y116A8C.463, Y17D7A.4, Y22D7AL.8, Y37A1B.13, Y37D8A.10, Y37D8A.13, Y37E11AR.5, Y38C1AA.1, Y38F2AR.7, Y38H6C.16, Y39E4B.9, Y39G10AR.6, Y39H10A.2, Y40D12A.2, Y41E3.3, Y42A5A.1, Y43F8A.3, Y45F10D.3, Y46G5A.17, Y46G5A.2, Y46G5A.5, Y46H3A.6, Y47D3A.30, Y48G9A.10, Y49A3A.1, Y49C4A.8, Y51H4A.25, Y53F4B.2, Y53G8B.2, Y54E10A.14, Y54E10BR.1, Y54E10BR.5, Y54E5A.1, Y56A3A.2, Y56A3A.32, Y56A3A.36, Y57G11C.17, Y5H2B.1, Y60A3A.14, Y66H1B.4, Y67A10A.1, Y67D8C.9, Y67H2A.8, Y6B3B.11, Y6B3B.5, Y71H2AM.25, Y71H2AM.4, Y71H2AR.2, Y73B6BL.7, Y7A5A.1, Y7A9A.1, Y80D3A.5, Y82E9BR.3, Y87G2A.14, Y94H6A.5, Y97E10AR.2, ZC101.3, ZC155.4, ZC376.1, ZC376.2, ZC376.3, ZC443.5, ZC443.6, ZC455.3, ZC455.4, ZC455.5, ZC455.6, ZC506.3, ZC513.5, ZC8.1, ZK1248.14, ZK1251.3, ZK154.7, ZK370.4, ZK40.1, ZK550.1, ZK563.6, ZK678.8, ZK792.1, ZK816.5, ZK858.1, ZK938.6, ZK938.7, ZK945.1, ZK970.1

**(354) SIGNALING**

AC7.1, AH6.1, AH9.1, B0024.6, B0198.3, B0212.5, B0213.2, B0240.3, B0244.10, B0244.5, B0244.7, B0252.1, B0273.4, B0286.2, B0334.11, B0334.6, B0410.2, B0457.1, B0464.3, B0511.12, B0511.13, B0563.6, C01F1.2, C01F1.4, C01G6.8, C02B8.5, C02D4.2, C02H7.2, C04H5.3, C05D2.1, C06A12.4, C06B8.7, C06G4.5, C07F11.1, C08H9.5, C09B7.1, C10C6.2, C10F3.3, C13B9.4, C13G3.2, C14F11.3, C15A7.2, C15B12.5, C15F1.3, C15H11.2, C16B8.1, C16D6.2, C16D9.2, C17D12.6, C17F4.6, C17H11.1, C18A11.5, C18B12.2, C18E3.8, C24A8.1, C24B5.1, C24B5.3, C24G6.2, C25B8.5, C25B8.7, C25F6.4, C25G6.5, C26F1.6, C26G2.1, C30A5.10, C30B5.5, C30F12.6, C31E10.8, C32D5.2, C32E8.8, C38C10.1, C39E6.6, C41D11.9, C41D7.2, C41G11.4, C43C3.2, C43G2.1, C44F1.5, C45B2.7, C48C5.1, C49A9.7, C49C3.21, C49H3.1, C50F7.1, C50H2.1, C50H2.2, C52B11.3, C53C11.3, C53C7.1, C54A12.1, C54A12.2, C56A3.3, C56G3.1, D1009.3, D1014.2, D1022.6, D1037.2, D1073.1, E01H11.1, E02D9.1, E04D5.2, F01E11.5, F02E8.2, F02E8.6, F02E9.7, F07C3.1, F08B1.2, F08F1.1, F09A5.2, F09B12.6, F09G2.1, F09G8.4, F11D5.3, F11E6.8, F13D2.2, F13D2.3, F14D12.6, F14F4.1, F15A8.5, F15B9.7, F16C3.1, F16D3.7, F17C8.1, F18F11.5, F18H3.5, F21C10.12, F21H12.4, F21H7.9, F22E5.3, F23H11.5, F23H12.6, F27E11.3, F29C4.1, F31A9.3, F31B9.1, F31D5.4, F31D5.5, F31F6.5, F32A7.3, F33D11.11, F33D4.2, F35G8.1, F35H10.10, F35H12.3, F36D4.4, F36H1.4, F39B2.8, F39B3.2, F40A3.5, F40B5.2, F41E7.3, F42C5.2, F43C9.4, F43D9.1, F44D12.2, F44F4.4, F44G4.8, F45D3.5, F45H11.4, F46C3.1, F46G10.5, F47D12.1, F52D10.4, F52E1.4, F53A9.5, F53B7.2, F54D7.3, F54F12.1, F54F7.5, F54G8.5, F55A11.3, F55E10.7, F55F8.1, F56B6.5, F56C11.2, F56D1.2, F56E10.3, F57A8.4, F57C9.6, F57H12.4, F58A3.2, F59B2.13, F59C12.2, F59D12.1, F59F3.1, F59F3.5, F59F5.3, H02I12.3, H06I04.5, H09F14.1, H10E21.2, H22D07.1, H23L24.4, K02E10.8, K02F2.6, K03H6.1, K03H6.5, K04B12.1, K04D7.4, K06C4.8, K06C4.9, K07A3.2, K07C10.1, K07F5.6, K08D9.3, K08F11.5, K09E4.5, K09F6.3, K09G1.4, K10B4.4, K10C8.2, K10F12.3, M03A1.1, M03F4.3, M05B5.1, M176.6, M176.7, R01E6.1, R02D5.6, R03A10.6, R03D7.8, R03E1.2, R06B10.1, R06B9.6, R09D1.12, R09H10.4, R106.2, R107.8, R11F4.2, R11G1.6, R12C12.3, R134.1, R134.2, R13H7.2, R155.2, R155.3, T01A4.1, T01C2.1, T01C4.2, T02C5.1, T02D1.6, T02E9.1, T02E9.3, T03D8.5, T04F8.2, T05A1.1, T05F1.1, T07C4.8, T07D10.2, T07D4.1, T07F8.2, T07H8.6, T09E8.3, T10H9.2, T14B1.2, T14E8.1, T14E8.3, T17A3.1, T19B4.7, T19F4.1, T21B4.4, T21H3.2, T22C1.7, T22D1.12, T23B3.4, T23C6.5, T23D8.1, T23G7.2, T26C12.4, T27B2.1, W02D7.7, W03F11.2, W03F11.4, W05B5.2, W05H12.1, W07G1.5, W10C4.1, Y105C5A.23, Y105C5B.2, Y110A2AL.8, Y113G7C.1, Y116A8B.5, Y18D10A.7, Y22D7AR.13, Y22D7AR.7, Y23H5B.4, Y34D9A.2, Y34D9B.1, Y37E11AL.5, Y38F1A.3, Y38H6C.20, Y39A1B.2, Y39A3B.5, Y39B6A.18, Y39B6A.30, Y40H4A.1, Y41D4A.8, Y41D4B.24, Y41G9A.4, Y47H9C.4, Y48B6A.10, Y48E1B.14, Y48G1C.5, Y4C6A.2, Y53C12A.1, Y53F4B.28, Y54E2A.1, Y54E2A.12, Y54E5B.1, Y54G2A.25, Y54G2A.35, Y55D5A.5, Y55F3AL.1, Y57A10C.10, Y58G8A.4, Y59H11AL.1, Y65B4BR.3, Y69A2AR.19, Y69E1A.3, Y70D2A.1, Y71F9B.5, Y73C8B.4, Y80D3A.7, Y80D3A.8, ZC190.2, ZC239.7, ZC374.1, ZC412.1, ZC412.2, ZC434.6, ZC504.5, ZC506.4, ZC84.4, ZK1067.1, ZK1086.1, ZK1307.7, ZK180.1, ZK270.1, ZK377.2, ZK418.6, ZK418.7, ZK455.2, ZK455.3, ZK524.1, ZK622.2, ZK643.3, ZK675.1, ZK792.7, ZK813.5, ZK896.8, ZK938.5, ZK945.9, ZK970.5, ZK970.6

**(65) TRAFFICKING**

B0244.2, B0272.2, B0513.9, C01A2.3, C05E11.1, C15C7.1, C18E9.10, C30A5.5, C30B5.2, C31E10.7, C44H4.2, C54H2.5, C56A3.7, D2013.10, D2092.1, F08F8.8, F10C5.2, F23H12.1, F25D7.1, F29D11.1, F31E8.2, F35C8.4, F36F2.4, F40G9.1, F41C3.4, F41H10.11, F42G8.11, F48F7.2, F55A11.2, F55A4.1, F56A8.7, F56H1.1, F57B10.10, F58G11.1, M01D7.2, M03E7.5, R12E2.2, R13A5.1, T01B11.3, T07A5.2, T07F10.4, T08A9.3, T08D2.2, T10B10.5, T10H9.4, T11F8.3, T12A2.15, T13F2.8, T14D7.3, T23H2.2, VF39H2L.1, W02B8.6, Y110A7A.11, Y11D7A.9, Y25C1A.7, Y47D7A.16, Y56A3A.22, Y57G11C.4, Y59E9AL.7, Y69A2AR.6, Y71A12C.2, Y71F9B.3, ZC155.7, ZK795.4, ZK858.7

**(58) CELL ADHESTION**

B0034.3, C01C10.1, C01C10.4, C03F11.3, C09F12.1, C18D1.4, C18F3.2, C24H10.1, C29A12.4, C45G7.5, C54G4.4, F02G3.1, F07A5.3, F08B4.2, F10A3.1, F11C1.3, F11C7.4, F12D9.1, F18F11.3, F20B10.1, F25F2.2, F39H12.4, F44G3.10, F53B3.5, F54D1.6, F54F2.1, F54G8.3, F57C7.3, F59C12.1, F59C6.11, H19M22.2, H30A04.1, K03H1.5, K07D8.1, K08E5.3, K10D6.2, R04F11.1, R05H10.6, R07B1.3, R09E10.5, R10F2.1, SSSD1.1, T05A10.2, T05E11.2, T21B6.1, T22C8.8, T28B4.4, W02B9.1, W03D8.6, Y38F2AL.1, Y42H9B.2, Y49E10.20, Y67A10A.9, Y71D11A.1, Y76A2B.6, ZK1058.2, ZK112.7, ZK563.4

**(54) ECM COMPONENTS**

B0024.14, B0348.1, C29E6.4, C34F6.2, C34F6.3, C34G6.6, C43C3.3, C47G2.1, E04D5.3, F10E7.10, F11C3.2, F13B9.6, F20D1.8, F22B5.3, F33A8.9, F35C8.7, F38A3.1, F41A4.1, F47G9.3, F52B11.3, F53B6.6, F53B7.4, F53F1.1, F54B11.1, F55A4.10, F59F3.2, K06A1.3, M01E10.2, M142.2, M28.1, M88.6, R07E3.3, T04F8.4, T13B5.4, T21B10.6, T21H3.4, T22C8.7, T23F1.5, T23G11.6, T24C2.1, W01A8.3, W04C9.3, W06D12.1, W07A12.5, Y11D7A.11, Y18H1A.12, Y38C1AA.5, Y53H1B.1, Y55D5A.6, Y55F3C.7, Y57G11C.31, Y69H2.14, ZC328.1, ZK783.1

**(805) others (small groups, with GO association, with description)**

AC3.10, B0024.3, B0035.2, B0198.1, B0212.1, B0222.1, B0222.10, B0240.2, B0244.8, B0244.9, B0284.3, B0303.4, B0336.11, B0348.2, B0393.5, B0416.1, B0464.4, B0464.6, B0546.5, B0554.5, B0554.7, B0563.2, B0563.4, C01G10.1, C01G10.12, C01G5.4, C01G8.2, C01G8.4, C01H6.6, C02C6.2, C02D5.2, C02E11.1, C02F12.1, C02F5.11, C02F5.13, C02F5.8, C03B1.12, C03B8.1, C03G6.13, C03H5.6, C04A2.7, C04E12.4, C04E12.5, C05D11.7, C05D9.2, C06C6.7, C06G3.4, C06G8.1, C07A9.12, C08E8.1, C08G5.1, C08G9.2, C09B9.8, C09E8.3, C09F5.2, C09G4.1, C12D8.14, C12D8.16, C12D8.17, C12D8.18, C12D8.19, C12D8.6, C13A2.1, C13A2.5, C13A2.9, C13F10.5, C14B9.3, C14C11.4, C14F5.1, C15A11.7, C15H9.4, C15H9.5, C16B8.4, C16C10.5, C16C10.7, C16C4.13, C16C4.6, C16C8.17, C16D9.4, C17B7.3, C17D12.1, C17D12.3, C17E4.3, C17G1.8, C17H11.6, C17H12.2, C18B12.4, C18B12.6, C18D11.2, C18E9.3, C18H2.1, C18H2.3, C18H2.5, C18H7.11, C23H4.1, C23H4.8, C24A11.8, C24A8.3, C25B8.4, C25F6.7, C25G4.10, C25G6.2, C26B2.8, C26D10.5, C26D10.7, C28H8.4, C30A5.7, C30B5.9, C30G12.4, C30H6.5, C31E10.6, C32A3.3, C33A11.2, C33D12.2, C33H5.1, C33H5.19, C34B2.8, C34B4.5, C34D10.1, C34D4.4, C34F6.10, C34F6.7, C35A5.5, C35D10.1, C36B7.6, C36E8.3, C36H8.1, C37A5.2, C37A5.4, C37C3.12, C37C3.7, C37E2.2, C37E2.3, C38D9.5, C41C4.7, C41G7.9, C42D8.8, C43H6.6, C43H6.7, C44B7.3, C44H4.1, C46F11.1, C47B2.1, C47G2.4, C48E7.5, C49G7.1, C50H2.12, C52A11.4, C52B9.4, C52D10.12, C52E2.7, C53A5.13, C53D5.2, C54D10.4, C54F6.4, C56A3.4, C56C10.13, C56G2.1, C56G7.2, cTel55X.1, D1007.5, D1044.2, D1044.3, D1046.5, D1065.1, D2013.8, D2021.2, D2089.2, D2092.5, D2092.7, D2096.2, E03H12.3, E_BE45912.2, F01D5.10, F01E11.4, F02C12.1, F02C9.4, F02E8.5, F07B10.1, F07B7.14, F07C3.10, F07E5.8, F07F6.7, F07F6.8, F07G11.1, F07G11.3, F07G6.1, F07H5.2, F08B12.1, F08B12.2, F08C6.4, F08F1.7, F08G12.5, F09B12.2, F09B9.3, F09E5.11, F09E8.2, F09F9.4, F10E7.2, F10F2.4, F11A10.5, F11E6.6, F11G11.9, F13A2.6, F13B9.8, F13E6.3, F13G3.9, F13H8.4, F14B8.7, F14D12.4, F14D2.6, F14E5.2, F15B9.2, F15D4.3, F15H10.4, F15H10.7, F15H10.8, F16A11.3, F16B12.1, F16D3.6, F16F9.1, F19H8.5, F20C5.2, F20C5.4, F20D1.1, F20D1.10, F20D1.7, F21A10.2, F21C10.1, F21C10.3, F21C10.4, F21F3.7, F22B5.10, F22B7.10, F22F7.3, F22F7.4, F23D12.6, F23H12.8, F25D7.2, F25D7.5, F25E5.2, F25H5.8, F26A1.8, F26D10.11, F26D2.10, F26E4.11, F26F2.7, F26F4.4, F26F4.9, F27D4.7, F28B3.10, F28B3.6, F28D1.8, F28E10.2, F28H1.4, F28H7.6, F30A10.6, F30F8.9, F31B12.3, F31D5.1, F31D5.2, F31D5.3, F31F4.15, F31F7.2, F32A11.7, F32A6.5, F32D1.3, F32D8.14, F32D8.5, F33C8.1, F33C8.3, F33D11.12, F33D4.7, F34D10.2, F34D6.4, F35A5.5, F35D11.2, F35D11.3, F35D2.3, F35D2.4, F35E12.4, F35E2.1, F35F10.10, F36D1.8, F36D3.5, F36G3.3, F36G9.13, F36H5.5, F36H9.4, F37A4.4, F37C12.2, F37E3.2, F38A1.11, F38A5.10, F38A5.11, F38A5.12, F38A5.14, F38A5.5, F38A5.9, F38B2.3, F38B6.6, F38B7.10, F38B7.11, F38C2.4, F38E1.9, F39C12.3, F39E9.12, F40A3.2, F40E10.6, F40E12.2, F40F9.1, F40F9.2, F40G9.18, F40G9.9, F41G3.4, F41G4.3, F41H10.5, F42A8.3, F42E11.2, F42G8.10, F42G8.9, F43G9.13, F43G9.2, F43G9.6, F44E7.9, F44F4.1, F45C12.5, F45C12.6, F45F2.1, F45F2.5, F45F2.6, F45F2.7, F46C5.8, F46G11.1, F47B3.3, F47B8.5, F47G3.4, F48C1.2, F48C11.2, F48C5.1, F48F7.8, F48G7.3, F49A5.2, F49A5.3, F49A5.5, F49A5.7, F49A5.9, F49C12.1, F49C12.4, F49H6.13, F49H6.3, F52C6.4, F52D10.5, F53B1.2, F53B2.2, F53B6.1, F53B6.4, F53B6.9, F53B7.5, F53C3.3, F53C3.4, F53C3.5, F53C3.6, F53F10.8, F53H2.2, F54B11.3, F54E2.5, F54F2.9, F54F7.4, F55A11.7, F55A3.1, F55B12.2, F55C12.5, F55D12.6, F55G1.6, F55H12.3, F56A8.1, F56A8.3, F56B3.11, F56B3.6, F56C3.6, F56D5.9, F56F3.2, F56F4.1, F56H1.2, F56H1.3, F57A8.2, F57B1.1, F57B1.8, F57B1.9, F57C7.4, F57G4.1, F57H12.2, F58A6.5, F58E6.12, F58F6.5, F58F6.6, F58G4.4, F58H1.6, F59B10.1, F59C6.2, F59D6.6, F59F4.2, F59G1.2, F59H6.4, H04J21.1, H04J21.3, H04M03.2, H06I04.2, H10E21.5, H19J13.1, H20E11.1, H20J04.1, H20J04.6, H22K11.4, H32C10.3, JC8.5, K01A2.1, K01D12.1, K01D12.6, K02B12.3, K02D7.5, K02E10.4, K02F3.8, K02F6.3, K02F6.4, K02G10.1, K02G10.6, K02G10.8, K03A11.4, K03E5.1, K03E6.5, K04A8.2, K04F1.12, K04G2.9, K05C4.11, K05C4.2, K05F1.1, K06A4.4, K06A5.2, K06A9.3, K07A1.8, K07A12.2, K07F5.12, K07F5.15, K08D9.6, K08F4.3, K09D9.11, K09E2.4, K09E9.2, K09F5.1, K09F6.4, K09G1.1, K09H9.6, K10C3.3, K10D11.5, K10D2.5, K11C4.2, K11D12.5, K11G12.6, K11H12.3, K12B6.2, K12H6.6, M01D7.6, M01E5.1, M01G5.3, M02B1.3, M02B7.4, M04G7.1, M05D6.5, M176.4, M18.8, M70.1, M70.3, R01B10.5, R01B10.6, R01H2.3, R02F11.2, R03G5.3, R04E5.2, R05D11.5, R05D3.2, R05D7.3, R05H5.5, R07B7.12, R07E5.13, R07E5.7, R08B4.4, R08C7.2, R09B5.10, R09B5.13, R09B5.2, R09B5.3, R09B5.4, R09B5.8, R09B5.9, R105.1, R10D12.5, R10D12.6, R10D12.7, R10D12.9, R10E4.9, R11G1.1, R11G11.12, R11H6.2, R11H6.3, R12C12.6, R13D7.11, R13F6.4, R13F6.5, R13G10.4, R144.6, R151.6, R155.4, R166.2, R186.6, R31.2, T01G9.3, T01H3.4, T02C1.2, T02C5.3, T02E1.7, T03F1.12, T03F6.6, T04A11.3, T04A8.12, T04A8.9, T04G9.3, T05A1.3, T05B4.8, T05C12.9, T06C12.9, T06D8.5, T06D8.7, T06D8.9, T06E4.5, T06E6.5, T07A5.6, T07E3.6, T07F12.2, T08G3.7, T09A12.5, T09A5.12, T09E8.4, T10E9.4, T10E9.6, T10H10.1, T11F9.12, T11F9.21, T12B5.14, T13C2.6, T13G4.3, T13H10.2, T13H5.8, T14A8.1, T14B4.4, T14G10.6, T14G10.7, T14G12.6, T15B7.2, T15D6.11, T16A1.2, T16A1.7, T16A9.5, T16G12.5, T19A6.1, T19A6.3, T19A6.4, T19B10.5, T19C3.4, T19D12.4, T19H12.8, T20B3.13, T20B3.15, T20D3.6, T20D4.13, T20H9.6, T21C9.1, T21C9.12, T21D12.9, T21E3.3, T22A3.6, T22C1.3, T22E7.1, T22E7.2, T22H2.6, T22H9.2, T23B12.5, T23B3.2, T23D8.2, T23F2.3, T23F2.5, T24B1.1, T24C4.4, T24C4.7, T24C4.8, T24F1.2, T24H7.1, T25D10.2, T25E12.10, T25E12.7, T25E12.8, T25E12.9, T25F10.3, T25G12.6, T26H2.2, T27C5.8, T27F6.6, T28D6.9, W01C9.3, W01D2.3, W01G7.5, W02A2.5, W02A2.9, W02B12.15, W02B3.6, W02B8.3, W02B8.4, W02C12.1, W02D3.4, W02D9.2, W02H5.4, W02H5.5, W03A5.2, W03D8.10, W03D8.9, W03G11.2, W04B5.2, W04G5.5, W05G11.2, W06A7.3, W06A7.4, W06G6.7, W06H3.1, W09B7.3, W09G3.8, W10C8.6, W10G11.5, Y102A11A.6, Y102A11A.8, Y102A5B.1, Y105C5B.23, Y106G6H.8, Y108G3AL.2, Y110A2AR.1, Y113G7B.12, Y116A8C.9, Y119C1B.3, Y119C1B.5, Y11D7A.3, Y17G7B.19, Y18D10A.12, Y18H1A.14, Y19D10B.5, Y22D7AL.11, Y22D7AL.15, Y22D7AR.14, Y22D7AR.2, Y23H5A.5, Y2H9A.4, Y32G9A.8, Y32H12A.5, Y34D9A.8, Y34F4.1, Y37A1A.2, Y37D8A.17, Y37D8A.26, Y37D8A.5, Y37D8A.6, Y37D8A.8, Y38F1A.2, Y38F1A.8, Y38F2AR.9, Y39A1A.22, Y39A1A.8, Y39A3A.3, Y39B6A.27, Y39B6A.29, Y39B6A.6, Y39B6A.8, Y39C12A.8, Y39D8A.1, Y39D8B.1, Y39D8B.3, Y39E4B.3, Y39E4B.4, Y39E4B.7, Y40H7A.11, Y41C4A.11, Y41D4A.4, Y42H9AR.2, Y43F8B.10, Y43H11AL.2, Y45F10B.1, Y45G12B.2, Y45G12C.1, Y46B2A.3, Y46G5A.8, Y46H3D.4, Y47D3B.11, Y47D3B.7, Y47D7A.14, Y47G6A.31, Y47H9A.1, Y47H9C.2, Y48E1B.2, Y48G1BM.9, Y48G8AL.13, Y49F6B.9, Y4C6A.3, Y50D4B.4, Y50D4B.5, Y50D4B.7, Y50E8A.17, Y51A2B.2, Y52B11A.7, Y52D5A.1, Y52E8A.4, Y53C10A.5, Y53C12A.3, Y53F4B.25, Y53G8AM.4, Y54F10AL.1, Y54F10AM.7, Y54F10BM.6, Y54G2A.2, Y55B1BM.1, Y55F3AR.1, Y55F3AR.2, Y57A10A.3, Y57A10B.1, Y57E12AL.1, Y57E12AM.1, Y57G11C.37, Y57G7A.6, Y59C2A.2, Y59E1A.1, Y60A3A.19, Y60C6A.1, Y62E10A.10, Y63D3A.6, Y63D3A.8, Y64G10A.6, Y66D12A.21, Y67A10A.3, Y67A10A.8, Y67H2A.4, Y69A2AR.31, Y69E1A.1, Y69H2.1, Y6B3B.10, Y6B3B.3, Y70G10A.2, Y71F9AR.1, Y71F9B.8, Y71G12B.23, Y71H2AM.2, Y71H9A.2, Y71H9A.3, Y73B6BR.1, Y73E7A.6, Y74C10AL.2, Y75B7AL.1, Y75B8A.5, Y77E11A.4, Y87G2A.13, Y87G2A.18, Y87G2A.19, Y97E10AR.6, ZC13.1, ZC190.1, ZC196.5, ZC196.8, ZC196.9, ZC262.10, ZC262.3, ZC262.9, ZC328.3, ZC482.3, ZC482.7, ZK1010.6, ZK1025.3, ZK1037.6, ZK1053.7, ZK1055.4, ZK1067.4, ZK1236.7, ZK185.4, ZK265.9, ZK381.4, ZK381.8, ZK418.3, ZK418.5, ZK6.8, ZK616.6, ZK632.10, ZK632.6, ZK682.5, ZK686.3, ZK757.1, ZK757.4, ZK858.5, ZK858.6, ZK899.2, ZK930.2

**CHANNELS**

**(102) ligand-gated ion channel (LGIC)** (B0491.4, D2062.12, T01H10.3, T01H10.5, T01H10.6, T19D7.1, Y55F3BR.4, Y74E4A.1 from no other domain)

B0207.12, B0491.4, C02C2.3, C04C3.2, C09G5.1, C15A7.1, C15A7.4, C27H5.8, C31H5.3, C33G3.3, C35C5.5, C39B10.2, C40C9.2, C43F9.9, C45B2.4, C50B6.11, C53D6.3, D2062.12, D2092.3, F07B10.5, F09C12.1, F09E8.7, F09G2.5, F11A5.10, F11C7.1, F11H8.2, F12B6.3, F15E6.2, F17E9.7, F17E9.8, F18G5.4, F21A3.7, F21F3.5, F25F8.2, F25G6.3, F25G6.4, F27B3.2, F28F8.1, F46F3.2, F47A4.1, F48E3.7, F53E10.2, F55D10.5, F58G6.4, F58H7.3, F59B1.9, H35N03.1, K03B8.9, K03F8.2, K06C4.6, K10D6.1, K11G12.2, K11G12.7, R01E6.4, R02E12.8, R03E1.3, R06A4.10, R11G10.1, R13A5.4, T01H10.1, T01H10.2, T01H10.3, T01H10.5, T01H10.6, T01H10.7, T05B4.1, T05C12.2, T08G11.5, T09A5.3, T15B7.16, T19D7.1, T20B12.9, T21C12.1, T21F2.1, T24D8.1, T26H10.1, T27A1.4, T27E9.9, W10G11.16, Y105C5B.16, Y110A7A.3, Y113G7A.5, Y39A3B.2, Y44A6E.1, Y45G5AL.2, Y46G5A.26, Y48B6A.4, Y55F3BR.4, Y57G11C.2, Y57G11C.49, Y58G8A.1, Y71D11A.5, Y73B6BL.26, Y73B6BL.42, Y73F8A.2, Y73F8A.30, Y74E4A.1, ZC317.3, ZC482.1, ZC482.5, ZC504.2, ZK973.5

**(92) domain:Neur_chan_LBD (PF02931)** Neurotransmitter-gated ion-channel ligand binding domain

B0207.12, C02C2.3, C04C3.2, C09G5.1, C15A7.1, C15A7.4, C27H5.8, C31H5.3, C33G3.3, C35C5.5, C39B10.2, C40C9.2, C43F9.9, C45B2.4, C50B6.11, C53D6.3, D2092.3, F07B10.5, F09C12.1, F09E8.7, F09G2.5, F11A5.10, F11C7.1, F11H8.2, F17E9.7, F17E9.8, F18G5.4, F21A3.7, F21F3.5, F25F8.2, F25G6.3, F25G6.4, F27B3.2, F28F8.1, F46F3.2, F47A4.1, F48E3.7, F53E10.2, F55D10.5, F58G6.4, F58H7.3, F59B1.9, H35N03.1, K03B8.9, K03F8.2, K06C4.6, K10D6.1, K11G12.2, K11G12.7, R01E6.4, R02E12.8, R03E1.3, R06A4.10, R11G10.1, R13A5.4, T01H10.1, T01H10.2, T01H10.7, T05B4.1, T05C12.2, T08G11.5, T09A5.3, T15B7.16, T20B12.9, T21C12.1, T21F2.1, T24D8.1, T26H10.1, T27A1.4, T27E9.9, W10G11.16, Y105C5B.16, Y110A7A.3, Y113G7A.5, Y39A3B.2, Y44A6E.1, Y45G5AL.2, Y46G5A.26, Y48B6A.4, Y57G11C.2, Y57G11C.49, Y58G8A.1, Y71D11A.5, Y73B6BL.26, Y73B6BL.42, Y73F8A.2, Y73F8A.30, ZC317.3, ZC482.1, ZC482.5, ZC504.2, ZK973.5

**(75) domain:Neur_chan_memb (PF02932)** Neurotransmitter-gated ion-channel transmembrane region

B0207.12, C04C3.2, C09G5.1, C27H5.8, C31H5.3, C35C5.5, C39B10.2, C40C9.2, C45B2.4, C50B6.11, C53D6.3, D2092.3, F07B10.5, F09C12.1, F09E8.7, F09G2.5, F11A5.10, F11C7.1, F11H8.2, F18G5.4, F21A3.7, F21F3.5, F25F8.2, F25G6.3, F25G6.4, F27B3.2, F28F8.1, F46F3.2, F47A4.1, F48E3.7, F53E10.2, F55D10.5, F58G6.4, F59B1.9, H35N03.1, K03B8.9, K03F8.2, K06C4.6, K10D6.1, K11G12.2, K11G12.7, R01E6.4, R02E12.8, R06A4.10, R11G10.1, R13A5.4, T05C12.2, T08G11.5, T09A5.3, T15B7.16, T20B12.9, T21C12.1, T21F2.1, T24D8.1, T26H10.1, T27E9.9, W10G11.16, Y110A7A.3, Y113G7A.5, Y39A3B.2, Y44A6E.1, Y46G5A.26, Y48B6A.4, Y57G11C.2, Y57G11C.49, Y71D11A.5, Y73B6BL.26, Y73B6BL.42, Y73F8A.2, Y73F8A.30, ZC317.3, ZC482.1, ZC482.5, ZC504.2, ZK973.5

**(53) gene:lgc-***

B0491.4, C04C3.2, C15A7.1, C15A7.4, C33G3.3, C39B10.2, C43F9.9, C50B6.11, D2062.12, F07B10.5, F09G2.5, F11H8.2, F12B6.3, F15E6.2, F17E9.7, F17E9.8, F18G5.4, F21A3.7, F46F3.2, F47A4.1, F48E3.7, F58H7.3, K10D6.1, R03E1.3, R13A5.4, T01H10.1, T01H10.2, T01H10.3, T01H10.5, T01H10.6, T01H10.7, T05B4.1, T15B7.16, T19D7.1, T20B12.9, T21F2.1, T24D8.1, T27A1.4, W10G11.16, Y105C5B.16, Y113G7A.5, Y39A3B.2, Y45G5AL.2, Y46G5A.26, Y55F3BR.4, Y57G11C.2, Y57G11C.49, Y58G8A.1, Y71D11A.5, Y73B6BL.26, Y73F8A.2, Y74E4A.1, ZC482.5

**(29) acetylcholine receptor** (Jones et al., 2007)

C31H5.3, C35C5.5, C40C9.2, D2092.3, F09E8.7, F21F3.5, F25G6.3, F25G6.4, F27B3.2, F28F8.1, F53E10.2, F59B1.9, K03B8.9, K03F8.2, K11G12.2, K11G12.7, R01E6.4, R02E12.8, R06A4.10, T05C12.2, T08G11.5, T09A5.3, T26H10.1, Y110A7A.3, Y48B6A.4, Y73B6BL.42, Y73F8A.30, ZC504.2, ZK973.5

**(71) potassium channels**

B0334.2, B0399.1, C03F11.1, C24A3.6, C24H11.8, C25B8.1, C30D11.1, C32C4.1, C33D12.3, C40C9.1, C44E12.3, C48E7.9, C52B9.6, C53A5.5, C53C9.3, F08A10.1, F08B12.3, F12F3.1, F14F11.1, F16B3.1, F17C8.5, F19D8.1, F20A1.7, F21C3.1, F22B7.7, F29F11.4, F31D4.7, F32H5.7, F33D11.5, F34D6.3, F36A2.4, F44A2.2, F46A9.3, F52E4.4, F53C11.6, F55C5.3, K01D12.4, K04A8.4, K04G11.5, K06B4.12, K11H3.7, M02A10.2, M04B2.5, M110.2, M60.5, R03E9.4, R04F11.4, R07A4.1, R12G8.2, R186.5, T01B4.1, T01B4.2, T06H11.1, T12C9.3, T28A8.1, W06D12.2, W06D12.5, Y37A1B.11, Y39B6A.19, Y47D3B.5, Y48A6B.6, Y51A2D.19, Y54G9A.3, Y55F3C.3, Y71H2AM.22, Y71H9A.1, Y73B6BL.19, ZC410.4, ZK1067.5, ZK1251.8, ZK1321.2

**(Salkoff et al., 2005 and http://nt-salkoff.wustl.edu/)**

**(11) voltage-gated potassium channel**

C32C4.1, C53C9.3, F12F3.1, F14F11.1, F44A2.2, R07A4.1, R186.5, Y48A6B.6, Y55F3C.3, Y73B6BL.19, ZK1321.2

**(3) 2TM inward rectifiers**

K04G11.5, M02A10.2, R03E9.4

**(3) KCNQ-like potassium channel subunits**

C25B8.1, M60.5, Y54G9A.3

**(2) eag-like K channels**

C30D11.1, F16B3.1

**(2) calcium-activated Slo channels**

F08B12.3, Y51A2D.19

**(4) SK calcium-activated K+ channels**

B0399.1, C03F11.1, C53A5.5, F08A10.1

**(44) 4TM K+ channels**

B0334.2, C24A3.6, C24H11.8, C33D12.3, C40C9.1, C44E12.3, C48E7.9, C52B9.6, F17C8.5, F19D8.1, F20A1.7, F21C3.1, F22B7.7, F29F11.4, F31D4.7, F32H5.7, F34D6.3, F36A2.4, F46A9.3, F52E4.4, F53C11.6, F55C5.3, K01D12.4, K04A8.4, K06B4.12, M04B2.5, M110.2, R04F11.4, R12G8.2, T01B4.1, T01B4.2, T06H11.1, T12C9.3, T28A8.1, W06D12.2, W06D12.5, Y37A1B.11, Y39B6A.19, Y47D3B.5, Y71H2AM.22, Y71H9A.1, ZC410.4, ZK1067.5, ZK1251.8

**(51) domain:K_chan_2pore (PF07885)** voltage-gated potassium channel 2 pore domain

B0334.2, B0399.1, C03F11.1, C24A3.6, C24H11.8, C33D12.3, C40C9.1, C44E12.3, C48E7.9, C52B9.6, C53A5.5, F08A10.1, F08B12.3, F17C8.5, F19D8.1, F20A1.7, F21C3.1, F22B7.7, F29F11.4, F31D4.7, F32H5.7, F33D11.5, F34D6.3, F36A2.4, F46A9.3, F52E4.4, F53C11.6, F55C5.3, K01D12.4, K04A8.4, K06B4.12, K11H3.7, M04B2.5, M110.2, R04F11.4, R12G8.2, T01B4.1, T01B4.2, T06H11.1, T12C9.3, T28A8.1, W06D12.2, W06D12.5, Y37A1B.11, Y39B6A.19, Y47D3B.5, Y71H2AM.22, Y71H9A.1, ZC410.4, ZK1067.5, ZK1251.8

**(46) sodium channels**

B0395.1, B0495.4, C07A9.11, C07A9.4, C10G8.5, C11E4.3, C13D9.7, C13D9.8, C18B2.6, C24G7.1, C24G7.2, C24G7.4, C27C12.5, C41C4.5, C46A5.2, C47C12.6, C54F6.13, E02H4.1, F02D10.5, F14B8.1, F16F9.5, F23B2.3, F25D1.4, F28A12.1, F35C12.2, F41E7.1, F41E7.2, F55G1.12, F57C7.2, F57G8.5, F58E1.6, K09C8.1, R13A1.4, T01C8.7, T21C9.3, T28B8.5, T28D9.7, T28F2.7, T28F4.2, Y113G7A.4, Y18D10A.6, Y32F6B.2, Y97E10B.7, ZC168.1, ZK770.1, ZK822.3

**(24) domain:Na_channels_AS (PF00858)** amiloride-sensitive sodium channel

C11E4.3, C18B2.6, C24G7.1, C24G7.2, C24G7.4, C27C12.5, C41C4.5, C46A5.2, C47C12.6, E02H4.1, F02D10.5, F16F9.5, F23B2.3, F25D1.4, F28A12.1, F55G1.12, R13A1.4, T01C8.7, T21C9.3, T28B8.5, T28D9.7, T28F2.7, T28F4.2, ZK770.1

**(12) domain:Na_H_Exchanger (PF00999)** sodium/hydrogen exchanger family

B0395.1, B0495.4, C54F6.13, F14B8.1, F41E7.1, F41E7.2, F57C7.2, F57G8.5, F58E1.6, K09C8.1, Y18D10A.6, ZK822.3

**(10) domain:Na_Ca_ex (PF01699)** sodium/calcium exchanger protein

C07A9.11, C07A9.4, C10G8.5, C13D9.7, C13D9.8, F35C12.2, Y113G7A.4, Y32F6B.2, Y97E10B.7, ZC168.1

**(27) domain:Ion_trans (PF00520)** four TM helices additionally present in many channels (C25B8.1, C30D11.1, C32C4.1, C53C9.3, F12F3.1, F14F11.1, F16B3.1, F44A2.2, M60.5, R07A4.1, R186.5, Y48A6B.6, Y51A2D.19, Y54G9A.3, Y55F3C.3, Y73B6BL.19, ZK1321.2 moved to potassium channel)

B0212.5, C05C12.3, C11D2.6, C23H5.7, C27F2.2, C29E6.2, C48A7.1, C54D2.5, F14H8.6, F28H7.10, F33D4.2, F36F2.5, F38E11.12, F54D1.5, K01A11.4, K11C4.5, R06B10.4, R13A5.1, T01H8.5, T02C5.5, T09A12.3, T10B10.7, Y40C5A.2, Y71A12B.4, Y76B12C.1, ZC21.2, ZC84.2

**(25) domain:innexin (PF00876)** gap junction component in invertebrates

C16E9.4, C18H7.2, C36H8.2, F07A5.1, F08G12.10, F13G3.8, F22F4.2, F26D11.10, K02B2.4, R07D5.1, R09F10.4, R12E2.4, R12E2.5, R12E2.9, R12H7.1, T16H5.1, T18H9.5, T23H4.1, W04D2.3, Y47G6A.1, Y47G6A.2, Y8G1A.2, ZK770.3, ZK792.2, ZK792.3

**(25) domain:Bestrophin (PF01062)** chloride channel

B0564.3, B0564.4, C01B12.3, C01B12.5, C07A9.8, C09B9.3, C29F4.2, C37A5.1, C43G2.4, C49A1.2, C49A1.3, F14H3.2, F32B6.9, F32G8.4, R13.3, R13A1.9, T19C3.1, T20G5.4, Y37E11AR.1, Y73F8A.11, ZC518.1, ZK675.3, ZK688.2, ZK849.4, ZK849.5

**(6) domain:Voltage_CLC (PF00654)** voltage gated chloride channel

B0491.8, C07H4.2, E04F6.11, R07B7.1, T06F4.2, T27D12.2

**(12) aquaporins** gene:aqp-*, domain:MIP (PF00230) (K07A1.16, Y57A10A.35, ZK1321.3, ZK525.2 from no other domain)

C01G6.1, C32C4.2, C35A5.1, F32A5.5, F40F9.9, K02G10.7, K07A1.16, M02F4.8, Y57A10A.35, Y69E1A.7, ZK1321.3, ZK525.2

**(10) gene:glr-*, nmr-*, domain:PBPe (SM00079)** GLutamate Receptor, NMDA class glutamate Receptor, Eukaryotic homologues of bacterial periplasmic substrate binding proteins

B0280.12, C06A8.9, C06E1.4, C43H6.9, F07F6.6, F22A3.3, F41B4.4, K10D3.1, T01C3.10, ZC196.7

**(243) GO: channel_activity** (F20B6.3, F56F4.5 were moved to transporters)

B0207.12, B0212.5, B0280.12, B0334.2, B0399.1, B0491.8, C02C2.3, C03F11.1, C04C3.2, C05C12.3, C06A8.9, C06E1.4, C07H4.2, C09G5.1, C11D2.6, C11E4.3, C15A7.1, C15A7.4, C18B2.6, C23H5.7, C24A3.6, C24G7.1, C24G7.2, C24G7.4, C25B8.1, C27C12.5, C27F2.2, C27H5.8, C29E6.2, C30D11.1, C31H5.3, C32C4.1, C33D12.3, C33G3.3, C35C5.5, C39B10.2, C40C9.1, C40C9.2, C41C4.5, C43F9.9, C43H6.9, C44E12.3, C45B2.4, C46A5.2, C47C12.6, C48A7.1, C48E7.9, C50B6.11, C52B9.6, C53A5.5, C53C9.3, C53D6.3, C54D2.5, D2092.3, E02H4.1, E04F6.11, F02D10.5, F07B10.5, F07F6.6, F08A10.1, F08B12.3, F09C12.1, F09E8.7, F09G2.5, F11A5.10, F11C7.1, F11H8.2, F12B6.3, F12F3.1, F14F11.1, F14H8.6, F15E6.2, F16B3.1, F16F9.5, F17C8.5, F17E9.7, F17E9.8, F18G5.4, F19D8.1, F20A1.7, F21A3.7, F21C3.1, F21F3.5, F22A3.3, F22B7.7, F22E5.11, F23B2.3, F25D1.4, F25F8.2, F25G6.3, F25G6.4, F27B3.2, F28A12.1, F28F8.1, F28H7.10, F29F11.4, F31D4.7, F32H5.7, F33D4.2, F34D6.3, F36A2.4, F36F2.5, F38E11.12, F41B4.4, F44A2.2, F46A9.3, F46F3.2, F47A4.1, F48E3.7, F52E4.4, F53C11.6, F53E10.2, F54D1.5, F55C5.3, F55D10.5, F55G1.12, F58G6.4, F58H7.3, F59B1.9, H35N03.1, K01A11.4, K01D12.4, K03B8.9, K03F8.2, K04A8.4, K04G11.5, K06B4.12, K06C4.6, K10D3.1, K10D6.1, K11C4.5, K11G12.2, K11G12.7, K11H3.7, M02A10.2, M04B2.5, M110.2, M60.5, R01E6.4, R02E12.8, R03E1.3, R03E9.4, R04F11.4, R06A4.10, R06B10.4, R07A4.1, R07B7.1, R11G10.1, R12G8.2, R13A1.4, R13A5.1, R13A5.4, R186.5, T01B4.1, T01B4.2, T01C3.10, T01C8.7, T01H10.1, T01H10.2, T01H10.7, T01H8.5, T02C5.5, T05B4.1, T05C12.2, T05F1.1, T06F4.2, T06H11.1, T08G11.5, T09A12.3, T09A5.3, T10B10.7, T12C9.3, T15B7.16, T20B12.9, T21C12.1, T21C9.3, T21F2.1, T24D8.1, T26H10.1, T27A1.4, T27D12.2, T27E9.9, T28A8.1, T28B8.5, T28D9.7, T28F2.7, T28F4.2, W06D12.2, W06D12.5, W10G11.16, Y105C5B.16, Y110A7A.3, Y113G7A.5, Y37A1B.11, Y39A3B.2, Y39B6A.19, Y40C5A.2, Y44A6E.1, Y45G5AL.2, Y46G5A.26, Y47D3B.5, Y48A6B.6, Y48B6A.4, Y51A2D.19, Y54G9A.3, Y55F3C.3, Y57A10A.10, Y57A10A.28, Y57G11C.2, Y57G11C.49, Y58G8A.1, Y69H2.11, Y69H2.12, Y69H2.2, Y71A12B.4, Y71D11A.5, Y71H2AM.22, Y71H9A.1, Y73B6BL.19, Y73B6BL.26, Y73B6BL.42, Y73F8A.1, Y73F8A.2, Y73F8A.30, Y76B12C.1, ZC196.7, ZC21.2, ZC239.16, ZC239.17, ZC239.5, ZC317.3, ZC410.4, ZC482.1, ZC482.5, ZC504.2, ZC84.2, ZK1067.5, ZK1251.8, ZK1321.2, ZK770.1, ZK770.3, ZK973.5

**(12) from "no other domain", not grouped in any of above**

C04F5.1, C29F5.4, F09G8.9, F26A3.6, F32D8.15, F58G6.8, F59F3.4, K01A2.8, T06A4.2, Y57G11C.44, ZK512.3, ZK520.2

**(347) CHANNELS combined**

B0207.12, B0212.5, B0280.12, B0334.2, B0395.1, B0399.1, B0491.4, B0491.8, B0495.4, B0564.3, B0564.4, C01B12.3, C01B12.5, C01G6.1, C02C2.3, C03F11.1, C04C3.2, C04F5.1, C05C12.3, C06A8.9, C06E1.4, C07A9.11, C07A9.4, C07A9.8, C07H4.2, C09B9.3, C09G5.1, C10G8.5, C11D2.6, C11E4.3, C13D9.7, C13D9.8, C15A7.1, C15A7.4, C16E9.4, C18B2.6, C18H7.2, C23H5.7, C24A3.6, C24G7.1, C24G7.2, C24G7.4, C24H11.8, C25B8.1, C27C12.5, C27F2.2, C27H5.8, C29E6.2, C29F4.2, C29F5.4, C30D11.1, C31H5.3, C32C4.1, C32C4.2, C33D12.3, C33G3.3, C35A5.1, C35C5.5, C36H8.2, C37A5.1, C39B10.2, C40C9.1, C40C9.2, C41C4.5, C43F9.9, C43G2.4, C43H6.9, C44E12.3, C45B2.4, C46A5.2, C47C12.6, C48A7.1, C48E7.9, C49A1.2, C49A1.3, C50B6.11, C52B9.6, C53A5.5, C53C9.3, C53D6.3, C54D2.5, C54F6.13, D2062.12, D2092.3, E02H4.1, E04F6.11, F02D10.5, F07A5.1, F07B10.5, F07F6.6, F08A10.1, F08B12.3, F08G12.10, F09C12.1, F09E8.7, F09G2.5, F09G8.9, F11A5.10, F11C7.1, F11H8.2, F12B6.3, F12F3.1, F13G3.8, F14B8.1, F14F11.1, F14H3.2, F14H8.6, F15E6.2, F16B3.1, F16F9.5, F17C8.5, F17E9.7, F17E9.8, F18G5.4, F19D8.1, F20A1.7, F21A3.7, F21C3.1, F21F3.5, F22A3.3, F22B7.7, F22E5.11, F22F4.2, F23B2.3, F25D1.4, F25F8.2, F25G6.3, F25G6.4, F26A3.6, F26D11.10, F27B3.2, F28A12.1, F28F8.1, F28H7.10, F29F11.4, F31D4.7, F32A5.5, F32B6.9, F32D8.15, F32G8.4, F32H5.7, F33D11.5, F33D4.2, F34D6.3, F35C12.2, F36A2.4, F36F2.5, F38E11.12, F40F9.9, F41B4.4, F41E7.1, F41E7.2, F44A2.2, F46A9.3, F46F3.2, F47A4.1, F48E3.7, F52E4.4, F53C11.6, F53E10.2, F54D1.5, F55C5.3, F55D10.5, F55G1.12, F57C7.2, F57G8.5, F58E1.6, F58G6.4, F58G6.8, F58H7.3, F59B1.9, F59F3.4, H35N03.1, K01A11.4, K01A2.8, K01D12.4, K02B2.4, K02G10.7, K03B8.9, K03F8.2, K04A8.4, K04G11.5, K06B4.12, K06C4.6, K07A1.16, K09C8.1, K10D3.1, K10D6.1, K11C4.5, K11G12.2, K11G12.7, K11H3.7, M02A10.2, M02F4.8, M04B2.5, M110.2, M60.5, R01E6.4, R02E12.8, R03E1.3, R03E9.4, R04F11.4, R06A4.10, R06B10.4, R07A4.1, R07B7.1, R07D5.1, R09F10.4, R11G10.1, R12E2.4, R12E2.5, R12E2.9, R12G8.2, R12H7.1, R13.3, R13A1.4, R13A1.9, R13A5.1, R13A5.4, R186.5, T01B4.1, T01B4.2, T01C3.10, T01C8.7, T01H10.1, T01H10.2, T01H10.3, T01H10.5, T01H10.6, T01H10.7, T01H8.5, T02C5.5, T05B4.1, T05C12.2, T05F1.1, T06A4.2, T06F4.2, T06H11.1, T08G11.5, T09A12.3, T09A5.3, T10B10.7, T12C9.3, T15B7.16, T16H5.1, T18H9.5, T19C3.1, T19D7.1, T20B12.9, T20G5.4, T21C12.1, T21C9.3, T21F2.1, T23H4.1, T24D8.1, T26H10.1, T27A1.4, T27D12.2, T27E9.9, T28A8.1, T28B8.5, T28D9.7, T28F2.7, T28F4.2, W04D2.3, W06D12.2, W06D12.5, W10G11.16, Y105C5B.16, Y110A7A.3, Y113G7A.4, Y113G7A.5, Y18D10A.6, Y32F6B.2, Y37A1B.11, Y37E11AR.1, Y39A3B.2, Y39B6A.19, Y40C5A.2, Y44A6E.1, Y45G5AL.2, Y46G5A.26, Y47D3B.5, Y47G6A.1, Y47G6A.2, Y48A6B.6, Y48B6A.4, Y51A2D.19, Y54G9A.3, Y55F3BR.4, Y55F3C.3, Y57A10A.10, Y57A10A.28, Y57A10A.35, Y57G11C.2, Y57G11C.44, Y57G11C.49, Y58G8A.1, Y69E1A.7, Y69H2.11, Y69H2.12, Y69H2.2, Y71A12B.4, Y71D11A.5, Y71H2AM.22, Y71H9A.1, Y73B6BL.19, Y73B6BL.26, Y73B6BL.42, Y73F8A.1, Y73F8A.11, Y73F8A.2, Y73F8A.30, Y74E4A.1, Y76B12C.1, Y8G1A.2, Y97E10B.7, ZC168.1, ZC196.7, ZC21.2, ZC239.16, ZC239.17, ZC239.5, ZC317.3, ZC410.4, ZC482.1, ZC482.5, ZC504.2, ZC518.1, ZC84.2, ZK1067.5, ZK1251.8, ZK1321.2, ZK1321.3, ZK512.3, ZK520.2, ZK525.2, ZK675.3, ZK688.2, ZK770.1, ZK770.3, ZK792.2, ZK792.3, ZK822.3, ZK849.4, ZK849.5, ZK973.5

**(343) CHANNELS combined, curated** (moved B0212.5, F33D4.2 and T05F1.1 to signaling; moved R13A5.1 to TRAFFICKING)

B0207.12, B0280.12, B0334.2, B0395.1, B0399.1, B0491.4, B0491.8, B0495.4, B0564.3, B0564.4, C01B12.3, C01B12.5, C01G6.1, C02C2.3, C03F11.1, C04C3.2, C04F5.1, C05C12.3, C06A8.9, C06E1.4, C07A9.11, C07A9.4, C07A9.8, C07H4.2, C09B9.3, C09G5.1, C10G8.5, C11D2.6, C11E4.3, C13D9.7, C13D9.8, C15A7.1, C15A7.4, C16E9.4, C18B2.6, C18H7.2, C23H5.7, C24A3.6, C24G7.1, C24G7.2, C24G7.4, C24H11.8, C25B8.1, C27C12.5, C27F2.2, C27H5.8, C29E6.2, C29F4.2, C29F5.4, C30D11.1, C31H5.3, C32C4.1, C32C4.2, C33D12.3, C33G3.3, C35A5.1, C35C5.5, C36H8.2, C37A5.1, C39B10.2, C40C9.1, C40C9.2, C41C4.5, C43F9.9, C43G2.4, C43H6.9, C44E12.3, C45B2.4, C46A5.2, C47C12.6, C48A7.1, C48E7.9, C49A1.2, C49A1.3, C50B6.11, C52B9.6, C53A5.5, C53C9.3, C53D6.3, C54D2.5, C54F6.13, D2062.12, D2092.3, E02H4.1, E04F6.11, F02D10.5, F07A5.1, F07B10.5, F07F6.6, F08A10.1, F08B12.3, F08G12.10, F09C12.1, F09E8.7, F09G2.5, F09G8.9, F11A5.10, F11C7.1, F11H8.2, F12B6.3, F12F3.1, F13G3.8, F14B8.1, F14F11.1, F14H3.2, F14H8.6, F15E6.2, F16B3.1, F16F9.5, F17C8.5, F17E9.7, F17E9.8, F18G5.4, F19D8.1, F20A1.7, F21A3.7, F21C3.1, F21F3.5, F22A3.3, F22B7.7, F22E5.11, F22F4.2, F23B2.3, F25D1.4, F25F8.2, F25G6.3, F25G6.4, F26A3.6, F26D11.10, F27B3.2, F28A12.1, F28F8.1, F28H7.10, F29F11.4, F31D4.7, F32A5.5, F32B6.9, F32D8.15, F3\2G8.4, F32H5.7, F33D11.5, F34D6.3, F35C12.2, F36A2.4, F36F2.5, F38E11.12, F40F9.9, F41B4.4, F41E7.1, F41E7.2, F44A2.2, F46A9.3, F46F3.2, F47A4.1, F48E3.7, F52E4.4, F53C11.6, F53E10.2, F54D1.5, F55C5.3, F55D10.5, F55G1.12, F57C7.2, F57G8.5, F58E1.6, F58G6.4, F58G6.8, F58H7.3, F59B1.9, F59F3.4, H35N03.1, K01A11.4, K01A2.8, K01D12.4, K02B2.4, K02G10.7, K03B8.9, K03F8.2, K04A8.4, K04G11.5, K06B4.12, K06C4.6, K07A1.16, K09C8.1, K10D3.1, K10D6.1, K11C4.5, K11G12.2, K11G12.7, K11H3.7, M02A10.2, M02F4.8, M04B2.5, M110.2, M60.5, R01E6.4, R02E12.8, R03E1.3, R03E9.4, R04F11.4, R06A4.10, R06B10.4, R07A4.1, R07B7.1, R07D5.1, R09F10.4, R11G10.1, R12E2.4, R12E2.5, R12E2.9, R12G8.2, R12H7.1, R13.3, R13A1.4, R13A1.9, R13A5.4, R186.5, T01B4.1, T01B4.2, T01C3.10, T01C8.7, T01H10.1, T01H10.2, T01H10.3, T01H10.5, T01H10.6, T01H10.7, T01H8.5, T02C5.5, T05B4.1, T05C12.2, T06A4.2, T06F4.2, T06H11.1, T08G11.5, T09A12.3, T09A5.3, T10B10.7, T12C9.3, T15B7.16, T16H5.1, T18H9.5, T19C3.1, T19D7.1, T20B12.9, T20G5.4, T21C12.1, T21C9.3, T21F2.1, T23H4.1, T24D8.1, T26H10.1, T27A1.4, T27D12.2, T27E9.9, T28A8.1, T28B8.5, T28D9.7, T28F2.7, T28F4.2, W04D2.3, W06D12.2, W06D12.5, W10G11.16, Y105C5B.16, Y110A7A.3, Y113G7A.4, Y113G7A.5, Y18D10A.6, Y32F6B.2, Y37A1B.11, Y37E11AR.1, Y39A3B.2, Y39B6A.19, Y40C5A.2, Y44A6E.1, Y45G5AL.2, Y46G5A.26, Y47D3B.5, Y47G6A.1, Y47G6A.2, Y48A6B.6, Y48B6A.4, Y51A2D.19, Y54G9A.3, Y55F3BR.4, Y55F3C.3, Y57A10A.10, Y57A10A.28, Y57A10A.35, Y57G11C.2, Y57G11C.44, Y57G11C.49, Y58G8A.1, Y69E1A.7, Y69H2.11, Y69H2.12, Y69H2.2, Y71A12B.4, Y71D11A.5, Y71H2AM.22, Y71H9A.1, Y73B6BL.19, Y73B6BL.26, Y73B6BL.42, Y73F8A.1, Y73F8A.11, Y73F8A.2, Y73F8A.30, Y74E4A.1, Y76B12C.1, Y8G1A.2, Y97E10B.7, ZC168.1, ZC196.7, ZC21.2, ZC239.16, ZC239.17, ZC239.5, ZC317.3, ZC410.4, ZC482.1, ZC482.5, ZC504.2, ZC518.1, ZC84.2, ZK1067.5, ZK1251.8, ZK1321.2, ZK1321.3, ZK512.3, ZK520.2, ZK525.2, ZK675.3, ZK688.2, ZK770.1, ZK770.3, ZK792.2, ZK792.3, ZK822.3, ZK849.4, ZK849.5, ZK973.5

**TRANSPORTERS**

**(117) domain:MFS_1 (PF07690)** major facilitator superfamily of transporters (F10G7.5, F14D7.6, T04H1.1, T05A1.5, W03C9.6, ZK563.1 from no other domain) (added C27C12.4)

B0252.3, B0361.11, B0416.5, C01B4.7, C01B4.8, C01B4.9, C02C2.4, C05G5.1, C06H5.6, C09D4.1, C10E2.6, C13C4.5, C13C4.6, C14A6.2, C18D1.2, C18H9.5, C25E10.5, C27C12.4, C35A5.3, C38C10.2, C39E9.10, C39E9.7, C42C1.8, C46C2.2, C49F8.2, C53B4.3, F09A5.1, F10C2.7, F10D7.2, F10G7.5, F11A5.9, F12B6.2, F14B8.3, F14D7.6, F16H11.1, F25G6.7, F27D9.2, F40F9.5, F41C3.2, F44E7.7, F45E4.11, F47B8.10, F52F12.1, F55A4.8, F56A4.10, F56A4.11, F56A4.12, F57A8.7, F58G11.4, F59F5.1, H11E01.2, K05B2.5, K05F1.6, K08C7.1, K09E9.1, K10G9.1, K10H10.1, K11D9.3, K11G9.5, M03B6.2, M117.1, M162.5, R10D12.1, R13A5.9, T01H3.3, T02B11.6, T02G5.12, T04H1.1, T05A1.5, T07A5.3, T09B9.2, T10C6.6, T11G6.2, T11G6.3, T11G6.4, T19D12.10, T19D12.9, T22F3.10, T22F3.11, T22F3.7, T22F3.8, T22F7.1, T25D3.4, T28C12.1, T28C12.2, T28F3.4, T28H11.8, W01B6.3, W01C8.6, W03C9.6, W04C9.6, W05E10.1, Y111B2A.19, Y15E3A.4, Y19D10A.10, Y19D10A.11, Y19D10A.12, Y19D10A.4, Y19D10A.5, Y19D10A.8, Y43F8A.5, Y4C6B.3, Y4C6B.4, Y4C6B.5, Y51A2D.18, Y51B9A.6, Y53G8AR.7, Y57G11C.23, Y66D12A.13, ZC416.8, ZK455.8, ZK512.6, ZK54.1, ZK550.2, ZK563.1, ZK682.2, ZK892.3

**(52) ABC transporters** (Zhao et al. 2004, out of 60 genes (+1 gene added after publication), 6 do not have TM, 3 are pseudogenes) (C56E6.5 from no other domain)

C05A9.1, C05D10.3, C10C6.5, C16C10.12, C18C4.2, C24F3.5, C30H6.6, C34G6.4, C44B7.8, C44B7.9, C47A10.1, C48B4.4, C54D1.1, C54G10.3, C56E6.1, C56E6.5, DH11.3, E03G2.2, F02E11.1, F12B6.1, F14F4.3, F19B6.4, F20B6.3, F21G4.2, F22E10.1, F22E10.2, F22E10.3, F42E11.1, F43E2.4, F57A10.3, F57C12.4, F57C12.5, K08E7.9, T02D1.5, T10H9.5, T21E8.1, T21E8.2, T21E8.3, T26A5.1, W04C9.1, W09D6.6, Y39D8C.1, Y42G9A.6, Y43F8C.12, Y47D3A.11, Y48G8AL.11, Y50E8A.16, Y53C10A.9, Y74C10AR.3, Y75B8A.26, ZK455.7, ZK484.2

**(36) domain:ABC_transporter_tm (PF00664, PF06472)** ABC transporter TM domain

C05A9.1, C30H6.6, C34G6.4, C44B7.8, C44B7.9, C47A10.1, C54D1.1, C54G10.3, DH11.3, E03G2.2, F14F4.3, F20B6.3, F21G4.2, F22E10.1, F22E10.2, F22E10.3, F42E11.1, F43E2.4, F57A10.3, F57C12.4, F57C12.5, K08E7.9, T02D1.5, T10H9.5, T21E8.1, T21E8.2, T21E8.3, W04C9.1, W09D6.6, Y43F8C.12, Y48G8AL.11, Y50E8A.16, Y74C10AR.3, Y75B8A.26, ZK455.7, ZK484.2

**(2) gene:abc*-*** (not include abce-1, abcf-1/2/3 as they do not have apparent TM domain)

C56E6.5, C56E6.1

**(4) gene:abt-*** (not include abt-3 as it is a psedugene, nore abt-6 as it does not have apparent TM domain)

C24F3.5, F12B6.1, Y39D8C.1, Y53C10A.9

**(7) gene:haf-*** (not include haf-8 as it does not have apparent TM domain)

C30H6.6, F43E2.4, F57A10.3, W04C9.1, Y48G8AL.11, Y50E8A.16, ZK484.2

**(8) gene:mrp-***

F57C12.5, F57C12.4, E03G2.2, F21G4.2, F14F4.3, F20B6.3, Y43F8C.12, Y75B8A.26

**(14) gene:pgp-*** (not include pgp-15 as it is a pseudogene)

K08E7.9, C54D1.1, DH11.3, F22E10.1, F22E10.2, F22E10.3, C34G6.4, ZK455.7, F42E11.1, C05A9.1, T21E8.1, T21E8.2, T21E8.3, C47A10.1

**(5) gene:pmp-***

C44B7.8, C44B7.9, C54G10.3, T02D1.5, T10H9.5

**(8) gene:wht-*** (not include wht-9 as it is a pseudogene)

C05D10.3, C10C6.5, C16C10.12, F02E11.1, F19B6.4, T26A5.1, Y42G9A.6, Y47D3A.11

**(4) gene:others** (abtm-1, ced-7, cft-1, hmt-1)

Y74C10AR.3, C48B4.4, C18C4.2, W09D6.6

**(43) domain:Sugar_tr (PF00083)** sugar (and other) transporter (K09C4.4 from no other domain)

C03B1.13, C35A11.4, C44C10.3, C46C11.2, C53B4.1, D1046.4, F11D5.7, F13B12.2, F14E5.1, F17C11.12, F21F8.11, F23F12.13, F23F12.3, F25D1.2, F32H5.4, F36G9.3, F45E10.2, F48E3.2, F53H8.3, F59A1.13, H17B01.1, K08F9.1, K08H10.6, K09C4.1, K09C4.4, K09C4.5, M01F1.5, R09B5.11, T01B11.7, T08B1.1, T12B3.2, T27D12.1, Y37A1A.3, Y39B6A.41, Y39E4B.5, Y51A2D.4, Y51A2D.5, Y59A8B.21, Y61A9LA.1, Y71G12B.25, Y82E9BR.16, ZK637.1, ZK829.9

**(36) amino acid transporters**

B0303.11, B0454.6, C03A3.2, C44B7.6, C50D2.2, C55C2.5, F07C3.7, F10E7.9, F13H10.3, F21D12.3, F23F1.6, F27C8.1, F28F9.4, F52H2.2, F54D12.3, F59B2.2, H16O14.1, H32K16.1, K02A2.3, R02F2.8, R13A1.2, T04B8.5, T11F9.4, T13A10.10, T20G5.6, T27A1.5, Y18D10A.23, Y32F6A.4, Y37A1C.1, Y38H6C.17, Y43F4B.7, Y4C6B.2, Y51F10.4, Y53F4B.12, Y53H1C.1, Y59H11AR.4

**(20) domain:AA_permease (PF00324)** amino acid permease

B0303.11, B0454.6, C50D2.2, C55C2.5, F07C3.7, F10E7.9, F23F1.6, F27C8.1, F28F9.4, F52H2.2, F54D12.3, H16O14.1, K02A2.3, R13A1.2, T04B8.5, T11F9.4, T13A10.10, Y37A1C.1, Y53F4B.12, Y53H1C.1

**(16) domain:Aa_trans (PF01490)** amino acid transporter

C03A3.2, C44B7.6, F13H10.3, F21D12.3, F59B2.2, H32K16.1, R02F2.8, T20G5.6, T27A1.5, Y18D10A.23, Y32F6A.4, Y38H6C.17, Y43F4B.7, Y4C6B.2, Y51F10.4, Y59H11AR.4

**(20) domain:E1-E2_ATPase (PF00122)** proton ATPase

B0365.3, C01G12.8, C02E7.1, C09H5.2, C10C6.6, F02C9.3, F36H2.1, K07E3.7, K11D9.2, R05C11.3, T24H7.5, W08D2.5, W09C2.3, W09D10.2, Y105E8A.12, Y49E10.11, Y59H11AR.2, Y67D8C.10, Y76A2A.2, ZK256.1

**(11) domain:Cation_ATPase_N (SM00831)**

B0365.3, C01G12.8, C02E7.1, C09H5.2, K11D9.2, R05C11.3, W09C2.3, Y105E8A.12, Y59H11AR.2, Y67D8C.10, ZK256.1

**(10) domain:Cation_ATPase_C (PF00689)**

B0365.3, C01G12.8, C02E7.1, C09H5.2, K11D9.2, R05C11.3, W09C2.3, Y105E8A.12, Y67D8C.10, ZK256.1

**(15) domain:Mito_carr (PF00153)** mitochondrial carrier

C14C10.1, C16C10.1, C47E12.2, D1046.3, F01G4.6, F13G3.7, F25B4.7, F43G9.3, F49E8.5, K01H12.2, R07B7.10, R07E3.4, T01B11.4, T27E9.1, W02D3.6

**(14) domain:Zip (PF02535)** zinc transporter

C06G8.3, C14H10.1, C18A3.2, C30H6.2, F30B5.7, F31C3.4, F55F8.9, F59A3.4, H13N06.5, T01D3.5, T11F9.2, T28F3.3, Y54G9A.4, Y55F3BL.2

**(14) domain:SNF (PF00209)** sodium neurotransmitter symporter

C49C3.1, F55H12.1, F56F4.3, M01G5.5, T03F7.1, T13B5.1, T23G5.5, T25B6.7, W03G9.1, Y32F6A.2, Y46G5A.30, Y54E10BR.7, ZK1010.9, ZK829.10

**(12) domain:Cation_efflux (PF01545)** cation efflux family, thought to discard toxic divalent metal ions

C15B12.7, F41C6.7, F56C9.3, K07G5.5, PDB1.1, R02F11.3, T18D3.3, Y105E8A.3, Y39E4A.2, Y71H2AM.9, ZC395.3, ZK185.5

**(7) domain:Mtc (PF03820)** tricarboxylate carrier

AH6.2, C14F5.4, C41C4.10, C47D12.3, F37H8.4, T04F8.1, Y6E2A.9

**(6) domain:TPT (PF03151)** triphosphate transporter

C50F4.14, C52E12.3, T21B6.5, Y47G6A.7, Y73B6BL.31, Y73E7A.3

**(10) gene:vha-*** vacuolar (H+)-ATPases (added ZK637.8)

C26H9A.1, F35H10.4, F49C12.13, R10E11.2, R10E11.8, T01H3.1, VW02B12L.1, Y38F2AL.4, Y55H10A.1, ZK637.8

**(8) gene:sulp-*** sulfate permease (PF00916+PF01740)

C55B7.6, F14D12.5, F41D9.5, K12G11.1, K12G11.2, W01B11.2, W04G3.6, ZK287.2

**(6) gene:glt-*, domain:SDF (PF00375)** Na_CO2_symporter

C12D12.2, K08F4.4, R05G6.6, T22E5.2, W03G1.1, Y53C12A.2

**(233) GO: transporter_activity** (aquaporins, glr-*,nmr-* were moved to channels)

AH6.2, B0212.4, B0222.2, B0222.3, B0240.1, B0252.3, B0285.6, B0331.2, B0361.11, B0365.3, B0454.6, C01F1.2, C01G12.8, C02E7.1, C03B1.13, C03H5.2, C05E11.4, C05E11.5, C06G8.2, C06G8.3, C06H5.6, C12D12.2, C14F5.4, C14H10.1, C15B12.7, C17B7.5, C18A3.2, C18E9.2, C26H9A.1, C30H6.2, C32E8.8, C35A11.4, C41C4.10, C44C10.3, C46C2.2, C47A4.2, C47D12.3, C47E12.2, C47G2.3, C48A7.2, C48D1.3, C50D2.2, C51E3.6, C53B4.1, C55B7.6, C55C2.5, E04A4.5, F01G12.1, F07C3.7, F08F1.5, F08F3.3, F09G2.3, F11D5.7, F13B12.2, F14B8.3, F14D12.5, F14E5.1, F15B10.1, F16H11.3, F17C11.12, F21F8.11, F21G4.1, F21G4.2, F23F1.6, F23F12.13, F23F12.3, F25B4.7, F27C1.2, F27C8.1, F28F9.4, F29D11.1, F30B5.7, F31C3.4, F31E8.2, F31E8.4, F31F6.6, F32D8.6, F32H5.4, F35H10.4, F36H2.2, F37H8.4, F41C6.7, F41D9.5, F42G8.11, F43E2.4, F44C8.7, F44D12.9, F45E10.2, F47B8.10, F47E1.2, F47E1.4, F48E3.2, F49C12.13, F49C12.6, F49E11.3, F49E8.5, F52B5.1, F52F12.1, F52H2.2, F52H2.4, F53B1.8, F53H8.3, F54D12.3, F55F8.9, F56C9.3, F56F4.5, F57C12.4, F57C12.5, F58G6.3, F58G6.9, F59A1.13, F59A3.4, H13N06.5, H17B01.1, K01H12.2, K02E11.1, K02G10.5, K04E7.2, K05F1.6, K06H6.3, K07G5.5, K07H8.2, K08E5.2, K08F9.1, K08H10.6, K09A9.3, K09C4.1, K09C4.5, K09E9.1, K11D9.3, K11G12.3, K11G12.4, K12C11.3, K12C11.6, K12C11.7, K12G11.1, K12G11.2, M01F1.5, M02B1.1, M195.3, PDB1.1, R02F11.3, R03E9.3, R05C11.3, R07B7.10, R07E3.4, R08F11.6, R09B5.11, R09H10.4, R107.1, R10E11.2, R10E11.8, R11E3.2, T01B11.4, T01B11.7, T01D3.5, T01H3.1, T04F8.1, T07G12.2, T07G12.5, T08B1.1, T09B9.2, T10C6.6, T11F9.2, T11F9.4, T12B3.2, T13A10.10, T18D3.3, T22F7.1, T23G5.5, T25D3.4, T27E9.1, T28F3.3, VW02B12L.1, W01B11.2, W01C8.6, W02D3.6, W04C9.1, W04G3.6, W05H5.3, W08D2.4, W09C2.3, W09D6.6, Y105E8A.3, Y32F6B.1, Y37A1A.3, Y37E3.16, Y38F2AL.4, Y39B6A.41, Y39E4A.2, Y39E4B.5, Y43F8C.12, Y51A2D.18, Y51A2D.4, Y51A2D.5, Y53F4B.12, Y53H1C.1, Y54G9A.4, Y55F3BL.2, Y57G11C.15, Y57G11C.23, Y58A7A.1, Y59E9AL.4, Y61A9LA.1, Y66D12A.13, Y67D8C.10, Y69A2AR.4, Y6E2A.9, Y70G10A.3, Y71D11A.1, Y71H2AM.9, Y76A2A.2, Y82E9BR.16, Y82E9BR.3, ZC250.3, ZC395.3, ZK1053.6, ZK180.3, ZK185.2, ZK185.5, ZK287.2, ZK370.7, ZK455.8, ZK484.2, ZK563.2, ZK637.1, ZK637.8, ZK682.2, ZK809.4, ZK822.5, ZK829.9, ZK892.3, ZK896.9

**(46) from other groups, not grouped in any of above**

B0240.4, C05D10.3, C06E1.3, C06H2.4, C08D8.1, C15H9.1, C16C10.12, C17E4.9, C18C4.2, C24F3.1, C24F3.5, C26C6.9, C29H12.2, C43F9.6, C43G2.2, C48B4.4, C50E3.16, C53B4.6, F02E11.1, F23H12.2, F27E11.1, F27E11.2, F36G9.3, F37B4.7, F47G9.1, F52D10.1, F54E7.1, F55F3.3, F56F4.5, F57B10.5, F57F10.1, K08E4.6, M03F8.2, M153.2, T04G9.5, T23H2.1, Y111B2A.20, Y38F2AR.2, Y53G8B.4, Y54G2A.18, Y54G2A.4, Y55H10A.1, Y71F9AM.6, Y71G10AR.4, Y73B6BL.36, ZK6.6

**(8) from "no other domain", not grouped in any of above**

C42C1.16, F01G12.2, F36H1.10, F36H1.5, F36H1.9, F45E6.1, R02E12.6, Y71G10AR.1

**(493) transporters combined**

AH6.2, B0212.4, B0222.2, B0222.3, B0240.1, B0240.4, B0252.3, B0285.6, B0303.11, B0331.2, B0361.11, B0365.3, B0416.5, B0454.6, C01B4.7, C01B4.8, C01B4.9, C01F1.2, C01G12.8, C02C2.4, C02E7.1, C03A3.2, C03B1.13, C03H5.2, C05A9.1, C05D10.3, C05E11.4, C05E11.5, C05G5.1, C06E1.3, C06G8.2, C06G8.3, C06H2.4, C06H5.6, C08D8.1, C09D4.1, C09H5.2, C10C6.5, C10C6.6, C10E2.6, C12D12.2, C13C4.5, C13C4.6, C14A6.2, C14C10.1, C14F5.4, C14H10.1, C15B12.7, C15H9.1, C16C10.1, C16C10.12, C17B7.5, C17E4.9, C18A3.2, C18C4.2, C18D1.2, C18E9.2, C18H9.5, C24F3.1, C24F3.5, C25E10.5, C26C6.9, C26H9A.1, C27C12.4, C29H12.2, C30H6.2, C30H6.6, C32E8.8, C34G6.4, C35A11.4, C35A5.3, C38C10.2, C39E9.10, C39E9.7, C41C4.10, C42C1.16, C42C1.8, C43F9.6, C43G2.2, C44B7.6, C44B7.8, C44B7.9, C44C10.3, C46C11.2, C46C2.2, C47A10.1, C47A4.2, C47D12.3, C47E12.2, C47G2.3, C48A7.2, C48B4.4, C48D1.3, C49C3.1, C49F8.2, C50D2.2, C50E3.16, C50F4.14, C51E3.6, C52E12.3, C53B4.1, C53B4.3, C53B4.6, C54D1.1, C54G10.3, C55B7.6, C55C2.5, C56E6.1, C56E6.5, D1046.3, D1046.4, DH11.3, E03G2.2, E04A4.5, F01G12.1, F01G12.2, F01G4.6, F02C9.3, F02E11.1, F07C3.7, F08F1.5, F08F3.3, F09A5.1, F09G2.3, F10C2.7, F10D7.2, F10E7.9, F10G7.5, F11A5.9, F11D5.7, F12B6.1, F12B6.2, F13B12.2, F13G3.7, F13H10.3, F14B8.3, F14D12.5, F14D7.6, F14E5.1, F14F4.3, F15B10.1, F16H11.1, F16H11.3, F17C11.12, F19B6.4, F20B6.3, F21D12.3, F21F8.11, F21G4.1, F21G4.2, F22E10.1, F22E10.2, F22E10.3, F23F1.6, F23F12.13, F23F12.3, F23H12.2, F25B4.7, F25D1.2, F25G6.7, F27C1.2, F27C8.1, F27D9.2, F27E11.1, F27E11.2, F28F9.4, F29D11.1, F30B5.7, F31C3.4, F31E8.2, F31E8.4, F31F6.6, F32D8.6, F32H5.4, F35H10.4, F36G9.3, F36H1.10, F36H1.5, F36H1.9, F36H2.1, F36H2.2, F37B4.7, F37H8.4, F40F9.5, F41C3.2, F41C6.7, F41D9.5, F42E11.1, F42G8.11, F43E2.4, F43G9.3, F44C8.7, F44D12.9, F44E7.7, F45E10.2, F45E4.11, F45E6.1, F47B8.10, F47E1.2, F47E1.4, F47G9.1, F48E3.2, F49C12.13, F49C12.6, F49E11.3, F49E8.5, F52B5.1, F52D10.1, F52F12.1, F52H2.2, F52H2.4, F53B1.8, F53H8.3, F54D12.3, F54E7.1, F55A4.8, F55F3.3, F55F8.9, F55H12.1, F56A4.10, F56A4.11, F56A4.12, F56C9.3, F56F4.3, F56F4.5, F57A10.3, F57A8.7, F57B10.5, F57C12.4, F57C12.5, F57F10.1, F58G11.4, F58G6.3, F58G6.9, F59A1.13, F59A3.4, F59B2.2, F59F5.1, H11E01.2, H13N06.5, H16O14.1, H17B01.1, H32K16.1, K01H12.2, K02A2.3, K02E11.1, K02G10.5, K04E7.2, K05B2.5, K05F1.6, K06H6.3, K07E3.7, K07G5.5, K07H8.2, K08C7.1, K08E4.6, K08E5.2, K08E7.9, K08F4.4, K08F9.1, K08H10.6, K09A9.3, K09C4.1, K09C4.4, K09C4.5, K09E9.1, K10G9.1, K10H10.1, K11D9.2, K11D9.3, K11G12.3, K11G12.4, K11G9.5, K12C11.3, K12C11.6, K12C11.7, K12G11.1, K12G11.2, M01F1.5, M01G5.5, M02B1.1, M03B6.2, M03F8.2, M117.1, M153.2, M162.5, M195.3, PDB1.1, R02E12.6, R02F11.3, R02F2.8, R03E9.3, R05C11.3, R05G6.6, R07B7.10, R07E3.4, R08F11.6, R09B5.11, R09H10.4, R107.1, R10D12.1, R10E11.2, R10E11.8, R11E3.2, R13A1.2, R13A5.9, T01B11.4, T01B11.7, T01D3.5, T01H3.1, T01H3.3, T02B11.6, T02D1.5, T02G5.12, T03F7.1, T04B8.5, T04F8.1, T04G9.5, T04H1.1, T05A1.5, T07A5.3, T07G12.2, T07G12.5, T08B1.1, T09B9.2, T10C6.6, T10H9.5, T11F9.2, T11F9.4, T11G6.2, T11G6.3, T11G6.4, T12B3.2, T13A10.10, T13B5.1, T18D3.3, T19D12.10, T19D12.9, T20G5.6, T21B6.5, T21E8.1, T21E8.2, T21E8.3, T22E5.2, T22F3.10, T22F3.11, T22F3.7, T22F3.8, T22F7.1, T23G5.5, T23H2.1, T24H7.5, T25B6.7, T25D3.4, T26A5.1, T27A1.5, T27D12.1, T27E9.1, T28C12.1, T28C12.2, T28F3.3, T28F3.4, T28H11.8, VW02B12L.1, W01B11.2, W01B6.3, W01C8.6, W02D3.6, W03C9.6, W03G1.1, W03G9.1, W04C9.1, W04C9.6, W04G3.6, W05E10.1, W05H5.3, W08D2.4, W08D2.5, W09C2.3, W09D10.2, W09D6.6, Y105E8A.12, Y105E8A.3, Y111B2A.19, Y111B2A.20, Y15E3A.4, Y18D10A.23, Y19D10A.10, Y19D10A.11, Y19D10A.12, Y19D10A.4, Y19D10A.5, Y19D10A.8, Y32F6A.2, Y32F6A.4, Y32F6B.1, Y37A1A.3, Y37A1C.1, Y37E3.16, Y38F2AL.4, Y38F2AR.2, Y38H6C.17, Y39B6A.41, Y39D8C.1, Y39E4A.2, Y39E4B.5, Y42G9A.6, Y43F4B.7, Y43F8A.5, Y43F8C.12, Y46G5A.30, Y47D3A.11, Y47G6A.7, Y48G8AL.11, Y49E10.11, Y4C6B.2, Y4C6B.3, Y4C6B.4, Y4C6B.5, Y50E8A.16, Y51A2D.18, Y51A2D.4, Y51A2D.5, Y51B9A.6, Y51F10.4, Y53C10A.9, Y53C12A.2, Y53F4B.12, Y53G8AR.7, Y53G8B.4, Y53H1C.1, Y54E10BR.7, Y54G2A.18, Y54G2A.4, Y54G9A.4, Y55F3BL.2, Y55H10A.1, Y57G11C.15, Y57G11C.23, Y58A7A.1, Y59A8B.21, Y59E9AL.4, Y59H11AR.2, Y59H11AR.4, Y61A9LA.1, Y66D12A.13, Y67D8C.10, Y69A2AR.4, Y6E2A.9, Y70G10A.3, Y71D11A.1, Y71F9AM.6, Y71G10AR.1, Y71G10AR.4, Y71G12B.25, Y71H2AM.9, Y73B6BL.31, Y73B6BL.36, Y73E7A.3, Y74C10AR.3, Y75B8A.26, Y76A2A.2, Y82E9BR.16, Y82E9BR.3, ZC250.3, ZC395.3, ZC416.8, ZK1010.9, ZK1053.6, ZK180.3, ZK185.2, ZK185.5, ZK256.1, ZK287.2, ZK370.7, ZK455.7, ZK455.8, ZK484.2, ZK512.6, ZK54.1, ZK550.2, ZK563.1, ZK563.2, ZK6.6, ZK637.1, ZK637.8, ZK682.2, ZK809.4, ZK822.5, ZK829.10, ZK829.9, ZK892.3, ZK896.9

**(484) transporter combined, curated** (removed C01F1.2, C32E8.8, F29D11.1, F31E8.2, F42G8.11, R09H10.4, W08D2.4, Y71D11A.1, Y82E9BR.3)

AH6.2, B0212.4, B0222.2, B0222.3, B0240.1, B0240.4, B0252.3, B0285.6, B0303.11, B0331.2, B0361.11, B0365.3, B0416.5, B0454.6, C01B4.7, C01B4.8, C01B4.9, C01G12.8, C02C2.4, C02E7.1, C03A3.2, C03B1.13, C03H5.2, C05A9.1, C05D10.3, C05E11.4, C05E11.5, C05G5.1, C06E1.3, C06G8.2, C06G8.3, C06H2.4, C06H5.6, C08D8.1, C09D4.1, C09H5.2, C10C6.5, C10C6.6, C10E2.6, C12D12.2, C13C4.5, C13C4.6, C14A6.2, C14C10.1, C14F5.4, C14H10.1, C15B12.7, C15H9.1, C16C10.1, C16C10.12, C17B7.5, C17E4.9, C18A3.2, C18C4.2, C18D1.2, C18E9.2, C18H9.5, C24F3.1, C24F3.5, C25E10.5, C26C6.9, C26H9A.1, C27C12.4, C29H12.2, C30H6.2, C30H6.6, C34G6.4, C35A11.4, C35A5.3, C38C10.2, C39E9.10, C39E9.7, C41C4.10, C42C1.16, C42C1.8, C43F9.6, C43G2.2, C44B7.6, C44B7.8, C44B7.9, C44C10.3, C46C11.2, C46C2.2, C47A10.1, C47A4.2, C47D12.3, C47E12.2, C47G2.3, C48A7.2, C48B4.4, C48D1.3, C49C3.1, C49F8.2, C50D2.2, C50E3.16, C50F4.14, C51E3.6, C52E12.3, C53B4.1, C53B4.3, C53B4.6, C54D1.1, C54G10.3, C55B7.6, C55C2.5, C56E6.1, C56E6.5, D1046.3, D1046.4, DH11.3, E03G2.2, E04A4.5, F01G12.1, F01G12.2, F01G4.6, F02C9.3, F02E11.1, F07C3.7, F08F1.5, F08F3.3, F09A5.1, F09G2.3, F10C2.7, F10D7.2, F10E7.9, F10G7.5, F11A5.9, F11D5.7, F12B6.1, F12B6.2, F13B12.2, F13G3.7, F13H10.3, F14B8.3, F14D12.5, F14D7.6, F14E5.1, F14F4.3, F15B10.1, F16H11.1, F16H11.3, F17C11.12, F19B6.4, F20B6.3, F21D12.3, F21F8.11, F21G4.1, F21G4.2, F22E10.1, F22E10.2, F22E10.3, F23F1.6, F23F12.13, F23F12.3, F23H12.2, F25B4.7, F25D1.2, F25G6.7, F27C1.2, F27C8.1, F27D9.2, F27E11.1, F27E11.2, F28F9.4, F30B5.7, F31C3.4, F31E8.4, F31F6.6, F32D8.6, F32H5.4, F35H10.4, F36G9.3, F36H1.10, F36H1.5, F36H1.9, F36H2.1, F36H2.2, F37B4.7, F37H8.4, F40F9.5, F41C3.2, F41C6.7, F41D9.5, F42E11.1, F43E2.4, F43G9.3, F44C8.7, F44D12.9, F44E7.7, F45E10.2, F45E4.11, F45E6.1, F47B8.10, F47E1.2, F47E1.4, F47G9.1, F48E3.2, F49C12.13, F49C12.6, F49E11.3, F49E8.5, F52B5.1, F52D10.1, F52F12.1, F52H2.2, F52H2.4, F53B1.8, F53H8.3, F54D12.3, F54E7.1, F55A4.8, F55F3.3, F55F8.9, F55H12.1, F56A4.10, F56A4.11, F56A4.12, F56C9.3, F56F4.3, F56F4.5, F57A10.3, F57A8.7, F57B10.5, F57C12.4, F57C12.5, F57F10.1, F58G11.4, F58G6.3, F58G6.9, F59A1.13, F59A3.4, F59B2.2, F59F5.1, H11E01.2, H13N06.5, H16O14.1, H17B01.1, H32K16.1, K01H12.2, K02A2.3, K02E11.1, K02G10.5, K04E7.2, K05B2.5, K05F1.6, K06H6.3, K07E3.7, K07G5.5, K07H8.2, K08C7.1, K08E4.6, K08E5.2, K08E7.9, K08F4.4, K08F9.1, K08H10.6, K09A9.3, K09C4.1, K09C4.4, K09C4.5, K09E9.1, K10G9.1, K10H10.1, K11D9.2, K11D9.3, K11G12.3, K11G12.4, K11G9.5, K12C11.3, K12C11.6, K12C11.7, K12G11.1, K12G11.2, M01F1.5, M01G5.5, M02B1.1, M03B6.2, M03F8.2, M117.1, M153.2, M162.5, M195.3, PDB1.1, R02E12.6, R02F11.3, R02F2.8, R03E9.3, R05C11.3, R05G6.6, R07B7.10, R07E3.4, R08F11.6, R09B5.11, R107.1, R10D12.1, R10E11.2, R10E11.8, R11E3.2, R13A1.2, R13A5.9, T01B11.4, T01B11.7, T01D3.5, T01H3.1, T01H3.3, T02B11.6, T02D1.5, T02G5.12, T03F7.1, T04B8.5, T04F8.1, T04G9.5, T04H1.1, T05A1.5, T07A5.3, T07G12.2, T07G12.5, T08B1.1, T09B9.2, T10C6.6, T10H9.5, T11F9.2, T11F9.4, T11G6.2, T11G6.3, T11G6.4, T12B3.2, T13A10.10, T13B5.1, T18D3.3, T19D12.10, T19D12.9, T20G5.6, T21B6.5, T21E8.1, T21E8.2, T21E8.3, T22E5.2, T22F3.10, T22F3.11, T22F3.7, T22F3.8, T22F7.1, T23G5.5, T23H2.1, T24H7.5, T25B6.7, T25D3.4, T26A5.1, T27A1.5, T27D12.1, T27E9.1, T28C12.1, T28C12.2, T28F3.3, T28F3.4, T28H11.8, VW02B12L.1, W01B11.2, W01B6.3, W01C8.6, W02D3.6, W03C9.6, W03G1.1, W03G9.1, W04C9.1, W04C9.6, W04G3.6, W05E10.1, W05H5.3, W08D2.5, W09C2.3, W09D10.2, W09D6.6, Y105E8A.12, Y105E8A.3, Y111B2A.19, Y111B2A.20, Y15E3A.4, Y18D10A.23, Y19D10A.10, Y19D10A.11, Y19D10A.12, Y19D10A.4, Y19D10A.5, Y19D10A.8, Y32F6A.2, Y32F6A.4, Y32F6B.1, Y37A1A.3, Y37A1C.1, Y37E3.16, Y38F2AL.4, Y38F2AR.2, Y38H6C.17, Y39B6A.41, Y39D8C.1, Y39E4A.2, Y39E4B.5, Y42G9A.6, Y43F4B.7, Y43F8A.5, Y43F8C.12, Y46G5A.30, Y47D3A.11, Y47G6A.7, Y48G8AL.11, Y49E10.11, Y4C6B.2, Y4C6B.3, Y4C6B.4, Y4C6B.5, Y50E8A.16, Y51A2D.18, Y51A2D.4, Y51A2D.5, Y51B9A.6, Y51F10.4, Y53C10A.9, Y53C12A.2, Y53F4B.12, Y53G8AR.7, Y53G8B.4, Y53H1C.1, Y54E10BR.7, Y54G2A.18, Y54G2A.4, Y54G9A.4, Y55F3BL.2, Y55H10A.1, Y57G11C.15, Y57G11C.23, Y58A7A.1, Y59A8B.21, Y59E9AL.4, Y59H11AR.2, Y59H11AR.4, Y61A9LA.1, Y66D12A.13, Y67D8C.10, Y69A2AR.4, Y6E2A.9, Y70G10A.3, Y71F9AM.6, Y71G10AR.1, Y71G10AR.4, Y71G12B.25, Y71H2AM.9, Y73B6BL.31, Y73B6BL.36, Y73E7A.3, Y74C10AR.3, Y75B8A.26, Y76A2A.2, Y82E9BR.16, ZC250.3, ZC395.3, ZC416.8, ZK1010.9, ZK1053.6, ZK180.3, ZK185.2, ZK185.5, ZK256.1, ZK287.2, ZK370.7, ZK455.7, ZK455.8, ZK484.2, ZK512.6, ZK54.1, ZK550.2, ZK563.1, ZK563.2, ZK6.6, ZK637.1, ZK637.8, ZK682.2, ZK809.4, ZK822.5, ZK829.10, ZK829.9, ZK892.3, ZK896.9

**ENZYMES** (except signaling enzymes)

**(71) acyltransferases**

B0395.2, B0399.2, C02A12.8, C06B3.2, C08B11.4, C08F8.4, C17A2.5, C31A11.1, C31A11.5, C31A11.7, C42C1.7, C43F9.10, C48E7.8, C49D10.4, C49D10.8, C54G7.2, D2063.2, E03H4.7, F09B9.1, F11A5.8, F14B6.5, F14F3.3, F17B5.2, F28G4.5, F35E2.6, F36G9.12, F37C4.1, F37C4.2, F37C4.3, F39G3.6, F41D3.10, F41D3.2, F41D3.4, F41D3.5, F41E6.14, F47F6.3, F52F10.3, F52F10.4, F53C11.2, F56G4.1, F56H6.11, F56H6.12, H12I19.4, H12I19.5, H19N07.4, K09E10.1, K09E10.2, R02C2.3, R03H4.1, R03H4.5, R03H4.6, R155.1, T06C12.8, T07H6.2, T09E11.4, T09E11.5, T09E11.7, T14D7.2, T23G4.4, T26H2.7, T27E4.6, W03B1.7, W03B1.8, W06G6.1, W07A12.6, W07A12.7, Y39H10A.2, Y57G11C.17, Y67A10A.1, ZC101.3, ZK550.1

**(59) domain:acyltransferase (PF01757)**

B0399.2, C02A12.8, C06B3.2, C08B11.4, C17A2.5, C31A11.1, C31A11.5, C31A11.7, C42C1.7, C43F9.10, C48E7.8, C49D10.4, C49D10.8, D2063.2, E03H4.7, F09B9.1, F11A5.8, F14B6.5, F17B5.2, F28G4.5, F35E2.6, F36G9.12, F37C4.1, F37C4.2, F37C4.3, F39G3.6, F41D3.10, F41D3.2, F41D3.4, F41D3.5, F41E6.14, F47F6.3, F52F10.3, F52F10.4, F56G4.1, F56H6.11, F56H6.12, H12I19.4, H12I19.5, K09E10.1, K09E10.2, R02C2.3, R03H4.1, R03H4.5, R03H4.6, T06C12.8, T09E11.4, T09E11.5, T09E11.7, T14D7.2, T23G4.4, T26H2.7, W03B1.7, W03B1.8, W06G6.1, W07A12.6, W07A12.7, Y39H10A.2, Y67A10A.1

**(58) gene:oac-* (O-acyltransferase)**

B0399.2, C02A12.8, C06B3.2, C17A2.5, C31A11.1, C31A11.5, C31A11.7, C42C1.7, C43F9.10, C48E7.8, C49D10.4, C49D10.8, D2063.2, E03H4.7, F09B9.1, F11A5.8, F14B6.5, F17B5.2, F28G4.5, F35E2.6, F36G9.12, F37C4.1, F37C4.2, F37C4.3, F39G3.6, F41D3.10, F41D3.2, F41D3.4, F41D3.5, F41E6.14, F47F6.3, F52F10.3, F52F10.4, F53C11.2, F56G4.1, F56H6.11, F56H6.12, H12I19.4, H12I19.5, K09E10.1, K09E10.2, R02C2.3, R03H4.1, R03H4.5, T06C12.8, T09E11.4, T09E11.5, T09E11.7, T14D7.2, T23G4.4, T26H2.7, T27E4.6, W03B1.7, W03B1.8, W06G6.1, W07A12.6, Y39H10A.2, Y67A10A.1

**(10) domain:MBOAT (PF03062)** membrane-bound o-acyltransferase

B0395.2, C08F8.4, C54G7.2, F14F3.3, H19N07.4, R155.1, T07H6.2, Y57G11C.17, ZC101.3, ZK550.1

**(67) domain:UDP_gluc_trans (PF00201)** UDP-glucoronosyl and UDP-glucosyl transferase

AC3.2, AC3.7, AC3.8, B0310.5, C03A7.11, C04F5.7, C07G3.9, C08B6.1, C08F11.8, C10H11.3, C10H11.4, C10H11.5, C10H11.6, C13D9.9, C17G1.3, C18C4.3, C23G10.6, C32C4.7, C33A12.6, C35A5.2, C44H9.1, C49A9.8, F01D4.1, F01D4.2, F01E11.1, F08G5.5, F09G2.6, F10D2.11, F10D2.12, F10D2.2, F10D2.5, F10D2.6, F10D2.7, F29F11.2, F31F4.7, F35H8.6, F39G3.1, F47C10.6, F54C1.1, F56B3.7, H23N18.1, H23N18.2, H23N18.3, K04A8.10, K08B4.3, K08B4.4, M88.1, R04B5.9, T01G5.2, T03D3.1, T04H1.7, T04H1.8, T07C5.1, T19H12.1, T19H12.10, T19H12.11, T19H12.9, T25B9.7, Y37E11AR.5, Y39G10AR.6, Y49C4A.8, ZC443.5, ZC443.6, ZC455.3, ZC455.4, ZC455.5, ZC455.6

**(50) domain:AAA (SM00382)** AAA ATPase

C05A9.1, C05D10.3, C10C6.5, C16C10.12, C30H6.6, C34G6.4, C44B7.8, C44B7.9, C47A10.1, C48B4.4, C54D1.1, C54G10.3, DH11.3, E03G2.2, F12B6.1, F14F4.3, F19B6.4, F20B6.3, F21G4.2, F22E10.1, F22E10.2, F22E10.3, F42E11.1, F43E2.4, F57A10.3, F57C12.4, F57C12.5, K04D7.2, K08E7.9, M03C11.5, T02D1.5, T21E8.1, T21E8.2, T21E8.3, T26A5.1, W04C9.1, W09D6.6, Y37A1B.13, Y38F2AR.7, Y39D8C.1, Y42G9A.6, Y43F8C.12, Y47D3A.11, Y48G8AL.11, Y50E8A.16, Y53C10A.9, Y74C10AR.3, Y75B8A.26, ZK455.7, ZK484.2

**(49) peptidases** (F21F8.2, F45E4.7 from no other domain)

AC3.5, C04A11.4, C33H5.11, C34B2.10, C34H3.1, C36B1.12, C48B4.2, C53D5.5, DY3.7, F12A10.4, F18A12.5, F18A12.8, F21F8.2, F26F4.3, F26G1.6, F27D9.7, F45E4.7, F49B2.6, F59A3.1, H14N18.4, K02E7.10, K03B8.2, K03B8.5, K04F10.4, K10C2.3, R07E3.1, R09F10.1, T03D8.6, T05E11.5, T06D4.4, T07F10.1, T18H9.2, T19H12.6, T23F1.7, Y116A8C.14, Y116A8C.16, Y37D8A.10, Y37D8A.13, Y40D12A.2, Y42A5A.1, Y54E10A.14, Y54E10BR.5, Y67D8C.9, Y71H2AM.25, Y71H2AR.2, Y7A9A.1, Y97E10AR.2, ZK154.7, ZK970.1

**(20) metallopeptidases (PF01433, PF01421, PF01431, PF00246, SM00235, SM00631)** (added ZK154.7)

C04A11.4, C34H3.1, DY3.7, F12A10.4, F18A12.5, F18A12.8, F26G1.6, F27D9.7, F59A3.1, K03B8.2, K03B8.5, T06D4.4, Y37D8A.13, ZK154.7, ZK970.1, AC3.5, F49B2.6, T07F10.1, Y42A5A.1, Y67D8C.9

**(9) serine peptidases (PF01694, PF00082, PF00326, PF00450, PF00717)**

C48B4.2, F26F4.3, Y116A8C.14, Y116A8C.16, Y54E10A.14, K04F10.4, T23F1.7, Y40D12A.2, Y54E10BR.5

**(6) domain:G_glu_transpept (PF01019)** gamma-glutamyl transpeptidase

C53D5.5, H14N18.4, T03D8.6, T19H12.6, Y7A9A.1, Y97E10AR.2

**(5) signal peptidases (PF04258, PF06645, SM00730, PF06703)**

C33H5.11, C34B2.10, C36B1.12, T05E11.5, Y37D8A.10

**(5) domain:Pept_C1 (SM00645)** papain family cysteine protease

K02E7.10, R07E3.1, R09F10.1, Y71H2AM.25, Y71H2AR.2

**(2) domain:Asp (PF00026)** aspartate proteases

K10C2.3, T18H9.2

**(19) domain:carbesterase (PF00135)** carboxylesterase

C01B10.10, C01B10.4, C17H12.4, C23H4.2, C23H4.3, C23H4.4, C23H4.7, C40C9.5, C42D4.2, E01G6.3, F55D10.3, R173.3, T02B5.1, T02B5.3, T07H6.1, T22D1.11, ZC376.1, ZC376.2, ZC376.3

**(19) domain:hydrolase (PF00702)** haloacid dehalogenase-like hydrolase

B0365.3, C01G12.8, C02E7.1, C09H5.2, C10C6.6, F02C9.3, F36H2.1, K11D9.2, R05C11.3, T24H7.5, W08D2.5, W09C2.3, W09D10.2, Y105E8A.12, Y49E10.11, Y59H11AR.2, Y67D8C.10, Y76A2A.2, ZK256.1

**(18) domain:chit (SM00636)** O-Glycosyl hydrolases, belong to chitinase II group

C08H9.10, C08H9.11, C08H9.12, C08H9.13, C08H9.4, C08H9.7, F15A4.8, M01B2.6, R09D1.1, R09D1.10, R09D1.11, R09D1.2, R09D1.3, R09D1.5, R09D1.6, T19H5.1, T19H5.2, ZK938.6

**(17) glycosyl transferases**

B0361.8, C14A4.3, C18G1.8, D2085.6, EGAP9.2, F11A5.5, F44F4.6, T10B10.8, T15D6.2, T26A5.4, T26E4.4, T27F7.3, Y45F10D.3, Y46H3A.6, Y5H2B.1, Y60A3A.14, ZC513.5

**(9) domain:glyco_transf_* (PF01501, PF01531, PF03901)**

C14A4.3, C18G1.8, EGAP9.2, F11A5.5, T10B10.8, T26E4.4, T27F7.3, Y5H2B.1, ZC513.5

**(6) domain: glycos_transf_* (PF00534, PF00535, PF00953)**

B0361.8, D2085.6, T26A5.4, Y45F10D.3, Y46H3A.6, Y60A3A.14

**(4) gene: gly-***

F44F4.6, T15D6.2, Y45F10D.3, Y46H3A.6

**(14) domain:PlsC (SM00563)** phosphate acyltransferase

C01C10.3, F08G5.2, F28B3.9, F44B9.5, F49H12.6, F55A11.5, F59F4.4, K07B1.5, M79.2, R07E3.5, T05H4.1, T06E8.1, Y38C1AA.1, ZK40.1

**(12) domain:cytochrome_P450 (PF00067)** (added T13C5.1)

B0304.3, F14H3.10, F28G4.1, R04D3.1, T09H2.1, T10B9.1, T10B9.10, T10B9.3, T10B9.5, T13C5.1, Y17D7A.4, Y80D3A.5

**(10) domain:DH_SDR (PF00106)** short-chain dehydrogenase/reductase

C04F6.5, C10F3.2, C15H11.4, C56G2.6, F27D9.6, F55A12.4, F55E10.6, F56D1.5, T11F9.11, ZK816.5

**(9) domain:ELO (PF01151)** fatty acid chain elongation enzyme

C40H1.4, D2024.3, F11E6.5, F41H10.7, F41H10.8, F56H11.3, F56H11.4, Y47D3A.30, Y53F4B.2

**(9) domain:FA_desaturase (PF00487)** fatty acid desaturase

F10D2.9, F33D4.4, T13F2.1, VZK822L.1, W02A2.1, W06D12.3, W08D2.4, Y54E5A.1, Y67H2A.8

**(8) domain:Branch (PF02485)** core-2/I-Branching enzyme

F30A10.4, F44F4.6, H41C03.3, R07B7.6, T09E11.6, T15D6.2, T28F3.9, Y51H4A.25

**(7) domain:B561 (SM00665)** Cytochrome b-561/ferric reductase transmembrane domain

C05D12.1, C13B4.1, F39G3.4, F39G3.5, F55H2.5, M03A1.3, M03A1.8

**(6) domain:Glyco_hydro_18/38/47/63(PF00704, PF01074, PF01532, PF03200)** glycosyl hydrolases

F13H10.4, F48C1.1, K08F9.3, R03E9.2, T03G11.4, T19H5.3

**(6) domain:His_Phos_2 (PF00328)** histidine phosphatase superfamily

B0361.7, EGAP2.3, F13D11.1, F14E5.3, ZK563.6, ZK792.1

**(5) domain:Steroid_dh (PF02544)** 3-oxo-5-alpha-steroid 4-dehydrogenase

B0024.13, C15F1.6, F19H6.4, F42F12.3, ZK1251.3

**(5) domain:FA_hydroxylase (PF04116)** fatty acid hydroxylase

BE10.2, C25A1.5, F35C8.5, F49E12.10, F49E12.9

**(5) domain:Galactosyl_T (PF01762)** galactosyltransferase

C02H6.1, T15D6.5, T22B11.2, Y39E4B.9, ZK678.8

**(5) domain:acidPPc (SM00014)** acid phosphatase

F13E6.5, F53C3.13, T06D8.3, T13C5.6, T28D9.3

**(5) domain:lipases (PF00657, PF01764)**

F36A2.9, R07B7.8, R07B7.9, T19D7.7, F42G9.6

**(10) gene: MTCE-* mitochondrially-encoded**, all involved in energy-transfer chain enzymatic activity

MTCE.11, MTCE.12, MTCE.21, MTCE.23, MTCE.25, MTCE.26, MTCE.31, MTCE.34, MTCE.35, MTCE.4

**(88) GO:catalytic_activity**

B0365.3, C01B10.9, C01G12.8, C02E7.1, C04F6.5, C08H9.10, C08H9.11, C08H9.12, C08H9.13, C08H9.4, C08H9.7, C09H5.2, C10C6.6, C10F3.2, C15H11.4, C15H9.1, C27A12.9, C27A7.1, C38C6.2, C46F4.2, C52B9.9, C56G2.6, D1009.1, E04F6.4, F02C9.3, F09G2.8, F13E6.5, F13H10.5, F15A4.8, F22F7.1, F26D10.9, F27D9.6, F28D1.9, F36H2.1, F42G8.6, F52H2.6, F53C3.13, F53F4.5, F55A12.4, F55E10.6, F56A11.5, F56D1.5, K02F3.6, K08C7.2, K08C7.5, K08F9.3, K11D9.2, M01B2.6, R05C11.3, R09B5.6, R09D1.1, R09D1.10, R09D1.11, R09D1.2, R09D1.3, R09D1.5, R09D1.6, T06D8.3, T08D10.2, T11F9.11, T13C5.6, T19H5.1, T19H5.2, T19H5.3, T22G5.5, T24H7.5, T28D9.3, T28F3.5, VF36H2L.1, W08D2.5, W09C2.3, W09D10.2, W10C8.5, Y105E8A.12, Y49E10.11, Y54E10BR.1, Y59H11AR.2, Y66H1B.4, Y67D8C.10, Y6B3B.11, Y6B3B.5, Y76A2A.2, Y7A5A.1, ZC190.1, ZC8.1, ZK256.1, ZK816.5, ZK938.6

**(588) GO:”ase_activity”** (There is no term like "ase_activity" but I can search with this worm in Gexplore.)

AC3.2, AC3.5, AC3.7, AC3.8, AH6.1, B0024.13, B0024.6, B0198.3, B0218.1, B0218.2, B0240.3, B0244.2, B0252.1, B0304.3, B0310.5, B0334.5, B0361.7, B0365.3, B0399.2, B0491.1, B0495.7, B0511.13, BE10.2, C01C10.3, C01G12.8, C01G6.8, C02A12.8, C02C2.5, C02E7.1, C02H6.1, C03A7.11, C03C10.3, C04A11.4, C04F12.10, C04F5.7, C04F6.5, C04H5.3, C05A9.1, C05D10.3, C05D2.1, C06A12.4, C06B3.2, C07G3.9, C08B11.4, C08B11.8, C08B6.1, C08F11.8, C08H9.10, C08H9.11, C08H9.12, C08H9.13, C08H9.3, C08H9.4, C08H9.5, C08H9.7, C09F9.2, C09H5.2, C10C6.5, C10C6.6, C10F3.2, C10F3.3, C10H11.3, C10H11.4, C10H11.5, C10H11.6, C12D8.5, C13B4.1, C13B9.4, C13D9.9, C14A4.3, C15F1.6, C15H11.4, C15H9.1, C16B8.1, C16C10.12, C16D9.2, C17A2.5, C17E4.9, C17F4.6, C17G1.3, C18A11.5, C18C4.2, C18C4.3, C18G1.8, C18H2.4, C23G10.6, C23H4.4, C24F3.5, C24G6.2, C25A1.5, C25F6.4, C30H6.6, C31A11.1, C31A11.5, C31A11.7, C31E10.8, C32C4.7, C32D5.2, C33A12.6, C33H5.11, C33H5.14, C33H5.18, C34B2.10, C34G6.4, C34H3.1, C35A5.2, C35C5.11, C35C5.2, C36B1.12, C42C1.7, C43F9.10, C43F9.6, C44B7.11, C44B7.8, C44B7.9, C44F1.5, C44H9.1, C47A10.1, C48B4.2, C48B4.4, C48E7.8, C49A9.8, C49D10.4, C49D10.8, C49F5.5, C49H3.1, C50H2.2, C52B11.3, C52B9.9, C53D5.5, C54D1.1, C54G10.3, C56G2.6, D1022.1, D1073.1, D2063.2, DH11.3, DY3.7, E01H11.1, E02D9.1, E03G2.2, E03H4.7, EGAP2.3, EGAP9.2, F01D4.1, F01D4.2, F01E11.1, F01G4.5, F02C9.3, F02E11.1, F02E9.7, F08B1.2, F08F1.1, F08G5.2, F08G5.5, F09A5.2, F09B12.6, F09B9.1, F09G2.1, F09G2.6, F10D2.11, F10D2.12, F10D2.2, F10D2.5, F10D2.6, F10D2.7, F10D2.9, F11A5.5, F11A5.8, F11C7.4, F11D5.3, F11E6.8, F12A10.4, F12B6.1, F13D11.1, F13H10.4, F14B6.5, F14E5.3, F14F3.3, F14F4.3, F14H3.10, F15A2.2, F15A4.8, F15A8.5, F17B5.2, F17C8.1, F18A12.5, F18A12.8, F18F11.5, F18H3.5, F19B6.4, F19H6.4, F20B6.3, F21F3.3, F21G4.2, F21H7.9, F22E10.1, F22E10.2, F22E10.3, F22E10.5, F22E5.3, F23H11.9, F23H12.6, F26E4.6, F26F4.3, F26G1.6, F27D9.6, F27D9.7, F28B3.9, F28G4.1, F28G4.5, F29C4.1, F29D11.1, F29F11.2, F30A10.4, F31F4.7, F33D4.4, F34D6.4, F35C8.5, F35E2.6, F35H8.6, F36A2.9, F36G9.12, F36H1.2, F36H2.1, F37C4.1, F37C4.2, F37C4.3, F39G3.1, F39G3.6, F40A3.5, F40B5.2, F41A4.1, F41D3.10, F41D3.2, F41D3.4, F41D3.5, F41E6.14, F42E11.1, F42F12.3, F42G9.6, F43E2.4, F44B9.5, F44D12.2, F44F4.6, F44G4.8, F45H10.1, F46C3.1, F47B7.2, F47C10.6, F47F6.3, F48A11.1, F48C1.1, F49B2.6, F49E12.10, F49E12.9, F49H12.6, F52E1.4, F52F10.3, F52F10.4, F52H2.6, F53B7.5, F53F4.5, F53G12.3, F54C1.1, F54D8.2, F54F12.1, F54F7.5, F55A11.5, F55A12.4, F55E10.6, F55F3.3, F56B3.7, F56C11.1, F56D1.5, F56G4.1, F56H6.11, F56H6.12, F57A10.3, F57B9.4, F57C12.4, F57C12.5, F57H12.4, F58A3.2, F59A1.10, F59A3.1, F59F3.1, F59F3.5, F59F4.4, F59F5.3, H06I04.5, H12I19.4, H12I19.5, H13N06.6, H14N18.4, H19N07.4, H23N18.1, H23N18.2, H23N18.3, H41C03.3, K02E7.10, K03B8.2, K03B8.5, K04A8.10, K04D7.2, K04D7.4, K04F1.1, K04F10.4, K07B1.4, K07B1.5, K07E3.7, K07F5.6, K08B4.3, K08B4.4, K08C7.2, K08C7.5, K08E7.9, K08F9.3, K09A9.6, K09E10.1, K09E10.2, K09E4.2, K09F6.3, K10C2.3, K10F12.3, K11D9.2, M01B2.6, M01D7.2, M01E11.1, M02F4.8, M03A1.1, M03C11.5, M05B5.1, M176.6, M176.7, M79.2, M88.1, R01E6.1, R02C2.3, R03D7.8, R03H4.1, R03H4.5, R03H4.6, R04B5.9, R04D3.1, R05C11.3, R06B10.1, R07B7.4, R07B7.5, R07B7.6, R07B7.8, R07B7.9, R07E3.1, R07E3.5, R08F11.1, R09B5.6, R09D1.1, R09D1.10, R09D1.11, R09D1.12, R09D1.2, R09D1.3, R09D1.5, R09D1.6, R09F10.1, R134.1, R134.2, R155.2, R155.3, R173.1, R57.1, T01A4.1, T01C2.1, T01G5.2, T02B5.1, T02B5.3, T02D1.5, T03D3.1, T03D8.5, T03D8.6, T03G11.4, T04H1.7, T04H1.8, T05E11.5, T05G5.5, T05H4.1, T05H4.13, T05H4.4, T05H4.5, T06C12.8, T06D4.4, T06E8.1, T07C4.7, T07C5.1, T07F10.1, T07H6.1, T08D10.2, T09A5.11, T09B4.1, T09E11.4, T09E11.5, T09E11.6, T09E11.7, T09H2.1, T10B10.8, T10B9.1, T10B9.10, T10B9.3, T10B9.5, T10H9.2, T10H9.5, T11F9.11, T12A2.2, T12B3.3, T12G3.4, T13C5.1, T13F2.1, T14D7.2, T14E8.1, T15D6.2, T15D6.5, T17A3.1, T18H9.2, T19B10.8, T19D7.7, T19H12.1, T19H12.10, T19H12.11, T19H12.6, T19H12.9, T19H5.1, T19H5.2, T19H5.3, T20D3.8, T21E8.1, T21E8.2, T21E8.3, T22B11.2, T22D1.4, T22F7.1, T22G5.5, T23F1.7, T23G4.4, T23G7.2, T24D1.4, T24H7.5, T25B9.7, T25G3.2, T26A5.1, T26C12.4, T26H2.7, T27F7.3, T28F3.5, T28F3.9, VZK822L.1, W01A11.2, W02A2.1, W02F12.2, W03B1.7, W03B1.8, W03F11.2, W03F11.4, W03F9.4, W04C9.1, W05H12.1, W06D12.3, W06G6.1, W07A12.6, W07A12.7, W08D2.4, W08D2.5, W09C2.3, W09D10.2, W09D6.6, W10C8.5, Y105C5B.2, Y105E8A.12, Y110A2AL.12, Y113G7C.1, Y116A8C.463, Y17D7A.4, Y22D7AR.7, Y37D8A.10, Y37D8A.13, Y37E11AR.5, Y38C1AA.1, Y38F2AR.7, Y38H6C.16, Y38H6C.20, Y39B6A.18, Y39B6A.30, Y39D8C.1, Y39E4B.9, Y39G10AR.6, Y39H10A.2, Y40D12A.2, Y41E3.3, Y42A5A.1, Y42G9A.6, Y43F8A.3, Y43F8C.12, Y46G5A.17, Y46G5A.2, Y46G5A.5, Y47D3A.11, Y47G6A.7, Y48G1C.5, Y48G8AL.11, Y48G9A.10, Y49A3A.1, Y49C4A.8, Y49E10.11, Y50E8A.16, Y51H4A.25, Y53C10A.9, Y53C12A.1, Y53G8B.2, Y54E10BR.1, Y54E10BR.5, Y54E2A.12, Y54E5A.1, Y54G2A.2, Y55D5A.5, Y55H10A.1, Y56A3A.2, Y56A3A.32, Y56A3A.36, Y59A8B.21, Y59H11AR.2, Y5H2B.1, Y61A9LA.1, Y66H1B.4, Y67A10A.1, Y67D8C.10, Y67D8C.9, Y67H2A.8, Y69A2AR.19, Y69E1A.3, Y6B3B.11, Y6B3B.5, Y71H2AM.25, Y71H2AR.2, Y73B6BL.7, Y74C10AR.3, Y75B8A.26, Y76A2A.2, Y7A5A.1, Y7A9A.1, Y80D3A.5, Y80D3A.8, Y87G2A.14, Y94H6A.5, Y97E10AR.2, ZC155.4, ZC239.7, ZC412.2, ZC416.8, ZC443.5, ZC443.6, ZC455.3, ZC455.4, ZC455.5, ZC455.6, ZC513.5, ZC8.1, ZK1067.1, ZK1248.14, ZK1251.3, ZK256.1, ZK370.4, ZK40.1, ZK455.2, ZK455.7, ZK484.2, ZK563.6, ZK675.3, ZK678.8, ZK783.1, ZK792.1, ZK792.7, ZK816.5, ZK858.1, ZK896.8, ZK938.5, ZK938.6, ZK970.1, ZK970.5, ZK970.6

**(12) GO:”ase and not ase_activity”** (manually curated; excluded "based", "polymerase", transporters, etc)

E04A4.5, F17C11.7, F23H12.2, F33D4.2, K03A1.2, K11C4.5, T02C5.5, T21B6.1, W01A8.4, Y82E9BR.3, ZK637.8, ZK938.7

**(52) GO:oxidoreductase_activity**

B0024.13, BE10.2, C02C2.5, C03C10.3, C04F6.5, C10F3.2, C12D8.5, C15F1.6, C15H11.4, C15H9.1, C25A1.5, C56G2.6, F10D2.9, F19H6.4, F27D9.6, F33D4.4, F35C8.5, F42F12.3, F45H10.1, F49E12.10, F49E12.9, F52H2.6, F53B7.5, F53F4.5, F53G12.3, F55A12.4, F55E10.6, F56C11.1, F56D1.5, M01D7.2, M02F4.8, R07B7.4, R07B7.5, R09B5.6, T05H4.13, T05H4.4, T05H4.5, T08D10.2, T11F9.11, T13F2.1, T22F7.1, VZK822L.1, W02A2.1, W06D12.3, W08D2.4, Y47G6A.7, Y54E5A.1, Y56A3A.32, Y67H2A.8, Y7A5A.1, ZK1251.3, ZK816.5

**(213) GO:transferase_activity**

AC3.2, AC3.7, AC3.8, B0310.5, B0334.5, B0399.2, B0491.1, C01C10.3, C01G6.8, C02A12.8, C02H6.1, C03A7.11, C04F5.7, C06B3.2, C07G3.9, C08B11.4, C08B11.8, C08B6.1, C08F11.8, C08H9.3, C10H11.3, C10H11.4, C10H11.5, C10H11.6, C13D9.9, C14A4.3, C17A2.5, C17G1.3, C18C4.3, C18G1.8, C23G10.6, C31A11.1, C31A11.5, C31A11.7, C32C4.7, C33A12.6, C33H5.18, C35A5.2, C42C1.7, C43F9.10, C44H9.1, C48E7.8, C49A9.8, C49D10.4, C49D10.8, C49F5.5, C50H2.2, C53D5.5, D2063.2, E03H4.7, EGAP9.2, F01D4.1, F01D4.2, F01E11.1, F01G4.5, F08G5.2, F08G5.5, F09B9.1, F09G2.6, F10D2.11, F10D2.12, F10D2.2, F10D2.5, F10D2.6, F10D2.7, F11A5.5, F11A5.8, F14B6.5, F14F3.3, F17B5.2, F21F3.3, F22E10.5, F23H11.9, F28B3.9, F28G4.5, F29D11.1, F29F11.2, F30A10.4, F31F4.7, F34D6.4, F35E2.6, F35H8.6, F36G9.12, F37C4.1, F37C4.2, F37C4.3, F39G3.1, F39G3.6, F41D3.10, F41D3.2, F41D3.4, F41D3.5, F41E6.14, F44B9.5, F44F4.6, F47C10.6, F47F6.3, F48A11.1, F49H12.6, F52F10.3, F52F10.4, F54C1.1, F55A11.5, F56B3.7, F56G4.1, F56H6.11, F56H6.12, F57B9.4, F59A1.10, F59F4.4, H12I19.4, H12I19.5, H14N18.4, H23N18.1, H23N18.2, H23N18.3, H41C03.3, K04A8.10, K04F1.1, K07B1.4, K07B1.5, K08B4.3, K08B4.4, K09E10.1, K09E10.2, K09E4.2, M01E11.1, M79.2, M88.1, R02C2.3, R03H4.1, R03H4.5, R03H4.6, R04B5.9, R07B7.6, R07E3.5, T01G5.2, T03D3.1, T03D8.6, T04H1.7, T04H1.8, T05G5.5, T05H4.1, T06C12.8, T06E8.1, T07C5.1, T09A5.11, T09B4.1, T09E11.4, T09E11.5, T09E11.6, T09E11.7, T10B10.8, T12A2.2, T14D7.2, T15D6.2, T15D6.5, T19H12.1, T19H12.10, T19H12.11, T19H12.6, T19H12.9, T20D3.8, T22B11.2, T22D1.4, T22G5.5, T23G4.4, T24D1.4, T25B9.7, T25G3.2, T26H2.7, T27F7.3, T28F3.9, W01A11.2, W03B1.7, W03B1.8, W03F9.4, W06G6.1, W07A12.6, W07A12.7, W10C8.5, Y110A2AL.12, Y37E11AR.5, Y38C1AA.1, Y39E4B.9, Y39G10AR.6, Y39H10A.2, Y46G5A.17, Y46G5A.2, Y46G5A.5, Y48G9A.10, Y49A3A.1, Y49C4A.8, Y51H4A.25, Y53G8B.2, Y54E10BR.1, Y56A3A.36, Y59A8B.21, Y5H2B.1, Y67A10A.1, Y7A9A.1, Y97E10AR.2, ZC416.8, ZC443.5, ZC443.6, ZC455.3, ZC455.4, ZC455.5, ZC455.6, ZC513.5, ZK40.1, ZK678.8, ZK858.1

**(64) GO:hydrolase_activity**

B0365.3, B0511.13, C01G12.8, C02E7.1, C08H9.10, C08H9.11, C08H9.12, C08H9.13, C08H9.4, C08H9.7, C09H5.2, C10C6.6, C33H5.14, F02C9.3, F02E9.7, F15A4.8, F36A2.9, F36H2.1, F40B5.2, F48C1.1, K07E3.7, K08F9.3, K10F12.3, K11D9.2, M01B2.6, R03D7.8, R05C11.3, R07B7.8, R07B7.9, R09D1.1, R09D1.10, R09D1.11, R09D1.2, R09D1.3, R09D1.5, R09D1.6, T02B5.1, T02B5.3, T07H6.1, T12B3.3, T19B10.8, T19D7.7, T19H5.1, T19H5.2, T19H5.3, T23G7.2, T24H7.5, W02F12.2, W08D2.5, W09C2.3, W09D10.2, Y105E8A.12, Y43F8A.3, Y49E10.11, Y54E10BR.1, Y59H11AR.2, Y67D8C.10, Y76A2A.2, Y87G2A.14, Y94H6A.5, ZC155.4, ZK256.1, ZK792.7, ZK938.6

**(31) GO:lyase_activity**

AH6.1, B0024.6, B0240.3, C04H5.3, C06A12.4, C10F3.3, C17F4.6, C44F1.5, C49H3.1, F08B1.2, F17C8.1, F21H7.9, F22E5.3, F23H12.6, F52E1.4, R01E6.1, R134.1, R134.2, T01A4.1, T01C2.1, T03D8.5, T26C12.4, W03F11.2, Y105C5B.2, Y66H1B.4, ZC239.7, ZC412.2, ZK455.2, ZK896.8, ZK970.5, ZK970.6

**(1) GO:isomerase_activity**

Y38H6C.16

**(8) GO:ligase_activity**

B0218.1, B0218.2, C18C4.3, C52B9.9, D1022.1, F11C7.4, F36H1.2, T28F3.5

**(17) from other groups, not grouped in any of above**

B0250.9, C25H3.9, C54G4.8, F09B12.3, F28D1.11, F33A8.5, F37A4.1, F46E10.9, F48F5.5, K03A1.2, R01B10.4, T14G10.7, T26A8.2, T27E9.5, ZC506.3, ZK154.7, ZK945.1

**(21) from "no other domain", not grouped in any of above**

B0035.18, B0511.2, C01B10.7, C27D8.4, C49D10.7, C50D2.9, F20B4.6, F28A10.7, F28C6.4, F33D11.9, F35C11.5, F53H8.4, F59G1.1, H06H21.10, H21P03.3, M01A10.3, R13D11.4, T06C12.10, T07D1.3, T08H10.4, Y22D7AL.8, Y71H2AM.4

**(724) ENZYMES combined**

AC3.2, AC3.5, AC3.7, AC3.8, AH6.1, B0024.13, B0024.6, B0035.18, B0198.3, B0218.1, B0218.2, B0240.3, B0244.2, B0250.9, B0252.1, B0304.3, B0310.5, B0334.5, B0361.7, B0361.8, B0365.3, B0395.2, B0399.2, B0491.1, B0495.7, B0511.13, B0511.2, BE10.2, C01B10.10, C01B10.4, C01B10.7, C01B10.9, C01C10.3, C01G12.8, C01G6.8, C02A12.8, C02C2.5, C02E7.1, C02H6.1, C03A7.11, C03C10.3, C04A11.4, C04F12.10, C04F5.7, C04F6.5, C04H5.3, C05A9.1, C05D10.3, C05D12.1, C05D2.1, C06A12.4, C06B3.2, C07G3.9, C08B11.4, C08B11.8, C08B6.1, C08F11.8, C08F8.4, C08H9.10, C08H9.11, C08H9.12, C08H9.13, C08H9.3, C08H9.4, C08H9.5, C08H9.7, C09F9.2, C09H5.2, C10C6.5, C10C6.6, C10F3.2, C10F3.3, C10H11.3, C10H11.4, C10H11.5, C10H11.6, C12D8.5, C13B4.1, C13B9.4, C13D9.9, C14A4.3, C15F1.6, C15H11.4, C15H9.1, C16B8.1, C16C10.12, C16D9.2, C17A2.5, C17E4.9, C17F4.6, C17G1.3, C17H12.4, C18A11.5, C18C4.2, C18C4.3, C18G1.8, C18H2.4, C23G10.6, C23H4.2, C23H4.3, C23H4.4, C23H4.7, C24F3.5, C24G6.2, C25A1.5, C25F6.4, C25H3.9, C27A12.9, C27A7.1, C27D8.4, C30H6.6, C31A11.1, C31A11.5, C31A11.7, C31E10.8, C32C4.7, C32D5.2, C33A12.6, C33H5.11, C33H5.14, C33H5.18, C34B2.10, C34G6.4, C34H3.1, C35A5.2, C35C5.11, C35C5.2, C36B1.12, C38C6.2, C40C9.5, C40H1.4, C42C1.7, C42D4.2, C43F9.10, C43F9.6, C44B7.11, C44B7.8, C44B7.9, C44F1.5, C44H9.1, C46F4.2, C47A10.1, C48B4.2, C48B4.4, C48E7.8, C49A9.8, C49D10.4, C49D10.7, C49D10.8, C49F5.5, C49H3.1, C50D2.9, C50H2.2, C52B11.3, C52B9.9, C53D5.5, C54D1.1, C54G10.3, C54G4.8, C54G7.2, C56G2.6, D1009.1, D1022.1, D1073.1, D2024.3, D2063.2, D2085.6, DH11.3, DY3.7, E01G6.3, E01H11.1, E02D9.1, E03G2.2, E03H4.7, E04A4.5, E04F6.4, EGAP2.3, EGAP9.2, F01D4.1, F01D4.2, F01E11.1, F01G4.5, F02C9.3, F02E11.1, F02E9.7, F08B1.2, F08F1.1, F08G5.2, F08G5.5, F09A5.2, F09B12.3, F09B12.6, F09B9.1, F09G2.1, F09G2.6, F09G2.8, F10D2.11, F10D2.12, F10D2.2, F10D2.5, F10D2.6, F10D2.7, F10D2.9, F11A5.5, F11A5.8, F11C7.4, F11D5.3, F11E6.5, F11E6.8, F12A10.4, F12B6.1, F13D11.1, F13E6.5, F13H10.4, F13H10.5, F14B6.5, F14E5.3, F14F3.3, F14F4.3, F14H3.10, F15A2.2, F15A4.8, F15A8.5, F17B5.2, F17C11.7, F17C8.1, F18A12.5, F18A12.8, F18F11.5, F18H3.5, F19B6.4, F19H6.4, F20B4.6, F20B6.3, F21F3.3, F21F8.2, F21G4.2, F21H7.9, F22E10.1, F22E10.2, F22E10.3, F22E10.5, F22E5.3, F22F7.1, F23H11.9, F23H12.2, F23H12.6, F26D10.9, F26E4.6, F26F4.3, F26G1.6, F27D9.6, F27D9.7, F28A10.7, F28B3.9, F28C6.4, F28D1.11, F28D1.9, F28G4.1, F28G4.5, F29C4.1, F29D11.1, F29F11.2, F30A10.4, F31F4.7, F33A8.5, F33D11.9, F33D4.2, F33D4.4, F34D6.4, F35C11.5, F35C8.5, F35E2.6, F35H8.6, F36A2.9, F36G9.12, F36H1.2, F36H2.1, F37A4.1, F37C4.1, F37C4.2, F37C4.3, F39G3.1, F39G3.4, F39G3.5, F39G3.6, F40A3.5, F40B5.2, F41A4.1, F41D3.10, F41D3.2, F41D3.4, F41D3.5, F41E6.14, F41H10.7, F41H10.8, F42E11.1, F42F12.3, F42G8.6, F42G9.6, F43E2.4, F44B9.5, F44D12.2, F44F4.6, F44G4.8, F45E4.7, F45H10.1, F46C3.1, F46E10.9, F47B7.2, F47C10.6, F47F6.3, F48A11.1, F48C1.1, F48F5.5, F49B2.6, F49E12.10, F49E12.9, F49H12.6, F52E1.4, F52F10.3, F52F10.4, F52H2.6, F53B7.5, F53C11.2, F53C3.13, F53F4.5, F53G12.3, F53H8.4, F54C1.1, F54D8.2, F54F12.1, F54F7.5, F55A11.5, F55A12.4, F55D10.3, F55E10.6, F55F3.3, F55H2.5, F56A11.5, F56B3.7, F56C11.1, F56D1.5, F56G4.1, F56H11.3, F56H11.4, F56H6.11, F56H6.12, F57A10.3, F57B9.4, F57C12.4, F57C12.5, F57H12.4, F58A3.2, F59A1.10, F59A3.1, F59F3.1, F59F3.5, F59F4.4, F59F5.3, F59G1.1, H06H21.10, H06I04.5, H12I19.4, H12I19.5, H13N06.6, H14N18.4, H19N07.4, H21P03.3, H23N18.1, H23N18.2, H23N18.3, H41C03.3, K02E7.10, K02F3.6, K03A1.2, K03B8.2, K03B8.5, K04A8.10, K04D7.2, K04D7.4, K04F1.1, K04F10.4, K07B1.4, K07B1.5, K07E3.7, K07F5.6, K08B4.3, K08B4.4, K08C7.2, K08C7.5, K08E7.9, K08F9.3, K09A9.6, K09E10.1, K09E10.2, K09E4.2, K09F6.3, K10C2.3, K10F12.3, K11C4.5, K11D9.2, M01A10.3, M01B2.6, M01D7.2, M01E11.1, M02F4.8, M03A1.1, M03A1.3, M03A1.8, M03C11.5, M05B5.1, M176.6, M176.7, M79.2, M88.1, MTCE.11, MTCE.12, MTCE.21, MTCE.23, MTCE.25, MTCE.26, MTCE.31, MTCE.34, MTCE.35, MTCE.4, R01B10.4, R01E6.1, R02C2.3, R03D7.8, R03E9.2, R03H4.1, R03H4.5, R03H4.6, R04B5.9, R04D3.1, R05C11.3, R06B10.1, R07B7.4, R07B7.5, R07B7.6, R07B7.8, R07B7.9, R07E3.1, R07E3.5, R08F11.1, R09B5.6, R09D1.1, R09D1.10, R09D1.11, R09D1.12, R09D1.2, R09D1.3, R09D1.5, R09D1.6, R09F10.1, R134.1, R134.2, R13D11.4, R155.1, R155.2, R155.3, R173.1, R173.3, R57.1, T01A4.1, T01C2.1, T01G5.2, T02B5.1, T02B5.3, T02C5.5, T02D1.5, T03D3.1, T03D8.5, T03D8.6, T03G11.4, T04H1.7, T04H1.8, T05E11.5, T05G5.5, T05H4.1, T05H4.13, T05H4.4, T05H4.5, T06C12.10, T06C12.8, T06D4.4, T06D8.3, T06E8.1, T07C4.7, T07C5.1, T07D1.3, T07F10.1, T07H6.1, T07H6.2, T08D10.2, T08H10.4, T09A5.11, T09B4.1, T09E11.4, T09E11.5, T09E11.6, T09E11.7, T09H2.1, T10B10.8, T10B9.1, T10B9.10, T10B9.3, T10B9.5, T10H9.2, T10H9.5, T11F9.11, T12A2.2, T12B3.3, T12G3.4, T13C5.1, T13C5.6, T13F2.1, T14D7.2, T14E8.1, T14G10.7, T15D6.2, T15D6.5, T17A3.1, T18H9.2, T19B10.8, T19D7.7, T19H12.1, T19H12.10, T19H12.11, T19H12.6, T19H12.9, T19H5.1, T19H5.2, T19H5.3, T20D3.8, T21B6.1, T21E8.1, T21E8.2, T21E8.3, T22B11.2, T22D1.11, T22D1.4, T22F7.1, T22G5.5, T23F1.7, T23G4.4, T23G7.2, T24D1.4, T24H7.5, T25B9.7, T25G3.2, T26A5.1, T26A5.4, T26A8.2, T26C12.4, T26E4.4, T26H2.7, T27E4.6, T27E9.5, T27F7.3, T28D9.3, T28F3.5, T28F3.9, VF36H2L.1, VZK822L.1, W01A11.2, W01A8.4, W02A2.1, W02F12.2, W03B1.7, W03B1.8, W03F11.2, W03F11.4, W03F9.4, W04C9.1, W05H12.1, W06D12.3, W06G6.1, W07A12.6, W07A12.7, W08D2.4, W08D2.5, W09C2.3, W09D10.2, W09D6.6, W10C8.5, Y105C5B.2, Y105E8A.12, Y110A2AL.12, Y113G7C.1, Y116A8C.14, Y116A8C.16, Y116A8C.463, Y17D7A.4, Y22D7AL.8, Y22D7AR.7, Y37A1B.13, Y37D8A.10, Y37D8A.13, Y37E11AR.5, Y38C1AA.1, Y38F2AR.7, Y38H6C.16, Y38H6C.20, Y39B6A.18, Y39B6A.30, Y39D8C.1, Y39E4B.9, Y39G10AR.6, Y39H10A.2, Y40D12A.2, Y41E3.3, Y42A5A.1, Y42G9A.6, Y43F8A.3, Y43F8C.12, Y45F10D.3, Y46G5A.17, Y46G5A.2, Y46G5A.5, Y46H3A.6, Y47D3A.11, Y47D3A.30, Y47G6A.7, Y48G1C.5, Y48G8AL.11, Y48G9A.10, Y49A3A.1, Y49C4A.8, Y49E10.11, Y50E8A.16, Y51H4A.25, Y53C10A.9, Y53C12A.1, Y53F4B.2, Y53G8B.2, Y54E10A.14, Y54E10BR.1, Y54E10BR.5, Y54E2A.12, Y54E5A.1, Y54G2A.2, Y55D5A.5, Y55H10A.1, Y56A3A.2, Y56A3A.32, Y56A3A.36, Y57G11C.17, Y59A8B.21, Y59H11AR.2, Y5H2B.1, Y60A3A.14, Y61A9LA.1, Y66H1B.4, Y67A10A.1, Y67D8C.10, Y67D8C.9, Y67H2A.8, Y69A2AR.19, Y69E1A.3, Y6B3B.11, Y6B3B.5, Y71H2AM.25, Y71H2AM.4, Y71H2AR.2, Y73B6BL.7, Y74C10AR.3, Y75B8A.26, Y76A2A.2, Y7A5A.1, Y7A9A.1, Y80D3A.5, Y80D3A.8, Y82E9BR.3, Y87G2A.14, Y94H6A.5, Y97E10AR.2, ZC101.3, ZC155.4, ZC190.1, ZC239.7, ZC376.1, ZC376.2, ZC376.3, ZC412.2, ZC416.8, ZC443.5, ZC443.6, ZC455.3, ZC455.4, ZC455.5, ZC455.6, ZC506.3, ZC513.5, ZC8.1, ZK1067.1, ZK1248.14, ZK1251.3, ZK154.7, ZK256.1, ZK370.4, ZK40.1, ZK455.2, ZK455.7, ZK484.2, ZK550.1, ZK563.6, ZK637.8, ZK675.3, ZK678.8, ZK783.1, ZK792.1, ZK792.7, ZK816.5, ZK858.1, ZK896.8, ZK938.5, ZK938.6, ZK938.7, ZK945.1, ZK970.1, ZK970.5, ZK970.6

**(517) ENZYMES combined, curated** (removed 108 SIGNALING, 84 CHANNELS/TRANSPOTERS, 2 TRAFFICKING, 4 ECM, 7 ETC)

AC3.2, AC3.5, AC3.7, AC3.8, B0024.13, B0035.18, B0218.1, B0218.2, B0250.9, B0304.3, B0310.5, B0334.5, B0361.7, B0361.8, B0395.2, B0399.2, B0491.1, B0495.7, B0511.2, BE10.2, C01B10.10, C01B10.4, C01B10.7, C01B10.9, C01C10.3, C02A12.8, C02C2.5, C02H6.1, C03A7.11, C03C10.3, C04A11.4, C04F12.10, C04F5.7, C04F6.5, C05D12.1, C06B3.2, C07G3.9, C08B11.4, C08B11.8, C08B6.1, C08F11.8, C08F8.4, C08H9.10, C08H9.11, C08H9.12, C08H9.13, C08H9.3, C08H9.4, C08H9.7, C09F9.2, C10F3.2, C10H11.3, C10H11.4, C10H11.5, C10H11.6, C12D8.5, C13B4.1, C13D9.9, C14A4.3, C15F1.6, C15H11.4, C17A2.5, C17G1.3, C17H12.4, C18C4.3, C18G1.8, C18H2.4, C23G10.6, C23H4.2, C23H4.3, C23H4.4, C23H4.7, C25A1.5, C25H3.9, C27A12.9, C27A7.1, C27D8.4, C31A11.1, C31A11.5, C31A11.7, C32C4.7, C33A12.6, C33H5.11, C33H5.14, C33H5.18, C34B2.10, C34H3.1, C35A5.2, C35C5.11, C35C5.2, C36B1.12, C38C6.2, C40C9.5, C40H1.4, C42C1.7, C42D4.2, C43F9.10, C44B7.11, C44H9.1, C46F4.2, C48B4.2, C48E7.8, C49A9.8, C49D10.4, C49D10.7, C49D10.8, C49F5.5, C50D2.9, C52B9.9, C53D5.5, C54G4.8, C54G7.2, C56G2.6, D1009.1, D1022.1, D2024.3, D2063.2, D2085.6, DY3.7, E01G6.3, E03H4.7, E04F6.4, EGAP2.3, EGAP9.2, F01D4.1, F01D4.2, F01E11.1, F01G4.5, F08G5.2, F08G5.5, F09B12.3, F09B9.1, F09G2.6, F09G2.8, F10D2.11, F10D2.12, F10D2.2, F10D2.5, F10D2.6, F10D2.7, F10D2.9, F11A5.5, F11A5.8, F11E6.5, F12A10.4, F13D11.1, F13E6.5, F13H10.4, F13H10.5, F14B6.5, F14E5.3, F14F3.3, F14H3.10, F15A2.2, F15A4.8, F17B5.2, F17C11.7, F18A12.5, F18A12.8, F19H6.4, F20B4.6, F21F3.3, F21F8.2, F22E10.5, F22F7.1, F23H11.9, F26D10.9, F26E4.6, F26F4.3, F26G1.6, F27D9.6, F27D9.7, F28A10.7, F28B3.9, F28C6.4, F28D1.11, F28D1.9, F28G4.1, F28G4.5, F29F11.2, F30A10.4, F31F4.7, F33A8.5, F33D11.9, F33D4.4, F35C11.5, F35C8.5, F35E2.6, F35H8.6, F36A2.9, F36G9.12, F36H1.2, F37A4.1, F37C4.1, F37C4.2, F37C4.3, F39G3.1, F39G3.4, F39G3.5, F39G3.6, F41D3.10, F41D3.2, F41D3.4, F41D3.5, F41E6.14, F41H10.7, F41H10.8, F42F12.3, F42G8.6, F42G9.6, F44B9.5, F44F4.6, F45E4.7, F45H10.1, F46E10.9, F47B7.2, F47C10.6, F47F6.3, F48A11.1, F48C1.1, F48F5.5, F49B2.6, F49E12.10, F49E12.9, F49H12.6, F52F10.3, F52F10.4, F52H2.6, F53C11.2, F53C3.13, F53F4.5, F53G12.3, F53H8.4, F54C1.1, F54D8.2, F55A11.5, F55A12.4, F55D10.3, F55E10.6, F55H2.5, F56A11.5, F56B3.7, F56C11.1, F56D1.5, F56G4.1, F56H11.3, F56H11.4, F56H6.11, F56H6.12, F57B9.4, F59A1.10, F59A3.1, F59F4.4, F59G1.1, H06H21.10, H12I19.4, H12I19.5, H13N06.6, H14N18.4, H19N07.4, H21P03.3, H23N18.1, H23N18.2, H23N18.3, H41C03.3, K02E7.10, K02F3.6, K03A1.2, K03B8.2, K03B8.5, K04A8.10, K04D7.2, K04F1.1, K04F10.4, K07B1.4, K07B1.5, K08B4.3, K08B4.4, K08C7.2, K08C7.5, K08F9.3, K09A9.6, K09E10.1, K09E10.2, K09E4.2, K10C2.3, M01A10.3, M01B2.6, M01E11.1, M03A1.3, M03A1.8, M03C11.5, M79.2, M88.1, MTCE.11, MTCE.12, MTCE.21, MTCE.23, MTCE.25, MTCE.26, MTCE.31, MTCE.34, MTCE.35, MTCE.4, R01B10.4, R02C2.3, R03E9.2, R03H4.1, R03H4.5, R03H4.6, R04B5.9, R04D3.1, R07B7.4, R07B7.5, R07B7.6, R07B7.8, R07B7.9, R07E3.1, R07E3.5, R08F11.1, R09B5.6, R09D1.1, R09D1.10, R09D1.11, R09D1.2, R09D1.3, R09D1.5, R09D1.6, R09F10.1, R13D11.4, R155.1, R173.1, R173.3, R57.1, T01G5.2, T02B5.1, T02B5.3, T03D3.1, T03D8.6, T03G11.4, T04H1.7, T04H1.8, T05E11.5, T05G5.5, T05H4.1, T05H4.13, T05H4.4, T05H4.5, T06C12.10, T06C12.8, T06D4.4, T06D8.3, T06E8.1, T07C4.7, T07C5.1, T07D1.3, T07F10.1, T07H6.1, T07H6.2, T08D10.2, T08H10.4, T09A5.11, T09B4.1, T09E11.4, T09E11.5, T09E11.6, T09E11.7, T09H2.1, T10B10.8, T10B9.1, T10B9.10, T10B9.3, T10B9.5, T11F9.11, T12A2.2, T12B3.3, T12G3.4, T13C5.1, T13C5.6, T13F2.1, T14D7.2, T15D6.2, T15D6.5, T18H9.2, T19B10.8, T19D7.7, T19H12.1, T19H12.10, T19H12.11, T19H12.6, T19H12.9, T19H5.1, T19H5.2, T19H5.3, T20D3.8, T22B11.2, T22D1.11, T22D1.4, T22G5.5, T23F1.7, T23G4.4, T24D1.4, T25B9.7, T25G3.2, T26A5.4, T26A8.2, T26E4.4, T26H2.7, T27E4.6, T27E9.5, T27F7.3, T28D9.3, T28F3.5, T28F3.9, VF36H2L.1, VZK822L.1, W01A11.2, W01A8.4, W02A2.1, W02F12.2, W03B1.7, W03B1.8, W03F9.4, W06D12.3, W06G6.1, W07A12.6, W07A12.7, W08D2.4, W10C8.5, Y110A2AL.12, Y116A8C.14, Y116A8C.16, Y116A8C.463, Y17D7A.4, Y22D7AL.8, Y37A1B.13, Y37D8A.10, Y37D8A.13, Y37E11AR.5, Y38C1AA.1, Y38F2AR.7, Y38H6C.16, Y39E4B.9, Y39G10AR.6, Y39H10A.2, Y40D12A.2, Y41E3.3, Y42A5A.1, Y43F8A.3, Y45F10D.3, Y46G5A.17, Y46G5A.2, Y46G5A.5, Y46H3A.6, Y47D3A.30, Y48G9A.10, Y49A3A.1, Y49C4A.8, Y51H4A.25, Y53F4B.2, Y53G8B.2, Y54E10A.14, Y54E10BR.1, Y54E10BR.5, Y54E5A.1, Y56A3A.2, Y56A3A.32, Y56A3A.36, Y57G11C.17, Y5H2B.1, Y60A3A.14, Y66H1B.4, Y67A10A.1, Y67D8C.9, Y67H2A.8, Y6B3B.11, Y6B3B.5, Y71H2AM.25, Y71H2AM.4, Y71H2AR.2, Y73B6BL.7, Y7A5A.1, Y7A9A.1, Y80D3A.5, Y82E9BR.3, Y87G2A.14, Y94H6A.5, Y97E10AR.2, ZC101.3, ZC155.4, ZC376.1, ZC376.2, ZC376.3, ZC443.5, ZC443.6, ZC455.3, ZC455.4, ZC455.5, ZC455.6, ZC506.3, ZC513.5, ZC8.1, ZK1248.14, ZK1251.3, ZK154.7, ZK370.4, ZK40.1, ZK550.1, ZK563.6, ZK678.8, ZK792.1, ZK816.5, ZK858.1, ZK938.6, ZK938.7, ZK945.1, ZK970.1

**SIGNALING**

**(129) domain:7tm_1 (PF00001)** rhodopsin-type 7 transmembrane receptor (B0244.7, F57A8.4, W10C4.1, Y41D4B.24, ZK1307.7, ZK418.6, ZK418.7 from no other domain) (B0244.6, R04D3.10 moved to serpentine receptors)

AC7.1, AH9.1, B0244.10, B0244.5, B0244.7, B0334.6, B0563.6, C01F1.4, C02D4.2, C02H7.2, C06G4.5, C09B7.1, C10C6.2, C15B12.5, C15H11.2, C16D6.2, C17H11.1, C24A8.1, C24B5.1, C25B8.5, C25B8.7, C25G6.5, C26F1.6, C30B5.5, C30F12.6, C38C10.1, C39E6.6, C43C3.2, C48C5.1, C49A9.7, C50F7.1, C50H2.1, C52B11.3, C53C7.1, C54A12.2, C56A3.3, C56G3.1, D1014.2, D1022.6, E04D5.2, F01E11.5, F02E8.2, F13D2.2, F13D2.3, F14D12.6, F14F4.1, F15A8.5, F16C3.1, F16D3.7, F21C10.12, F31B9.1, F35G8.1, F36D4.4, F39B3.2, F41E7.3, F42C5.2, F47D12.1, F52D10.4, F53A9.5, F53B7.2, F54D7.3, F55E10.7, F56B6.5, F57A8.4, F57C9.6, F57H12.4, F59B2.13, F59C12.2, F59D12.1, H02I12.3, H09F14.1, H10E21.2, H22D07.1, H23L24.4, K02F2.6, K03H6.1, K03H6.5, K06C4.8, K06C4.9, K09G1.4, K10B4.4, K10C8.2, M03F4.3, R02D5.6, R03A10.6, R106.2, R11F4.2, R12C12.3, R13H7.2, T02D1.6, T02E9.1, T02E9.3, T05A1.1, T07D10.2, T07D4.1, T07F8.2, T14B1.2, T14E8.3, T19F4.1, T21B4.4, T22D1.12, T23B3.4, T23C6.5, T27B2.1, W05B5.2, W10C4.1, Y105C5A.23, Y116A8B.5, Y22D7AR.13, Y23H5B.4, Y34D9A.2, Y39A3B.5, Y40H4A.1, Y41D4A.8, Y41D4B.24, Y54E2A.1, Y54G2A.35, Y57A10C.10, Y58G8A.4, Y59H11AL.1, Y70D2A.1, ZC374.1, ZC412.1, ZC84.4, ZK1307.7, ZK418.6, ZK418.7, ZK455.3, ZK813.5

**(14) domain:7tm_2,3,6,7 (PF00002, PF00003, PF02949, PF08395)** 7 transmembrane receptors

B0286.2, B0457.1, C13B9.4, C14F11.3, C18B12.2, C50H2.2, F35H10.10, F39B2.8, F45H11.4, Y41G9A.4, Y4C6A.2, ZC504.5, ZC506.4, ZK180.1

**(69) domain:kinase (PF00069, PF07714, SM00219, SM00220, SM00221)**

AH6.1, B0024.6, B0198.3, B0240.3, B0252.1, C01G6.8, C04H5.3, C05D2.1, C06A12.4, C08H9.5, C16B8.1, C16D9.2, C17F4.6, C24G6.2, C25F6.4, C32D5.2, C49H3.1, D1073.1, E01H11.1, E02D9.1, F08B1.2, F08F1.1, F09A5.2, F09B12.6, F09G2.1, F11D5.3, F11E6.8, F18F11.5, F18H3.5, F21H7.9, F22E5.3, F23H12.6, F29C4.1, F40A3.5, F46C3.1, F52E1.4, F54F7.5, F58A3.2, F59F3.1, F59F3.5, F59F5.3, M03A1.1, M176.6, M176.7, R01E6.1, R09D1.12, R134.1, R134.2, T01A4.1, T03D8.5, T10H9.2, T14E8.1, T17A3.1, T26C12.4, W03F11.2, Y105C5B.2, Y38H6C.20, Y48B6A.10, Y53C12A.1, Y55D5A.5, Y69E1A.3, ZC239.7, ZC412.2, ZK1067.1, ZK455.2, ZK896.8, ZK938.5, ZK970.5, ZK970.6

**(33) domain:ANF_receptor (PF01094)** receptor family ligand binding region

AH6.1, B0024.6, B0280.12, C04H5.3, C06A8.9, C17F4.6, C30A5.10, C49H3.1, F07F6.6, F08B1.2, F21H7.9, F23H12.6, F45H11.4, F52E1.4, R01E6.1, R134.1, R134.2, T01A4.1, T01C3.10, T03D8.5, T26C12.4, W03F11.2, Y41G9A.4, Y4C6A.2, ZC196.7, ZC239.7, ZC412.2, ZC506.4, ZK180.1, ZK455.2, ZK896.8, ZK970.5, ZK970.6

**(30) domain:gcyc (PF00211, SM00044)** adenylate and guanylate cyclase catalytic domain

AH6.1, B0024.6, B0240.3, C04H5.3, C06A12.4, C10F3.3, C17F4.6, C44F1.5, C49H3.1, F08B1.2, F17C8.1, F21H7.9, F22E5.3, F23H12.6, F52E1.4, R01E6.1, R134.1, R134.2, T01A4.1, T01C2.1, T03D8.5, T26C12.4, W03F11.2, Y105C5B.2, ZC239.7, ZC412.2, ZK455.2, ZK896.8, ZK970.5, ZK970.6

**(30) domain:Patched (PF02460)** receptor for the morphogene Sonic Hedgehog

C24B5.3, C32E8.8, C41D7.2, C45B2.7, C53C11.3, C54A12.1, F02E8.6, F09G8.4, F21H12.4, F31F6.5, F43D9.1, F44F4.4, F46G10.5, F54G8.5, F55F8.1, F56C11.2, K07A3.2, K07C10.1, R09H10.4, T07H8.6, T21H3.2, Y110A2AL.8, Y18D10A.7, Y38F1A.3, Y39A1B.2, Y53F4B.28, Y65B4BR.3, Y80D3A.7, ZK270.1, ZK675.1

**(19) domain: PTP (PF00102, PF00782, SM00194, SM00195)** protein tyrosine phosphatases

B0244.2, F44G4.8, F54F12.1, H06I04.5, K04D7.4, K07F5.6, K09F6.3, M05B5.1, R06B10.1, R155.2, R155.3, W03F11.4, Y113G7C.1, Y22D7AR.7, Y39B6A.18, Y39B6A.30, Y48G1C.5, Y69A2AR.19, Y80D3A.8

**(6) domain:Phtase (PF00149, SM00156)** phosphoprotein phosphatases

B0511.13, F02E9.7, F40B5.2, R03D7.8, T23G7.2, ZK792.7

**(224) GO:signal**

AC7.1, AH6.1, AH9.1, B0024.6, B0212.5, B0240.3, B0273.4, B0286.2, B0334.11, B0334.6, B0457.1, B0563.6, C01F1.4, C01G6.8, C02D4.2, C02H7.2, C04H5.3, C05D2.1, C06A12.4, C06E1.4, C06G4.5, C07F11.1, C09B7.1, C10C6.2, C10F3.3, C13B9.4, C15B12.5, C15B12.7, C15H11.2, C16D6.2, C17F4.6, C17H11.1, C18B12.2, C18E3.8, C24A8.1, C24B5.1, C25B8.1, C25B8.5, C25B8.7, C25G6.5, C26F1.6, C26G2.1, C30B5.5, C30F12.6, C31E10.8, C32D5.2, C34B2.10, C38C10.1, C39E6.6, C43C3.2, C43G2.1, C44F1.5, C48B4.2, C48C5.1, C49A9.7, C49H3.1, C50F7.1, C50H2.1, C50H2.2, C52B11.3, C53C7.1, C54A12.2, C56A3.3, C56A3.7, C56G3.1, D1009.3, D1014.2, D1022.6, DY3.7, E01H11.1, E04D5.2, F01E11.5, F02E8.2, F08B1.2, F13D2.2, F13D2.3, F14D12.6, F14F4.1, F15A8.5, F15B9.7, F16B3.1, F16C3.1, F16D3.7, F17C8.1, F21C10.12, F21H7.9, F22E5.3, F23H12.6, F26F4.3, F27E11.3, F31B9.1, F31D5.4, F31D5.5, F31F6.5, F35G8.1, F35H10.10, F35H12.3, F36D4.4, F36H1.4, F39B2.8, F39B3.2, F41E7.3, F42C5.2, F45D3.5, F45H11.4, F47D12.1, F52D10.4, F52E1.4, F53A9.5, F53B7.2, F54D7.3, F55A11.3, F55E10.7, F56A11.5, F56B6.5, F57C9.6, F57H12.4, F58A3.2, F59B2.13, F59C12.2, F59D12.1, H02I12.3, H09F14.1, H10E21.2, H22D07.1, H23L24.4, K02E10.8, K02F2.6, K03H6.1, K03H6.5, K06C4.6, K06C4.8, K06C4.9, K07F5.6, K08F11.5, K09G1.4, K10B4.4, K10C8.2, K10F12.3, M03A1.1, M03F4.3, R01E6.1, R03A10.6, R106.2, R107.8, R11F4.2, R11G1.6, R12C12.3, R134.1, R134.2, R13H7.2, T01A4.1, T01C2.1, T02C5.5, T02D1.6, T02E9.1, T02E9.3, T03D8.5, T05A1.1, T07D10.2, T07D4.1, T07F8.2, T09E8.3, T10H10.1, T13C5.1, T13F2.8, T14B1.2, T14E8.3, T19F4.1, T21B4.4, T21C12.1, T22D1.12, T23B3.4, T23C6.5, T23D8.1, T26C12.4, T27B2.1, W02D7.7, W03F11.2, W05B5.2, W07G1.5, Y105C5A.23, Y105C5B.2, Y116A8B.5, Y22D7AR.13, Y23H5B.4, Y34D9A.2, Y37D8A.10, Y37E11AL.5, Y39A3B.5, Y40H4A.1, Y41D4A.8, Y41G9A.4, Y48E1B.14, Y4C6A.2, Y54E10BR.5, Y54E2A.1, Y54G2A.35, Y54G9A.3, Y55D5A.5, Y55F3AL.1, Y58G8A.4, Y59H11AL.1, Y70D2A.1, Y71F9B.5, ZC239.7, ZC374.1, ZC412.1, ZC412.2, ZC506.4, ZC84.4, ZK1058.2, ZK1067.1, ZK1086.1, ZK180.1, ZK455.2, ZK455.3, ZK524.1, ZK643.3, ZK813.5, ZK896.8, ZK945.9, ZK970.5, ZK970.6

**(320) GO:receptor**

AC7.1, AH9.1, B0240.3, B0244.2, B0273.4, B0280.12, B0286.2, B0334.6, B0361.8, B0416.5, B0457.1, B0563.6, C01A2.3, C01F1.2, C01F1.4, C01G6.8, C02D4.2, C02H7.2, C03C10.3, C05D2.1, C06A8.9, C06B8.7, C06E1.4, C06G4.5, C07F11.1, C08B11.4, C09B7.1, C10C6.2, C13B9.4, C14F11.3, C15B12.5, C15C7.1, C15H11.2, C16D6.2, C17D12.6, C17E4.9, C17H11.1, C18B12.2, C18E9.10, C24A8.1, C24B5.1, C24B5.3, C25B8.1, C25B8.5, C25B8.7, C25G6.5, C26F1.6, C26G2.1, C26H9A.1, C30B5.5, C30F12.6, C31E10.7, C31E10.8, C32D5.2, C32E8.8, C34B2.10, C35C5.5, C38C10.1, C39E6.6, C39E9.10, C41D7.2, C43C3.2, C43H6.9, C44H4.2, C45B2.7, C47E12.2, C48C5.1, C49A9.7, C49H3.1, C50F7.1, C50H2.1, C50H2.2, C52B11.3, C53C11.3, C53C7.1, C54A12.1, C54A12.2, C56A3.3, C56A3.7, C56C10.13, C56G3.1, D1009.3, D1014.2, D1022.6, D2089.2, D2092.3, E04D5.2, EGAP2.3, F01E11.5, F02E8.2, F02E8.6, F07C3.10, F07F6.6, F09E8.7, F09G8.4, F10C5.2, F10D2.9, F11H8.2, F13D2.2, F13D2.3, F14D12.6, F14F4.1, F15A8.5, F15B9.7, F16C3.1, F16D3.7, F21C10.12, F21F3.5, F21H12.4, F22A3.3, F23H12.6, F25G6.4, F27E11.3, F28D1.9, F29C4.1, F31B9.1, F31F6.5, F33D11.11, F33D4.2, F35C8.4, F35G8.1, F35H10.10, F35H12.3, F36D4.4, F36F2.4, F36H1.2, F36H1.4, F39B2.8, F39B3.2, F40G9.1, F41B4.4, F41E7.3, F42C5.2, F43D9.1, F44D12.2, F44F4.4, F45H11.4, F46G10.5, F47D12.1, F48F7.2, F48G7.3, F49B2.6, F52D10.4, F53A9.5, F53B7.2, F54D1.6, F54D7.3, F54F2.1, F54G8.3, F54G8.5, F55A11.2, F55E10.7, F55F8.1, F56A8.7, F56B6.5, F56C11.2, F57B10.10, F57C9.6, F57H12.4, F58A3.2, F58G11.1, F59B1.9, F59B2.13, F59C12.2, F59D12.1, H02I12.3, H04J21.3, H09F14.1, H10E21.2, H19M22.2, H22D07.1, H23L24.4, H35N03.1, JC8.5, K01H12.2, K02E10.8, K02F2.6, K03F8.2, K03H6.1, K03H6.5, K06C4.6, K06C4.8, K06C4.9, K07A3.2, K07C10.1, K07D8.1, K07F5.6, K09G1.4, K09H9.6, K10B4.4, K10C8.2, K10D3.1, K11D9.2, K11G12.2, K11G12.7, M02B1.1, M03A1.1, M03F4.3, R03A10.6, R03E1.2, R09H10.4, R106.2, R107.8, R10E11.2, R10E11.8, R11F4.2, R11G11.12, R12C12.3, R12E2.2, R13H7.2, T01A4.1, T01B11.3, T01B11.4, T01C3.10, T01H3.1, T02C5.1, T02C5.5, T02D1.6, T02E9.1, T02E9.3, T03D8.5, T05A1.1, T05H4.4, T05H4.5, T07C4.7, T07D10.2, T07D4.1, T07F8.2, T07H8.6, T08D2.2, T08G11.5, T10H9.4, T11F8.3, T14B1.2, T14E8.3, T14G10.7, T19F4.1, T19H12.8, T21B4.4, T21C12.1, T21H3.2, T22D1.12, T23B3.4, T23C6.5, T23D8.1, T26C12.4, T27B2.1, T27E9.1, VZK822L.1, W01A8.4, W02A2.1, W02B9.1, W05B5.2, W05H12.1, Y105C5A.23, Y110A2AL.8, Y110A7A.11, Y110A7A.3, Y116A8B.5, Y11D7A.9, Y18D10A.7, Y22D7AR.13, Y23H5B.4, Y25C1A.7, Y34D9A.2, Y37D8A.10, Y38F1A.3, Y38F2AR.7, Y39A1B.2, Y39A3B.5, Y39E4B.7, Y40H4A.1, Y41D4A.8, Y41G9A.4, Y47D7A.16, Y47H9C.4, Y49E10.11, Y4C6A.2, Y53C12A.1, Y53F4B.28, Y54E2A.1, Y54G2A.25, Y54G2A.35, Y54G9A.3, Y55D5A.5, Y55F3AL.1, Y55H10A.1, Y56A3A.22, Y56A3A.32, Y58G8A.4, Y59H11AL.1, Y60A3A.14, Y65B4BR.3, Y70D2A.1, Y71F9B.5, Y73C8B.4, Y80D3A.7, Y82E9BR.3, ZC196.7, ZC374.1, ZC412.1, ZC482.1, ZC506.4, ZC84.4, ZK1058.2, ZK1067.1, ZK1086.1, ZK180.1, ZK270.1, ZK455.3, ZK637.8, ZK643.3, ZK675.1, ZK686.3, ZK792.2, ZK792.3, ZK813.5, ZK858.7, ZK896.8, ZK938.5

**(57) GO:protein_kinase_activity**

AH6.1, B0024.6, B0252.1, C01G6.8, C05D2.1, C06A12.4, C08H9.5, C16B8.1, C16D9.2, C17F4.6, C24G6.2, C32D5.2, D1073.1, E01H11.1, E02D9.1, F08B1.2, F08F1.1, F09A5.2, F09B12.6, F09G2.1, F11D5.3, F11E6.8, F18F11.5, F18H3.5, F21H7.9, F23H12.6, F29C4.1, F40A3.5, F46C3.1, F52E1.4, F58A3.2, F59F3.1, F59F3.5, F59F5.3, M03A1.1, M176.6, M176.7, R09D1.12, R134.1, R134.2, T01A4.1, T03D8.5, T10H9.2, T14E8.1, T17A3.1, T26C12.4, W03F11.2, Y38H6C.20, Y53C12A.1, Y55D5A.5, Y69E1A.3, ZC412.2, ZK1067.1, ZK896.8, ZK938.5, ZK970.5, ZK970.6

**(69) GO:protein_serine/threonine_kinase_activity**

AH6.1, B0024.6, B0198.3, B0240.3, B0252.1, C01G6.8, C04H5.3, C05D2.1, C06A12.4, C08H9.5, C16B8.1, C16D9.2, C17F4.6, C24G6.2, C25F6.4, C32D5.2, C49H3.1, D1073.1, E01H11.1, E02D9.1, F08B1.2, F08F1.1, F09A5.2, F09B12.6, F09G2.1, F11D5.3, F11E6.8, F18F11.5, F18H3.5, F21H7.9, F22E5.3, F23H12.6, F29C4.1, F40A3.5, F44D12.2, F46C3.1, F52E1.4, F58A3.2, F59F3.1, F59F3.5, F59F5.3, M03A1.1, M176.6, M176.7, R01E6.1, R09D1.12, R134.1, R134.2, T01A4.1, T03D8.5, T10H9.2, T14E8.1, T17A3.1, T26C12.4, W03F11.2, W05H12.1, Y105C5B.2, Y38H6C.20, Y53C12A.1, Y55D5A.5, Y69E1A.3, ZC239.7, ZC412.2, ZK1067.1, ZK455.2, ZK896.8, ZK938.5, ZK970.5, ZK970.6

**(68) GO:protein_tyrosine_kinase_activity**

AH6.1, B0024.6, B0198.3, B0240.3, B0252.1, C01G6.8, C04H5.3, C05D2.1, C06A12.4, C08H9.5, C16B8.1, C16D9.2, C17F4.6, C24G6.2, C25F6.4, C31E10.8, C32D5.2, C49H3.1, D1073.1, E01H11.1, E02D9.1, F08B1.2, F08F1.1, F09A5.2, F09B12.6, F09G2.1, F11D5.3, F11E6.8, F18F11.5, F18H3.5, F21H7.9, F22E5.3, F23H12.6, F29C4.1, F40A3.5, F46C3.1, F52E1.4, F54F7.5, F58A3.2, F59F3.1, F59F3.5, F59F5.3, M03A1.1, M176.6, M176.7, R01E6.1, R09D1.12, R134.1, R134.2, T01A4.1, T03D8.5, T10H9.2, T14E8.1, T17A3.1, T26C12.4, W03F11.2, Y38H6C.20, Y53C12A.1, Y55D5A.5, Y69E1A.3, ZC239.7, ZC412.2, ZK1067.1, ZK455.2, ZK896.8, ZK938.5, ZK970.5, ZK970.6

**(19) GO:protein_tyrosine_phosphatase_activity**

B0244.2, F44G4.8, F54F12.1, H06I04.5, K04D7.4, K07F5.6, K09F6.3, M05B5.1, R06B10.1, R155.2, R155.3, W03F11.4, Y113G7C.1, Y22D7AR.7, Y39B6A.18, Y39B6A.30, Y48G1C.5, Y69A2AR.19, Y80D3A.8

**(131) GO:G-protein_coupled_receptor_protein_signaling_pathway** (removed K07F5.6)

AC7.1, AH9.1, B0286.2, B0334.6, B0457.1, B0563.6, C01F1.4, C02D4.2, C02H7.2, C06G4.5, C09B7.1, C10C6.2, C13B9.4, C15B12.5, C15H11.2, C16D6.2, C17H11.1, C18B12.2, C24A8.1, C24B5.1, C25B8.5, C25B8.7, C25G6.5, C26F1.6, C30B5.5, C30F12.6, C38C10.1, C39E6.6, C43C3.2, C48C5.1, C49A9.7, C50F7.1, C50H2.1, C50H2.2, C52B11.3, C53C7.1, C54A12.2, C56A3.3, C56G3.1, D1014.2, D1022.6, E04D5.2, F01E11.5, F02E8.2, F13D2.2, F13D2.3, F14D12.6, F14F4.1, F15A8.5, F16C3.1, F16D3.7, F21C10.12, F31B9.1, F35G8.1, F35H10.10, F36D4.4, F39B2.8, F39B3.2, F41E7.3, F42C5.2, F45H11.4, F47D12.1, F52D10.4, F53A9.5, F53B7.2, F54D7.3, F55E10.7, F56B6.5, F57C9.6, F57H12.4, F59B2.13, F59C12.2, F59D12.1, H02I12.3, H09F14.1, H10E21.2, H22D07.1, H23L24.4, K02F2.6, K03H6.1, K03H6.5, K06C4.8, K06C4.9, K09G1.4, K10B4.4, K10C8.2, M03F4.3, R03A10.6, R106.2, R11F4.2, R12C12.3, R13H7.2, T02D1.6, T02E9.1, T02E9.3, T05A1.1, T07D10.2, T07D4.1, T07F8.2, T14B1.2, T14E8.3, T19F4.1, T21B4.4, T22D1.12, T23B3.4, T23C6.5, T27B2.1, W05B5.2, Y105C5A.23, Y116A8B.5, Y22D7AR.13, Y23H5B.4, Y34D9A.2, Y39A3B.5, Y40H4A.1, Y41D4A.8, Y41G9A.4, Y4C6A.2, Y54E2A.1, Y54G2A.35, Y58G8A.4, Y59H11AL.1, Y70D2A.1, ZC374.1, ZC412.1, ZC506.4, ZC84.4, ZK180.1, ZK455.3, ZK643.3, ZK813.5

**(28) from other groups, not grouped in any of above**

B0410.2, B0511.12, C01F1.2, C15A7.2, C18A11.5, C41D11.9, D1037.2, F31A9.3, F32A7.3, F33D4.2, F43C9.4, F56D1.2, K04B12.1, K08D9.3, R06B9.6, R107.8, T01C4.2, T04F8.2, T07C4.8, T19B4.7, T22C1.7, Y34D9B.1, Y54E2A.12, Y54E5B.1, Y73C8B.4, ZC434.6, ZK377.2, F33D4.2, R107.8, T05F1.1, Y73C8B.4

**(13) from "no other domain", not grouped in any of above**

B0213.2, B0464.3, C02B8.5, C13G3.2, C15F1.3, C41G11.4, C49C3.21, F07C3.1, F23H11.5, F56E10.3, K09E4.5, ZC190.2, ZK622.2

**(396) SIGNALING combined** (removed inx-*,grl-*,nmr-*,"GO:receptor-mediated endocytosis" except F21H12.4, F58A3.2, Y53C12A.1, ZK675.1, ZK792.2, ZK792.3)

AC7.1, AH6.1, AH9.1, B0024.6, B0198.3, B0212.5, B0213.2, B0240.3, B0244.10, B0244.2, B0244.5, B0244.7, B0252.1, B0273.4, B0286.2, B0334.11, B0334.6, B0410.2, B0457.1, B0464.3, B0511.12, B0511.13, B0563.6, C01F1.2, C01F1.4, C01G6.8, C02B8.5, C02D4.2, C02H7.2, C04H5.3, C05D2.1, C06A12.4, C06B8.7, C06G4.5, C07F11.1, C08H9.5, C09B7.1, C10C6.2, C10F3.3, C13B9.4, C13G3.2, C14F11.3, C15A7.2, C15B12.5, C15B12.7, C15C7.1, C15F1.3, C15H11.2, C16B8.1, C16D6.2, C16D9.2, C17D12.6, C17F4.6, C17H11.1, C18A11.5, C18B12.2, C18E3.8, C24A8.1, C24B5.1, C24B5.3, C24G6.2, C25B8.1, C25B8.5, C25B8.7, C25F6.4, C25G6.5, C26F1.6, C26G2.1, C30A5.10, C30B5.5, C30F12.6, C31E10.8, C32D5.2, C32E8.8, C35C5.5, C38C10.1, C39E6.6, C39E9.10, C41D11.9, C41D7.2, C41G11.4, C43C3.2, C43G2.1, C44F1.5, C45B2.7, C48B4.2, C48C5.1, C49A9.7, C49C3.21, C49H3.1, C50F7.1, C50H2.1, C50H2.2, C52B11.3, C53C11.3, C53C7.1, C54A12.1, C54A12.2, C56A3.3, C56A3.7, C56G3.1, D1009.3, D1014.2, D1022.6, D1037.2, D1073.1, D2092.3, DY3.7, E01H11.1, E02D9.1, E04D5.2, F01E11.5, F02E8.2, F02E8.6, F02E9.7, F07C3.1, F07C3.10, F08B1.2, F08F1.1, F09A5.2, F09B12.6, F09E8.7, F09G2.1, F09G8.4, F11D5.3, F11E6.8, F11H8.2, F13D2.2, F13D2.3, F14D12.6, F14F4.1, F15A8.5, F15B9.7, F16B3.1, F16C3.1, F16D3.7, F17C8.1, F18F11.5, F18H3.5, F21C10.12, F21F3.5, F21H12.4, F21H7.9, F22E5.3, F23H11.5, F23H12.6, F25G6.4, F26F4.3, F27E11.3, F29C4.1, F31A9.3, F31B9.1, F31D5.4, F31D5.5, F31F6.5, F32A7.3, F33D11.11, F33D4.2, F35C8.4, F35G8.1, F35H10.10, F35H12.3, F36D4.4, F36F2.4, F36H1.4, F39B2.8, F39B3.2, F40A3.5, F40B5.2, F41E7.3, F42C5.2, F43C9.4, F43D9.1, F44D12.2, F44F4.4, F44G4.8, F45D3.5, F45H11.4, F46C3.1, F46G10.5, F47D12.1, F48F7.2, F48G7.3, F52D10.4, F52E1.4, F53A9.5, F53B7.2, F54D7.3, F54F12.1, F54F7.5, F54G8.5, F55A11.3, F55E10.7, F55F8.1, F56A11.5, F56A8.7, F56B6.5, F56C11.2, F56D1.2, F56E10.3, F57A8.4, F57C9.6, F57H12.4, F58A3.2, F59B1.9, F59B2.13, F59C12.2, F59D12.1, F59F3.1, F59F3.5, F59F5.3, H02I12.3, H06I04.5, H09F14.1, H10E21.2, H22D07.1, H23L24.4, H35N03.1, K02E10.8, K02F2.6, K03H6.1, K03H6.5, K04B12.1, K04D7.4, K06C4.6, K06C4.8, K06C4.9, K07A3.2, K07C10.1, K07F5.6, K08D9.3, K08F11.5, K09E4.5, K09F6.3, K09G1.4, K10B4.4, K10C8.2, K10F12.3, K11G12.2, K11G12.7, M03A1.1, M03F4.3, M05B5.1, M176.6, M176.7, R01E6.1, R02D5.6, R03A10.6, R03D7.8, R03E1.2, R06B10.1, R06B9.6, R09D1.12, R09H10.4, R106.2, R107.8, R11F4.2, R11G1.6, R11G11.12, R12C12.3, R134.1, R134.2, R13H7.2, R155.2, R155.3, T01A4.1, T01C2.1, T01C4.2, T02C5.1, T02C5.5, T02D1.6, T02E9.1, T02E9.3, T03D8.5, T04F8.2, T05A1.1, T05F1.1, T07C4.8, T07D10.2, T07D4.1, T07F8.2, T07H8.6, T08G11.5, T09E8.3, T10H10.1, T10H9.2, T10H9.4, T13C5.1, T13F2.8, T14B1.2, T14E8.1, T14E8.3, T17A3.1, T19B4.7, T19F4.1, T19H12.8, T21B4.4, T21C12.1, T21H3.2, T22C1.7, T22D1.12, T23B3.4, T23C6.5, T23D8.1, T23G7.2, T26C12.4, T27B2.1, W02D7.7, W03F11.2, W03F11.4, W05B5.2, W05H12.1, W07G1.5, W10C4.1, Y105C5A.23, Y105C5B.2, Y110A2AL.8, Y110A7A.3, Y113G7C.1, Y116A8B.5, Y18D10A.7, Y22D7AR.13, Y22D7AR.7, Y23H5B.4, Y34D9A.2, Y34D9B.1, Y37E11AL.5, Y38F1A.3, Y38H6C.20, Y39A1B.2, Y39A3B.5, Y39B6A.18, Y39B6A.30, Y40H4A.1, Y41D4A.8, Y41D4B.24, Y41G9A.4, Y47H9C.4, Y48B6A.10, Y48E1B.14, Y48G1C.5, Y4C6A.2, Y53C12A.1, Y53F4B.28, Y54E10BR.5, Y54E2A.1, Y54E2A.12, Y54E5B.1, Y54G2A.25, Y54G2A.35, Y54G9A.3, Y55D5A.5, Y55F3AL.1, Y57A10C.10, Y58G8A.4, Y59H11AL.1, Y65B4BR.3, Y69A2AR.19, Y69E1A.3, Y70D2A.1, Y71F9B.5, Y73C8B.4, Y80D3A.7, Y80D3A.8, ZC190.2, ZC239.7, ZC374.1, ZC412.1, ZC412.2, ZC434.6, ZC482.1, ZC504.5, ZC506.4, ZC84.4, ZK1058.2, ZK1067.1, ZK1086.1, ZK1307.7, ZK180.1, ZK270.1, ZK377.2, ZK418.6, ZK418.7, ZK455.2, ZK455.3, ZK524.1, ZK622.2, ZK643.3, ZK675.1, ZK792.7, ZK813.5, ZK896.8, ZK938.5, ZK945.9, ZK970.5, ZK970.6

**(354) SIGNALING combined, curated** (removed 23 TRANSPORTERS, 8 TRAFFICKING, 6 ENZYMES, 6 ETC)

AC7.1, AH6.1, AH9.1, B0024.6, B0198.3, B0212.5, B0213.2, B0240.3, B0244.10, B0244.5, B0244.7, B0252.1, B0273.4, B0286.2, B0334.11, B0334.6, B0410.2, B0457.1, B0464.3, B0511.12, B0511.13, B0563.6, C01F1.2, C01F1.4, C01G6.8, C02B8.5, C02D4.2, C02H7.2, C04H5.3, C05D2.1, C06A12.4, C06B8.7, C06G4.5, C07F11.1, C08H9.5, C09B7.1, C10C6.2, C10F3.3, C13B9.4, C13G3.2, C14F11.3, C15A7.2, C15B12.5, C15F1.3, C15H11.2, C16B8.1, C16D6.2, C16D9.2, C17D12.6, C17F4.6, C17H11.1, C18A11.5, C18B12.2, C18E3.8, C24A8.1, C24B5.1, C24B5.3, C24G6.2, C25B8.5, C25B8.7, C25F6.4, C25G6.5, C26F1.6, C26G2.1, C30A5.10, C30B5.5, C30F12.6, C31E10.8, C32D5.2, C32E8.8, C38C10.1, C39E6.6, C41D11.9, C41D7.2, C41G11.4, C43C3.2, C43G2.1, C44F1.5, C45B2.7, C48C5.1, C49A9.7, C49C3.21, C49H3.1, C50F7.1, C50H2.1, C50H2.2, C52B11.3, C53C11.3, C53C7.1, C54A12.1, C54A12.2, C56A3.3, C56G3.1, D1009.3, D1014.2, D1022.6, D1037.2, D1073.1, E01H11.1, E02D9.1, E04D5.2, F01E11.5, F02E8.2, F02E8.6, F02E9.7, F07C3.1, F08B1.2, F08F1.1, F09A5.2, F09B12.6, F09G2.1, F09G8.4, F11D5.3, F11E6.8, F13D2.2, F13D2.3, F14D12.6, F14F4.1, F15A8.5, F15B9.7, F16C3.1, F16D3.7, F17C8.1, F18F11.5, F18H3.5, F21C10.12, F21H12.4, F21H7.9, F22E5.3, F23H11.5, F23H12.6, F27E11.3, F29C4.1, F31A9.3, F31B9.1, F31D5.4, F31D5.5, F31F6.5, F32A7.3, F33D11.11, F33D4.2, F35G8.1, F35H10.10, F35H12.3, F36D4.4, F36H1.4, F39B2.8, F39B3.2, F40A3.5, F40B5.2, F41E7.3, F42C5.2, F43C9.4, F43D9.1, F44D12.2, F44F4.4, F44G4.8, F45D3.5, F45H11.4, F46C3.1, F46G10.5, F47D12.1, F52D10.4, F52E1.4, F53A9.5, F53B7.2, F54D7.3, F54F12.1, F54F7.5, F54G8.5, F55A11.3, F55E10.7, F55F8.1, F56B6.5, F56C11.2, F56D1.2, F56E10.3, F57A8.4, F57C9.6, F57H12.4, F58A3.2, F59B2.13, F59C12.2, F59D12.1, F59F3.1, F59F3.5, F59F5.3, H02I12.3, H06I04.5, H09F14.1, H10E21.2, H22D07.1, H23L24.4, K02E10.8, K02F2.6, K03H6.1, K03H6.5, K04B12.1, K04D7.4, K06C4.8, K06C4.9, K07A3.2, K07C10.1, K07F5.6, K08D9.3, K08F11.5, K09E4.5, K09F6.3, K09G1.4, K10B4.4, K10C8.2, K10F12.3, M03A1.1, M03F4.3, M05B5.1, M176.6, M176.7, R01E6.1, R02D5.6, R03A10.6, R03D7.8, R03E1.2, R06B10.1, R06B9.6, R09D1.12, R09H10.4, R106.2, R107.8, R11F4.2, R11G1.6, R12C12.3, R134.1, R134.2, R13H7.2, R155.2, R155.3, T01A4.1, T01C2.1, T01C4.2, T02C5.1, T02D1.6, T02E9.1, T02E9.3, T03D8.5, T04F8.2, T05A1.1, T05F1.1, T07C4.8, T07D10.2, T07D4.1, T07F8.2, T07H8.6, T09E8.3, T10H9.2, T14B1.2, T14E8.1, T14E8.3, T17A3.1, T19B4.7, T19F4.1, T21B4.4, T21H3.2, T22C1.7, T22D1.12, T23B3.4, T23C6.5, T23D8.1, T23G7.2, T26C12.4, T27B2.1, W02D7.7, W03F11.2, W03F11.4, W05B5.2, W05H12.1, W07G1.5, W10C4.1, Y105C5A.23, Y105C5B.2, Y110A2AL.8, Y113G7C.1, Y116A8B.5, Y18D10A.7, Y22D7AR.13, Y22D7AR.7, Y23H5B.4, Y34D9A.2, Y34D9B.1, Y37E11AL.5, Y38F1A.3, Y38H6C.20, Y39A1B.2, Y39A3B.5, Y39B6A.18, Y39B6A.30, Y40H4A.1, Y41D4A.8, Y41D4B.24, Y41G9A.4, Y47H9C.4, Y48B6A.10, Y48E1B.14, Y48G1C.5, Y4C6A.2, Y53C12A.1, Y53F4B.28, Y54E2A.1, Y54E2A.12, Y54E5B.1, Y54G2A.25, Y54G2A.35, Y55D5A.5, Y55F3AL.1, Y57A10C.10, Y58G8A.4, Y59H11AL.1, Y65B4BR.3, Y69A2AR.19, Y69E1A.3, Y70D2A.1, Y71F9B.5, Y73C8B.4, Y80D3A.7, Y80D3A.8, ZC190.2, ZC239.7, ZC374.1, ZC412.1, ZC412.2, ZC434.6, ZC504.5, ZC506.4, ZC84.4, ZK1067.1, ZK1086.1, ZK1307.7, ZK180.1, ZK270.1, ZK377.2, ZK418.6, ZK418.7, ZK455.2, ZK455.3, ZK524.1, ZK622.2, ZK643.3, ZK675.1, ZK792.7, ZK813.5, ZK896.8, ZK938.5, ZK945.9, ZK970.5, ZK970.6

**TRAFFICKING**

**(10) domain:t-SNARE (SM00397)** helical region found in SNAREs

C15C7.1, F35C8.4, F36F2.4, F48F7.2, F55A11.2, F56A8.7, T01B11.3, VF39H2L.1, Y57G11C.4, ZC155.7

**(8) domain: Synaptobrevin (PF00957)**

B0513.9, C30A5.5, F23H12.1, F55A4.1, T10H9.4, T14D7.3, Y69A2AR.6, ZK795.4

**(5) gene:snt-* (added D2092.1, T12A2.15) SyNapTotagmin**

D2092.1, F31E8.2, T10B10.5, T12A2.15, T23H2.2

**(85) GO:endocytosis** (including all (79) receptor-mediated endocytosis)

B0361.8, B0416.5, C01A2.3, C01F1.2, C02C2.3, C03C10.3, C08B11.4, C17E4.9, C18E9.10, C26H9A.1, C31E10.7, C34B2.10, C44H4.2, C47E12.2, C56C10.13, D2089.2, EGAP2.3, F10C5.2, F10D2.9, F21H12.4, F25D7.1, F28D1.9, F31E8.2, F33D4.2, F36H1.2, F40G9.1, F49B2.6, F54D1.6, F54F2.1, F54G8.3, F55A11.2, F57B10.10, F58A3.2, F58G11.1, H04J21.3, H19M22.2, JC8.5, K01H12.2, K03F8.2, K07D8.1, K09H9.6, K11D9.2, M02B1.1, R107.8, R10E11.2, R10E11.8, R12E2.2, R13A5.1, T01B11.3, T01B11.4, T01H3.1, T05H4.4, T05H4.5, T07C4.7, T08D2.2, T11F8.3, T14G10.7, T27E9.1, VZK822L.1, W01A8.4, W02A2.1, W02B9.1, Y110A7A.11, Y11D7A.9, Y25C1A.7, Y37D8A.10, Y38F2AR.7, Y39E4B.7, Y47D7A.16, Y49E10.11, Y53C12A.1, Y55H10A.1, Y56A3A.22, Y56A3A.32, Y60A3A.14, Y67D8C.10, Y73C8B.4, Y82E9BR.3, ZK637.8, ZK675.1, ZK686.3, ZK792.2, ZK792.3, ZK858.7

**(4) GO:exocytosis**

B0244.2, F31E8.2, T08A9.3, T10H9.4

**(26) GO:vesicle**

B0244.2, B0513.9, C05E11.1, C30A5.5, C42D8.8, C48A7.2, C48D1.3, F21G4.2, F23H12.1, F29D11.1, F31E8.2, F41C3.4, F42G8.11, F45D3.5, F55A4.1, K01A11.4, R13A5.1, T08A9.3, T10H9.4, T11F8.3, T13F2.8, T14D7.3, W01C8.6, Y47H9C.4, Y69A2AR.6, ZK795.4

**(8) GO:SNAP_receptor_activity**

C15C7.1, F35C8.4, F36F2.4, F48F7.2, F55A11.2, F56A8.7, T01B11.3, T10H9.4

**(14) GO:”secretion”** (secretion_by_cell, acetylcholine_secretion, …)

C31E10.7, C54H2.5, F31E8.2, F41C3.4, F54F2.1, F55A11.2, F58A3.2, R12E2.2, T05H4.4, T05H4.5, W02B8.6, W08D2.4, Y51A2D.19, Y56A3A.32

**(13) from other groups, not grouped in any of above**

B0272.2, C30B5.2, C56A3.7, D2013.10, F08F8.8, F41H10.11, M01D7.2, M03E7.5, T07A5.2, T07F10.4, Y59E9AL.7, Y71A12C.2, Y71F9B.3, D2013.10, T07F10.4, Y71A12C.2

**(1) from "no other domain", not grouped in any of above**

F56H1.1

**(137) TRAFFICKING combined**

B0244.2, B0272.2, B0361.8, B0416.5, B0513.9, C01A2.3, C01F1.2, C02C2.3, C03C10.3, C05E11.1, C08B11.4, C15C7.1, C17E4.9, C18E9.10, C26H9A.1, C30A5.5, C30B5.2, C31E10.7, C34B2.10, C42D8.8, C44H4.2, C47E12.2, C48A7.2, C48D1.3, C54H2.5, C56A3.7, C56C10.13, D2013.10, D2089.2, D2092.1, EGAP2.3, F08F8.8, F10C5.2, F10D2.9, F21G4.2, F21H12.4, F23H12.1, F25D7.1, F28D1.9, F29D11.1, F31E8.2, F33D4.2, F35C8.4, F36F2.4, F36H1.2, F40G9.1, F41C3.4, F41H10.11, F42G8.11, F45D3.5, F48F7.2, F49B2.6, F54D1.6, F54F2.1, F54G8.3, F55A11.2, F55A4.1, F56A8.7, F56H1.1, F57B10.10, F58A3.2, F58G11.1, H04J21.3, H19M22.2, JC8.5, K01A11.4, K01H12.2, K03F8.2, K07D8.1, K09H9.6, K11D9.2, M01D7.2, M02B1.1, M03E7.5, R107.8, R10E11.2, R10E11.8, R12E2.2, R13A5.1, T01B11.3, T01B11.4, T01H3.1, T05H4.4, T05H4.5, T07A5.2, T07C4.7, T07F10.4, T08A9.3, T08D2.2, T10B10.5, T10H9.4, T11F8.3, T12A2.15, T13F2.8, T14D7.3, T14G10.7, T23H2.2, T27E9.1, VF39H2L.1, VZK822L.1, W01A8.4, W01C8.6, W02A2.1, W02B8.6, W02B9.1, W08D2.4, Y110A7A.11, Y11D7A.9, Y25C1A.7, Y37D8A.10, Y38F2AR.7, Y39E4B.7, Y47D7A.16, Y47H9C.4, Y49E10.11, Y51A2D.19, Y53C12A.1, Y55H10A.1, Y56A3A.22, Y56A3A.32, Y57G11C.4, Y59E9AL.7, Y60A3A.14, Y67D8C.10, Y69A2AR.6, Y71A12C.2, Y71F9B.3, Y73C8B.4, Y82E9BR.3, ZC155.7, ZK637.8, ZK675.1, ZK686.3, ZK792.2, ZK792.3, ZK795.4, ZK858.7

**(65) TRAFFICKING combined, curated** (removed 22 TRANSPORTERS, 26 METABOLIC ENZYMES, 11 SIGNALING, 2 ADHESION, 11 ETC)

B0244.2, B0272.2, B0513.9, C01A2.3, C05E11.1, C15C7.1, C18E9.10, C30A5.5, C30B5.2, C31E10.7, C44H4.2, C54H2.5, C56A3.7, D2013.10, D2092.1, F08F8.8, F10C5.2, F23H12.1, F25D7.1, F29D11.1, F31E8.2, F35C8.4, F36F2.4, F40G9.1, F41C3.4, F41H10.11, F42G8.11, F48F7.2, F55A11.2, F55A4.1, F56A8.7, F56H1.1, F57B10.10, F58G11.1, M01D7.2, M03E7.5, R12E2.2, R13A5.1, T01B11.3, T07A5.2, T07F10.4, T08A9.3, T08D2.2, T10B10.5, T10H9.4, T11F8.3, T12A2.15, T13F2.8, T14D7.3, T23H2.2, VF39H2L.1, W02B8.6, Y110A7A.11, Y11D7A.9, Y25C1A.7, Y47D7A.16, Y56A3A.22, Y57G11C.4, Y59E9AL.7, Y69A2AR.6, Y71A12C.2, Y71F9B.3, ZC155.7, ZK795.4, ZK858.7

**CELL ADHESION**

**Based on Cox et al., 2004; known or predicted to be involved in adhesion**

**(8) domain:IG (not all IG proteins, but ones known to be involved in adhesion)** immunoglobulin

C18F3.2, C26G2.1, F02G3.1, F39H12.4, K02E10.8, SSSD1.1, Y42H9B.2, Y54G2A.25

**(12) domain:CA(PF00028, SM00112)** cadherin

B0034.3, C45G7.5, F08B4.2, F15B9.7, F18F11.3, F25F2.2, F59C12.1, R05H10.6, R10F2.1, W02B9.1, Y71D11A.1, ZK112.7

**(19) claudin homologs** (C09F12.1, C18D1.4, F12D9.1, F53B3.5, F59C6.11, T05A10.2, T22C8.8, T28B4.4, ZK563.4 from no other domain)

C01C10.1, C01C10.4, C09F12.1, C18D1.4, C24H10.1, F10A3.1, F12D9.1, F44G3.10, F53B3.5, F59C6.11, K10D6.2, R04F11.1, T05A10.2, T05E11.2, T22C8.8, T28B4.4, Y38F2AL.1, Y67A10A.9, ZK563.4

**(10) domain:clc-like (PF07062)** (added F10A3.1, F44G3.10)

C01C10.1, C01C10.4, C24H10.1, F10A3.1, F44G3.10, K10D6.2, R04F11.1, T05E11.2, Y38F2AL.1, Y67A10A.9

**(5) gene:clc-***

C01C10.1, C01C10.4, C09F12.1, T05A10.2, ZK563.4

**(8) domain:laminin G (PF00054, PF02210, SM00210, SM00282)**

C29A12.4, F11C7.4, F15B9.7, F20B10.1, F25F2.2, W02B9.1, W03D8.6, ZK112.7

**(27) GO:cell adhesion**

B0034.3, C03F11.3, C18F3.2, C25F6.4, C32D5.2, C45G7.5, F02G3.1, F07A5.3, F08B4.2, F11C1.3, F11D5.3, F15B9.7, F18F11.3, F25F2.2, F54F2.1, F54G8.3, F59C12.1, R05H10.6, R07B1.3, R10F2.1, W02B9.1, Y37D8A.13, Y49E10.20, Y71D11A.1, Y76A2B.6, ZK1058.2, ZK112.7

**(11) from other groups, not grouped in any of above**

C54G4.4, F11C7.4, F54D1.6, F57C7.3, H19M22.2, H30A04.1, K03H1.5, K07D8.1, K08E5.3, R09E10.5, T21B6.1

**(66) CELL ADHESION combined**

B0034.3, C01C10.1, C01C10.4, C03F11.3, C09F12.1, C18D1.4, C18F3.2, C24H10.1, C25F6.4, C26G2.1, C29A12.4, C32D5.2, C45G7.5, C54G4.4, F02G3.1, F07A5.3, F08B4.2, F10A3.1, F11C1.3, F11C7.4, F11D5.3, F12D9.1, F15B9.7, F18F11.3, F20B10.1, F25F2.2, F39H12.4, F44G3.10, F53B3.5, F54D1.6, F54F2.1, F54G8.3, F57C7.3, F59C12.1, F59C6.11, H19M22.2, H30A04.1, K02E10.8, K03H1.5, K07D8.1, K08E5.3, K10D6.2, R04F11.1, R05H10.6, R07B1.3, R09E10.5, R10F2.1, SSSD1.1, T05A10.2, T05E11.2, T21B6.1, T22C8.8, T28B4.4, W02B9.1, W03D8.6, Y37D8A.13, Y38F2AL.1, Y42H9B.2, Y49E10.20, Y54G2A.25, Y67A10A.9, Y71D11A.1, Y76A2B.6, ZK1058.2, ZK112.7, ZK563.4

**(58) CELL ADHESTION combined, curated** (removed 7 SIGNALING, 1 ENZYME)

B0034.3, C01C10.1, C01C10.4, C03F11.3, C09F12.1, C18D1.4, C18F3.2, C24H10.1, C29A12.4, C45G7.5, C54G4.4, F02G3.1, F07A5.3, F08B4.2, F10A3.1, F11C1.3, F11C7.4, F12D9.1, F18F11.3, F20B10.1, F25F2.2, F39H12.4, F44G3.10, F53B3.5, F54D1.6, F54F2.1, F54G8.3, F57C7.3, F59C12.1, F59C6.11, H19M22.2, H30A04.1, K03H1.5, K07D8.1, K08E5.3, K10D6.2, R04F11.1, R05H10.6, R07B1.3, R09E10.5, R10F2.1, SSSD1.1, T05A10.2, T05E11.2, T21B6.1, T22C8.8, T28B4.4, W02B9.1, W03D8.6, Y38F2AL.1, Y42H9B.2, Y49E10.20, Y67A10A.9, Y71D11A.1, Y76A2B.6, ZK1058.2, ZK112.7, ZK563.4

**ECM COMPONENTS**

**(34) cuticlin** (ZK783.1 from no other domain)

C29E6.4, C34G6.6, C43C3.3, C47G2.1, E04D5.3, F10E7.10, F13B9.6, F20D1.8, F22B5.3, F41A4.1, F47G9.3, F52B11.3, F53B6.6, F53F1.1, F55A4.10, K06A1.3, M01E10.2, M142.2, M28.1, R07E3.3, T04F8.4, T21B10.6, T21H3.4, T22C8.7, T23F1.5, T24C2.1, W01A8.3, W04C9.3, W06D12.1, Y38C1AA.5, Y53H1B.1, Y55D5A.6, Y55F3C.7, ZK783.1

**(31) domain:CT (PF00100, SM00241)**

C29E6.4, C34G6.6, C43C3.3, C47G2.1, E04D5.3, F10E7.10, F13B9.6, F20D1.8, F22B5.3, F47G9.3, F52B11.3, F53B6.6, F53F1.1, K06A1.3, M01E10.2, M142.2, M28.1, R07E3.3, T04F8.4, T21B10.6, T21H3.4, T22C8.7, T23F1.5, T24C2.1, W01A8.3, W04C9.3, W06D12.1, Y38C1AA.5, Y53H1B.1, Y55D5A.6, Y55F3C.7

**(22) gene:cutl-*** (F55A4.10 from no other domain)

C29E6.4, F10E7.10, F13B9.6, F20D1.8, F41A4.1, F47G9.3, F53B6.6, F53F1.1, F55A4.10, K06A1.3, M28.1, T04F8.4, T21B10.6, T21H3.4, T22C8.7, W01A8.3, W04C9.3, W06D12.1, Y38C1AA.5, Y53H1B.1, Y55D5A.6, Y55F3C.7

**(11) gene:col-*** (added Y69H2.14)

C34F6.2, C34F6.3, F33A8.9, F38A3.1, F54B11.1, F59F3.2, T13B5.4, W07A12.5, Y11D7A.11, Y18H1A.12, Y69H2.14

**(5) GO:molting_cycle_collagen_and_cuticulin-based_cuticle**

B0024.14, B0348.1, F53B7.4, M88.6, T23G11.6

**(2) GO:collagen_and_cuticulin-based_cuticle_development**

F35C8.7, Y57G11C.31

**(1) from other groups, not grouped in any of above**

F11C3.2

**(1) from "no other domain", not grouped in any of above**

ZC328.1

**(54) ECM COMPONENTS, combined**

B0024.14, B0348.1, C29E6.4, C34F6.2, C34F6.3, C34G6.6, C43C3.3, C47G2.1, E04D5.3, F10E7.10, F11C3.2, F13B9.6, F20D1.8, F22B5.3, F33A8.9, F35C8.7, F38A3.1, F41A4.1, F47G9.3, F52B11.3, F53B6.6, F53B7.4, F53F1.1, F54B11.1, F55A4.10, F59F3.2, K06A1.3, M01E10.2, M142.2, M28.1, M88.6, R07E3.3, T04F8.4, T13B5.4, T21B10.6, T21H3.4, T22C8.7, T23F1.5, T23G11.6, T24C2.1, W01A8.3, W04C9.3, W06D12.1, W07A12.5, Y11D7A.11, Y18H1A.12, Y38C1AA.5, Y53H1B.1, Y55D5A.6, Y55F3C.7, Y57G11C.31, Y69H2.14, ZC328.1, ZK783.1

**Other proteins**

**(56) domain:Znf (PF01428, PF01529, PF02135, PF01363, PF00643, PF00569, PF00642, PF00320, PF00105, PF00641, PF00098, PF00628, PF00096, SM00064, SM00154, SM00184, SM00249, SM00251, SM00291, SM00336, SM00343, SM0352, SM00355, SM00356, SM00399, SM00401, SM00451, SM00547, SM00551, SM00744)** zinc finger

AC3.10, C16C10.5, C16C10.7, C17D12.1, C17E4.3, C17H11.6, C18B12.4, C43H6.7, C53D5.2, C56A3.4, D2021.2, D2089.2, F02C12.1, F07C3.10, F08B12.2, F08G12.5, F09B12.2, F10E7.2, F13E6.3, F15H10.4, F26E4.11, F33D11.12, F48G7.3, F55A3.1, F55A11.7, F55G1.6, F56H1.2, F58E6.12, F59C6.2, H10E21.5, H32C10.3, K02G10.1, M18.8, R11G11.12, R13F6.5, T02C1.2, T09A5.12, T19H12.8, T22E7.2, T24C4.7, W04B5.2, Y119C1B.5, Y38F1A.2, Y39E4B.7, Y45G12B.2, Y47D3B.11, Y47G6A.31, Y47H9C.2, Y49F6B.9, Y4C6A.3, Y53G8AM.4, Y54F10BM.6, Y57A10B.1, ZC13.1, ZK757.4, ZK930.2

**(22) domain:EGF (PF00008, PF07974, PF07645, SM00001, SM00179, SM00181)** epidermal growth factor-like

B0393.5, C23H4.8, C37C3.7, D1044.2, F09E8.2, F25D7.5, F28E10.2, F33C8.1, F35D2.3, F40A3.2, F48C5.1, F55H12.3, F58G4.4, F58H1.6, K06A9.3, R11G1.1, R13F6.4, T13C2.6, T21E3.3, T25F10.3, W02C12.1, Y70G10A.2

**(23) c-type lectins**

C25B8.4, C43H6.6, E03H12.3, F25D7.5, F48C11.2, F49A5.2, F49A5.3, F49A5.5, F49A5.7, F49A5.9, F55H12.3, R10D12.5, T20B3.13, T20B3.15, T25E12.10, T25E12.7, T25E12.8, T25E12.9, W10G11.5, Y102A5B.1, Y18D10A.12, Y50D4B.5, Y70G10A.2

**(21) domain:CL (PF00059, SM00034)**

C25B8.4, C43H6.6, F25D7.5, F48C11.2, F49A5.2, F49A5.3, F49A5.5, F49A5.7, F49A5.9, F55H12.3, T20B3.13, T20B3.15, T25E12.10, T25E12.7, T25E12.8, T25E12.9, W10G11.5, Y102A5B.1, Y18D10A.12, Y50D4B.5, Y70G10A.2

**(18) gene:clec-***

C25B8.4, E03H12.3, F49A5.2, F49A5.3, F49A5.5, F49A5.7, F49A5.9, R10D12.5, T20B3.13, T20B3.15, T25E12.10, T25E12.7, T25E12.8, T25E12.9, W10G11.5, Y102A5B.1, Y18D10A.12, Y50D4B.5

**(22) tetraspannin** (added F59G1.2, Y39B6A.6 from no other domain)

B0198.1, B0563.2, C02F12.1, C02F5.11, C02F5.8, C17G1.8, C25G6.2, C54D10.4, D2092.7, F01E11.4, F33C8.3, F39C12.3, F53B2.2, F53B6.1, F59G1.2, T05C12.9, T14B4.4, T14G10.6, T23D8.2, Y39B6A.6, Y39E4B.4, Y45F10B.1

**(20) domain:tetraspannin (PF00335)**

B0198.1, B0563.2, C02F12.1, C02F5.11, C02F5.8, C17G1.8, C25G6.2, C54D10.4, D2092.7, F01E11.4, F33C8.3, F39C12.3, F53B2.2, F53B6.1, T05C12.9, T14B4.4, T14G10.6, T23D8.2, Y39E4B.4, Y45F10B.1

**(20) gene:tsp-***

B0198.1, B0563.2, C02F12.1, C02F5.11, C02F5.8, C17G1.8, C25G6.2, D2092.7, F01E11.4, F33C8.3, F39C12.3, F53B2.2, F53B6.1, F59G1.2, T14B4.4, T14G10.6, T23D8.2, Y39B6A.6, Y39E4B.4, Y45F10B.1

**(18) domain:Ankyr (PF00023, SM00248)** ankyrin repeat

C01G10.1, C18H2.1, C18H2.3, C18H2.5, C49G7.1, D2021.2, F26D2.10, F31B12.3, F36D3.5, F37A4.4, F40E12.2, F56D5.9, H32C10.3, K02F6.4, K09D9.11, R31.2, T08G3.7, Y67A10A.3

**(15) domain:BRCT (PF00533, SM00292)** BRCA1 C Terminus (BRCT) domain

C01G10.1, C06G3.4, C18H2.1, C18H2.3, C18H2.5, C49G7.1, F26D2.10, F36D3.5, F37A4.4, F40E12.2, F56D5.9, F56H1.3, K02F6.4, K09D9.11, T08G3.7

**(13) domain:IG (PF07654, PF07679, PF07686, PF00047, PF08205, SM00406, SM00407, SM00408, SM00409, SM00410)** immunoglobulin domain

C25G4.10, F28D1.8, F28E10.2, F48C5.1, K09E2.4, K10C3.3, T02C5.3, T04A11.3, T21D12.9, T25D10.2, Y102A11A.8, Y32G9A.8, ZC262.3

**(12) domain:LITAF (SM00714)** Possible membrane-associated motif in LPS-induced tumor necrosis factor alpha factor

B0348.2, C08E8.1, F16F9.1, F36G3.3, Y22D7AL.15, Y37D8A.26, Y37D8A.6, Y37D8A.8, Y40H7A.11, Y41C4A.11, Y87G2A.18, Y87G2A.19

**(12) domain:RcpL (PF01030)** receptor L domain (not necessarily a receptor domain, since none of RcpL containing proteins have been characterized to have receptor function)

F02C9.4, F14D2.6, F28H7.6, F38A5.11, F42G8.9, F59D6.6, K04F1.12, R03G5.3, Y19D10B.5, Y46H3D.4, ZC482.3, ZC482.7

**(12) domain:LRR (PF00560, SM00369, SM00370)** Leucine-Rich Repeat

C44H4.1, F10F2.4, F20D1.7, F37E3.2, F56A8.3, K07A12.2, T01G9.3, T05A1.3, T21D12.9, T22E7.1, Y71F9B.8, ZK682.5

**(11) domain:CUB (PF00431, PF02408, SM00042)** CUB domain

C14C11.4, C43H6.6, F16B12.1, F33C8.1, F35E12.4, F38B2.3, H20E11.1, K03E5.1, K05C4.11, K10D11.5, ZK1037.6

**(10) domain:dnaJ (SM00271), gene:dnj-*** DnaJ molecular chaperone homology domain

B0035.2, C01G10.12, C01G8.4, C04A2.7, C56C10.13, F54F2.9, K02G10.8, T04A8.9, Y39C12A.8, Y63D3A.6

**(10) domain:Band_7 (PF01145, SM00244)** SPFH domain / Band 7 family

F08C6.4, F14D12.4, F32A6.5, F41G4.3, F52D10.5, F57H12.2, K03E6.5, T24H7.1, Y71H9A.2, Y71H9A.3

**(10) domain:Pmp3 (PF01679)** proteolipid membrane potential modulator

F25H5.8, R10D12.6, R10D12.7, T06C12.9, T23B3.2, T23F2.3, T23F2.5, W02A2.9, W10C8.6, ZK632.10

**(8) domain:TLC (SM00724)** TRAM, LAG1 and CLN8 homology domain

C09G4.1, F41H10.5, K02G10.6, K12H6.6, M02B1.3, Y48G8AL.13, Y63D3A.8, Y6B3B.10

**(8) domain:Frag1 (PF10277)** Frag1/DRAM/Sfk1 family

C14B9.3, C33A11.2, F11E6.6, T04A8.12, T23B12.5, W03A5.2, Y38F1A.8, ZK185.4

**(7) domain:MtN3_slv (PF03083)** MtN3/saliva family

C06G8.1, C54F6.4, K02D7.5, K06A4.4, K11D12.5, R10D12.9, Y39A1A.8

**(15) gene:fbxa-*,fbxb-*, domain:Fbox (PF00646, SM00256), Fba_2 (PF07735)**

C16C4.6, C47B2.1, C52E2.7, C56G7.2, F31F4.15, F36H5.5, F40G9.18, F40G9.9, F45C12.5, F48C1.2, T06E6.5, T26H2.2, Y22D7AR.2, Y46G5A.8, Y59E1A.1

**(12) gene:fip-*, fipr-*** (all from no other domain) Fungus-Induced Protein(-Related)

C12D8.14, C12D8.16, C12D8.17, C12D8.18, C12D8.19, C12D8.6, C37A5.2, C37A5.4, C50H2.12, F23D12.6, F23H12.8, F53B6.9

**(8) gene:cnc-*** (all from no other domain) CaeNaCin Caenorhabditis bacteriocin)

F53H2.2, R09B5.10, R09B5.13, R09B5.2, R09B5.3, R09B5.8, R09B5.9, R13D7.11

**(210) with gene name and/or description**

AC3.10, B0035.2, B0198.1, B0212.1, B0240.2, B0244.8, B0563.2, B0563.4, C01G10.12, C01G8.2, C01G8.4, C01H6.6, C02F12.1, C02F5.11, C02F5.8, C03G6.13, C04A2.7, C05D9.2, C09E8.3, C09F5.2, C09G4.1, C14F5.1, C16C10.5, C16C10.7, C16C4.13, C16C4.6, C17G1.8, C18D11.2, C24A11.8, C25B8.4, C25G6.2, C30A5.7, C33D12.2, C33H5.19, C41C4.7, C42D8.8, C46F11.1, C47B2.1, C48E7.5, C52A11.4, C52D10.12, C52E2.7, C56C10.13, cTel55X.1, D1044.3, D1065.1, D2013.8, D2021.2, D2092.7, D2096.2, E03H12.3, F01E11.4, F02E8.5, F07B10.1, F07C3.10, F07H5.2, F08B12.1, F08B12.2, F08C6.4, F08F1.7, F09B12.2, F09B9.3, F13G3.9, F14B8.7, F14D12.4, F15B9.2, F15D4.3, F15H10.4, F16A11.3, F20C5.2, F20D1.10, F22B7.10, F25D7.2, F26D10.11, F26E4.11, F26F4.4, F30A10.6, F31B12.3, F31D5.3, F31F4.15, F32A6.5, F33C8.1, F33C8.3, F34D6.4, F36H5.5, F36H9.4, F37C12.2, F38A5.10, F38A5.12, F38A5.14, F38A5.5, F38A5.9, F39C12.3, F39E9.12, F40F9.1, F40F9.2, F41G4.3, F42E11.2, F43G9.6, F45C12.5, F45C12.6, F45F2.1, F45F2.5, F45F2.6, F45F2.7, F46C5.8, F48C1.2, F48C11.2, F48G7.3, F49A5.2, F49A5.3, F49A5.5, F49A5.7, F49A5.9, F52D10.5, F53B2.2, F53B6.1, F53B7.5, F54B11.3, F55A3.1, F56F3.2, F56F4.1, F56H1.2, F57A8.2, F57H12.2, F58G4.4, F59B10.1, F59H6.4, H04J21.3, H04M03.2, H06I04.2, H19J13.1, H20J04.6, H22K11.4, K02G10.6, K02G10.8, K03E6.5, K07A1.8, K07F5.15, K09E2.4, K09E9.2, K09H9.6, K10C3.3, M01D7.6, R01B10.5, R01B10.6, R01H2.3, R04E5.2, R05D3.2, R07E5.13, R10D12.5, R11G11.12, R13F6.4, R13G10.4, T02C5.3, T04A8.12, T04A8.9, T04G9.3, T06E6.5, T07A5.6, T09A5.12, T10H10.1, T13H5.8, T14B4.4, T14G10.6, T16G12.5, T19B10.5, T19H12.8, T20B3.13, T20B3.15, T21C9.12, T21E3.3, T22H2.6, T22H9.2, T23D8.2, T24C4.7, T24H7.1, T25E12.10, T25E12.7, T25E12.8, T25E12.9, T26H2.2, T28D6.9, W01C9.3, W01G7.5, W03A5.2, W06A7.3, W06H3.1, W10G11.5, Y102A5B.1, Y18D10A.12, Y32H12A.5, Y39C12A.8, Y39E4B.4, Y41C4A.11, Y45F10B.1, Y47D3B.7, Y47H9C.2, Y50D4B.5, Y59E1A.1, Y63D3A.6, Y67A10A.8, Y6B3B.10, Y71H9A.2, Y71H9A.3, Y77E11A.4, ZC190.1, ZK265.9, ZK632.6, ZK686.3

**(263) with GO association** (other than "integral_to_membrane", "membrane", "intracellular")

B0244.8, B0303.4, B0336.11, B0393.5, B0464.6, B0546.5, B0554.7, C01G8.2, C02D5.2, C03B8.1, C03H5.6, C04E12.4, C04E12.5, C05D11.7, C08G5.1, C08G9.2, C09F5.2, C09G4.1, C13F10.5, C14B9.3, C14C11.4, C14F5.1, C15A11.7, C15H9.4, C16B8.4, C17D12.3, C18B12.6, C18D11.2, C18H2.1, C18H2.5, C24A11.8, C28H8.4, C30A5.7, C32A3.3, C33H5.19, C34B2.8, C34D10.1, C35D10.1, C36H8.1, C41G7.9, C42D8.8, C43H6.6, C44H4.1, C46F11.1, C52A11.4, C53A5.13, C56G2.1, cTel55X.1, D1007.5, D1044.2, D2013.8, D2096.2, F07B10.1, F07H5.2, F09B9.3, F09E8.2, F09F9.4, F10F2.4, F11A10.5, F11E6.6, F13G3.9, F14E5.2, F15B9.2, F16A11.3, F20C5.2, F22B7.10, F25D7.5, F26A1.8, F26F2.7, F26F4.4, F26F4.9, F28D1.8, F28H7.6, F30A10.6, F30F8.9, F31B12.3, F32A11.7, F32D1.3, F32D8.5, F33C8.1, F33D4.7, F34D10.2, F34D6.4, F35A5.5, F35D2.3, F35F10.10, F36H9.4, F37A4.4, F37C12.2, F37E3.2, F38A5.11, F38A5.14, F38A5.5, F38B6.6, F39E9.12, F42A8.3, F42G8.10, F43G9.2, F43G9.6, F44F4.1, F45C12.6, F45F2.7, F48C11.2, F52C6.4, F53B6.4, F53B7.5, F54B11.3, F55H12.3, F56A8.1, F56A8.3, F56B3.11, F56D5.9, F56F3.2, F56H1.3, F57B1.1, F58F6.5, F58G4.4, F58H1.6, F59B10.1, H04J21.1, H04J21.3, H06I04.2, H22K11.4, JC8.5, K02B12.3, K02D7.5, K02E10.4, K05C4.11, K05C4.2, K06A5.2, K07A1.8, K07A12.2, K07F5.12, K08F4.3, K09E2.4, K09G1.1, K09H9.6, K10D2.5, K11D12.5, K11H12.3, K12B6.2, M01D7.6, M01G5.3, M70.1, M70.3, R01H2.3, R05D3.2, R05H5.5, R07E5.13, R07E5.7, R08C7.2, R105.1, R10E4.9, R13F6.4, R13G10.4, R144.6, R151.6, R155.4, T01G9.3, T02C5.3, T03F1.12, T03F6.6, T04A8.12, T04G9.3, T05A1.3, T06D8.5, T06D8.7, T06D8.9, T07A5.6, T10E9.6, T10H10.1, T13C2.6, T14G10.7, T15B7.2, T16G12.5, T19B10.5, T19C3.4, T19D12.4, T20D3.6, T20D4.13, T20H9.6, T21C9.1, T21C9.12, T21D12.9, T21E3.3, T22C1.3, T24C4.4, T24C4.8, T24F1.2, T28D6.9, W01G7.5, W02B12.15, W02B3.6, W02B8.3, W02B8.4, W02C12.1, W02D3.4, W02D9.2, W02H5.4, W03D8.9, W04G5.5, W05G11.2, W06A7.3, W06H3.1, Y106G6H.8, Y18H1A.14, Y32H12A.5, Y37D8A.17, Y37D8A.26, Y37D8A.5, Y37D8A.6, Y37D8A.8, Y39A1A.22, Y39A1A.8, Y39A3A.3, Y39B6A.8, Y39D8B.1, Y41C4A.11, Y41D4A.4, Y43H11AL.2, Y46B2A.3, Y47D3B.7, Y47H9A.1, Y48E1B.2, Y50D4B.4, Y50D4B.7, Y53F4B.25, Y54G2A.2, Y55F3AR.1, Y55F3AR.2, Y57A10A.3, Y57E12AL.1, Y60A3A.19, Y63D3A.8, Y64G10A.6, Y66D12A.21, Y67A10A.8, Y67H2A.4, Y69A2AR.31, Y6B3B.10, Y70G10A.2, Y71F9B.8, Y71H2AM.2, Y75B8A.5, Y77E11A.4, Y87G2A.19, ZC190.1, ZC262.3, ZC328.3, ZC482.3, ZC482.7, ZK1010.6, ZK1053.7, ZK1236.7, ZK418.3, ZK418.5, ZK616.6, ZK632.10, ZK632.6, ZK682.5, ZK686.3, ZK757.1, ZK858.5

(233) nematode-specific domains (DUF*, CW, CX, DB, EB, MD, PAW, WSN)(E_BE45912.2 from "no other domain")

B0024.3, B0212.1, B0222.1, B0546.5, B0554.5, B0554.7, C01G10.1, C01G5.4, C01H6.6, C03B8.1, C04E12.4, C04E12.5, C06C6.7, C07A9.12, C08G5.1, C08G9.2, C09E8.3, C13A2.1, C13A2.5, C13A2.9, C16C8.17, C16D9.4, C17B7.3, C17D12.3, C17H12.2, C18H2.1, C18H2.5, C18H7.11, C25G4.10, C26B2.8, C30G12.4, C33D12.2, C33H5.1, C33H5.19, C34B4.5, C34D4.4, C35A5.5, C35D10.1, C38D9.5, C46F11.1, C47B2.1, C52D10.12, D1044.3, D1065.1, E03H12.3, E_BE45912.2, F01D5.10, F07B7.14, F07E5.8, F07G11.1, F07G11.3, F13A2.6, F14B8.7, F15H10.7, F15H10.8, F16D3.6, F19H8.5, F21C10.1, F21C10.3, F21C10.4, F21F3.7, F22B5.10, F22F7.3, F22F7.4, F25E5.2, F26D2.10, F26F2.7, F28B3.10, F28B3.6, F31D5.1, F31D5.2, F31F4.15, F32A11.7, F32D1.3, F32D8.5, F35D11.3, F35E2.1, F35F10.10, F36D1.8, F36D3.5, F36G9.13, F37A4.4, F38A1.11, F38A5.10, F38A5.12, F38A5.14, F38A5.5, F38A5.9, F38B6.6, F38B7.10, F38B7.11, F38C2.4, F40E10.6, F40E12.2, F45F2.1, F45F2.5, F45F2.6, F45F2.7, F47B8.5, F49C12.1, F49C12.4, F49H6.13, F49H6.3, F53B7.5, F53C3.3, F53C3.4, F53C3.5, F53C3.6, F54E2.5, F55B12.2, F55C12.5, F56H1.2, F56H1.3, F57B1.1, F57B1.8, F57B1.9, F57G4.1, F58A6.5, F58F6.5, F58F6.6, H04J21.1, H04M03.2, H19J13.1, H20J04.1, H20J04.6, K01D12.1, K01D12.6, K02E10.4, K02F6.3, K02F6.4, K03A11.4, K04A8.2, K05C4.2, K05F1.1, K07F5.12, K07F5.15, K08D9.6, K09D9.11, K09F6.4, K11G12.6, M01E5.1, M70.1, M70.3, R01B10.5, R02F11.2, R04E5.2, R05D11.5, R05D7.3, R07B7.12, R08B4.4, R10D12.5, R13G10.4, R144.6, R155.4, T04A11.3, T06D8.9, T06E6.5, T07A5.6, T09E8.4, T10E9.4, T11F9.21, T13H10.2, T14G12.6, T15D6.11, T16A1.2, T16A9.5, T16G12.5, T19A6.4, T20D4.13, T20H9.6, T24C4.4, T24C4.8, T24F1.2, T27C5.8, W02B8.3, W02B8.4, W02D3.4, W03D8.10, W04G5.5, W06A7.4, W06G6.7, W09B7.3, Y102A11A.6, Y105C5B.23, Y106G6H.8, Y11D7A.3, Y17G7B.19, Y18H1A.14, Y22D7AR.14, Y22D7AR.2, Y2H9A.4, Y34F4.1, Y37A1A.2, Y39B6A.27, Y39B6A.29, Y39B6A.8, Y39D8A.1, Y39D8B.1, Y39D8B.3, Y43F8B.10, Y45G12C.1, Y47D7A.14, Y47H9A.1, Y50D4B.4, Y50D4B.7, Y51A2B.2, Y52B11A.7, Y52D5A.1, Y52E8A.4, Y53F4B.25, Y57G11C.37, Y57G7A.6, Y59E1A.1, Y60C6A.1, Y62E10A.10, Y67A10A.3, Y69E1A.1, Y69H2.1, Y6B3B.3, ZC196.5, ZC196.8, ZC196.9, ZC262.10, ZC262.9, ZK1010.6, ZK1025.3, ZK1053.7, ZK1055.4, ZK381.8, ZK418.5, ZK6.8, ZK616.6, ZK757.1

**(49) from "no other domain", not grouped in any of above**

B0222.10, B0244.9, B0284.3, B0464.4, C02C6.2, C02E11.1, C03B1.12, C18E9.3, C23H4.1, C24A8.3, C26D10.5, C26D10.7, C36E8.3, C44B7.3, F07F6.7, F07F6.8, F07G6.1, F09E5.11, F13B9.8, F13H8.4, F35D11.2, F41G3.4, F46G11.1, F54F7.4, F55D12.6, F56C3.6, K01A2.1, K02F3.8, K09F5.1, R09B5.4, R11H6.3, T01H3.4, T06E4.5, T07E3.6, T14A8.1, T16A1.7, T24B1.1, W01D2.3, W02A2.5, Y23H5A.5, Y39E4B.3, Y50E8A.17, Y55B1BM.1, Y71F9AR.1, Y73B6BR.1, Y75B7AL.1, ZK1067.4, ZK381.4, ZK899.2

**(85) ungrouped**

B0416.1, C02F5.13, C09B9.8, C15H9.5, C25F6.7, C30B5.9, C30H6.5, C31E10.6, C34F6.10, C34F6.7, C36B7.6, C37C3.12, C37E2.2, C37E2.3, C47G2.4, C52B9.4, D1046.5, D2092.5, F11G11.9, F20C5.4, F20D1.1, F21A10.2, F27D4.7, F28H1.4, F31F7.2, F32D8.14, F35D2.4, F38E1.9, F43G9.13, F44E7.9, F47B3.3, F47G3.4, F48F7.8, F53B1.2, F53F10.8, F56B3.6, F57C7.4, F59F4.2, K04G2.9, K11C4.2, M02B7.4, M04G7.1, M05D6.5, M176.4, R11H6.2, R12C12.6, R166.2, R186.6, T02E1.7, T05B4.8, T07F12.2, T09A12.5, T11F9.12, T12B5.14, T13G4.3, T19A6.1, T19A6.3, T22A3.6, T25G12.6, T27F6.6, W02H5.5, W03G11.2, W09G3.8, Y108G3AL.2, Y110A2AR.1, Y113G7B.12, Y116A8C.9, Y119C1B.3, Y22D7AL.11, Y34D9A.8, Y38F2AR.9, Y42H9AR.2, Y48G1BM.9, Y53C10A.5, Y53C12A.3, Y54F10AL.1, Y54F10AM.7, Y57E12AM.1, Y59C2A.2, Y71G12B.23, Y73E7A.6, Y74C10AL.2, Y87G2A.13, Y97E10AR.6, ZK858.6

Reference

Jones AK, Davis P, Hodgkin J, Sattelle DB. The nicotinic acetylcholine receptor gene family of the nematode Caenorhabditis elegans: an update on nomenclature. Invert Neurosci. 2007. 7(2):129-31.

Salkoff L, Wei AD, Baban B, Butler A, Fawcett G, Ferreira G, Santi CM. Potassium channels in C. elegans. WormBook. 2005. 30:1-15.

Zhao Z, Sheps JA, Ling V, Fang LL, Baillie DL. Expression analysis of ABC transporters reveals differential functions of tandemly duplicated genes in Caenorhabditis elegans. J Mol Biol. 2004. 344(2):409-17.

Cox EA, Tuskey C, Hardin J. Cell adhesion receptors in C. elegans. J Cell Sci. 2004. 117(Pt 10):1867-70.
